# Metabolic traits in obesity and normal BMI in industrialised countries: a multi-country analysis of national population-based studies

**DOI:** 10.1016/S0140-6736(26)00758-0

**Published:** 2026-07-01

**Authors:** NCD Risk Factor Collaboration (NCD-RisC), Ysé d’Ailhaud de Brisis, Lakshya Jain, James E Bennett, Bin Zhou, Edward W Gregg, Ana Barradas-Pires, Rodrigo M Carrillo-Larco, Agnese Galeazzi, Fulvio Deo, Olivia N O’Driscoll, Yefeng Fan, Nayu Ikeda, Hsien-Ho Lin, Gretchen A Stevens, Wichai Aekplakon, Tuija Jääskeläinen, Pekka Jousilahti, Niina E Kaartinen, Young-Ho Khang, Kamlesh Khunti, Seppo Koskinen, Tiina Laatikainen, Jouni Lahti, Yi-Jing Lin, Wei-Cheng Lo, Annamari Lundqvist, Satu Männistö, Nareemarn Neelapaichit, Kyungwon Oh, Suvi Parikka, Suyeon Park, Roengrudee Patanavanich, Markku Peltonen, Markus Perola, Katri Sääksjärvi, Veikko Salomaa, Ching-Fen Shen, Kenji Shibuya, Paibul Suriyawongpaisal, Hanna K Tolonen, Yi-Ren Wang, Chao-Yu Yeh, Francesco Zaccardi, Paul W Franks, Goodarz Danaei, Majid Ezzati

**Affiliations:** Imperial College London, UK; RCSI University of Medicine and Health Sciences, Ireland; Imperial College London, UK; Emory University, USA; Imperial College London, UK; National Institutes of Biomedical Innovation, Health and Nutrition, Japan; National Taiwan University, Taiwan; World Health Organization, Switzerland; Mahidol University, Thailand; Finnish Institute for Health and Welfare, Finland; Seoul National University College of Medicine, Republic of Korea; University of Leicester, UK; Finnish Institute for Health and Welfare, Finland; University of Eastern Finland, Finland; Finnish Institute for Health and Welfare, Finland; Ministry of Health and Welfare, Taiwan; Taipei Medical University, Taiwan; Finnish Institute for Health and Welfare, Finland; Mahidol University, Thailand; Korea Disease Control and Prevention Agency, Republic of Korea; Finnish Institute for Health and Welfare, Finland; Korea Disease Control and Prevention Agency, Republic of Korea; Faculty of Medicine Ramathibodi Hospital, Thailand; Finnish Institute for Health and Welfare, Finland; University of Turku, Finland; Ministry of Health and Welfare, Taiwan; Medical Excellence JAPAN, Japan; Mahidol University, Thailand; Finnish Institute for Health and Welfare, Finland; Ministry of Health and Welfare, Taiwan; University of Leicester, UK; Lund University, Sweden; Queen Mary University of London, UK; Harvard TH Chan School of Public Health, USA; Imperial College London, UK; Imperial Global Ghana, Ghana; University of Ghana, Ghana

## Abstract

**Background:**

Effective treatments are available for obesity and for hypertension and hypercholesterolaemia, which mediate the cardiovascular and renal effects of obesity. Our aim was to compare blood pressure, cholesterol, and the use of antihypertensive and lipid-lowering medicines in people with obesity and normal weight and assess whether the BMI-associated excess risk has diminished.

**Methods:**

Our primary outcomes were mean systolic blood pressure (SBP), non-HDL cholesterol and HDL cholesterol, and the proportion of the participants who used antihypertensive and lipid-lowering medicines. We used data from 110 health surveys conducted from 1990 to 2024 with 978 425 participants aged 20–79 years sampled from national populations of seven countries: Japan, South Korea, Taiwan, Thailand, Finland, England, and the USA. We used graphical presentation and trend analysis to evaluate changes over time in these outcomes in participants in the normal BMI range (20·0 to <25·0 kg/m^2^), and changes in the difference between participants with obesity (separately for class I obesity [30·0 to <35·0 kg/m^2^] and class II and III obesity [BMI ≥35·0 kg/m^2^]) or overweight (25·0 to <30·0 kg/m^2^) and those in the normal BMI range.

**Findings:**

Mean non-HDL cholesterol and SBP declined over time, especially among those older than 40 years, with the notable exception of some sex–age groups in Thailand. When pooled across all countries, age groups, and obesity and overweight BMI ranges, the difference in mean non-HDL cholesterol with normal BMI became smaller by –0·05 mmol/L per decade (95% CI –0·07 to –0·03) for females and –0·07 mmol/L per decade (–0·09 to –0·05) for males. For SBP, the pooled estimate of change in the difference with normal BMI across all countries, age groups, and obesity and overweight BMI ranges was –0·7 mmHg per decade (95% CI –1·0 to –0·4) for females and –0·6 mmHg per decade (–0·9 to –0·4) for males. The declines were larger in individuals with obesity, especially class II and III obesity, than in normal BMI, leading to a convergence of these risk factors between obesity and normal BMI in people older than 40 years. As a result of these trends, in England, the USA, Thailand, South Korea, and Japan, older people with obesity often became indistinguishable from, or better off than, those with normal BMI in terms of non-HDL cholesterol and SBP. These trends accompanied a larger increase in the use of lipid-lowering and antihypertensive medicines in middle-aged and older people with obesity than in those with normal BMI. The pooled estimate for the increase in difference in lipid-lowering medicines compared with normal BMI across all countries, age groups, and obesity and overweight BMI ranges was 1·5 percentage points per decade (1·0–2·1) for females and 1·6 percentage points per decade (1·0–2·2) for males. For antihypertensive medicines, the pooled estimate was 0·7 percentage points per decade (0·3–1·0) for females and 2·0 percentage points per decade (1·3–2·8) for males. Mean HDL cholesterol increased more in people with normal BMI than those with obesity, leading to a divergence. For people younger than 40 years, there has been little change in the gap between those with obesity or overweight and those with normal BMI; young adults were rarely treated for high cholesterol or blood pressure regardless of their BMI.

**Interpretation:**

In industrialised countries, blood pressure and non-HDL cholesterol in older adults with obesity are increasingly similar to those with normal BMI, with higher use of antihypertensive and lipid-lowering medicines a possible driver of this convergence. There is nonetheless heterogeneity across countries in the extent of convergence. Young adults with obesity remain metabolically at higher risk than their counterparts with normal weight.

## Introduction

Obesity has increased in most countries since the last decades of the 20th century, although the pace of increase has varied substantially across countries.^1,2^ Obesity leads to higher blood pressure and dyslipidaemia (higher non-HDL cholesterol and lower HDL cholesterol).^3–6^ Blood pressure and non-HDL cholesterol mediate the cardiovascular impacts of obesity.^7^ HDL cholesterol is associated with lower cardiovascular risk, although this connection has not been established as causal.^8^

Blood pressure and cholesterol have determinants beyond obesity, including smoking; alcohol use; physical activity; and consumption of salt, fruits, vegetables, saturated versus unsaturated fats, and processed versus unprocessed carbohydrates. Some of these determinants have changed independently of trends in obesity. Additionally, clinical guidelines have progressively lowered thresholds for treatment initiation and the treatment targets for people with high blood pressure, high cholesterol, and high absolute cardiovascular risk.^9–12^ In addition to lowering blood pressure and cholesterol in entire populations,^13,14^ changes in diet and treatment might have had comparably larger effects on those with obesity as they are more likely to be screened, treated, or given diet and lifestyle advice. This effect would lead to convergence of metabolic traits between people with obesity and those with a normal BMI. In contrast, shared causes of obesity and high blood pressure or cholesterol (eg, higher consumption of processed carbohydrates) might have worsened the metabolic traits of those with obesity beyond the effects of excess weight, leading to divergence across BMI ranges.

There is little information on blood pressure and cholesterol levels (and how they have changed) for people with obesity compared with people with a normal BMI;^15–20^ this limits our ability to evaluate the clinical relevance of obesity^21^ and prioritise the treatment of obesity, high blood pressure and cholesterol, or their combination. We examined multi-decadal changes in blood pressure and cholesterol in people with obesity or overweight compared with people with normal BMI in industrialised countries in Asia, Europe, and North America. We also examined trends in the use of antihypertensive and lipid-lowering medicines as a potential mechanism for differential changes in blood pressure and cholesterol between obesity or overweight and normal BMI.

## Methods

### Overview

We analysed blood pressure and cholesterol in people with obesity, overweight, and normal BMI in seven industrialised countries in Asia (Japan, South Korea, Taiwan, Thailand), Europe (Finland, England) and North America (the USA). These countries had multiple nationally representative studies since 2000 or earlier with measured data on BMI, blood pressure, and cholesterol. Data were available for Australia, New Zealand, and Singapore, but were either not provided or not in the format required for this analysis.

Our outcomes were mean systolic blood pressure (SBP), non-HDL cholesterol and HDL cholesterol, and the proportion of the sample who used antihypertensive and lipid-lowering medicines. We report these outcomes in participants aged 20–79 years in the normal BMI range (20·0 to <25·0 kg/m^2^), and the difference between participants in the obesity or overweight BMI ranges and those in the normal BMI range. Class I obesity was defined as BMI of 30·0 to <35·0 kg/m^2^; class II and III obesity as BMI ≥35·0 kg/m^2^; and overweight as BMI of 25·0 to <30·0 kg/m^2^. We report these outcomes for females and males in the following age groups: 20–39 years (young adults), 40–59 years (middle-aged adults), and 60–79 years (older adults). Additional information on outcomes, BMI ranges, and age groups is provided in the appendix (pp 1–2).

The pooled analysis was approved by Imperial College Research Ethics Committee (reference number 7076633). The participating studies followed their institutional approval process at the time of data collection.

### Data

We used 110 studies from 1990 to 2024 with measurement of height, weight, and at least one of blood pressure and cholesterol in representative samples of the national populations. Data inclusion and exclusion criteria and detailed information on the included studies are stated in the appendix (pp 3–5, 9–11). These studies together had 978 425 participants aged 20–79 years. Data cleaning steps are detailed in the appendix (pp 3–5, 25–26).

### Statistical analysis

All outcomes were calculated by study, sex, 10-year age band, and BMI range (normal BMI, overweight, class I obesity, and class II and III obesity). The calculations accounted for sampling weights and complex survey design when applicable. We calculated age-standardised means (for SBP and non-HDL and HDL cholesterol) or proportions (for medicine use) for 20-year age groups using WHO standard population weights^22^ to account for different age structures of populations across countries and time.

Participants from each study–year were split into up to 24 units (two sexes, three 20-year age groups, and four BMI ranges). With the exception of older adults in Finland, which were missing from some studies as detailed in the appendix (pp 3–5), all these units in each study–year were represented in the data; however, some had a small number of participants, which can result in unstable estimates. To ensure robust trend analysis, we removed any of the 24 sex–age–BMI-specific time series in a country if fewer than three units in the time series had 25 or more participants (appendix p 12). Most of the excluded time series were class II and III obesity in countries in Asia due to low obesity prevalence.

In each country, sex, and age group, we analysed change in outcomes for normal BMI and change in the difference in outcomes between obesity or overweight and normal BMI. To estimate change in outcomes in the normal BMI range, we fitted a linear regression with time as the independent variable to the entire time series for each country–sex–age group. We used a logit transformation on the proportion of the sample who used medicines to avoid negative predictions. We used the regression coefficients to calculate the outcomes for the earliest and latest years of data (appendix p 12) and total change between these 2 years. For obesity and overweight, we calculated the differences in outcomes compared with the normal BMI range and fitted a linear regression to these differences against time for each country–sex–age group. We also calculated change per decade, to make consistent comparisons across countries when data were available for different time periods.

We used a simulation approach to account for the sampling uncertainty of the survey data when age standardising and fitting the regressions. Specifically, we generated 10 000 draws from the uncertainty distributions of each datapoint. Uncertainty was represented by a normal distribution for means and a binomial distribution for proportions. For binomial distributions, we used effective sample sizes that accounted for the complex survey design instead of the nominal sample sizes. Binomial uncertainty shrinks to zero when the proportion is 0 or 1, even for small sample sizes. To avoid excessive leverage from these datapoints, we used additive smoothing, with uncertainty represented as a beta distribution.^23,24^ We then age-standardised these simulated outcomes into the three 20-year age bands and fitted a linear regression to each of the 10 000 simulated age-standardised datasets. We also calculated the difference in outcomes between obesity or overweight and normal BMI in each of the 10 000 simulated datasets and fitted a separate linear regression for each.

We report the median of the 10 000 estimates of outcomes for normal BMI and of the 10 000 estimated differences between obesity or overweight and normal BMI. We estimated the probability (*P*) that an estimated increase in an outcome represents a true increase as the proportion of the slope coefficients that were greater than zero, and vice versa for an estimated decrease. Therefore, an increase as well as a decrease with high certainty would both have *P* close to 1. The reported 95% CIs represent the 2·5th–97·5th percentiles of the 10 000 simulated estimates.

We pooled the estimated change per decade in the difference in outcomes between obesity or overweight BMI ranges and the normal BMI range across countries using a random-effect model with inverse variance weights.^25^ We assessed heterogeneity across countries with the *I*^*2*^ statistic.

We conducted sensitivity analyses to examine whether our conclusions were affected by using a linear trend model and exclusion of datapoints with fewer than 25 participants (appendix pp 6–7). All analyses were performed in R (version 4.5.2).

### Role of the funding source

The funders of the study had no role in study design, data collection, data analysis, data interpretation, or writing of the report.

## Results

According to the systematic analysis of worldwide data on obesity,^1,2^ among the seven countries we studied, obesity prevalence was highest in the USA, followed by the UK, and it was lowest in Japan and South Korea (appendix pp 27–28). The range of prevalence in 2024 across sex–age groups was 35·7–47·2% in the USA and 3·2–9·6% in Japan. For class II and III obesity, the range in 2024 was 14·6–27·3% in the USA and 0·5–2·3% in Japan. The prevalence of obesity increased in all seven countries and all age groups. The largest increase from 1990 to 2024 was in the USA, ranging 21–26 percentage points across sex–age groups, and the smallest in Japan, ranging 1–8 percentage points.

During the analysis period, mean non-HDL cholesterol declined in the normal BMI range in all ages and both sexes in England, Finland, and the USA, with the change ranging from –0·5 to –1·3 mmol/L for females (*P*>0·999 in all age–country combinations) and –0·4 to –1·3 mmol/L for males (*P*>0·999; figure 1; appendix pp 33–36). After accounting for differences in the earliest and latest year of data, these declines amounted to –0·1 to –0·6 mmol/L per decade across sex and age groups in these three countries. Mean non-HDL cholesterol also declined in older adults in South Korea and Taiwan (*P*>0·999 in all sex–country combinations). In young and middle-aged adults in these two countries, and in Japan and Thailand, there was no or little change in mean non-HDL cholesterol in the normal BMI range, with the change having a mix of positive and negative directions.

Mean HDL cholesterol increased in normal BMI in England by 0·26 to 0·52 mmol/L across sex–age combinations (*P*>0·999; figure 1; appendix pp 33–36). Mean HDL cholesterol also increased in Japan, South Korea, and Thailand, with the change ranging from 0·16 to 0·42 mmol/L for females and 0·14 to 0·28 mmol/L for males (*P*>0·999 in all sex–age–country combinations). The increase in Finland and the USA was smaller than in the other countries. In Taiwan, there was no or very little change in mean HDL cholesterol.

Change in mean SBP in normal BMI was negative in all sex–age combinations in six countries, with larger decreases in older adults. The exception was Thailand, where mean SBP increased by 6 to 12 mmHg in five of the six sex–age groups (*P*>0·999; figure 1; appendix pp 33–36). Mean SBP decreased in Japan, South Korea, and England by –3 to –25 mmHg (*P*>0·999 in all sex– age–country combinations); the range of decadal decrease was –1 to –9 mmHg per decade. The decrease in mean SBP was smaller in Finland, the USA, and Taiwan, and not detectable at *P* of 0·975 in the sex–age groups younger than 60 years in the USA.

People with obesity or overweight in these seven countries had larger declines in mean non-HDL cholesterol than those with normal BMI, leading to a convergence in mean non-HDL cholesterol (figure 2; appendix pp 29–32). When pooled across all countries, age groups, and obesity and overweight BMI ranges, the difference with normal BMI shrank by –0·05 mmol/L per decade (95% CI –0·07 to –0·03) for females and –0·07 mmol/L per decade (–0·09 to –0·05) for males (figure 3).

The largest reductions in difference were those in older adults, by –0·09 mmol/L per decade (95% CI –0·13 to –0·05) for females and –0·10 mmol/L per decade (–0·14 to –0·06) for males when pooled across all countries and obesity and overweight BMI ranges (figure 3). Within this age group, the largest reductions were for class II and III obesity in older females (–0·17 mmol/L per decade [–0·25 to –0·09]), and class I obesity in older males (–0·15 mmol/L per decade [–0·29 to –0·02]). The *I*^*2*^ statistic across all countries, age groups, and BMI ranges was 48·4% for females and 42·6% for males, which suggests moderate heterogeneity.

In England and the USA, the decline in mean non-HDL cholesterol in older adults with class II and III obesity was such that, by the end of the analysis period, their mean non-HDL cholesterol was below that of those with normal BMI (figure 2; appendix pp 29–32, 37–40). In Asian countries, mean non-HDL cholesterol in class I obesity, and in some cases overweight, also became lower than in the normal BMI range in older adults (except for females in Taiwan).

The predominant trend in middle-aged adults was also convergence. Across countries, the pooled estimates were –0·10 mmol/L per decade (95% CI –0·15 to –0·05) for females and –0·13 mmol/L per decade (–0·20 to –0·06) for males in class II and III obesity (figure 3). In this age group, the extent of convergence towards normal BMI increased with increasing BMI (figure 2; appendix pp 29–32). Nonetheless, mean non-HDL cholesterol in obesity and overweight remained higher than in normal BMI by the latest year of data (figure 2; appendix pp 29–32, 37–40). In young adults, the gap between obesity or overweight and normal BMI decreased by smaller amounts than in middle-aged and older adults (figure 2; appendix pp 29–32) and it might have even increased in the case of Taiwan. Most of these changes were not detectable at *P* of 0·975.

In contrast to non-HDL cholesterol, mean HDL cholesterol in obesity and overweight mainly diverged from (ie, moved further below) mean HDL cholesterol in normal BMI (figure 2; appendix pp 29–32). When pooled across all countries, age groups, and obesity and overweight BMI ranges, the mean HDL cholesterol difference with normal BMI changed by –0·04 mmol/L per decade (95% CI –0·05 to –0·03) for females and by –0·01 mmol/L per decade (–0·02 to –0·01) for males (figure 3). In contrast to non-HDL cholesterol, the size of the change did not vary systematically across age groups.

The gap in mean SBP between obesity or overweight and normal BMI became smaller (ie, a convergence) in most sex–age combinations and countries (figure 2; appendix pp 29–32). The main exception was Taiwan, where there was a divergence in mean SBP in relation to BMI. The pooled estimate across all countries, age groups, and obesity and overweight BMI ranges was –0·7 mmHg per decade (95% CI –1·0 to –0·4) fo females and –0·6 mmHg per decade (–0·9 to –0·4) for males (figure 3). The *I*^*2*^ statistic across all countries, age groups, and BMI ranges was 72·2% for females, which suggests substantial heterogeneity; the *I*^*2*^ statistic was 55·8% for males, which suggests moderate heterogeneity.

Convergence of mean SBP towards normal BMI was larger for those with obesity than those with overweight, and larger in older adults than those younger than 60 years (figure 2; appendix pp 29–32). The largest reductions were seen in class I obesity in older females (–1·8 mmHg per decade [95% CI –2·4 to –1·1]) and in class II and III obesity in older males (–2·3 mmHg per decade [–3·6 to –1·1]; figure 3). In older adults in the USA, mean SBP in those with obesity and overweight dropped below the levels in the normal BMI range by 2022; in most other countries, despite convergence, it remained above that of normal BMI by the latest year of data (figure 2; appendix pp 29–32, 37–40).

The results were also consistent with the continuous relationship between BMI and non-HDL cholesterol or SBP (appendix pp 110–115). Specifically, these associations attenuated over time in older adults but remained positive in young adults.

The proportion of participants who used lipid-lowering medicines was below 13% for all BMI and age groups before 2000. The proportion of older adults with normal BMI who used lipid-lowering medicines increased (*P*>0·999; figure 4; appendix pp 44–46). The increase was largest in South Korea, Thailand, England, and the USA, by up to 49 percentage points for females in South Korea (*P*>0·999, 95% CI 45–53 percentage points) and 38 percentage points for males in the USA (*P*>0·999, 28–47 percentage points; appendix pp 44–46); the increase was smaller in Japan and Taiwan (8 to 11 percentage points). At the end of the analysis period, the proportion of older adults with normal BMI who used lipid-lowering medicines was 16–53% for females and 12–48% for males, with Taiwan having the lowest treatment rate (in both sexes) and South Korea (females) and the USA (males) the highest. In middle-aged adults with normal BMI, the use of lipid-lowering medicines increased in fewer countries, with the largest increase in South Korea.

**Figure 5:**
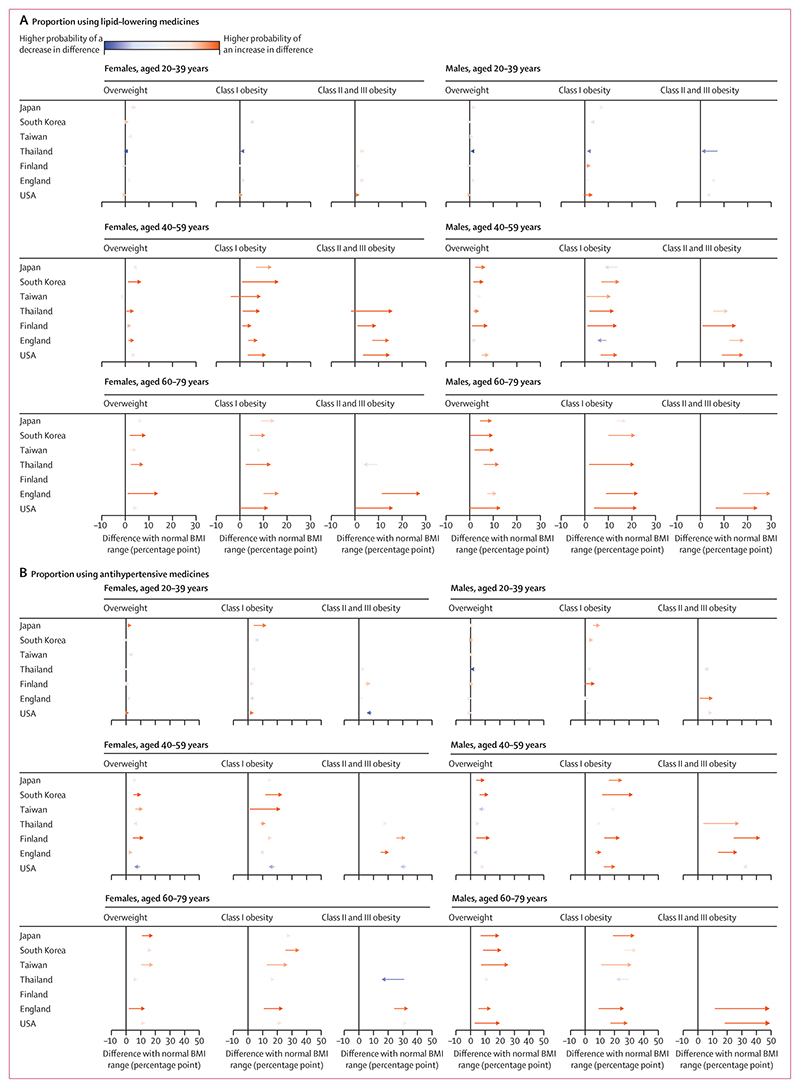
Change in difference in use of lipid-lowering and antihypertensive medicines between obesity or overweight and normal BMI Arrows show how the difference in treatment between obesity or overweight and normal BMI changed from earliest to latest years of data. Arrows pointing towards the vertical line at 0 indicate convergence, arrows pointing away from the vertical line indicate divergence. Regardless of the arrow’s direction, a positive number means the proportion of those treated is higher in those with obesity and overweight than in those with a normal BMI, and vice versa. Orange arrows indicate a rightward (positive) change towards or above the level of normal BMI, and blue arrows indicate a leftward (negative) change towards or below the level of normal BMI. If an increase in difference is indistinguishable from a decrease in difference, *P* is 0·50, and is shown in white. Probabilities close to 0·50 indicate less certainty of an increase or decrease in difference. Probabilities closer to 1 indicate more certainty that the estimated decrease or increase is a true change in that direction. For an estimated decrease, these high probabilities are shown in darker blue; for an estimated increase, they are shown in darker orange. Analysis period from earliest to latest year of data for lipid-lowering medicines was Japan (2003–22), South Korea (2005–24), Taiwan (1995–2019), Thailand (2000–20), Finland (1992–2023), England (1998–2021), and the USA (1991–2022); and for antihypertensive medicines was Japan (1990–2022), South Korea (1998–2024), Taiwan (1995–2019), Thailand (1995–2020), Finland (1992–2023), England (1992–2021), and the USA (1991–2022). See the appendix (pp 104–106) for figure without exclusions based on the number of participants. Age range for data from Finland is 30–59 years, grouped into 30–39 years and 40–59 years.

Among middle-aged and older adults, not only was the use of lipid-lowering medicines more common for those with obesity and overweight than those with a normal BMI throughout the analysis period (figure 4), but also the gap increased over time (ie, a divergence; figure 5; appendix pp 41–43). Among young adults, the proportion of treated participants remained close to 0% in those with overweight and no more than 15% in those with obesity throughout the analysis period (figure 4). The pooled estimate for the differential increase in lipid-lowering medicines compared with normal BMI across all countries, age groups, and obesity and overweight BMI ranges was 1·5 percentage points per decade (95% CI 1·0–2·1) for females and 1·6 percentage points per decade (1·0–2·2) for males (figure 6). The extent of divergence increased with age. The pooled results were 3·1 (2·1–4·2) percentage points per decade for older females and 4·2 (3·0–5·4) percentage points per decade for older males. The largest rises in difference were for class II and III obesity in older females (5·3 percentage points per decade [2·3–8·3]) and for class I obesity in older males (6·0 percentage points per decade [3·3–8·7]). In older adults, divergence was largest for males in Thailand and females and males in England and the USA with obesity, whose advantage in medicine use grew by as much as 19 percentage points. At the latest year of data, 70–72% of older males in England and the USA with class II and III obesity used lipid-lowering medicines, compared with 40–48% among those with normal BMI (figure 5; appendix pp 41–43, 47–49).

The proportion of older adults with normal BMI who used antihypertensive medicines increased in Asian countries by up to 26 percentage points (*P*>0·999, 95% CI 20–31 percentage points) for females in Thailand and 25 percentage points (*P*>0·999, 21–29 percentage points) for males in South Korea during the analysis period (figure 4; appendix pp 44–46). The increase in the use of antihypertensive medicines in those with a normal BMI was not detectable in the USA, at *P* of 0·975, in most age groups, possibly because some of these increases had happened before our analysis period.^9^ There was no or little change in the use of antihypertensive medicines in people with a normal BMI in middle-aged adults.

The use of antihypertensive medicines was more common in those with obesity and overweight than those with a normal BMI (figure 4). Further, for middle-aged and older adults, the use of antihypertensive medicines increased more in those with obesity and overweight than in those with a normal BMI in most countries (figure 5; appendix pp 41–43). The use of antihypertensive medicines in young adults was close to 0% in those with overweight and up to 25% in those with obesity throughout the analysis period (figure 4). The pooled estimate for the differential increase in use of antihypertensive medicines compared with individuals with a normal BMI across all countries, age groups, and obesity and overweight BMI ranges was 0·7 percentage points per decade (95% CI 0·3–1·0) for females and 2·0 percentage points per decade (1·3–2·8) for males (figure 6). The pooled results in older adults were 2·1 (1·2–3·0) percentage points per decade for females and 5·2 (3·3–7·1) percentage points per decade for males. At the extreme, in older males in the USA and England, the treatment gap between class II and III obesity and normal BMI became approximately 50 percentage points larger, with antihypertensive treatment rate reaching 75–80% in class II and III obesity compared with 25–31% in those with a normal BMI (figure 5, appendix pp 41–43, 47–49).

Sensitivity analyses showed that the findings were robust to using a linear trend model and to the exclusion of any of the 24 sex–age–BMI-specific time series in a country if fewer than three units in the time series had 25 or more participants (appendix pp 6–7, 82–109).

## Discussion

Our multi-country analysis has revealed shifts in the adverse cardiometabolic traits associated with obesity and the age groups affected by them. We found that differences in non-HDL cholesterol and SBP between those with obesity and those with a normal BMI narrowed or disappeared, especially in older adults, in some cases making those with and without obesity indistinguishable in terms of these cardiometabolic traits. These trends accompanied a larger increase in the use of lipid-lowering and antihypertensive medicines in middle-aged and older adults with obesity, especially class II and III obesity, than in those with a normal BMI. There was heterogeneity across countries in the extent of convergence. Countries in which convergence was greatest came from both Asia (eg, South Korea) and Europe or North America (eg, England and the USA). HDL cholesterol, which is not affected by these medicines, diverged in those with obesity (ie, moved further below the levels in the normal BMI range). For young adults, who are less commonly treated for high cholesterol or blood pressure regardless of BMI, there has been little change in the gap in treatment or cardiometabolic traits between obesity and normal BMI. As a result, young adults with obesity still have less healthy lipid profiles and higher blood pressure than those with a normal BMI.

The strengths of our study include its scope of characterising important cardiometabolic traits associated with obesity in multiple countries using high-quality national data. Our data covered multiple decades during which effective medicines and lifestyle interventions have become available and incorporated in clinical guidelines.

Like all longitudinal multi-country studies, our study is also affected by some limitations. We started our analysis from the 1990s because few countries had data before then. This time period coincides with when lipid-lowering medicines were scaled up in most countries;^26,27^ for antihypertensive medicines and blood pressure, an additional early decade of data would be ideal.^9^ Studies from different countries spanned different years; nonetheless, all countries had at least one study in the 1990s and one after 2019. Sample sizes were small in some study–sex–age–BMI units, particularly those with obesity in Asian countries. To robustly estimate trends, we used time series when more than three time points had sufficient sample sizes. Sensitivity analysis (appendix pp 6–7) showed that our overall conclusions were not affected by this strict criterion for inclusion. In Finland, data did not cover the older ages, hence results are unavailable for this age group. How lipids and blood pressure are measured has changed over time. For lipids, 93 (96%) of 97 studies had standardised their lipid measurement, which minimises the effects of method changes (appendix pp 9–11). For blood pressure, 71 (65%) of 110 studies used a standard mercury device and another 35% used a digital oscillometric device. Although the change in measurement method may have a small effect on trend in each BMI range,^28,29^ it would not affect the difference between obesity or overweight and normal BMI because the same measurement method was used in each study irrespective of participants’ BMI. Health surveys are affected by non-response, and in some countries the extent of non-response has increased over time.^30,31^ The response rates in the surveys used in our analysis were mostly 60–80%, with higher rates in Thailand and lower rates in post-COVID-19-pandemic surveys in England and the USA. Although post-stratification sampling weights adjust for non-response as related to the measured characteristics of participants, a residual effect might remain based on unmeasured characteristics. Some surveys did not collect information on medicines and were not included in the analyses of treatment; nonetheless, data on use of medicines were available for 81 (84%) of 97 studies for cholesterol and 106 (96%) of 110 studies for blood pressure. Further, it would have been ideal to have data on type and intensity of treatment to evaluate if they are different across different BMI ranges and if they have changed over time. We did not analyse trends in different HDL cholesterol particles and emerging lipid markers such as apolipoprotein B and apolipoprotein A-I because such information is not available in most population-based surveys, nor are these commonly used in clinical settings yet.^32,33^ Future research should also address variations in metabolic traits and treatment in obesity and normal BMI in relation to characteristics such as education or urban versus rural residence to target prevention and treatment to specific population subgroups. Finally, it would be ideal to evaluate convergence in a larger number of countries, including low-income and middle-income countries where the use of antihypertensive and lipid-lowering medicines might be lower.

Precise attribution of the observed trends and their variations across BMI ranges, age groups, and countries requires granular data on diet and health behaviours, with repeated measurements over time, and separately by age and BMI; these data are currently unavailable. In the absence of such data, the explanations for these changes must draw on the broader evidence on these determinants.

The declines in non-HDL cholesterol and SBP across BMI ranges and their variations across countries are at least partly due to changes in diet, smoking, and alcohol use. In Europe and North America, the replacement of saturated with unsaturated fats and reductions in trans-fats intake would have lowered non-HDL cholesterol.^13,26,34–39^ In contrast, the steadiness or more moderate decline in non-HDL cholesterol in Asia, especially in younger age groups, could be due to the combination of increased consumption of animal-based fats and higher intake of fibre-rich vegetables.^34–37,40^ A decrease in smoking and carbohydrate intake and an increase in non-trans-fat intake and alcohol use might have contributed to the increase in HDL cholesterol in Japan, South Korea, and Thailand.^34–37,40–46^ Similarly, lower smoking rates and trans-fats intake might have contributed to the rise in HDL cholesterol in Europe and North America,^26,37,38,43^ whereas higher consumption of fruits and vegetables^34–36,47^ and lower salt consumption, alcohol use, and smoking^38,43,46,48–50^ might have contributed to lower blood pressure. These differences would themselves arise from variations in the cost and availability of various foods, alcohol, and tobacco and the fiscal and regulatory policies that affect them.^51–54^

Greater use of lipid-lowering and antihypertensive medicines are also likely to have contributed to the decline in non-HDL cholesterol and blood pressure in middle-aged and older adults across the normal-to-obesity BMI spectrum.^39,55–58^ We found that the use of lipid-lowering and antihypertensive medicines increased more in middle-aged and older adults with obesity compared with those with a normal BMI, which might be because those with obesity are screened more or more likely to be prescribed medicine. This differential increase in the use of medicines would explain part of the convergence of non-HDL cholesterol and blood pressure to individuals with a normal BMI. For example, clinical trials showed a reduction of 6–22 mmHg in SBP using antihypertensive medicines at varying intensities.^59^ A reduction of such magnitude, when combined with a 30-percentage-point differential increase in antihypertensive use between obesity and normal BMI, would account for 1·8–6·6 mmHg of the observed 18 mmHg convergence in SBP between BMI groups. Similarly, statins reduce non-HDL cholesterol by 1·09–2·27 mmol/L.^60^ A 19-percentage-point larger increase in the use of statins in those with obesity compared with those with a normal BMI would account for 0·20–0·43 mmol/L of the 1·09 mmol/L convergence observed in our results. These numbers are smaller than the total magnitudes of convergence observed in mean SBP or non-HDL cholesterol across BMI ranges, which might be partly due to differences in treatment intensity. Differential intensity would occur because clinical guidelines do not suggest different targets for cholesterol and SBP control in relation to BMI, and physicians might adjust treatment intensity so that patients reach similar risk factor levels regardless of BMI. Intensive lifestyle modification in individuals with higher BMI might also account for some of the convergence in the outcomes. We also found heterogeneity in the extent of medicine use and how much it changed, which could be because clinical guidelines and health system policies and programmes that influence how much these medicines are prescribed and used differ across countries.^9,61–66^ The negligible effect of lipid-lowering medicines on HDL cholesterol and shared determinants of low HDL cholesterol and obesity (eg, low physical activity and consumption of low-fibre processed carbohydrates) could explain divergence, mostly in the form of smaller increase in HDL cholesterol in people with obesity compared with those with a normal BMI.

The persistence of blood pressure and cholesterol differences between young adults with obesity and those with a normal BMI might be because treatment rates remained low in these ages, regardless of BMI. The low treatment rates in younger adults might be because treatment decisions at least partly rely on calculated absolute risk,^9^ which increases with age. Additionally, health-care use or medication adherence might be lower in young adults, either because of their actual or perceived need for care or because they are less able to do so as they transition into work.^67–71^ Another reason for larger convergence in older ages compared with younger ages could be that people with obesity who have low blood pressure and optimal lipid profiles increasingly survive to older ages, lowering mean blood pressure and cholesterol in this group.

Our findings on the substantial decline in blood pressure and cholesterol, which are important mediators of the cardiovascular risks associated with obesity,^7^ in those with obesity mean that cardiovascular effects of obesity might have been dampened through addressing these mediators, although effects through other pathways, including inflammation, hyperglycaemia, and insulin resistance might remain.^4^ This finding, that convergence of blood pressure and cholesterol between obesity and normal BMI in older age groups might have partly reduced the difference in cardiovascular risk, is consistent with the convergence of cardiovascular mortality between the two groups.^72^ Definitive establishment of this effect would require repeated population-based cohorts with measurements of BMI, blood pressure, cholesterol, and other mediators at baseline and linkage to hospital and death records. If this trend continues, the risks associated with obesity and the benefits of its prevention and treatment in older ages might come from a combination of vascular and non-vascular conditions that are also affected by obesity,^72^ including diabetes, cancers, renal and liver diseases, and musculoskeletal conditions. This shift might in turn indicate a need to broaden the advice and treatment for patients with obesity based on multiple morbidities. Finally, our results show that, although older adults with obesity have probably benefited from medical treatment in terms of their cardiovascular risk factors, young adults with obesity remain at elevated cardiometabolic risk. Public health and health system programmes should use early lifestyle interventions, screening and, when appropriate, pharmacological treatment in this younger group to prevent long-term cardiovascular and other complications. As weight reduction medicines become increasingly used in high-income countries, our results provide a detailed picture of important cardiometabolic traits in people who might use such medicines and allow tracking how these traits change as weight reduction medicines supplement medicines for hypertension and lipids. In low-income and middle-income countries, where treatment use is lower,^61^ obesity might be an even larger threat for the entire range of diseases affected by it. With the current high cost of weight reduction medicines, improved detection and treatment of high blood pressure and cholesterol could help address some of its cardiovascular harms.

## Supplementary Material

Supplementery Appendix

## Figures and Tables

**A:**
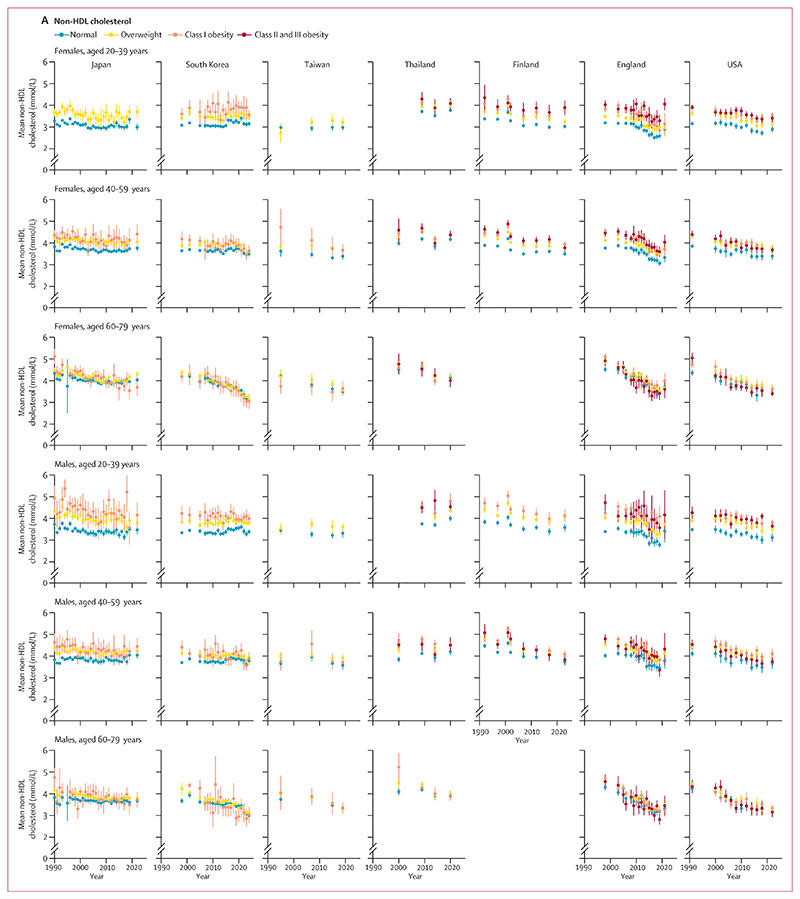


**B:**
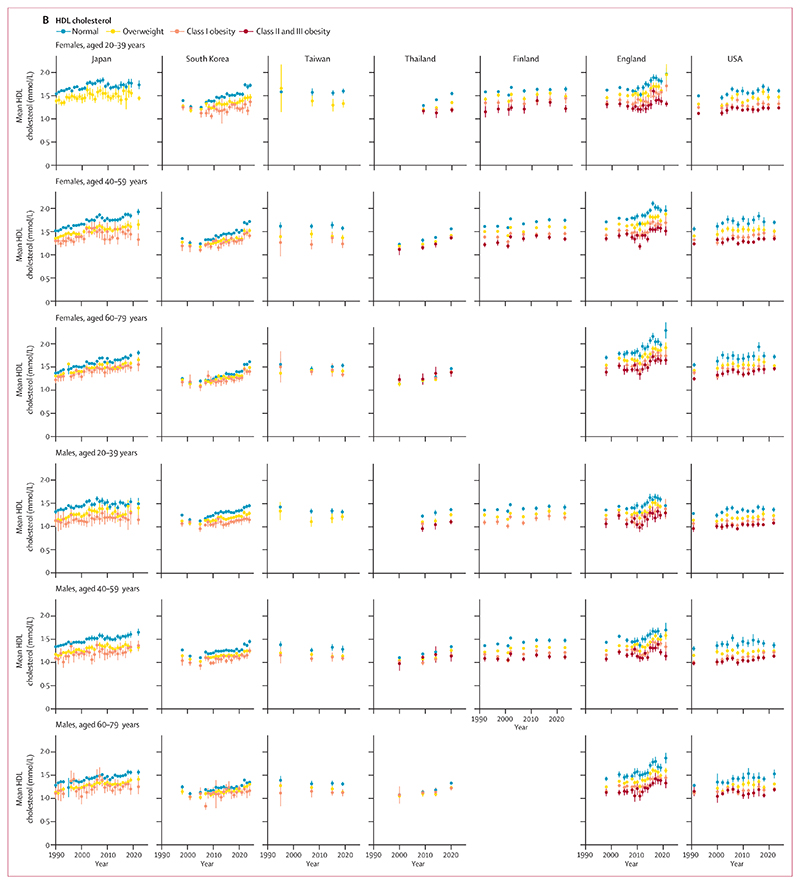


**C:**
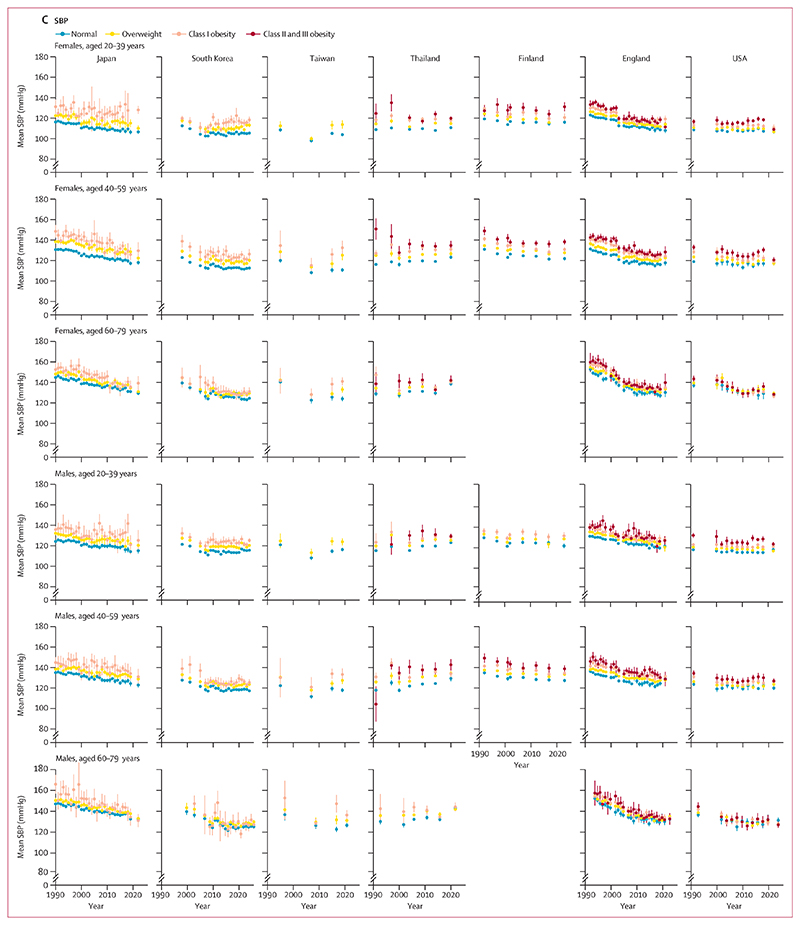


**A:**
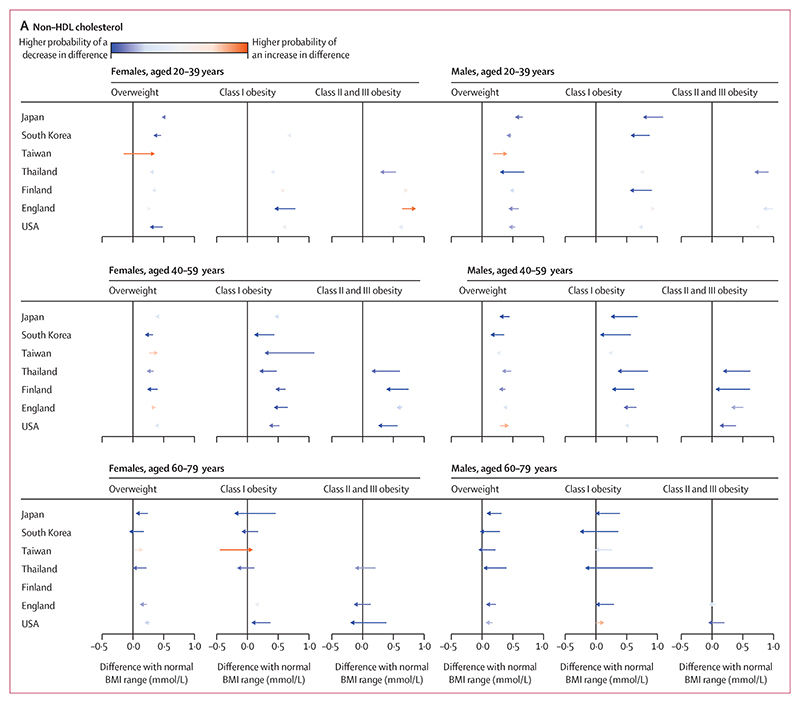


**B:**
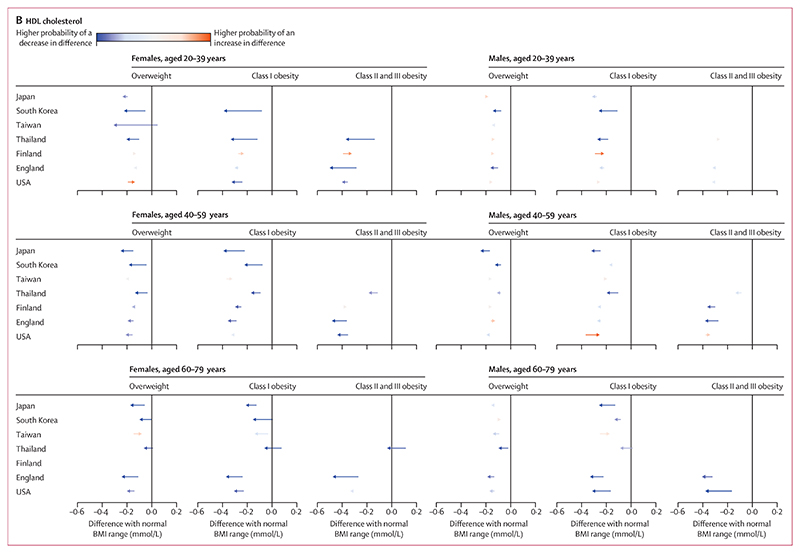


**C:**
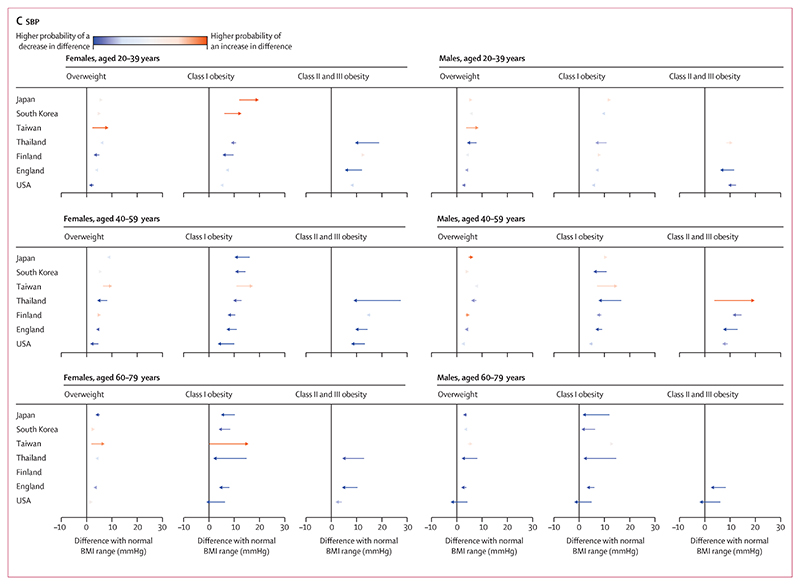


**A:**
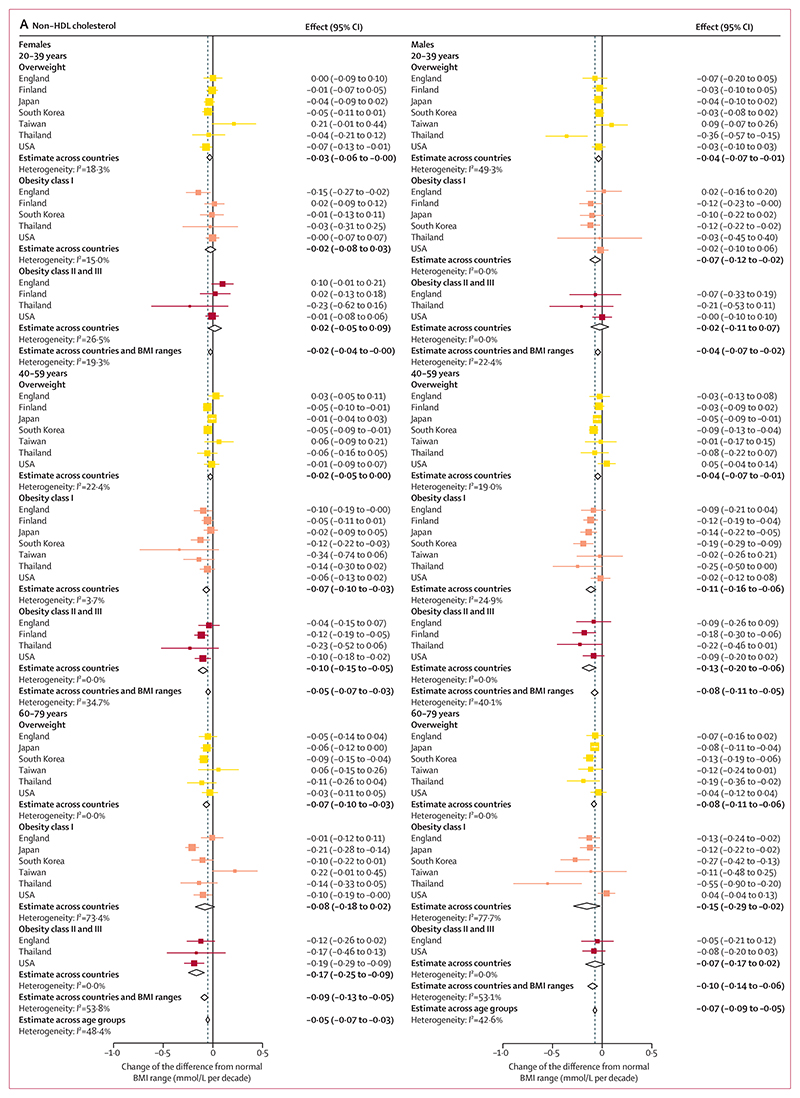


**B:**
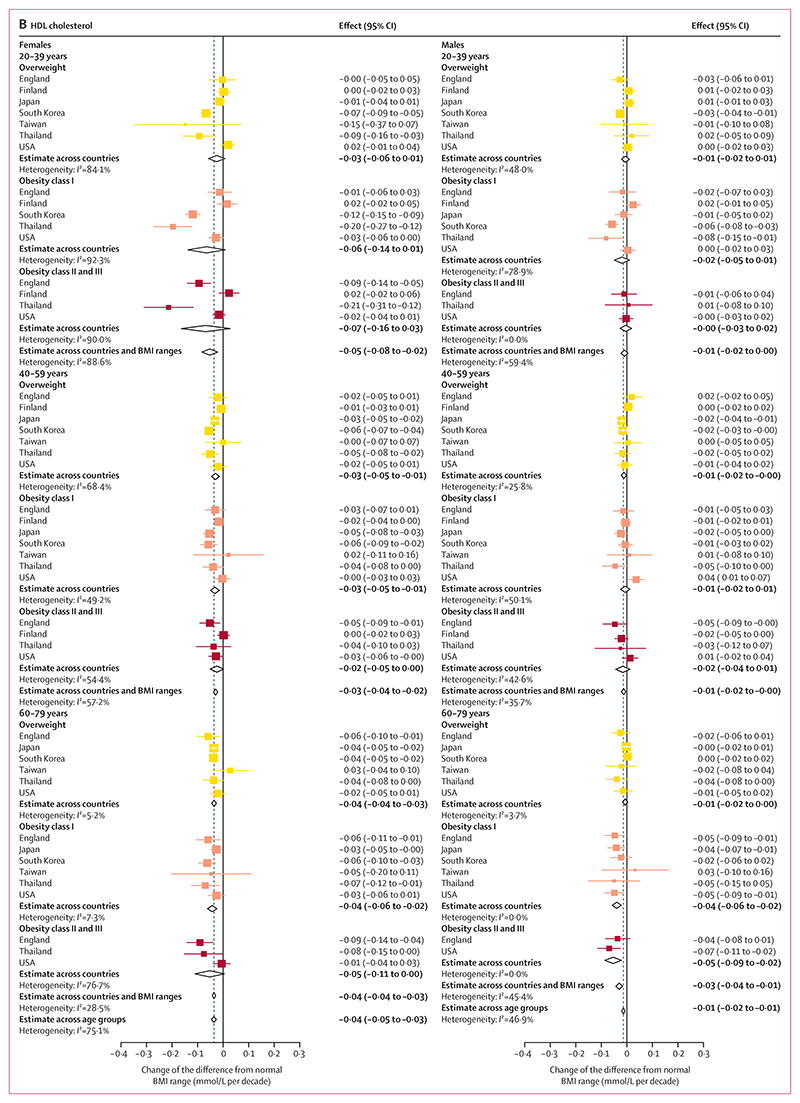


**C:**
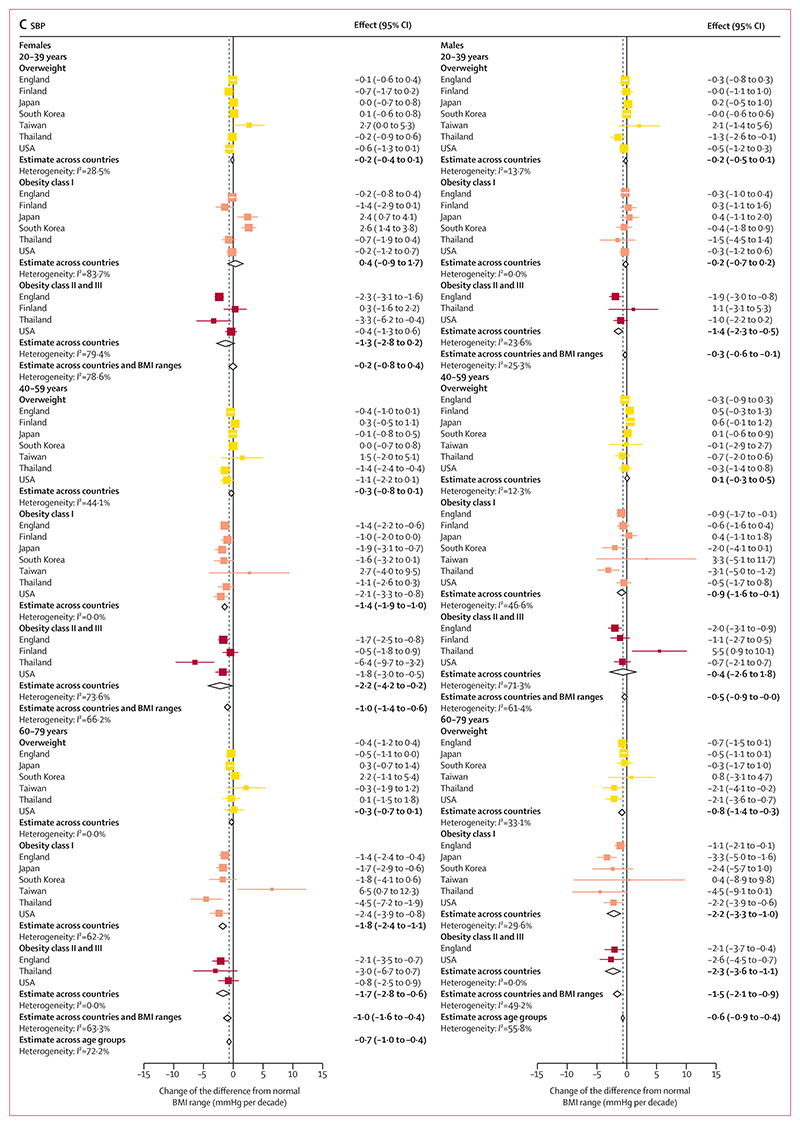


**A:**
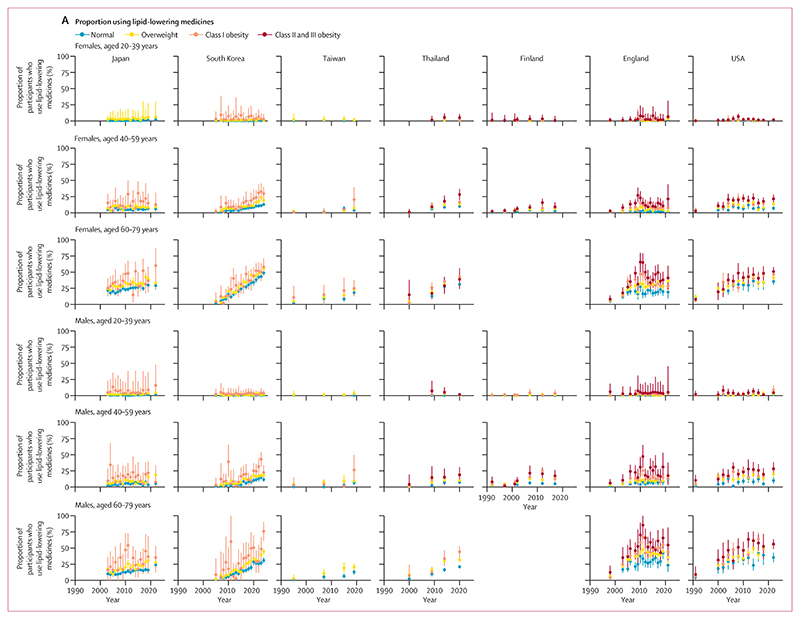


**B:**
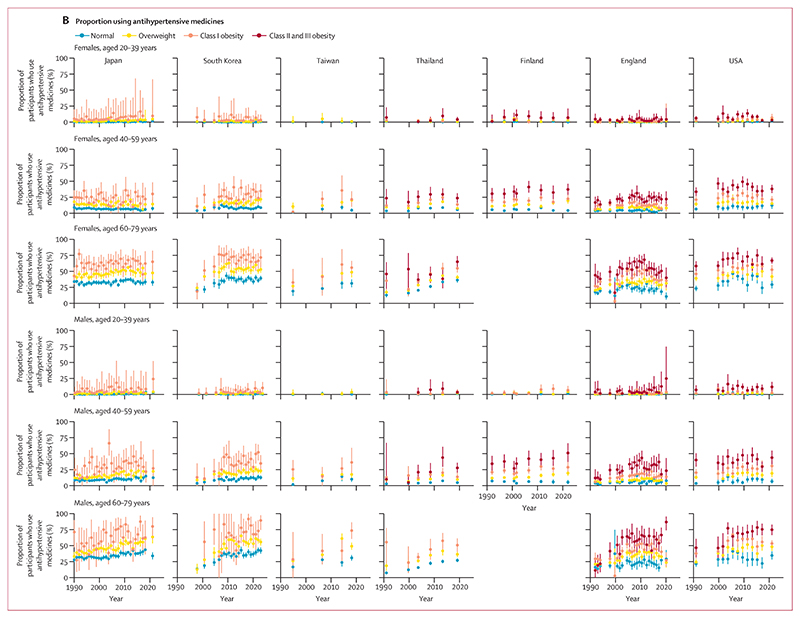


**A:**
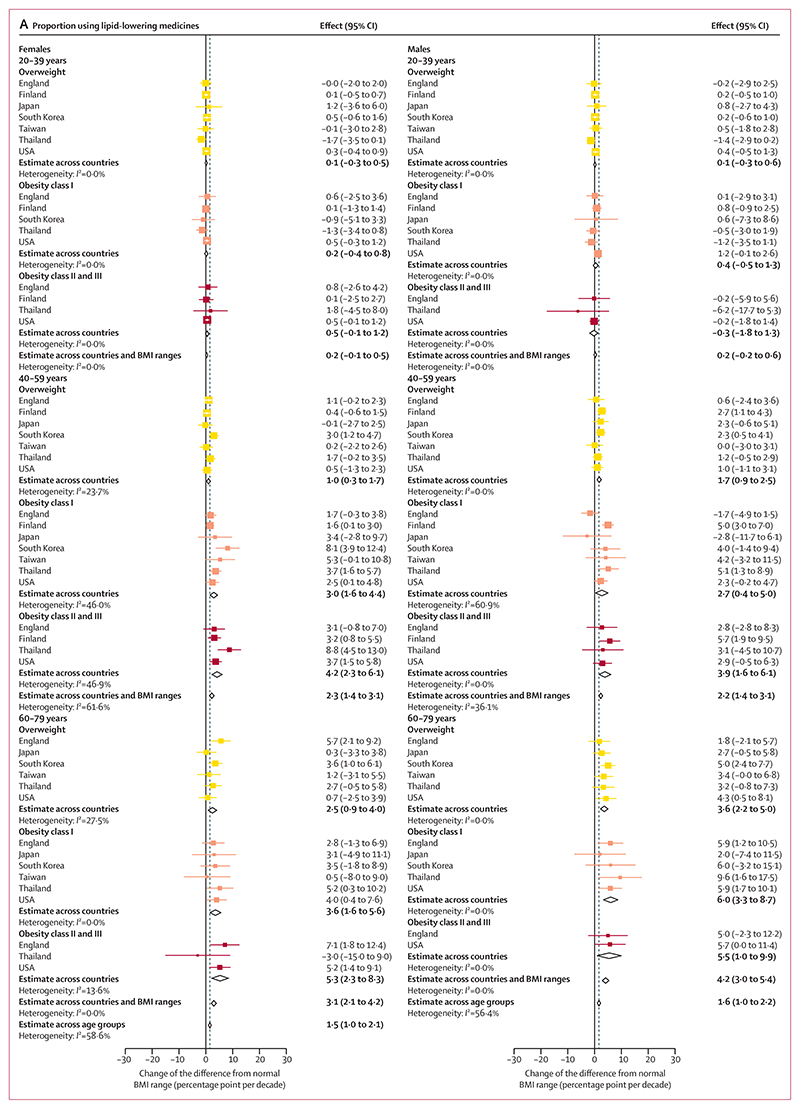


**B:**
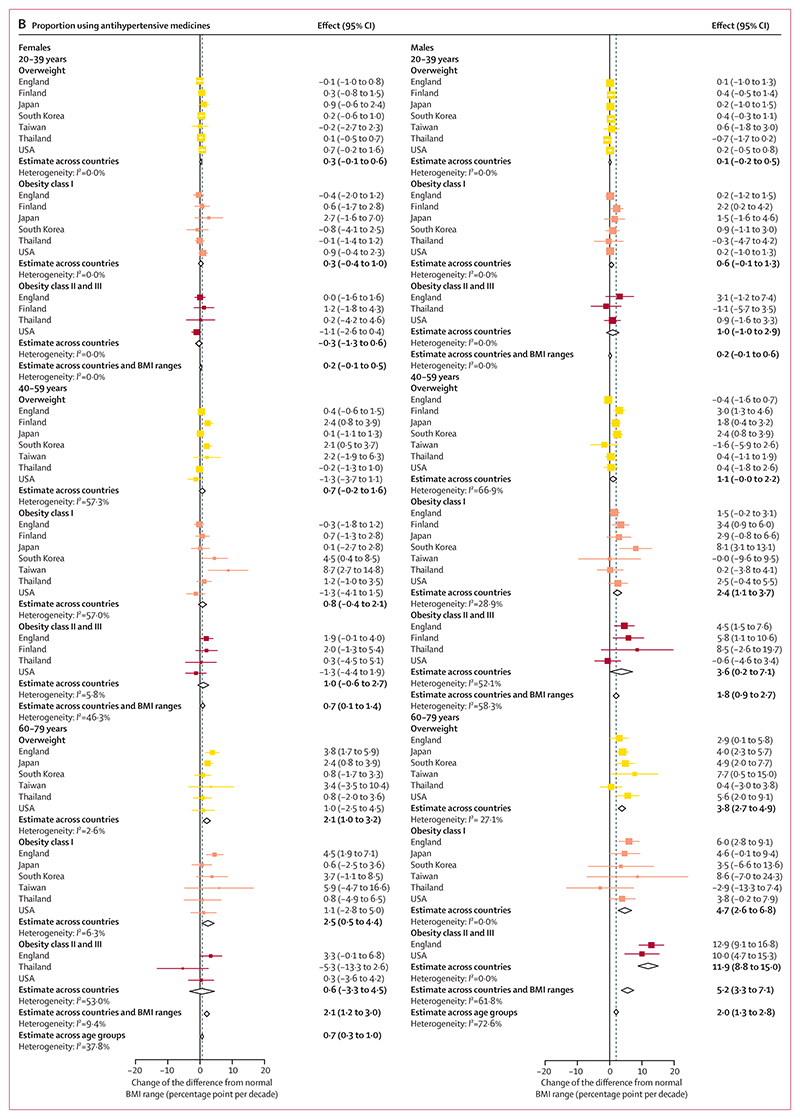


## Data Availability

Data used in this research are governed by data-sharing protocols of participating studies. Information on data access, including data download sites and contact information for data custodians, are provided at https://www.ncdrisc.org and Zenodo (https://doi.org/10.5281/zenodo.19856243). The computer code is also available from the same sites.

## Research in context

### Evidence before this study

We searched PubMed for articles published from database inception to Nov 10, 2025, with no language restrictions, using the following search terms: (“Body Mass Index”[mh] OR Obesity[mh:noexp] OR Overweight[mh:noexp] OR overnutrition[mh:noexp]) AND (“Blood Pressure”[mh] OR “Lipoproteins, LDL”[mh] OR “Lipoproteins, HDL”[mh] OR “Hypertension”[mh] OR “Dyslipidemias”[mh:noexp] OR “Hyperlipidemias”[mh] OR (cardiovascular[tiab] AND risk factor*[tiab])) AND (“body mass index group*”[tiab] OR “BMI group*”[tiab] OR “body mass index categor*”[tiab] OR “BMI categor*”[tiab] OR “body mass index range*”[tiab] OR “BMI range*”[tiab]) AND trend*[tiab] AND “Adult”[mh] AND “Humans”[mh] NOT (Clinical Trial[ptyp] OR Randomized Controlled Trial[ptyp] OR Comment[ptyp] OR Case Reports[ptyp]).

Many studies explored the association between BMI and blood pressure, lipids, or cardiovascular disease in specific cohorts, but these studies did not report time trends of these conditions and risk factors at different BMI ranges. These studies generally found that higher BMI is associated with higher blood pressure and LDL or non-HDL cholesterol, or with higher prevalence of hypertension and dyslipidaemia. Eight studies reported time trends in hypertension, mean blood pressure, dyslipidaemia, or cholesterol stratified by BMI. These studies were all conducted in single countries (the USA, England, Peru, Brazil, and Sweden), some using data from national populations and others from specific communities or subgroups of the population. Five studies found that hypertension prevalence or mean blood pressure decreased more in individuals in higher BMI ranges than those with a normal BMI. Four studies found that there was a greater decrease of dyslipidaemia prevalence or serum cholesterol in people in higher BMI ranges than those with a normal BMI.

### Added value of this study

To our knowledge, this is the first study that uses repeated population-based data from national populations of multiple countries to characterise blood pressure, cholesterol, and their respective treatments in people with obesity or overweight compared with those with a normal BMI. We used data from seven industrialised countries in Asia, Europe, and North America. We also evaluated changes in the difference in blood pressure and cholesterol between obesity or overweight and normal BMI and whether the differences have shrunk over decades when prevention and treatment of hypertension and hypercholesterolaemia have been increasingly incorporated into public health programmes and clinical guidelines.

### Implications of all the available evidence

In industrialised countries, there have been major shifts in the adverse cardiometabolic traits associated with obesity and the age groups affected by them since the last decade of the 20th century. As a result, systolic blood pressure and non-HDL cholesterol of older people with obesity are similar to, and in some cases even lower than, those of people with a normal BMI. This finding might be partly because older people with obesity use more antihypertensive and lipid-lowering medicines than those with a normal BMI. Young adults with obesity remain metabolically at higher risk than their counterparts with a normal BMI and should be a focus of prevention and, when relevant, treatment.
