# Supplementary material for: Metabolic traits in obesity and normal BMI in industrialised countries: a multi-country analysis of national population-based studies
Source: Lancet. Author manuscript; Available in PMC 2026 Aug 8. (PMC7619335; doi:10.1016/S0140-6736(26)00758-0)
Supplement: Supplementary file 1 [file EMS216203-supplement-Supplementery_Appendix.pdf]

THE LANCET

Supplementary appendix

This appendix formed part of the original submission and has been peer reviewed. We post it as supplied by the authors.

Supplement to: NCD Risk Factor Collaboration (NCD-RisC). Metabolic traits in obesity and normal BMI in industrialised countries: a multi-country analysis of national population-based studies. *Lancet* 2026; published online July 1. https://doi.org/10.1016/S0140-6736(26)00758-0.

Table of Contents

Appendix Text 1. Outcomes and age groups..1

Appendix Text 2. Data sources, cleaning and management..3

Appendix Text 3. Sensitivity analyses..6

Appendix Text 4. Comparison with other studies...8

Appendix Table 1. Data sources used in this analysis...9

Appendix Table 2. Data used for analysis in each country..12

Appendix Table 3. Regression coefficients and associated 95% confidence interval and P for linear models for cholesterol and blood pressure over time in normal BMI..........................13

Appendix Table 4. Regression coefficients and associated 95% confidence interval and P for linear models for treatment over time in normal BMI..15

Appendix Table 5. Regression coefficients and associated 95% confidence intervals and P for linear models for the difference in cholesterol and blood pressure between obesity and overweight and normal BMI..18

Appendix Table 6. Regression coefficients and associated 95% confidence intervals and P for linear models for the difference in treatment between obesity and overweight and normal BMI...21

Appendix Figure 1. Flowchart of data cleaning...25

Appendix Figure 2. Trends in prevalence of obesity and overweight...................................27

Appendix Figure 3. Trends in difference in age-standardised mean non-HDL cholesterol, mean HDL cholesterol and mean SBP between obesity and overweight and normal BMI....29

Appendix Figure 4. Cholesterol and blood pressure in normal BMI.....................................33

Appendix Figure 5. Difference in cholesterol and blood pressure between obesity and overweight and normal BMI..37

Appendix Figure 6. Trends in difference in age-standardised proportion of participants using lipid-lowering or antihypertensive medicines between obesity and overweight and normal BMI...41

Appendix Figure 7. Use of lipid-lowering and antihypertensive medicines in normal BMI.....44

Appendix Figure 8. Difference in use of lipid-lowering or antihypertensive medicines between obesity and overweight and normal BMI...47

Appendix Figure 9. Trends in age-standardised mean non-HDL cholesterol, HDL cholesterol and SBP in normal BMI with fitted linear and LOESS regressions.......................................50

Appendix Figure 10. Trends in difference in age-standardised mean non-HDL cholesterol, mean HDL cholesterol and mean SBP between obesity and overweight and normal BMI with fitted linear and LOESS regressions...58

Appendix Figure 11. Trends in age-standardised proportion of participants using lipid-lowering or antihypertensive medicines in normal BMI with fitted linear and LOESS regressions..66

Appendix Figure 12. Trends in difference in age-standardised proportion of participants using lipid-lowering or antihypertensive medicines between obesity and overweight and normal BMI with fitted linear and LOESS regressions...74

Appendix Figure 13. Trends in age-standardised mean non-HDL cholesterol, HDL cholesterol and SBP by BMI range without exclusions based on number of participants......82

Appendix Figure 14. Trends in difference in age-standardised proportion of participants using lipid-lowering or antihypertensive medicines between obesity and overweight and normal BMI without exclusions based on number of participants..86

Appendix Figure 15. Change in difference in cholesterol and blood pressure between obesity or overweight and normal BMI without exclusions based on number of participants............90

Appendix Figure 16. Pooled analysis of change in difference in cholesterol and blood pressure between obesity or overweight and normal BMI without exclusions based on number of participants..94

Appendix Figure 17. Trends in age-standardised proportion of participants using lipid-lowering or antihypertensive medicines, by BMI range without exclusions based on number of participants...98

Appendix Figure 18. Trends in difference in age-standardised proportion of participants using lipid-lowering or antihypertensive medicines between obesity and overweight and normal BMI without exclusions based on number of participants......................................101

Appendix Figure 19. Change in difference in use of lipid-lowering and antihypertensive medicines between obesity and overweight and normal BMI without exclusions based on number of participants...104

Appendix Figure 20. Pooled analysis of change in difference in antihypertensive and lipid-lowering medicines between obesity or overweight and normal BMI without exclusions based on number of participants...107

Appendix Figure 21. Relationship between BMI and non-HDL cholesterol per decade......110

Appendix Figure 22. Relationship between BMI and systolic blood pressure per decade...113

References...116

Appendix Text 1. Outcomes and age groups.

Our outcomes were mean systolic blood pressure (SBP) and non-HDL and HDL cholesterol, and the proportion of the sample who use antihypertensive and lipid-lowering medicines. We used non-HDL cholesterol rather than low-density lipoprotein (LDL) cholesterol because most studies in our analysis had measured total cholesterol (TC) and HDL cholesterol, from which non-HDL cholesterol can be calculated by subtraction. By contrast, LDL cholesterol was directly measured in only 25 (26%) of the 97 studies that measured lipids. When LDL cholesterol is not directly measured, it can be calculated using data on triglycerides which were available in 55 (76%) of the remaining 72 studies. Non-HDL and LDL cholesterol are also highly correlated (Pearson correlation coefficient = 0.94 in studies with data on both variables).[1] Furthermore, non-HDL cholesterol has been shown to predict the risk of ischemic heart disease at least as well as LDL cholesterol.[2,3]

We report these outcomes in participants aged 20-79 years in the normal BMI range (20.0 to <25.0 kg/m^2), and the difference between participants in the obesity or overweight BMI ranges and those in the normal BMI range. Class I obesity was defined as having a BMI of 30.0 to <35.0 kg/m^2; class II and III obesity as BMI ≥35.0 kg/m^2; and overweight as BMI of 25.0 to <30.0 kg/m^2. We used a cut-off of 30 kg/m^2 for obesity in all countries because the latest WHO consultation did not recommend regional-specific cut-offs.[4] Further, a global analysis has shown that BMI is strongly correlated with abdominal adiposity in all regions, and that abdominal adiposity at any BMI level is similar between the populations of Europe and north America and those of countries in east Asia which are analysed here, with excess abdominal adiposity being largely limited to south Asian populations.[5] We analysed the two obesity BMI ranges separately because those in the class II and III obesity BMI range are at a higher risk than class I obesity and may be more likely to be triaged for treatment. We report these outcomes for females and males in the following age groups: 20-39-year-olds, 40-59-year-olds and 60-79-year-olds. We did not use data for those younger than 20 years because the definitions of overweight and obesity are different for children and adolescents compared to

adults.[6] We did not use data for those 80 years and older because fewer studies had data in these ages and because treatment decisions may be different in these ages. We did not use data on race and ethnicity, because the scope of our analysis was national populations of multiple countries, and because race and ethnicity are not defined or measured consistently across countries.

Appendix Text 2. Data sources, cleaning and management.

We used studies from 1990 to 2024 with measurement of height, weight and at least one of blood pressure and cholesterol in representative samples of the national populations of the seven countries chosen for analysis. The studies were collated by the NCD Risk Factor Collaboration (NCD-RisC) as detailed previously.[7-10] In summary, the studies were either publicly available health surveys or those accessed through requests to national health and statistical agencies. The inclusion criteria were: (1) data were collected using probabilistic sampling with a defined sampling frame; and (2) height, weight and at least one of blood pressure and cholesterol were measured with a validated protocol. Studies were excluded if they (1) included or excluded participants based on health status or cardiovascular risk; (2) were conducted only among ethnic minorities or specific educational, occupational, or other socioeconomic subgroups; and (3) were solely based on self-reported height, weight, blood pressure or cholesterol without any measurement because such data are subject to bias. For this analysis, we also excluded studies that (1) had an age range smaller than 40 years of the target range of 20-79 years; (2) were not nationally representative; or (3) were follow-up rounds of studies that had repeated measurements of the same cohorts over time, to avoid overrepresenting the same participants and lack of statistical independence in the estimates. All data were checked by at least two persons independently to ensure that they met the inclusion criteria and that variable labels and units were appropriately recorded and used.

We used 110 studies that were done from 1990 to 2024 in these seven countries. All studies had data on blood pressure and 97 had data on cholesterol. Of 110 studies with blood pressure data, 106 had data on the use of antihypertensive medicines. Of 97 studies with cholesterol data, 81 had data on the use of lipid-lowering medicines. Details of the studies included in the analysis are shown in Appendix Table 1. These studies together had 1,386,728 participants, of whom 978,425 participants were aged 20-79 years.

Data cleaning and management

Data cleaning steps are shown in Appendix Figure 1. We excluded 469 participants with missing age and sex (<0.1% of all participants) and restricted data to participants aged 20-79 years, which led to data on 978,425 participants. We excluded 6,854 participants who were pregnant at the time of measurement (0.7% of participants aged 20-79 years) as body weight, blood pressure and lipids change during pregnancy.[11,12]

After these exclusions, we cleaned the data based on anthropometric variables (height, weight and BMI). As in previous uses of these data,[8] we excluded 168,992 participants (17.3% of participants aged 20-79 years) with missing anthropometric measurements; most of these were from the Health Survey for England and deemed as unreliable by their validation protocol. We also excluded 35 participants (<0.01% of participants aged 20-79 years) with weight <12 kg or >300 kg, height <100 cm or >250 cm, or BMI <10 kg/m^2 or >80 kg/m^2, because such values are likely to be due to measurement or data recording errors. Further, we did not use data for those with BMI <20 kg/m^2 because they were not a part of our study design.

We then cleaned the data for blood pressure and cholesterol separately to keep participants with valid blood pressure data in the blood pressure analysis even if they did not have data on cholesterol or were excluded based on their cholesterol measurements, and vice versa. For the blood pressure analysis, we excluded 95,352 participants (13.5% of participants with BMI ≥20 kg/m^2) with missing blood pressure data. In studies with data on blood pressure, the median difference across all study-sex groups in BMI of people with and without blood pressure data was -0.15 kg/m^2. We excluded 42 participants (<0.1%) with SBP <70 mmHg or >270 mmHg, DBP <30 mmHg or >150 mmHg, or SBP ≤ DBP + 10 mmHg, because these values are likely to reflect measurement or data recording errors. For the cholesterol analysis, we removed 13 studies with 135,594 participants that had not measured TC or HDL cholesterol. In the remaining 97 studies, we excluded 129,824 participants (18.4%) with missing data for TC or HDL cholesterol. In studies with data on cholesterol, the median

difference across all sex-age groups in BMI of people with and without non-HDL data was -0.10 kg/m^2. We excluded 146 participants (<0.1%) with TC <1.75 mmol/L or >20 mmol/L, HDL cholesterol <0.4 mmol/L or >10 mmol/L, or TC < HDL cholesterol, because these values are likely to reflect measurement or data recording errors. We also excluded six participants with TC < LDL cholesterol or TC minus (LDL cholesterol + HDL cholesterol) < predefined margin of error (<0.1%) from studies that also had data on LDL cholesterol. The margin of error was determined using the Cholesterol Reference Method Laboratory Network's permitted measurement error limits (8.9% for TC, 12% for LDL cholesterol and 13% for HDL cholesterol)[13-15] and taking a worst-case scenario where TC is underestimated and LDL and HDL cholesterol are overestimated.

For Finland, five of the eight available studies did not measure those aged 20-24 and 75 to 79 years. One of these five studies did not measure those aged 65-74 years; in a further three, data for participants aged 65 years and above were only collected in some regions and therefore were not nationally representative. A further study in Finland only measured those aged 30 years and older. For this reason, we limited the analysis for Finland to ages 30-59 years, divided into 30-39 years and 40-59 years.

Appendix Text 3. Sensitivity analyses.

To assess sensitivity of our results to possible nonlinearity of trends, we repeated the analyses using locally estimated scatterplot smoothing (LOESS) regressions for all countries except Thailand and Taiwan which had less than five data points in all their sex-age time series. We fitted the LOESS models with smoothing windows (alpha) from 0.5 to 1.0 to determine the appropriate degree of smoothing. Those with alpha = 0.5 overfitted the data and those with alpha = 1.0 were similar to a linear fit. Therefore, we used an alpha of 0.75 for all LOESS models. We calculated the mean difference, mean absolute difference and correlation coefficients between the estimates at the earliest and latest years using linear versus LOESS models, by outcome and country-sex-age time series.

The mean difference between the estimates at the earliest and latest years using linear versus LOESS models was -1.46 mmHg for mean SBP, 0.0071 mmol/L for mean non-HDL cholesterol and -0.013 mmol/L for mean HDL cholesterol. The mean absolute difference was 1.76 mmHg for mean SBP, 0.094 mmol/L for mean non-HDL cholesterol and 0.052 mmol/L for mean HDL cholesterol. The correlation coefficient between estimates from the two approaches was 0.96 for mean SBP, 0.94 for mean non-HDL cholesterol and 0.95 for mean HDL cholesterol.

In an additional sensitivity analysis, we did not apply a cut-off for number of participants and included all country-sex-age group-BMI range times series, allowing the effect of number of participants to be via the uncertainty of the estimates. Removing the threshold of 25 participants for inclusion of a data point led to inclusion of a larger number of studies for the low-prevalence groups, especially obesity categories in countries in Asian countries, which were then also used in the analysis albeit with large uncertainty in their estimates due to the small number of participants (Appendix Figures 13-20).

The results were similar to the main analysis for mean non-HDL cholesterol, mean HDL cholesterol and mean SBP (Figure 3 and Appendix Figure 16). In the obesity categories in young adults, the pooled numerical values of the extent of convergence or divergence in relation to normal BMI changed after data were included for Japan, South Korea, Taiwan, Thailand and Finland but these were within the range of statistical uncertainty. The results were also similar to the main analysis for treatment with the exception of lipid-lowering medicines in older females and males with class I and II obesity. In this group, the inclusion of data from Japan, South Korea, Taiwan, Thailand and Finland led to the divergence in medication use seen in the main analysis no longer being distinguishable from no change in the difference. This happened because the estimates for these countries, although highly uncertain due to very small number of participants, still influenced the pooled result in a random-effect meta-analysis.

Appendix Text 4. Comparison with other studies.

To our knowledge, there are no studies that compared metabolic traits by obesity status in multiple national populations. Our findings are consistent with the few studies in individual countries that examined one or both of cholesterol and blood pressure by BMI ranges.[16-21] The BMI-specific trends, often in the form of declines, in cholesterol and blood pressure are also largely consistent with the national trends in these outcomes.[1,9,10,22-33] Similarly, the increase in the use of lipid-lowering and antihypertensive medicines is consistent with reports based on health survey or prescription data, as is the plateauing of antihypertensive medicines around 2010.[10,22,23,27-29,31,34-39] Finally, the convergence of blood pressure and cholesterol between obesity and normal BMI in older ages is consistent with the converging trend of cardiovascular mortality between the two groups.[40]

Appendix Table 1. Data sources used in this analysis.

	Country	Study years	Survey/Study name	Age range used in this analysis	Sample size used in blood pressure analysis*		Sample size used in cholesterol analysis*		Antihypertensive medicine use available?	Lipid-lowering medicine use available?	Type of blood pressure measurement device†	Lipids standardisation programme used?‡	Notes
					Female	Male	Female	Male					
1	England	1991-1992	Health Survey for England	20-79	2,581	2,464			Yes	No	Digital oscillometric		
2	England	1993	Health Survey for England	20-79	6,252	5,889			Yes	No	Digital oscillometric		
3	England	1994	Health Survey for England	20-79	5,843	5,343			Yes	No	Digital oscillometric		
4	England	1995	Health Survey for England	20-79	5,779	5,268			No	No	Digital oscillometric		
5	England	1996	Health Survey for England	20-79	6,124	5,540			No	No	Digital oscillometric		
6	England	1997	Health Survey for England	20-79	3,215	2,995			No	No	Digital oscillometric		
7	England	1998	Health Survey for England	20-79	5,774	5,135	4,576	4,291	Yes	Yes	Digital oscillometric	Yes (CDC/CRMLN)	
8	England	2000	Health Survey for England	20-79	2,829	2,534			Yes	No	Digital oscillometric		1
9	England	2001	Health Survey for England	20-79	5,289	4,682			Yes	No	Digital oscillometric		
10	England	2002	Health Survey for England	20-79	3,140	2,537			Yes	No	Digital oscillometric		
11	England	2003	Health Survey for England	20-79	4,899	4,272	3,711	3,303	Yes	Yes	Digital oscillometric	Yes (CDC/CRMLN)	
12	England	2005	Health Survey for England	20-79	2,984	2,579	787	726	Yes	Yes	Digital oscillometric	Yes (CDC/CRMLN)	1
13	England	2006	Health Survey for England	20-79	4,400	3,815	3,311	2,899	Yes	Yes	Digital oscillometric	Yes (CDC/CRMLN)	
14	England	2007	Health Survey for England	20-79	2,128	1,823			Yes	No	Digital oscillometric		
15	England	2008	Health Survey for England	20-79	4,512	3,825	3,179	2,795	Yes	Yes	Digital oscillometric	Yes (CDC/CRMLN)	
16	England	2009	Health Survey for England	20-79	1,352	1,204	1,004	912	Yes	Yes	Digital oscillometric	Yes (CDC/CRMLN)	
17	England	2010	Health Survey for England	20-79	2,377	1,930	1,736	1,425	Yes	Yes	Digital oscillometric	Yes (CDC/CRMLN)	
18	England	2011	Health Survey for England	20-79	2,439	1,993	1,760	1,454	Yes	Yes	Digital oscillometric	Yes (CDC/CRMLN)	
19	England	2012	Health Survey for England	20-79	2,310	1,926	1,748	1,485	Yes	Yes	Digital oscillometric	Yes (CDC/CRMLN)	
20	England	2013	Health Survey for England	20-79	2,616	2,189	1,949	1,733	Yes	Yes	Digital oscillometric	Yes (CDC/CRMLN)	
21	England	2014	Health Survey for England	20-79	2,368	1,983	1,740	1,541	Yes	Yes	Digital oscillometric	Yes (CDC/CRMLN)	
22	England	2015	Health Survey for England	20-79	2,290	1,898	1,711	1,477	Yes	Yes	Digital oscillometric	Yes (CDC/CRMLN)	
23	England	2016	Health Survey for England	20-79	2,131	1,740	1,666	1,393	Yes	Yes	Digital oscillometric	Yes (CDC/CRMLN)	
24	England	2017	Health Survey for England	20-79	2,271	1,754	1,755	1,394	Yes	Yes	Digital oscillometric	Yes (CDC/CRMLN)	
25	England	2018	Health Survey for England	20-79	2,068	1,674	1,582	1,323	Yes	Yes	Digital oscillometric	Yes (other)	
26	England	2019	Health Survey for England	20-79	2,060	1,680	1,571	1,294	Yes	Yes	Digital oscillometric	Yes (other)	
27	England	2021	Health Survey for England	20-79	719	594	551	455	Yes	Yes	Digital oscillometric	Yes (other)	
28	Finland	1992	The National FINRISK Study	30-59	2,213	2,162	2,193	2,159	Yes	Yes	Standard mercury	Yes (WHO-LRC)	
29	Finland	1997	The National FINRISK Study	30-59	2,697	2,560	2,682	2,553	Yes	Yes	Standard mercury	Yes (WHO-LRC)	
30	Finland	2000-2001	Health 2000 Survey	30-59	1,945	1,889	1,933	1,881	Yes	Yes	Standard mercury	Yes (other)	
31	Finland	2002	The National FINRISK Study	30-59	3,050	2,677	3,047	2,671	Yes	Yes	Standard mercury	Yes (WHO-LRC)	
32	Finland	2007	The National FINRISK Study	30-59	1,855	1,696	1,850	1,690	Yes	Yes	Standard mercury	Yes (WHO-LRC)	
33	Finland	2012	The National FINRISK Study	30-59	1,647	1,531	1,646	1,531	Yes	Yes	Standard mercury	Yes (WHO-LRC)	
34	Finland	2017	The FinHealth Survey	30-59	1,486	1,376	1,482	1,373	Yes	Yes	Standard mercury	Yes (CDC/CRMLN)	
35	Finland	2022-2023	Healthy Finland Survey	30-59	1,312	1,234	1,307	1,231	Yes	No	Digital oscillometric	Yes (CDC/CRMLN)	
36	Japan	1990	National Nutrition Survey and National Cardiovascular Survey	20-79	4,205	3,206	3,603	2,688	Yes	No	Standard mercury	Yes (CDC/CRMLN)	
37	Japan	1991	National Nutrition Survey	20-79	3,978	3,068	3,670	2,781	Yes	No	Standard mercury	Yes (CDC/CRMLN)	
38	Japan	1992	National Nutrition Survey	20-79	3,751	2,896	3,244	2,367	Yes	No	Standard mercury	Yes (CDC/CRMLN)	
39	Japan	1993	National Nutrition Survey	20-79	3,537	2,640	3,019	2,165	Yes	No	Standard mercury	Yes (CDC/CRMLN)	
40	Japan	1994	National Nutrition Survey	20-79	3,373	2,485	2,175	1,525	Yes	No	Standard mercury	Yes (CDC/CRMLN)	
41	Japan	1995	National Nutrition Survey	20-79	3,291	2,427	2,105	1,469	Yes	No	Standard mercury	Yes (CDC/CRMLN)	
42	Japan	1996	National Nutrition Survey	20-79	3,222	2,383	2,715	1,857	Yes	No	Standard mercury	Yes (CDC/CRMLN)	
43	Japan	1997	National Nutrition Survey	20-79	3,159	2,336	2,923	2,096	Yes	No	Standard mercury	Yes (CDC/CRMLN)	
44	Japan	1998	National Nutrition Survey	20-79	3,343	2,558	2,987	2,224	Yes	No	Standard mercury	Yes (CDC/CRMLN)	
45	Japan	1999	National Nutrition Survey	20-79	2,733	1,996	2,434	1,730	Yes	No	Standard mercury	Yes (CDC/CRMLN)	
46	Japan	2000	National Nutrition Survey and National Cardiovascular Survey	20-79	2,699	2,114	2,493	1,921	Yes	No	Standard mercury	Yes (CDC/CRMLN)	
47	Japan	2001	National Nutrition Survey	20-79	2,672	1,920	2,512	1,813	Yes	No	Standard mercury	Yes (CDC/CRMLN)	
48	Japan	2002	National Nutrition Survey	20-79	2,479	1,904	2,370	1,810	Yes	No	Standard mercury	Yes (CDC/CRMLN)	
49	Japan	2003	National Health and Nutrition Survey	20-79	2,476	1,826	2,340	1,729	Yes	Yes	Standard mercury	Yes (CDC/CRMLN)	
50	Japan	2004	National Health and Nutrition Survey	20-79	1,810	1,399	1,732	1,320	Yes	Yes	Standard mercury	Yes (CDC/CRMLN)	
51	Japan	2005	National Health and Nutrition Survey	20-79	1,791	1,381	1,718	1,309	Yes	Yes	Standard mercury	Yes (CDC/CRMLN)	
52	Japan	2006	National Health and Nutrition Survey	20-79	1,967	1,539	1,876	1,462	Yes	Yes	Standard mercury	Yes (CDC/CRMLN)	
53	Japan	2007	National Health and Nutrition Survey	20-79	1,786	1,436	1,677	1,353	Yes	Yes	Standard mercury	Yes (CDC/CRMLN)	
54	Japan	2008	National Health and Nutrition Survey	20-79	1,946	1,562	1,873	1,481	Yes	Yes	Standard mercury	Yes (CDC/CRMLN)	
55	Japan	2009	National Health and Nutrition Survey	20-79	1,814	1,509	1,745	1,462	Yes	Yes	Standard mercury	Yes (CDC/CRMLN)	
56	Japan	2010	National Health and Nutrition Survey	20-79	1,679	1,372	1,591	1,301	Yes	Yes	Standard mercury	Yes (CDC/CRMLN)	
57	Japan	2011	National Health and Nutrition Survey	20-79	1,539	1,280	1,442	1,188	Yes	Yes	Standard mercury	Yes (CDC/CRMLN)	
58	Japan	2012	National Health and Nutrition Survey	20-79	6,175	5,033	5,813	4,742	Yes	Yes	Standard mercury	Yes (CDC/CRMLN)	
59	Japan	2013	National Health and Nutrition Survey	20-79	1,429	1,196	1,321	1,119	Yes	Yes	Standard mercury	Yes (CDC/CRMLN)	
60	Japan	2014	National Health and Nutrition Survey	20-79	1,468	1,237	1,389	1,181	Yes	Yes	Standard mercury	Yes (CDC/CRMLN)	
61	Japan	2015	National Health and Nutrition Survey	20-79	1,403	1,146	1,327	1,075	Yes	Yes	Standard mercury	Yes (CDC/CRMLN)	
62	Japan	2016	National Health and Nutrition Survey	20-79	4,766	4,021	4,509	3,776	Yes	Yes	Standard mercury	Yes (CDC/CRMLN)	

	Country	Study years	Survey/Study name	Age range used in this analysis	Sample size used in blood pressure analysis*		Sample size used in cholesterol analysis*		Antihypertensive medicine use available?	Lipid-lowering medicine use available?	Type of blood pressure measurement device†	Lipids standardisation programme used?‡	Notes
					Female	Male	Female	Male					
63	Japan	2017	National Health and Nutrition Survey	20-79	1,235	1,044	1,160	978	Yes	Yes	Standard mercury	Yes (CDC/CRMLN)	
64	Japan	2018	National Health and Nutrition Survey	20-79	1,223	1,033	1,160	970	Yes	Yes	Standard mercury	Yes (CDC/CRMLN)	
65	Japan	2019	National Health and Nutrition Survey	20-79	1,015	865	943	815	Yes	Yes	Standard mercury	Yes (CDC/CRMLN)	
66	Japan	2022	National Health and Nutrition Survey	20-79	778	715	725	674	Yes	Yes	Standard mercury	Yes (CDC/CRMLN)	
67	South Korea	1998	Korea National Health and Nutrition Examination Survey	20-79	3,539	3,041	3,259	2,742	Yes	No	Standard mercury	No	
68	South Korea	2001	Korea National Health and Nutrition Examination Survey	20-79	2,770	2,266	2,538	2,133	Yes	No	Standard mercury	No	
69	South Korea	2005	Korea National Health and Nutrition Examination Survey	20-79	2,618	2,076	2,544	2,002	Yes	Yes	Standard mercury	No	
70	South Korea	2007	Korea National Health and Nutrition Examination Survey	20-79	1,408	1,114	1,308	1,023	Yes	Yes	Standard mercury	No	
71	South Korea	2008	Korea National Health and Nutrition Examination Survey	20-79	3,163	2,489	3,001	2,360	Yes	Yes	Standard mercury	Yes (CDC/CRMLN)	
72	South Korea	2009	Korea National Health and Nutrition Examination Survey	20-79	3,408	2,811	3,217	2,676	Yes	Yes	Standard mercury	Yes (CDC/CRMLN)	
73	South Korea	2010	Korea National Health and Nutrition Examination Survey	20-79	2,849	2,366	2,663	2,239	Yes	Yes	Standard mercury	Yes (CDC/CRMLN)	
74	South Korea	2011	Korea National Health and Nutrition Examination Survey	20-79	2,823	2,298	2,681	2,177	Yes	Yes	Standard mercury	Yes (CDC/CRMLN)	
75	South Korea	2012	Korea National Health and Nutrition Examination Survey	20-79	2,772	2,194	2,563	2,070	Yes	Yes	Standard mercury	Yes (CDC/CRMLN)	
76	South Korea	2013	Korea National Health and Nutrition Examination Survey	20-79	2,599	2,166	2,358	2,025	Yes	Yes	Standard mercury	Yes (CDC/CRMLN)	
77	South Korea	2014	Korea National Health and Nutrition Examination Survey	20-79	2,589	2,069	2,302	1,876	Yes	Yes	Standard mercury	Yes (CDC/CRMLN)	
78	South Korea	2015	Korea National Health and Nutrition Examination Survey	20-79	2,521	2,125	2,307	1,980	Yes	Yes	Standard mercury	Yes (CDC/CRMLN)	
79	South Korea	2016	Korea National Health and Nutrition Examination Survey	20-79	2,739	2,315	2,569	2,212	Yes	Yes	Standard mercury	Yes (CDC/CRMLN)	
80	South Korea	2017	Korea National Health and Nutrition Examination Survey	20-79	2,686	2,390	2,536	2,297	Yes	Yes	Standard mercury	Yes (CDC/CRMLN)	
81	South Korea	2018	Korea National Health and Nutrition Examination Survey	20-79	2,737	2,379	2,630	2,280	Yes	Yes	Standard mercury	Yes (CDC/CRMLN)	
82	South Korea	2019	Korea National Health and Nutrition Examination Survey	20-79	2,689	2,436	2,591	2,324	Yes	Yes	Standard mercury	Yes (CDC/CRMLN)	
83	South Korea	2020	Korea National Health and Nutrition Examination Survey	20-79	2,516	2,261	2,444	2,204	Yes	Yes	Standard mercury	Yes (CDC/CRMLN)	
84	South Korea	2021	Korea National Health and Nutrition Examination Survey	20-79	2,411	2,102	2,343	2,023	Yes	Yes	Standard mercury	Yes (CDC/CRMLN)	
85	South Korea	2022	Korea National Health and Nutrition Examination Survey	20-79	2,304	1,970	2,264	1,968	Yes	Yes	Digital oscillometric	Yes (CDC/CRMLN)	
86	South Korea	2023	Korea National Health and Nutrition Examination Survey	20-79	2,515	2,181	2,468	2,147	Yes	Yes	Digital oscillometric	Yes (CDC/CRMLN)	
87	South Korea	2024	Korea National Health and Nutrition Examination Survey	20-79	2,555	2,218	2,488	2,185	Yes	Yes	Digital oscillometric	Yes (CDC/CRMLN)	
88	Taiwan	1993-1996	Nutrition and Health Survey in Taiwan	20-79	1,333	1,183	1,244	1,126	Yes	Yes	Standard mercury	Yes (other)	
89	Taiwan	2005-2008	Nutrition and Health Survey in Taiwan	20-79	1,108	1,108	1,097	1,098	Yes	Yes	Digital oscillometric	Yes (other)	
90	Taiwan	2013-2016	Nutrition and Health Survey in Taiwan	20-79	1,233	1,263	1,208	1,245	Yes	Yes	Digital oscillometric	Yes (CDC/CRMLN)	
91	Taiwan	2017-2020	Nutrition and Health Survey in Taiwan	20-79	1,816	1,870	1,795	1,861	Yes	Yes	Digital oscillometric	Yes (CDC/CRMLN)	
92	Thailand	1991	Thailand National Health Examination Survey I	20-79	5,737	3,695			Yes	No	Standard mercury		
93	Thailand	1997	Thailand National Health Examination Survey II	20-59	1,547	864			No	No	Digital oscillometric		
94	Thailand	2000	InterASIA	40-79	2,399	1,398	2,299	1,348	Yes	Yes	Standard mercury	Yes (CDC/CRMLN)	
95	Thailand	2004	Thailand National Health Examination Survey III	20-79	15,687	13,291			Yes	No	Standard mercury		
96	Thailand	2009	Thailand National Health Examination Survey IV	20-79	8,143	6,792	8,009	6,714	Yes	Yes	Digital oscillometric	Unknown	
97	Thailand	2014	Thailand National Health Examination Survey V	20-79	8,770	5,940	8,349	5,671	Yes	Yes	Digital oscillometric	Unknown	
98	Thailand	2019-2020	Thailand National Health Examination Survey VI	20-79	10,362	6,880	10,328	6,859	Yes	Yes	Digital oscillometric	Unknown	
99	United States of America	1988-1994	US NHANES III	20-79	7,084	6,666	6,795	6,446	Yes	Yes	Standard mercury	Yes (CDC/CRMLN)	
100	United States of America	1999-2000	US NHANES 1999-2000	20-79	1,749	1,778	1,702	1,698	Yes	Yes	Standard mercury	Yes (CDC/CRMLN)	
101	United States of America	2001-2002	US NHANES 2001-2002	20-79	1,799	1,932	1,778	1,911	Yes	Yes	Standard mercury	Yes (CDC/CRMLN)	
102	United States of America	2003-2004	US NHANES 2003-2004	20-79	1,708	1,872	1,750	1,888	Yes	Yes	Standard mercury	Yes (CDC/CRMLN)	
103	United States of America	2005-2006	US NHANES 2005-2006	20-79	1,725	1,926	1,717	1,906	Yes	Yes	Standard mercury	Yes (CDC/CRMLN)	
104	United States of America	2007-2008	US NHANES 2007-2008	20-79	2,344	2,392	2,312	2,346	Yes	Yes	Standard mercury	Yes (CDC/CRMLN)	
105	United States of America	2009-2010	US NHANES 2009-2010	20-79	2,549	2,519	2,529	2,457	Yes	Yes	Standard mercury	Yes (CDC/CRMLN)	
106	United States of America	2011-2012	US NHANES 2011-2012	20-79	2,171	2,271	2,122	2,190	Yes	Yes	Standard mercury	Yes (CDC/CRMLN)	
107	United States of America	2013-2014	US NHANES 2013-2014	20-79	2,393	2,328	2,380	2,292	Yes	Yes	Standard mercury	Yes (CDC/CRMLN)	
108	United States of America	2015-2016	US NHANES 2015-2016	20-79	2,367	2,294	2,317	2,215	Yes	Yes	Standard mercury	Yes (CDC/CRMLN)	
109	United States of America	2017-2018	US NHANES 2017-2018	20-79	2,194	2,149	2,180	2,093	Yes	Yes	Standard mercury	Yes (CDC/CRMLN)	
110	United States of America	2021-2023	US NHANES 2021-2023	20-79	2,806	2,366	2,629	2,222	Yes	Yes	Digital oscillometric	Yes (CDC/CRMLN)	

* Sample size is after cleaning data.

† Blood pressure measurement devices include:
Standard: standard mercury sphygmomanometer or aneroid sphygmomanometer; Digital: digital oscillometric device

‡ Participating to a lipid standardisation programme or quality control schemes abbreviations:
CDC/CRMLN = CDC/Cholesterol Reference Method Laboratory Network Lipid Standardization Program; Other = Participation in internal and external quality control schemes;
WHO-LRC = WHO Regional Lipid Reference Centre in Prague; Unknown = Information unavailable or lipids measured in multiple laboratories.

1. Medicine use only collected for participants aged 60+.

Appendix Table 2. Data used for analysis in each country.

Country	Blood pressure		Cholesterol	
	**Years of data**	**Sex-age group-BMI range dropped***	**Years of data**	**Sex-age group-BMI range dropped***
Japan	1990-2022	Females and males, all age groups, class II and III obesity	1990-2022	Females and males, all age groups, class II and III obesity; females 20-39 years class I obesity
South Korea	1998-2024	Females and males, all age groups, class II and III obesity	1998-2024	Females and males, all age groups, class II and III obesity
Taiwan	1995-2019	Females and males, all age groups, class II and III obesity; females and males 20-39 years, class I obesity	1995-2019	Females and males, all age groups, class II and III obesity; females and males 20-39 years class I obesity
Thailand	1995-2020	Males 60-79 years, class II and III obesity	2000-2020	Males 60-79 years, class II and III obesity
Finland	1992-2023	Males 30-39 years, class II and III obesity	1992-2023	Males 30-39 years, class II and III obesity
England	1992-2021	NA	1998-2021	NA
USA	1991-2022	NA	1991-2022	NA

* As stated in Methods, these time series had fewer than three observations with at least 25 participants.

Appendix Table 3. Regression coefficients and associated 95% confidence interval and *P* for linear models for cholesterol and blood pressure over time in normal BMI.

The confidence intervals and probabilities *P* were obtained using the simulation with 10,000 draws as described in Methods.

Country	Age group	Non-HDL cholesterol				HDL cholesterol				Systolic blood pressure			
		Slope and 95% CI	Probability (*P*)*	Predicted value at earliest year and 95% CI	Predicted value at latest year and 95% CI	Slope and 95% CI	Probability (*P*)*	Predicted value at earliest year and 95% CI	Predicted value at latest year and 95% CI	Slope and 95% CI	Probability (*P*)*	Predicted value at earliest year and 95% CI	Predicted value at latest year and 95% CI
**Females**													
England	20-39	-0.0326 [-0.0374, -0.0278]	>.999	3.35 [3.29, 3.41]	2.60 [2.54, 2.67]	0.0158 [0.0129, 0.0188]	>.999	1.51 [1.48, 1.54]	1.87 [1.84, 1.91]	-0.5 [-0.6, -0.5]	>.999	121.8 [121.4, 122.1]	106.6 [105.9, 107.2]
England	40-59	-0.0360 [-0.0411, -0.0308]	>.999	4.01 [3.94, 4.07]	3.18 [3.11, 3.25]	0.0154 [0.0129, 0.0179]	>.999	1.66 [1.63, 1.69]	2.01 [1.98, 2.05]	-0.5 [-0.6, -0.5]	>.999	129.3 [128.7, 129.8]	113.3 [112.6, 114.0]
England	60-79	-0.0576 [-0.0647, -0.0508]	>.999	4.73 [4.64, 4.83]	3.41 [3.32, 3.49]	0.0224 [0.0187, 0.0261]	>.999	1.62 [1.57, 1.66]	2.13 [2.09, 2.18]	-0.9 [-0.9, -0.8]	>.999	148.9 [148.0, 149.8]	124.0 [123.0, 125.1]
Finland	20-39	-0.0165 [-0.0196, -0.0134]	>.999	3.48 [3.43, 3.53]	2.96 [2.90, 3.03]	0.0023 [0.0008, 0.0038]	>.999	1.58 [1.56, 1.60]	1.65 [1.61, 1.68]	-0.1 [-0.1, -0.0]	0.998	117.4 [116.6, 118.2]	114.8 [113.5, 116.0]
Finland	40-59	-0.0154 [-0.0185, -0.0123]	>.999	3.95 [3.90, 4.00]	3.47 [3.41, 3.54]	0.0049 [0.0037, 0.0062]	>.999	1.61 [1.59, 1.63]	1.76 [1.74, 1.79]	-0.2 [-0.3, -0.2]	>.999	128.5 [127.6, 129.3]	120.7 [119.6, 121.9]
Japan	20-39	-0.0058 [-0.0081, -0.0036]	>.999	3.18 [3.15, 3.21]	2.99 [2.94, 3.04]	0.0050 [0.0038, 0.0062]	>.999	1.62 [1.61, 1.64]	1.78 [1.76, 1.81]	-0.3 [-0.4, -0.3]	>.999	116.1 [115.7, 116.5]	105.7 [105.0, 106.4]
Japan	40-59	-0.0050 [-0.0067, -0.0033]	>.999	3.78 [3.76, 3.80]	3.62 [3.58, 3.66]	0.0110 [0.0102, 0.0118]	>.999	1.55 [1.54, 1.56]	1.90 [1.88, 1.92]	-0.4 [-0.5, -0.4]	>.999	131.3 [130.9, 131.7]	117.1 [116.5, 117.8]
Japan	60-79	-0.0075 [-0.0131, -0.0018]	0.995	4.13 [4.00, 4.25]	3.89 [3.83, 3.95]	0.0116 [0.0108, 0.0125]	>.999	1.40 [1.39, 1.42]	1.78 [1.76, 1.79]	-0.5 [-0.5, -0.4]	>.999	145.3 [144.7, 145.8]	129.9 [129.3, 130.5]
South Korea	20-39	0.0072 [0.0045, 0.0099]	>.999	3.03 [2.99, 3.07]	3.22 [3.18, 3.26]	0.0160 [0.0149, 0.0171]	>.999	1.23 [1.22, 1.25]	1.65 [1.63, 1.67]	-0.1 [-0.2, -0.1]	>.999	107.3 [106.8, 107.8]	103.9 [103.5, 104.3]
South Korea	40-59	-0.0034 [-0.0058, -0.0011]	0.998	3.69 [3.65, 3.73]	3.60 [3.57, 3.63]	0.0163 [0.0154, 0.0172]	>.999	1.19 [1.17, 1.20]	1.61 [1.60, 1.62]	-0.3 [-0.3, -0.3]	>.999	118.4 [117.7, 119.2]	110.9 [110.4, 111.3]
South Korea	60-79	-0.0383 [-0.0417, -0.0349]	>.999	4.36 [4.30, 4.42]	3.37 [3.33, 3.41]	0.0152 [0.0141, 0.0163]	>.999	1.10 [1.08, 1.11]	1.49 [1.48, 1.50]	-0.5 [-0.5, -0.4]	>.999	134.7 [133.4, 136.0]	122.8 [122.1, 123.5]
Taiwan	20-39	0.0004 [-0.0088, 0.0094]	0.53	2.97 [2.81, 3.12]	2.98 [2.86, 3.10]	0.0001 [-0.0030, 0.0034]	0.54	1.58 [1.52, 1.63]	1.58 [1.54, 1.62]	-0.1 [-0.2, -0.0]	0.996	105.8 [104.0, 107.8]	102.5 [101.3, 103.7]
Taiwan	40-59	-0.0114 [-0.0233, 0.0002]	0.97	3.61 [3.40, 3.83]	3.33 [3.22, 3.45]	-0.0009 [-0.0050, 0.0032]	0.67	1.62 [1.55, 1.70]	1.60 [1.56, 1.65]	-0.4 [-0.5, -0.2]	>.999	117.7 [115.5, 119.8]	108.7 [107.1, 110.3]
Taiwan	60-79	-0.0299 [-0.0428, -0.0168]	>.999	4.20 [3.95, 4.45]	3.49 [3.38, 3.60]	-0.0010 [-0.0066, 0.0047]	0.64	1.53 [1.42, 1.63]	1.50 [1.46, 1.55]	-0.6 [-0.8, -0.5]	>.999	137.2 [134.3, 140.1]	121.7 [119.6, 123.8]
Thailand	20-39	0.0057 [-0.0048, 0.0163]	0.86	3.64 [3.56, 3.72]	3.70 [3.63, 3.78]	0.0235 [0.0189, 0.0280]	>.999	1.29 [1.26, 1.32]	1.55 [1.51, 1.58]	0.0 [-0.0, 0.1]	0.76	109.3 [108.6, 109.9]	109.7 [108.9, 110.5]
Thailand	40-59	0.0042 [-0.0026, 0.0109]	0.89	4.01 [3.91, 4.10]	4.09 [4.02, 4.16]	0.0159 [0.0136, 0.0183]	>.999	1.20 [1.16, 1.23]	1.52 [1.49, 1.54]	0.2 [0.1, 0.3]	>.999	116.1 [115.1, 117.0]	121.9 [120.8, 123.0]
Thailand	60-79	-0.0222 [-0.0321, -0.0121]	>.999	4.60 [4.44, 4.75]	4.15 [4.07, 4.24]	0.0159 [0.0133, 0.0186]	>.999	1.10 [1.07, 1.13]	1.42 [1.39, 1.45]	0.3 [0.2, 0.4]	>.999	126.8 [125.0, 128.7]	134.9 [133.6, 136.2]
USA	20-39	-0.0156 [-0.0194, -0.0117]	>.999	3.29 [3.21, 3.36]	2.80 [2.74, 2.87]	0.0052 [0.0034, 0.0069]	>.999	1.49 [1.46, 1.52]	1.65 [1.62, 1.68]	-0.0 [-0.1, 0.0]	0.76	108.2 [107.3, 109.1]	107.7 [106.8, 108.5]
USA	40-59	-0.0154 [-0.0206, -0.0101]	>.999	3.84 [3.74, 3.94]	3.36 [3.28, 3.45]	0.0053 [0.0031, 0.0076]	>.999	1.61 [1.57, 1.65]	1.77 [1.73, 1.82]	-0.1 [-0.2, 0.0]	0.97	118.2 [116.6, 119.8]	115.8 [114.5, 117.1]
USA	60-79	-0.0309 [-0.0374, -0.0244]	>.999	4.37 [4.25, 4.49]	3.41 [3.30, 3.53]	0.0067 [0.0043, 0.0092]	>.999	1.60 [1.55, 1.65]	1.80 [1.76, 1.85]	-0.4 [-0.5, -0.3]	>.999	138.9 [136.4, 141.2]	126.6 [124.5, 128.7]
**Males**													
England	20-39	-0.0248 [-0.0343, -0.0150]	>.999	3.57 [3.46, 3.68]	3.00 [2.87, 3.13]	0.0114 [0.0088, 0.0140]	>.999	1.33 [1.29, 1.36]	1.59 [1.55, 1.63]	-0.4 [-0.4, -0.4]	>.999	130.9 [130.4, 131.3]	119.4 [118.6, 120.1]
England	40-59	-0.0321 [-0.0404, -0.0240]	>.999	4.28 [4.18, 4.38]	3.54 [3.43, 3.65]	0.0114 [0.0081, 0.0146]	>.999	1.38 [1.35, 1.42]	1.65 [1.60, 1.69]	-0.4 [-0.5, -0.4]	>.999	133.9 [133.3, 134.5]	122.1 [121.2, 123.1]
England	60-79	-0.0553 [-0.0626, -0.0479]	>.999	4.34 [4.24, 4.44]	3.07 [2.98, 3.17]	0.0180 [0.0150, 0.0211]	>.999	1.35 [1.31, 1.39]	1.76 [1.72, 1.80]	-0.7 [-0.8, -0.7]	>.999	147.3 [146.4, 148.4]	126.5 [125.3, 127.7]
Finland	20-39	-0.0141 [-0.0194, -0.0087]	>.999	3.88 [3.81, 3.96]	3.45 [3.33, 3.56]	0.0022 [0.0003, 0.0038]	0.991	1.37 [1.35, 1.39]	1.44 [1.40, 1.47]	-0.2 [-0.2, -0.1]	>.999	127.0 [126.1, 128.0]	122.3 [120.8, 123.8]
Finland	40-59	-0.0216 [-0.0261, -0.0171]	>.999	4.45 [4.39, 4.52]	3.78 [3.69, 3.88]	0.0036 [0.0021, 0.0051]	>.999	1.39 [1.37, 1.41]	1.50 [1.47, 1.53]	-0.2 [-0.3, -0.1]	>.999	132.9 [131.9, 134.0]	126.5 [125.3, 127.8]
Japan	20-39	-0.0081 [-0.0109, -0.0053]	>.999	3.55 [3.51, 3.59]	3.29 [3.23, 3.35]	0.0045 [0.0032, 0.0058]	>.999	1.39 [1.38, 1.41]	1.54 [1.51, 1.57]	-0.3 [-0.4, -0.3]	>.999	125.5 [125.0, 126.0]	115.3 [114.4, 116.2]
Japan	40-59	0.0016 [-0.0008, 0.0039]	0.91	3.82 [3.79, 3.85]	3.87 [3.81, 3.92]	0.0079 [0.0070, 0.0089]	>.999	1.37 [1.36, 1.38]	1.62 [1.60, 1.65]	-0.4 [-0.4, -0.3]	>.999	135.6 [135.1, 136.2]	123.9 [123.0, 124.7]
Japan	60-79	-0.0026 [-0.0066, 0.0013]	0.90	3.72 [3.63, 3.80]	3.63 [3.59, 3.68]	0.0079 [0.0068, 0.0091]	>.999	1.31 [1.28, 1.33]	1.56 [1.54, 1.58]	-0.4 [-0.5, -0.4]	>.999	147.6 [147.0, 148.2]	133.6 [132.9, 134.2]
South Korea	20-39	0.0048 [0.0018, 0.0077]	0.999	3.33 [3.28, 3.38]	3.45 [3.41, 3.49]	0.0106 [0.0096, 0.0115]	>.999	1.13 [1.12, 1.15]	1.41 [1.40, 1.42]	-0.1 [-0.2, -0.1]	>.999	116.7 [116.1, 117.2]	113.5 [113.0, 113.9]
South Korea	40-59	0.0030 [-0.0001, 0.0061]	0.97	3.74 [3.69, 3.79]	3.82 [3.77, 3.86]	0.0079 [0.0069, 0.0090]	>.999	1.13 [1.12, 1.15]	1.34 [1.32, 1.35]	-0.3 [-0.3, -0.2]	>.999	124.0 [123.2, 124.9]	116.5 [115.9, 117.1]
South Korea	60-79	-0.0235 [-0.0269, -0.0201]	>.999	3.86 [3.80, 3.92]	3.25 [3.21, 3.29]	0.0070 [0.0059, 0.0082]	>.999	1.12 [1.10, 1.14]	1.30 [1.29, 1.31]	-0.5 [-0.6, -0.4]	>.999	134.7 [133.3, 136.1]	122.2 [121.4, 123.0]
Taiwan	20-39	-0.0068 [-0.0166, 0.0030]	0.92	3.41 [3.25, 3.56]	3.24 [3.11, 3.38]	-0.0042 [-0.0071, -0.0012]	0.997	1.42 [1.37, 1.46]	1.32 [1.27, 1.36]	-0.2 [-0.3, -0.0]	0.996	117.4 [115.0, 119.8]	113.3 [111.8, 114.7]
Taiwan	40-59	-0.0036 [-0.0164, 0.0086]	0.72	3.76 [3.55, 3.99]	3.68 [3.53, 3.82]	-0.0032 [-0.0072, 0.0010]	0.93	1.36 [1.29, 1.42]	1.28 [1.23, 1.34]	-0.1 [-0.3, 0.0]	0.92	119.5 [116.6, 122.3]	116.7 [114.9, 118.4]
Taiwan	60-79	-0.0170 [-0.0257, -0.0084]	>.999	3.84 [3.68, 4.01]	3.43 [3.33, 3.54]	-0.0030 [-0.0075, 0.0014]	0.92	1.38 [1.29, 1.46]	1.30 [1.27, 1.34]	-0.5 [-0.7, -0.2]	>.999	135.0 [130.3, 139.7]	123.2 [121.3, 125.2]
Thailand	20-39	0.0237 [0.0101, 0.0370]	>.999	3.69 [3.61, 3.77]	3.95 [3.84, 4.06]	0.0125 [0.0077, 0.0174]	>.999	1.24 [1.20, 1.27]	1.37 [1.34, 1.41]	0.2 [0.2, 0.3]	>.999	115.5 [114.5, 116.5]	121.8 [120.9, 122.7]
Thailand	40-59	0.0149 [0.0052, 0.0243]	0.998	3.87 [3.77, 3.97]	4.17 [4.04, 4.29]	0.0114 [0.0091, 0.0137]	>.999	1.09 [1.06, 1.12]	1.32 [1.29, 1.34]	0.3 [0.2, 0.4]	>.999	118.4 [117.2, 119.8]	127.6 [126.0, 129.2]
Thailand	60-79	-0.0122 [-0.0205, -0.0037]	0.998	4.16 [4.03, 4.28]	3.91 [3.84, 3.98]	0.0119 [0.0093, 0.0145]	>.999	1.05 [1.02, 1.09]	1.29 [1.26, 1.32]	0.4 [0.3, 0.5]	>.999	127.0 [125.0, 129.1]	138.6 [137.2, 140.1]
USA	20-39	-0.0156 [-0.0206, -0.0106]	>.999	3.55 [3.46, 3.64]	3.07 [2.98, 3.16]	0.0037 [0.0020, 0.0053]	>.999	1.28 [1.25, 1.31]	1.40 [1.37, 1.43]	-0.0 [-0.1, 0.0]	0.92	116.9 [115.9, 117.9]	115.7 [114.8, 116.6]
USA	40-59	-0.0218 [-0.0289, -0.0147]	>.999	4.19 [4.05, 4.33]	3.51 [3.40, 3.63]	0.0031 [0.0007, 0.0055]	0.994	1.36 [1.31, 1.40]	1.45 [1.41, 1.49]	-0.0 [-0.1, 0.0]	0.87	121.8 [120.3, 123.4]	120.3 [118.8, 121.8]
USA	60-79	-0.0378 [-0.0441, -0.0316]	>.999	4.26 [4.14, 4.38]	3.09 [2.98, 3.20]	0.0075 [0.0044, 0.0106]	>.999	1.28 [1.22, 1.34]	1.51 [1.46, 1.56]	-0.1 [-0.3, -0.0]	0.991	133.0 [130.7, 135.2]	128.5 [126.6, 130.5]

* Probability of the slope being a true negative or a true positive

Appendix Table 4. Regression coefficients and associated 95% confidence interval and *P* for linear models for treatment over time in normal BMI.

The confidence intervals and probabilities *P* were obtained using the simulation with 10,000 draws as described in Methods.

Country	Age group	Lipid-lowering medicine use				Antihypertensive medicine use			
		Slope and 95% CI	Probability (*P*)*	Predicted value at earliest year and 95% CI	Predicted value at latest year and 95% CI	Slope and 95% CI	Probability (*P*)*	Predicted value at earliest year and 95% CI	Predicted value at latest year and 95% CI
**Females**									
England	20-39	10.04 [-2.11, 22.40]	0.95	0 [0, 0]	1 [0, 2]	2.32 [-4.02, 8.01]	0.80	0 [0, 0]	0 [0, 1]
England	40-59	-0.14 [-7.10, 4.21]	0.53	2 [1, 4]	2 [1, 3]	-0.09 [-1.63, 1.28]	0.55	4 [3, 5]	4 [3, 5]
England	60-79	4.40 [2.33, 6.57]	>.999	11 [8, 14]	25 [20, 29]	0.45 [-0.69, 2.09]	0.78	20 [15, 24]	22 [20, 25]
Finland	20-39	3.54 [-18.03, 23.20]	0.65	0 [0, 1]	0 [0, 1]	-3.69 [-16.70, 3.06]	0.84	1 [1, 4]	0 [0, 2]
Finland	40-59	10.22 [5.63, 27.47]	>.999	0 [0, 1]	5 [3, 17]	-0.31 [-2.00, 1.22]	0.65	6 [5, 7]	5 [4, 7]
Japan	20-39	6.47 [-6.23, 17.25]	0.87	0 [0, 1]	1 [0, 3]	3.57 [-1.90, 8.48]	0.92	0 [0, 0]	1 [0, 2]
Japan	40-59	2.26 [0.07, 4.24]	0.98	4 [3, 5]	6 [5, 8]	-0.95 [-1.76, -0.25]	0.995	8 [7, 8]	6 [5, 7]
Japan	60-79	3.59 [2.56, 4.61]	>.999	19 [17, 20]	32 [29, 34]	0.38 [0.03, 0.73]	0.98	32 [30, 33]	34 [33, 36]
South Korea	20-39	5.12 [-6.93, 15.45]	0.83	0 [0, 0]	0 [0, 1]	1.24 [-7.30, 10.85]	0.63	0 [0, 1]	0 [0, 1]
South Korea	40-59	13.31 [11.11, 17.22]	>.999	1 [1, 2]	14 [12, 17]	1.30 [0.26, 2.41]	0.994	7 [6, 8]	9 [8, 10]
South Korea	60-79	16.60 [15.01, 18.63]	>.999	5 [4, 6]	53 [50, 57]	2.72 [1.94, 3.53]	>.999	26 [23, 29]	42 [40, 44]
Taiwan	20-39	-0.71 [-13.85, 14.11]	0.54	0 [0, 2]	0 [0, 1]	0.12 [-17.10, 15.35]	0.51	0 [0, 2]	0 [0, 1]
Taiwan	40-59	4.42 [-1.04, 25.94]	0.95	1 [0, 4]	4 [2, 10]	4.60 [0.33, 12.84]	0.98	3 [0, 5]	8 [5, 12]
Taiwan	60-79	8.26 [2.58, 24.72]	>.999	3 [0, 8]	16 [10, 29]	2.98 [0.57, 5.73]	0.994	19 [12, 26]	32 [26, 38]
Thailand	20-39	14.09 [-2.50, 30.06]	0.96	0 [0, 1]	2 [0, 3]	-2.06 [-7.99, 1.02]	0.90	1 [1, 2]	0 [0, 1]
Thailand	40-59	13.51 [8.41, 24.57]	>.999	1 [0, 2]	14 [11, 21]	2.56 [1.09, 4.18]	>.999	4 [3, 5]	8 [7, 10]
Thailand	60-79	14.78 [11.54, 20.04]	>.999	3 [1, 5]	39 [35, 45]	5.12 [3.93, 6.43]	>.999	12 [9, 15]	38 [34, 41]
USA	20-39	-0.89 [-10.81, 11.64]	0.58	1 [0, 2]	0 [0, 2]	2.91 [-4.85, 8.58]	0.82	0 [0, 1]	1 [0, 3]
USA	40-59	3.49 [0.72, 6.45]	0.991	3 [2, 5]	9 [6, 13]	1.68 [-0.20, 3.56]	0.96	7 [5, 10]	12 [9, 15]
USA	60-79	5.07 [3.60, 6.57]	>.999	13 [10, 17]	42 [36, 48]	0.82 [-0.48, 2.03]	0.90	31 [26, 36]	37 [31, 42]
**Males**									
England	20-39	8.90 [-1.45, 24.55]	0.96	0 [0, 1]	1 [0, 4]	4.26 [-2.30, 9.55]	0.91	0 [0, 0]	1 [0, 2]
England	40-59	7.18 [2.96, 11.49]	0.994	2 [1, 4]	11 [6, 15]	2.74 [0.58, 4.57]	0.99	3 [3, 4]	7 [5, 10]
England	60-79	7.06 [5.05, 9.22]	>.999	12 [9, 15]	40 [35, 45]	1.46 [0.16, 2.80]	0.99	18 [14, 22]	25 [21, 28]
Finland	20-39	-1.88 [-22.53, 12.11]	0.60	0 [0, 2]	0 [0, 1]	1.45 [-2.79, 6.09]	0.73	1 [0, 2]	1 [1, 3]
Finland	40-59	4.18 [1.17, 7.18]	0.995	2 [1, 3]	6 [4, 9]	-0.60 [-2.90, 1.26]	0.73	7 [5, 8]	5 [3, 8]
Japan	20-39	4.08 [-9.86, 15.42]	0.75	0 [0, 1]	1 [0, 3]	5.67 [0.88, 9.78]	0.99	0 [0, 0]	1 [0, 3]
Japan	40-59	3.63 [-0.44, 7.21]	0.96	3 [2, 4]	6 [4, 8]	1.63 [0.84, 2.36]	>.999	8 [7, 8]	12 [11, 14]
Japan	60-79	5.11 [3.76, 6.45]	>.999	9 [8, 10]	21 [18, 23]	1.56 [1.16, 1.95]	>.999	29 [28, 31]	41 [39, 42]
South Korea	20-39	6.84 [-4.95, 19.60]	0.89	0 [0, 1]	1 [0, 2]	3.12 [-4.94, 11.91]	0.82	0 [0, 1]	1 [0, 1]
South Korea	40-59	14.19 [11.80, 17.05]	>.999	1 [1, 2]	15 [13, 18]	3.70 [2.61, 4.87]	>.999	6 [5, 7]	14 [12, 15]
South Korea	60-79	16.00 [13.74, 19.27]	>.999	3 [2, 4]	37 [33, 41]	4.54 [3.67, 5.47]	>.999	20 [18, 23]	45 [43, 48]
Taiwan	20-39	-1.13 [-17.18, 14.06]	0.57	0 [0, 2]	0 [0, 1]	2.97 [-11.37, 23.15]	0.65	0 [0, 2]	1 [0, 2]
Taiwan	40-59	14.33 [5.87, 27.97]	>.999	0 [0, 1]	4 [1, 8]	8.62 [3.78, 18.53]	>.999	2 [0, 5]	15 [9, 22]
Taiwan	60-79	10.13 [4.39, 29.48]	>.999	1 [0, 4]	12 [8, 25]	2.74 [0.52, 5.37]	0.992	18 [12, 25]	30 [25, 35]
Thailand	20-39	18.86 [3.13, 42.23]	0.99	0 [0, 0]	1 [0, 3]	5.30 [0.70, 9.92]	0.99	0 [0, 0]	1 [0, 2]
Thailand	40-59	12.38 [8.51, 19.97]	>.999	1 [0, 2]	9 [7, 13]	2.54 [0.95, 4.19]	0.999	3 [2, 4]	6 [5, 8]
Thailand	60-79	12.07 [8.92, 16.80]	>.999	3 [1, 5]	25 [21, 30]	5.63 [4.42, 6.96]	>.999	8 [6, 10]	31 [28, 34]
USA	20-39	8.18 [-0.08, 19.81]	0.97	0 [0, 1]	2 [0, 6]	-1.89 [-9.15, 5.64]	0.73	1 [0, 3]	1 [0, 2]
USA	40-59	5.51 [2.88, 8.61]	>.999	2 [1, 4]	12 [8, 17]	2.55 [0.32, 4.82]	0.99	5 [3, 7]	10 [7, 13]

Country	Age group	Lipid-lowering medicine use				Antihypertensive medicine use			
		Slope and 95% CI	Probability (*P*)*	Predicted value at earliest year and 95% CI	Predicted value at latest year and 95% CI	Slope and 95% CI	Probability (*P*)*	Predicted value at earliest year and 95% CI	Predicted value at latest year and 95% CI
USA	60-79	6.89 [5.00, 8.91]	>.999	10 [7, 13]	48 [40, 55]	0.61 [-0.82, 2.00]	0.80	28 [22, 33]	31 [26, 37]

* Probability of the slope being a true negative or a true positive

Appendix Table 5. Regression coefficients and associated 95% confidence intervals and *P* for linear models for the difference in cholesterol and blood pressure between obesity and overweight and normal BMI.

The confidence intervals and probabilities *P* were obtained using the simulation with 10,000 draws as described in Methods.

Country	Age group	BMI range	Non-HDL cholesterol				HDL cholesterol				Systolic blood pressure			
			Slope and 95% CI	Probability (*P*)*	Predicted value for difference at earliest year and 95% CI	Predicted value for difference at latest year and 95% CI	Slope and 95% CI	Probability (*P*)*	Predicted value for difference at earliest year and 95% CI	Predicted value for difference at latest year and 95% CI	Slope and 95% CI	Probability (*P*)*	Predicted value for difference at earliest year and 95% CI	Predicted value for difference at latest year and 95% CI
**Females**														
England	20-39	Overweight	0.0001 [-0.0094, 0.0096]	0.51	0.30 [0.18, 0.41]	0.30 [0.18, 0.42]	-0.0003 [-0.0055, 0.0048]	0.55	-0.14 [-0.20, -0.08]	-0.15 [-0.21, -0.08]	-0.0 [-0.1, 0.0]	0.62	3.5 [2.9, 4.1]	3.3 [2.3, 4.3]
England	20-39	Obesity Class I	-0.0147 [-0.0274, -0.0024]	0.99	0.78 [0.62, 0.94]	0.44 [0.28, 0.59]	-0.0013 [-0.0059, 0.0032]	0.72	-0.27 [-0.33, -0.21]	-0.30 [-0.36, -0.24]	-0.0 [-0.1, 0.0]	0.71	7.1 [6.3, 8.0]	6.6 [5.4, 7.8]
England	20-39	Obesity Class II/III	0.0097 [-0.0016, 0.0207]	0.95	0.64 [0.48, 0.81]	0.87 [0.72, 1.00]	-0.0094 [-0.0140, -0.0049]	>.999	-0.28 [-0.35, -0.22]	-0.50 [-0.56, -0.44]	-0.2 [-0.3, -0.2]	>.999	12.2 [11.0, 13.3]	5.4 [3.9, 6.8]
England	40-59	Overweight	0.0031 [-0.0045, 0.0108]	0.78	0.30 [0.20, 0.40]	0.37 [0.28, 0.47]	-0.0021 [-0.0055, 0.0013]	0.89	-0.15 [-0.19, -0.11]	-0.20 [-0.24, -0.15]	-0.0 [-0.1, 0.0]	0.93	5.0 [4.2, 5.8]	3.8 [2.7, 4.8]
England	40-59	Obesity Class I	-0.0097 [-0.0191, -0.0000]	0.98	0.65 [0.53, 0.78]	0.43 [0.31, 0.55]	-0.0031 [-0.0069, 0.0008]	0.94	-0.29 [-0.34, -0.24]	-0.36 [-0.41, -0.31]	-0.1 [-0.2, -0.1]	>.999	11.0 [9.9, 12.1]	6.9 [5.5, 8.4]
England	40-59	Obesity Class II/III	-0.0039 [-0.0148, 0.0068]	0.76	0.64 [0.49, 0.79]	0.55 [0.42, 0.69]	-0.0052 [-0.0090, -0.0014]	0.994	-0.36 [-0.41, -0.31]	-0.48 [-0.53, -0.43]	-0.2 [-0.3, -0.1]	>.999	14.3 [13.0, 15.7]	9.5 [8.0, 11.0]
England	60-79	Overweight	-0.0049 [-0.0140, 0.0043]	0.85	0.22 [0.10, 0.35]	0.11 [-0.00, 0.23]	-0.0058 [-0.0103, -0.0014]	0.996	-0.11 [-0.16, -0.06]	-0.24 [-0.30, -0.19]	-0.0 [-0.1, 0.0]	0.84	3.9 [2.7, 5.0]	2.7 [1.3, 4.2]
England	60-79	Obesity Class I	-0.0007 [-0.0121, 0.0106]	0.55	0.14 [-0.02, 0.29]	0.12 [-0.02, 0.26]	-0.0059 [-0.0106, -0.0012]	0.992	-0.24 [-0.29, -0.18]	-0.37 [-0.43, -0.31]	-0.1 [-0.2, -0.0]	0.997	8.1 [6.5, 9.6]	4.0 [2.3, 5.7]
England	60-79	Obesity Class II/III	-0.0121 [-0.0262, 0.0023]	0.95	0.13 [-0.06, 0.33]	-0.14 [-0.31, 0.03]	-0.0091 [-0.0141, -0.0041]	>.999	-0.27 [-0.34, -0.20]	-0.48 [-0.54, -0.41]	-0.2 [-0.4, -0.1]	0.999	10.4 [8.1, 12.7]	4.2 [1.9, 6.5]
Finland	20-39	Overweight	-0.0007 [-0.0065, 0.0052]	0.60	0.33 [0.23, 0.43]	0.31 [0.19, 0.42]	0.0002 [-0.0023, 0.0028]	0.57	-0.13 [-0.17, -0.10]	-0.13 [-0.18, -0.07]	-0.1 [-0.2, 0.0]	0.94	5.1 [3.6, 6.6]	2.8 [0.9, 4.7]
Finland	20-39	Obesity Class I	0.0016 [-0.0085, 0.0117]	0.62	0.57 [0.40, 0.73]	0.62 [0.41, 0.82]	0.0015 [-0.0023, 0.0053]	0.78	-0.27 [-0.33, -0.22]	-0.23 [-0.30, -0.15]	-0.1 [-0.3, 0.0]	0.97	9.7 [7.4, 12.1]	5.4 [2.3, 8.4]
Finland	20-39	Obesity Class II/III	0.0024 [-0.0128, 0.0177]	0.62	0.67 [0.37, 0.95]	0.74 [0.49, 1.00]	0.0023 [-0.0016, 0.0062]	0.88	-0.39 [-0.46, -0.32]	-0.32 [-0.39, -0.25]	0.0 [-0.2, 0.2]	0.63	12.3 [8.7, 15.9]	13.3 [9.9, 16.8]
Finland	40-59	Overweight	-0.0055 [-0.0100, -0.0010]	0.993	0.40 [0.33, 0.47]	0.23 [0.14, 0.32]	-0.0008 [-0.0026, 0.0009]	0.82	-0.14 [-0.16, -0.11]	-0.16 [-0.20, -0.12]	0.0 [-0.0, 0.1]	0.78	4.6 [3.3, 5.9]	5.6 [4.0, 7.2]
Finland	40-59	Obesity Class I	-0.0053 [-0.0114, 0.0008]	0.96	0.62 [0.52, 0.72]	0.45 [0.33, 0.57]	-0.0016 [-0.0036, 0.0004]	0.94	-0.25 [-0.28, -0.22]	-0.30 [-0.34, -0.26]	-0.1 [-0.2, 0.0]	0.97	10.4 [8.7, 12.2]	7.4 [5.4, 9.4]
Finland	40-59	Obesity Class II/III	-0.0116 [-0.0187, -0.0045]	>.999	0.75 [0.62, 0.86]	0.38 [0.24, 0.52]	0.0002 [-0.0021, 0.0025]	0.56	-0.37 [-0.41, -0.33]	-0.36 [-0.41, -0.32]	-0.0 [-0.2, 0.1]	0.75	15.5 [13.1, 17.9]	14.0 [11.6, 16.4]
Japan	20-39	Overweight	-0.0035 [-0.0090, 0.0020]	0.90	0.53 [0.45, 0.61]	0.42 [0.30, 0.54]	-0.0013 [-0.0036, 0.0009]	0.87	-0.19 [-0.23, -0.16]	-0.24 [-0.29, -0.19]	0.0 [-0.1, 0.1]	0.55	6.2 [5.1, 7.3]	6.4 [4.8, 8.0]
Japan	20-39	Obesity Class I									0.2 [0.1, 0.4]	0.997	12.0 [9.5, 14.5]	19.7 [15.9, 23.4]
Japan	40-59	Overweight	-0.0008 [-0.0043, 0.0027]	0.67	0.39 [0.34, 0.44]	0.36 [0.29, 0.44]	-0.0032 [-0.0047, -0.0016]	>.999	-0.15 [-0.17, -0.13]	-0.25 [-0.29, -0.22]	-0.0 [-0.1, 0.1]	0.63	8.4 [7.6, 9.4]	8.1 [6.6, 9.5]
Japan	40-59	Obesity Class I	-0.0021 [-0.0094, 0.0050]	0.72	0.51 [0.40, 0.61]	0.44 [0.28, 0.59]	-0.0054 [-0.0077, -0.0030]	>.999	-0.22 [-0.26, -0.18]	-0.39 [-0.44, -0.34]	-0.2 [-0.3, -0.1]	0.999	16.1 [14.1, 18.2]	10.2 [7.6, 12.7]
Japan	60-79	Overweight	-0.0061 [-0.0123, 0.0001]	0.97	0.24 [0.11, 0.37]	0.05 [-0.03, 0.12]	-0.0037 [-0.0049, -0.0024]	>.999	-0.06 [-0.08, -0.04]	-0.17 [-0.20, -0.15]	-0.1 [-0.1, 0.0]	0.97	5.2 [4.3, 6.2]	3.5 [2.4, 4.6]
Japan	60-79	Obesity Class I	-0.0209 [-0.0281, -0.0140]	>.999	0.46 [0.34, 0.57]	-0.21 [-0.35, -0.08]	-0.0027 [-0.0050, -0.0004]	0.99	-0.13 [-0.16, -0.09]	-0.21 [-0.26, -0.17]	-0.2 [-0.3, -0.1]	0.998	10.3 [8.3, 12.3]	4.7 [2.6, 7.0]
South Korea	20-39	Overweight	-0.0049 [-0.0107, 0.0009]	0.95	0.46 [0.37, 0.55]	0.33 [0.25, 0.41]	-0.0066 [-0.0087, -0.0045]	>.999	-0.05 [-0.09, -0.02]	-0.22 [-0.25, -0.19]	0.0 [-0.1, 0.1]	0.59	5.4 [4.1, 6.7]	5.6 [4.7, 6.5]
South Korea	20-39	Obesity Class I	-0.0010 [-0.0130, 0.0113]	0.56	0.67 [0.46, 0.87]	0.65 [0.49, 0.81]	-0.0119 [-0.0147, -0.0089]	>.999	-0.08 [-0.13, -0.03]	-0.39 [-0.43, -0.35]	0.3 [0.1, 0.4]	>.999	6.1 [4.0, 8.1]	12.9 [11.3, 14.5]
South Korea	40-59	Overweight	-0.0050 [-0.0094, -0.0006]	0.99	0.33 [0.25, 0.39]	0.19 [0.13, 0.26]	-0.0055 [-0.0070, -0.0039]	>.999	-0.04 [-0.07, -0.02]	-0.19 [-0.21, -0.16]	0.0 [-0.1, 0.1]	0.50	6.1 [4.8, 7.5]	6.1 [5.2, 7.0]
South Korea	40-59	Obesity Class I	-0.0125 [-0.0221, -0.0028]	0.994	0.43 [0.27, 0.60]	0.11 [-0.01, 0.23]	-0.0057 [-0.0089, -0.0025]	>.999	-0.08 [-0.13, -0.02]	-0.22 [-0.26, -0.18]	-0.2 [-0.3, 0.0]	0.97	14.4 [11.4, 17.5]	10.4 [8.5, 12.1]
South Korea	60-79	Overweight	-0.0092 [-0.0147, -0.0039]	>.999	0.18 [0.08, 0.27]	-0.06 [-0.13, 0.00]	-0.0038 [-0.0054, -0.0021]	>.999	-0.00 [-0.03, 0.03]	-0.10 [-0.12, -0.08]	0.0 [-0.1, 0.1]	0.73	2.4 [0.5, 4.4]	3.2 [2.2, 4.3]
South Korea	60-79	Obesity Class I	-0.0105 [-0.0217, 0.0008]	0.96	0.17 [-0.02, 0.37]	-0.10 [-0.23, 0.04]	-0.0062 [-0.0095, -0.0029]	>.999	0.01 [-0.05, 0.07]	-0.16 [-0.19, -0.12]	-0.2 [-0.4, 0.1]	0.93	8.4 [3.9, 12.7]	3.8 [1.8, 6.0]
Taiwan	20-39	Overweight	0.0212 [-0.0012, 0.0435]	0.97	-0.15 [-0.56, 0.25]	0.36 [0.12, 0.60]	-0.0149 [-0.0364, 0.0075]	0.90	0.05 [-0.39, 0.48]	-0.31 [-0.43, -0.18]	0.3 [0.0, 0.5]	0.98	2.3 [-2.1, 6.7]	8.6 [5.4, 11.9]
Taiwan	40-59	Overweight	0.0062 [-0.0084, 0.0209]	0.80	0.26 [0.01, 0.51]	0.41 [0.22, 0.59]	-0.0000 [-0.0071, 0.0070]	0.50	-0.21 [-0.34, -0.08]	-0.21 [-0.29, -0.13]	0.2 [-0.2, 0.5]	0.80	6.5 [0.3, 12.8]	10.1 [6.3, 13.9]
Taiwan	40-59	Obesity Class I	-0.0338 [-0.0743, 0.0058]	0.95	1.09 [0.32, 1.86]	0.28 [-0.08, 0.63]	0.0020 [-0.0115, 0.0156]	0.62	-0.37 [-0.63, -0.10]	-0.32 [-0.42, -0.22]	0.3 [-0.4, 1.0]	0.79	10.9 [-2.1, 23.5]	17.4 [11.8, 23.0]
Taiwan	60-79	Overweight	0.0056 [-0.0148, 0.0266]	0.71	0.03 [-0.37, 0.43]	0.17 [-0.02, 0.36]	0.0028 [-0.0041, 0.0096]	0.78	-0.15 [-0.28, -0.01]	-0.08 [-0.14, -0.02]	0.2 [-0.1, 0.5]	0.90	1.9 [-4.0, 8.0]	7.1 [3.9, 10.3]
Taiwan	60-79	Obesity Class I	0.0222 [-0.0007, 0.0453]	0.97	-0.45 [-0.84, -0.05]	0.09 [-0.21, 0.38]	-0.0047 [-0.0203, 0.0110]	0.72	-0.03 [-0.33, 0.28]	-0.14 [-0.24, -0.04]	0.6 [0.1, 1.2]	0.99	0.1 [-11.1, 11.3]	15.6 [10.8, 20.3]
Thailand	20-39	Overweight	-0.0042 [-0.0210, 0.0126]	0.69	0.32 [0.19, 0.45]	0.27 [0.14, 0.41]	-0.0094 [-0.0156, -0.0031]	0.998	-0.10 [-0.14, -0.06]	-0.20 [-0.25, -0.16]	-0.0 [-0.1, 0.1]	0.66	5.8 [4.4, 7.2]	5.4 [4.1, 6.6]
Thailand	20-39	Obesity Class I	-0.0026 [-0.0303, 0.0253]	0.57	0.40 [0.18, 0.62]	0.38 [0.18, 0.57]	-0.0197 [-0.0271, -0.0120]	>.999	-0.12 [-0.17, -0.06]	-0.33 [-0.39, -0.28]	-0.1 [-0.2, 0.0]	0.89	10.8 [8.7, 12.9]	8.7 [6.7, 10.6]
Thailand	20-39	Obesity Class II/III	-0.0232 [-0.0617, 0.0152]	0.88	0.54 [0.23, 0.86]	0.29 [0.02, 0.55]	-0.0214 [-0.0309, -0.0116]	>.999	-0.14 [-0.22, -0.06]	-0.37 [-0.44, -0.30]	-0.3 [-0.6, -0.0]	0.99	18.9 [12.6, 25.2]	9.4 [6.8, 12.0]
Thailand	40-59	Overweight	-0.0056 [-0.0158, 0.0046]	0.86	0.34 [0.20, 0.48]	0.22 [0.13, 0.32]	-0.0051 [-0.0084, -0.0018]	>.999	-0.03 [-0.08, 0.01]	-0.14 [-0.17, -0.10]	-0.1 [-0.2, -0.0]	0.996	8.2 [6.5, 9.9]	4.2 [2.5, 5.9]
Thailand	40-59	Obesity Class I	-0.0140 [-0.0300, 0.0018]	0.96	0.47 [0.24, 0.71]	0.19 [0.06, 0.32]	-0.0039 [-0.0079, 0.0001]	0.97	-0.09 [-0.15, -0.03]	-0.17 [-0.21, -0.13]	-0.1 [-0.3, 0.0]	0.94	12.9 [10.0, 15.8]	9.6 [7.5, 11.6]
Thailand	40-59	Obesity Class II/III	-0.0231 [-0.0528, 0.0056]	0.95	0.61 [0.18, 1.06]	0.14 [-0.07, 0.36]	-0.0037 [-0.0104, 0.0030]	0.86	-0.11 [-0.22, -0.01]	-0.19 [-0.24, -0.13]	-0.6 [-1.0, -0.3]	>.999	27.5 [20.9, 34.3]	8.8 [4.9, 12.6]
Thailand	60-79	Overweight	-0.0111 [-0.0261, 0.0037]	0.93	0.22 [0.00, 0.44]	-0.00 [-0.13, 0.12]	-0.0037 [-0.0077, 0.0003]	0.96	0.01 [-0.05, 0.07]	-0.06 [-0.10, -0.03]	-0.0 [-0.2, 0.1]	0.67	4.4 [1.3, 7.5]	3.5 [1.5, 5.4]
Thailand	60-79	Obesity Class I	-0.0139 [-0.0325, 0.0047]	0.93	0.11 [-0.17, 0.39]	-0.17 [-0.33, -0.01]	-0.0070 [-0.0125, -0.0015]	0.993	0.08 [0.00, 0.15]	-0.06 [-0.12, -0.01]	-0.5 [-0.7, -0.2]	>.999	14.9 [9.5, 20.5]	1.7 [-1.0, 4.4]
Thailand	60-79	Obesity Class II/III	-0.0167 [-0.0464, 0.0133]	0.87	0.21 [-0.22, 0.63]	-0.12 [-0.41, 0.15]	-0.0075 [-0.0151, 0.0002]	0.97	0.11 [0.01, 0.22]	-0.04 [-0.13, 0.06]	-0.3 [-0.7, 0.1]	0.94	13.0 [5.3, 20.6]	4.3 [-0.1, 8.8]
USA	20-39	Overweight	-0.0069 [-0.0132, -0.0005]	0.98	0.49 [0.36, 0.62]	0.27 [0.17, 0.37]	0.0018 [-0.0006, 0.0043]	0.93	-0.19 [-0.24, -0.15]	-0.14 [-0.18, -0.09]	-0.1 [-0.1, 0.0]	0.96	2.9 [1.6, 4.2]	0.9 [-0.2, 2.1]
USA	20-39	Obesity Class I	-0.0003 [-0.0072, 0.0065]	0.53	0.57 [0.44, 0.71]	0.56 [0.45, 0.68]	-0.0028 [-0.0057, -0.0000]	0.98	-0.24 [-0.29, -0.18]	-0.33 [-0.37, -0.28]	-0.0 [-0.1, 0.1]	0.68	5.1 [3.4, 6.9]	4.5 [2.9, 6.0]
USA	20-39	Obesity Class II/III	-0.0008 [-0.0076, 0.0059]	0.59	0.61 [0.48, 0.74]	0.59 [0.47, 0.70]	-0.0016 [-0.0039, 0.0008]	0.90	-0.35 [-0.40, -0.31]	-0.40 [-0.44, -0.36]	-0.0 [-0.1, 0.1]	0.75	8.6 [6.6, 10.7]	7.5 [6.0, 9.0]
USA	40-59	Overweight	-0.0012 [-0.0090, 0.0064]	0.62	0.39 [0.25, 0.54]	0.35 [0.22, 0.49]	-0.0018 [-0.0049, 0.0013]	0.87	-0.16 [-0.21, -0.10]	-0.21 [-0.27, -0.16]	-0.1 [-0.2, 0.0]	0.96	4.7 [2.4, 7.0]	1.4 [-0.4, 3.3]
USA	40-59	Obesity Class I	-0.0055 [-0.0132, 0.0022]	0.91	0.52 [0.37, 0.67]	0.35 [0.22, 0.48]	-0.0003 [-0.0034, 0.0028]	0.58	-0.32 [-0.38, -0.26]	-0.33 [-0.39, -0.27]	-0.2 [-0.3, -0.1]	>.999	10.0 [7.5, 12.5]	3.5 [1.5, 5.5]
USA	40-59	Obesity Class II/III	-0.0101 [-0.0184, -0.0019]	0.993	0.57 [0.40, 0.73]	0.25 [0.12, 0.38]	-0.0028 [-0.0057, 0.0000]	0.97	-0.35 [-0.41, -0.30]	-0.44 [-0.49, -0.39]	-0.2 [-0.3, -0.0]	0.997	13.3 [10.8, 15.8]	7.9 [5.8, 9.9]
USA	60-79	Overweight	-0.0030 [-0.0111, 0.0053]	0.76	0.28 [0.13, 0.43]	0.19 [0.05, 0.33]	-0.0020 [-0.0052, 0.0011]	0.89	-0.14 [-0.20, -0.08]	-0.20 [-0.26, -0.14]	0.0 [-0.2, 0.2]	0.56	2.0 [-1.2, 5.3]	2.4 [-0.4, 5.2]
USA	60-79	Obesity Class I	-0.0100 [-0.0193, -0.0005]	0.98	0.37 [0.19, 0.55]	0.07 [-0.10, 0.22]	-0.0026 [-0.0059, 0.0007]	0.94	-0.23 [-0.30, -0.16]	-0.31 [-0.36, -0.25]	-0.2 [-0.4, -0.1]	0.999	6.4 [3.3, 9.6]	-1.0 [-3.6, 1.6]
USA	60-79	Obesity Class II/III	-0.0188 [-0.0291, -0.0089]	>.999	0.39 [0.18, 0.59]	-0.20 [-0.36, -0.04]	-0.0006 [-0.0039, 0.0027]	0.62	-0.32 [-0.39, -0.25]	-0.34 [-0.39, -0.28]	-0.1 [-0.3, 0.1]	0.82	4.2 [0.6, 7.8]	1.7 [-1.0, 4.5]
**Males**														
England	20-39	Overweight	-0.0073 [-0.0199, 0.0053]	0.87	0.60 [0.45, 0.74]	0.43 [0.27, 0.59]	-0.0027 [-0.0061, 0.0008]	0.94	-0.10 [-0.14, -0.06]	-0.16 [-0.21, -0.12]	-0.0 [-0.1, 0.0]	0.83	4.1 [3.4, 4.7]	3.2 [2.1, 4.4]
England	20-39	Obesity Class I	0.0018 [-0.0166, 0.0195]	0.57	0.92 [0.72, 1.14]	0.96 [0.73, 1.20]	-0.0017 [-0.0069, 0.0034]	0.75	-0.22 [-0.28, -0.15]	-0.26 [-0.32, -0.19]	-0.0 [-0.1, 0.0]	0.76	7.2 [6.2, 8.2]	6.4 [5.1, 7.8]
England	20-39	Obesity Class II/III	-0.0068 [-0.0329, 0.0193]	0.69	0.99 [0.67, 1.31]	0.83 [0.49, 1.17]	-0.0011 [-0.0060, 0.0037]	0.67	-0.30 [-0.36, -0.23]	-0.32 [-0.38, -0.26]	-0.2 [-0.3, -0.1]	>.999	11.6 [9.9, 13.3]	6.1 [4.1, 8.0]
England	40-59	Overweight	-0.0026 [-0.0129, 0.0079]	0.69	0.40 [0.28, 0.53]	0.34 [0.21, 0.48]	0.0016 [-0.0022, 0.0053]	0.79	-0.16 [-0.21, -0.11]	-0.12 [-0.18, -0.07]	-0.0 [-0.1, 0.0]	0.85	4.1 [3.4, 4.9]	3.2 [2.0, 4.4]
England	40-59	Obesity Class I	-0.0088 [-0.0214, 0.0036]	0.92	0.66 [0.50, 0.81]	0.45 [0.29, 0.61]	-0.0013 [-0.0053, 0.0027]	0.74	-0.24 [-0.29, -0.20]	-0.27 [-0.33, -0.22]	-0.1 [-0.2, -0.0]	0.99	9.1 [8.0, 10.2]	6.4 [5.0, 7.9]
England	40-59	Obesity Class II/III	-0.0086 [-0.0261, 0.0089]	0.83	0.51 [0.29, 0.72]	0.31 [0.09, 0.54]	-0.0047 [-0.0092, -0.0001]	0.98	-0.27 [-0.33, -0.21]	-0.38 [-0.44, -0.32]	-0.2 [-0.3, -0.1]	>.999	13.0 [11.2, 14.8]	7.2 [5.2, 9.2]
England	60-79	Overweight	-0.0070 [-0.0162, 0.0018]	0.94	0.23 [0.10, 0.35]	0.06 [-0.05, 0.18]	-0.0025 [-0.0060, 0.0011]	0.91	-0.13 [-0.18, -0.09]	-0.19 [-0.24, -0.14]	-0.1 [-0.2, 0.0]	0.96	3.8 [2.6, 5.0]	1.7 [0.3, 3.2]
England	60-79	Obesity Class I	-0.0130 [-0.0240, -0.0019]	0.99	0.29 [0.15, 0.44]	-0.00 [-0.14, 0.13]	-0.0047 [-0.0086, -0.0009]	0.993	-0.22 [-0.27, -0.17]	-0.33 [-0.38, -0.28]	-0.1 [-0.2, -0.0]	0.98	6.1 [4.6, 7.6]	3.0 [1.2, 4.7]
England	60-79	Obesity Class II/III	-0.0048 [-0.0211, 0.0118]	0.72	0.06 [-0.18, 0.30]	-0.05 [-0.23, 0.13]	-0.0036 [-0.0084, 0.0011]	0.93	-0.32 [-0.39, -0.26]	-0.41 [-0.46, -0.35]	-0.2 [-0.4, -0.0]	0.993	8.3 [5.3, 11.3]	2.4 [0.0, 4.7]
Finland	20-39	Overweight	-0.0025 [-0.0099, 0.0049]	0.75	0.53 [0.42, 0.64]	0.45 [0.30, 0.60]	0.0007 [-0.0017, 0.0033]	0.73	-0.15 [-0.19, -0.12]	-0.13 [-0.19, -0.08]	-0.0 [-0.1, 0.1]	0.54	3.6 [2.2, 5.0]	3.5 [1.2, 5.8]
Finland	20-39	Obesity Class I	-0.0116 [-0.0233, 0.0000]	0.97	0.91 [0.74, 1.09]	0.55 [0.31, 0.79]	0.0024 [-0.0005, 0.0054]	0.95	-0.29 [-0.34, -0.25]	-0.22 [-0.28, -0.15]	0.0 [-0.1, 0.2]	0.65	7.8 [5.8, 9.9]	8.6 [5.8, 11.6]
Finland	40-59	Overweight	-0.0033 [-0.0087, 0.0022]	0.88	0.38 [0.30, 0.46]	0.28 [0.16, 0.39]	0.0001 [-0.0016, 0.0018]	0.55	-0.16 [-0.18, -0.13]	-0.15 [-0.19, -0.12]	0.0 [-0.0, 0.1]	0.88	3.6 [2.3, 4.9]	5.1 [3.4, 6.7]
Finland	40-59	Obesity Class I	-0.0116 [-0.0191, -0.0044]	>.999	0.62 [0.51, 0.73]	0.26 [0.10, 0.41]	-0.0005 [-0.0025, 0.0014]	0.70	-0.25 [-0.29, -0.22]	-0.27 [-0.31, -0.23]	-0.1 [-0.2, 0.0]	0.86	8.8 [7.1, 10.5]	7.0 [4.9, 9.1]
Finland	40-59	Obesity Class II/III	-0.0181 [-0.0301, -0.0061]	0.999	0.62 [0.41, 0.83]	0.06 [-0.16, 0.27]	-0.0021 [-0.0047, 0.0005]	0.94	-0.30 [-0.34, -0.26]	-0.36 [-0.41, -0.31]	-0.1 [-0.3, 0.0]	0.91	14.5 [11.6, 17.5]	11.0 [8.0, 14.1]
Japan	20-39	Overweight	-0.0040 [-0.0096, 0.0017]	0.91	0.66 [0.58, 0.74]	0.54 [0.41, 0.66]	0.0008 [-0.0014, 0.0030]	0.76	-0.21 [-0.23, -0.18]	-0.18 [-0.23, -0.13]	0.0 [-0.1, 0.1]	0.73	5.4 [4.4, 6.4]	6.2 [4.5, 7.9]
Japan	20-39	Obesity Class I	-0.0103 [-0.0223, 0.0017]	0.95	1.09 [0.90, 1.28]	0.76 [0.52, 1.00]	-0.0012 [-0.0046, 0.0021]	0.77	-0.28 [-0.33, -0.22]	-0.32 [-0.38, -0.25]	0.0 [-0.1, 0.2]	0.71	11.2 [8.9, 13.5]	12.6 [9.2, 15.9]

Country	Age group	BMI range	Non-HDL cholesterol				HDL cholesterol				Systolic blood pressure			
			Slope and 95% CI	Probability (P)*	Predicted value for difference at earliest year and 95% CI	Predicted value for difference at latest year and 95% CI	Slope and 95% CI	Probability (P)*	Predicted value for difference at earliest year and 95% CI	Predicted value for difference at latest year and 95% CI	Slope and 95% CI	Probability (P)*	Predicted value for difference at earliest year and 95% CI	Predicted value for difference at latest year and 95% CI
Japan	40-59	Overweight	-0.0050 [-0.0089, -0.0012]	0.996	0.44 [0.39, 0.50]	0.28 [0.20, 0.37]	-0.0024 [-0.0038, -0.0010]	>.999	-0.17 [-0.19, -0.15]	-0.24 [-0.28, -0.21]	0.1 [-0.0, 0.1]	0.97	4.6 [3.7, 5.6]	6.5 [5.2, 7.9]
Japan	40-59	Obesity Class I	-0.0136 [-0.0219, -0.0049]	0.999	0.68 [0.53, 0.82]	0.24 [0.08, 0.41]	-0.0023 [-0.0047, 0.0001]	0.97	-0.25 [-0.29, -0.20]	-0.32 [-0.37, -0.27]	0.0 [-0.1, 0.2]	0.69	10.0 [7.5, 12.4]	11.1 [8.4, 13.9]
Japan	60-79	Overweight	-0.0076 [-0.0109, -0.0042]	>.999	0.32 [0.26, 0.37]	0.07 [0.01, 0.14]	-0.0003 [-0.0017, 0.0011]	0.65	-0.15 [-0.17, -0.13]	-0.16 [-0.18, -0.13]	-0.0 [-0.1, 0.0]	0.93	3.9 [2.9, 5.0]	2.5 [1.4, 3.6]
Japan	60-79	Obesity Class I	-0.0124 [-0.0224, -0.0023]	0.992	0.39 [0.19, 0.59]	-0.01 [-0.16, 0.15]	-0.0041 [-0.0074, -0.0008]	0.993	-0.13 [-0.19, -0.06]	-0.26 [-0.31, -0.21]	-0.3 [-0.5, -0.2]	>.999	12.0 [8.6, 15.5]	1.4 [-1.4, 4.1]
South Korea	20-39	Overweight	-0.0028 [-0.0078, 0.0021]	0.86	0.47 [0.39, 0.55]	0.40 [0.33, 0.47]	-0.0026 [-0.0041, -0.0011]	>.999	-0.08 [-0.10, -0.05]	-0.14 [-0.17, -0.12]	-0.0 [-0.1, 0.1]	0.51	5.0 [4.0, 6.0]	5.0 [4.2, 5.8]
South Korea	20-39	Obesity Class I	-0.0119 [-0.0221, -0.0017]	0.99	0.87 [0.69, 1.06]	0.56 [0.45, 0.68]	-0.0058 [-0.0083, -0.0033]	>.999	-0.11 [-0.15, -0.07]	-0.26 [-0.29, -0.23]	-0.0 [-0.2, 0.1]	0.73	10.1 [7.6, 12.5]	9.0 [7.6, 10.4]
South Korea	40-59	Overweight	-0.0086 [-0.0133, -0.0038]	>.999	0.36 [0.28, 0.44]	0.14 [0.07, 0.20]	-0.0019 [-0.0034, -0.0005]	0.996	-0.08 [-0.10, -0.05]	-0.13 [-0.15, -0.11]	0.0 [-0.1, 0.1]	0.61	4.5 [3.1, 5.8]	4.7 [3.9, 5.6]
South Korea	40-59	Obesity Class I	-0.0191 [-0.0293, -0.0091]	>.999	0.57 [0.39, 0.75]	0.07 [-0.06, 0.19]	-0.0006 [-0.0033, 0.0022]	0.66	-0.16 [-0.21, -0.11]	-0.18 [-0.21, -0.15]	-0.2 [-0.4, 0.0]	0.97	10.9 [6.9, 14.9]	5.6 [3.6, 7.5]
South Korea	60-79	Overweight	-0.0126 [-0.0195, -0.0060]	>.999	0.29 [0.17, 0.41]	-0.03 [-0.11, 0.04]	0.0002 [-0.0017, 0.0021]	0.59	-0.09 [-0.12, -0.05]	-0.08 [-0.10, -0.06]	-0.0 [-0.2, 0.1]	0.68	3.6 [1.2, 6.1]	2.8 [1.5, 4.1]
South Korea	60-79	Obesity Class I	-0.0272 [-0.0419, -0.0123]	>.999	0.36 [0.12, 0.60]	-0.26 [-0.43, -0.09]	-0.0022 [-0.0064, 0.0018]	0.87	-0.08 [-0.15, -0.02]	-0.13 [-0.18, -0.09]	-0.2 [-0.6, 0.1]	0.92	6.3 [0.6, 12.1]	0.9 [-1.9, 3.9]
Taiwan	20-39	Overweight	0.0095 [-0.0069, 0.0257]	0.87	0.18 [-0.09, 0.46]	0.41 [0.19, 0.63]	-0.0010 [-0.0100, 0.0083]	0.58	-0.13 [-0.30, 0.04]	-0.15 [-0.24, -0.07]	0.2 [-0.1, 0.6]	0.88	3.7 [-3.1, 10.2]	8.6 [5.4, 11.9]
Taiwan	40-59	Overweight	-0.0014 [-0.0169, 0.0149]	0.57	0.27 [-0.02, 0.54]	0.23 [0.04, 0.43]	0.0000 [-0.0053, 0.0051]	0.50	-0.15 [-0.24, -0.07]	-0.15 [-0.22, -0.09]	-0.0 [-0.3, 0.3]	0.53	7.4 [2.5, 12.3]	7.1 [4.1, 10.2]
Taiwan	40-59	Obesity Class I	-0.0024 [-0.0254, 0.0213]	0.58	0.26 [-0.19, 0.71]	0.20 [-0.08, 0.49]	0.0010 [-0.0078, 0.0096]	0.59	-0.22 [-0.38, -0.06]	-0.19 [-0.27, -0.11]	0.3 [-0.5, 1.2]	0.78	7.1 [-9.3, 23.5]	15.1 [9.4, 20.7]
Taiwan	60-79	Overweight	-0.0117 [-0.0240, 0.0005]	0.97	0.22 [-0.01, 0.45]	-0.06 [-0.21, 0.09]	-0.0022 [-0.0081, 0.0037]	0.77	-0.09 [-0.21, 0.02]	-0.15 [-0.20, -0.09]	0.1 [-0.3, 0.5]	0.66	4.3 [-3.2, 12.0]	6.2 [2.9, 9.6]
Taiwan	60-79	Obesity Class I	-0.0114 [-0.0468, 0.0250]	0.73	0.26 [-0.44, 0.94]	-0.01 [-0.34, 0.31]	0.0032 [-0.0100, 0.0159]	0.68	-0.25 [-0.50, 0.01]	-0.17 [-0.26, -0.09]	0.0 [-0.9, 1.0]	0.54	12.5 [-5.6, 31.0]	13.6 [7.4, 19.6]
Thailand	20-39	Overweight	-0.0358 [-0.0569, -0.0146]	>.999	0.69 [0.54, 0.83]	0.29 [0.13, 0.45]	0.0019 [-0.0049, 0.0086]	0.71	-0.15 [-0.20, -0.10]	-0.13 [-0.18, -0.07]	-0.1 [-0.3, -0.0]	0.98	7.8 [5.1, 10.5]	4.0 [2.4, 5.6]
Thailand	20-39	Obesity Class I	-0.0026 [-0.0447, 0.0402]	0.55	0.75 [0.50, 0.99]	0.71 [0.37, 1.07]	-0.0081 [-0.0150, -0.0012]	0.99	-0.18 [-0.24, -0.13]	-0.27 [-0.33, -0.22]	-0.2 [-0.5, 0.1]	0.84	10.9 [4.4, 17.2]	6.4 [3.3, 9.6]
Thailand	20-39	Obesity Class II/III	-0.0209 [-0.0531, 0.0109]	0.89	0.92 [0.64, 1.20]	0.69 [0.43, 0.95]	0.0007 [-0.0086, 0.0099]	0.56	-0.27 [-0.35, -0.19]	-0.26 [-0.33, -0.20]	0.1 [-0.3, 0.5]	0.70	8.5 [1.5, 15.4]	11.0 [7.0, 15.0]
Thailand	40-59	Overweight	-0.0075 [-0.0215, 0.0070]	0.85	0.47 [0.29, 0.66]	0.32 [0.17, 0.48]	-0.0015 [-0.0048, 0.0018]	0.81	-0.08 [-0.12, -0.04]	-0.11 [-0.15, -0.07]	-0.1 [-0.2, 0.1]	0.86	7.7 [5.5, 9.9]	5.7 [3.6, 7.9]
Thailand	40-59	Obesity Class I	-0.0248 [-0.0496, 0.0003]	0.97	0.85 [0.50, 1.20]	0.35 [0.13, 0.58]	-0.0046 [-0.0095, 0.0002]	0.97	-0.10 [-0.17, -0.04]	-0.20 [-0.25, -0.15]	-0.3 [-0.5, -0.1]	>.999	16.7 [13.3, 20.0]	7.7 [4.6, 10.7]
Thailand	40-59	Obesity Class II/III	-0.0224 [-0.0455, 0.0003]	0.97	0.62 [0.36, 0.89]	0.18 [-0.13, 0.48]	-0.0025 [-0.0124, 0.0074]	0.69	-0.09 [-0.22, 0.05]	-0.14 [-0.25, -0.03]	0.5 [0.1, 1.0]	0.991	3.7 [-5.0, 12.6]	19.7 [13.9, 25.4]
Thailand	60-79	Overweight	-0.0189 [-0.0356, -0.0021]	0.99	0.40 [0.15, 0.65]	0.02 [-0.11, 0.14]	-0.0040 [-0.0080, 0.0000]	0.97	-0.02 [-0.08, 0.04]	-0.10 [-0.14, -0.06]	-0.2 [-0.4, -0.0]	0.98	8.1 [4.0, 12.1]	1.9 [-0.4, 4.2]
Thailand	60-79	Obesity Class I	-0.0550 [-0.0894, -0.0209]	>.999	0.92 [0.41, 1.45]	-0.17 [-0.42, 0.07]	-0.0050 [-0.0149, 0.0052]	0.83	0.01 [-0.14, 0.16]	-0.09 [-0.16, -0.02]	-0.4 [-0.9, 0.0]	0.97	14.7 [4.7, 25.0]	1.7 [-2.7, 6.3]
USA	20-39	Overweight	-0.0035 [-0.0103, 0.0035]	0.84	0.54 [0.41, 0.66]	0.43 [0.31, 0.55]	0.0003 [-0.0020, 0.0026]	0.60	-0.16 [-0.20, -0.12]	-0.15 [-0.18, -0.11]	-0.0 [-0.1, 0.0]	0.89	3.4 [2.1, 4.8]	2.0 [0.8, 3.2]
USA	20-39	Obesity Class I	-0.0016 [-0.0095, 0.0063]	0.65	0.75 [0.59, 0.90]	0.69 [0.57, 0.82]	0.0004 [-0.0025, 0.0032]	0.61	-0.26 [-0.31, -0.21]	-0.25 [-0.30, -0.20]	-0.0 [-0.1, 0.1]	0.76	6.0 [4.3, 7.7]	5.0 [3.6, 6.4]
USA	20-39	Obesity Class II/III	-0.0000 [-0.0100, 0.0100]	0.50	0.71 [0.51, 0.91]	0.71 [0.56, 0.85]	-0.0003 [-0.0030, 0.0023]	0.59	-0.32 [-0.37, -0.26]	-0.33 [-0.37, -0.29]	-0.1 [-0.2, 0.0]	0.95	12.4 [9.7, 15.0]	9.2 [7.5, 11.0]
USA	40-59	Overweight	0.0046 [-0.0045, 0.0136]	0.84	0.29 [0.13, 0.46]	0.44 [0.28, 0.59]	-0.0008 [-0.0037, 0.0021]	0.71	-0.17 [-0.22, -0.12]	-0.20 [-0.25, -0.15]	-0.0 [-0.1, 0.1]	0.69	2.7 [0.7, 4.8]	1.9 [0.0, 3.8]
USA	40-59	Obesity Class I	-0.0017 [-0.0116, 0.0083]	0.63	0.52 [0.33, 0.71]	0.47 [0.31, 0.63]	0.0037 [0.0006, 0.0067]	0.99	-0.37 [-0.43, -0.31]	-0.25 [-0.31, -0.20]	-0.0 [-0.2, 0.1]	0.77	5.4 [3.0, 7.8]	3.9 [1.8, 6.0]
USA	40-59	Obesity Class II/III	-0.0087 [-0.0194, 0.0023]	0.94	0.39 [0.17, 0.61]	0.12 [-0.05, 0.29]	0.0012 [-0.0018, 0.0042]	0.78	-0.38 [-0.44, -0.32]	-0.34 [-0.39, -0.29]	-0.1 [-0.2, 0.1]	0.84	9.1 [6.3, 11.9]	6.9 [4.6, 9.2]
USA	60-79	Overweight	-0.0037 [-0.0117, 0.0045]	0.81	0.17 [0.02, 0.32]	0.06 [-0.08, 0.20]	-0.0014 [-0.0049, 0.0022]	0.77	-0.13 [-0.20, -0.06]	-0.17 [-0.23, -0.11]	-0.2 [-0.4, -0.1]	>.999	4.2 [1.4, 7.1]	-2.4 [-4.9, -0.0]
USA	60-79	Obesity Class I	0.0044 [-0.0045, 0.0132]	0.83	0.00 [-0.17, 0.16]	0.14 [-0.02, 0.29]	-0.0049 [-0.0085, -0.0012]	0.996	-0.16 [-0.24, -0.09]	-0.31 [-0.37, -0.25]	-0.2 [-0.4, -0.1]	0.996	4.9 [1.7, 8.2]	-1.9 [-4.6, 0.8]
USA	60-79	Obesity Class II/III	-0.0083 [-0.0203, 0.0031]	0.93	0.20 [-0.03, 0.43]	-0.06 [-0.24, 0.12]	-0.0069 [-0.0114, -0.0024]	>.999	-0.16 [-0.26, -0.07]	-0.38 [-0.45, -0.31]	-0.3 [-0.5, -0.1]	0.997	6.1 [2.4, 9.9]	-2.1 [-5.3, 1.0]

* Probability of the slope being a true negative or a true positive

Appendix Table 6. Regression coefficients and associated 95% confidence intervals and *P* for linear models for the difference in treatment between obesity and overweight and normal BMI.

The confidence intervals and probabilities *P* were obtained using the simulation with 10,000 draws as described in Methods.

Country	Age group	BMI range	Lipid-lowering medicine use				Antihypertensive medicine use			
			Slope and 95% CI	Probability (*P*)*	Predicted value for difference at earliest year and 95% CI	Predicted value for difference at latest year and 95% CI	Slope and 95% CI	Probability (*P*)*	Predicted value for difference at earliest year and 95% CI	Predicted value for difference at latest year and 95% CI
**Females**										
England	20-39	Overweight	-0.00 [-0.19, 0.25]	0.51	0 [-2, 2]	0 [-2, 4]	-0.01 [-0.10, 0.10]	0.58	0 [-1, 1]	0 [-2, 2]
England	20-39	Obesity Class I	0.06 [-0.17, 0.46]	0.69	1 [-3, 4]	2 [-1, 8]	-0.04 [-0.16, 0.16]	0.70	2 [-0, 4]	1 [-1, 5]
England	20-39	Obesity Class II/III	0.08 [-0.20, 0.50]	0.71	2 [-2, 6]	4 [1, 10]	0.00 [-0.15, 0.17]	0.51	3 [1, 5]	3 [1, 6]
England	40-59	Overweight	0.11 [-0.02, 0.24]	0.96	1 [-0, 3]	4 [2, 6]	0.04 [-0.06, 0.15]	0.80	3 [2, 5]	4 [3, 6]
England	40-59	Obesity Class I	0.17 [-0.01, 0.41]	0.97	4 [1, 6]	8 [5, 11]	-0.03 [-0.17, 0.13]	0.64	9 [7, 11]	8 [5, 11]
England	40-59	Obesity Class II/III	0.31 [-0.04, 0.73]	0.96	7 [2, 12]	15 [10, 20]	0.19 [-0.01, 0.40]	0.97	15 [12, 18]	21 [17, 24]
England	60-79	Overweight	0.57 [0.21, 0.92]	>.999	1 [-4, 5]	14 [9, 19]	0.38 [0.18, 0.60]	>.999	2 [-2, 5]	13 [10, 16]
England	60-79	Obesity Class I	0.28 [-0.13, 0.68]	0.91	10 [5, 15]	16 [11, 22]	0.45 [0.20, 0.71]	>.999	11 [6, 15]	24 [20, 28]
England	60-79	Obesity Class II/III	0.71 [0.19, 1.25]	0.996	11 [4, 18]	28 [21, 35]	0.33 [-0.02, 0.67]	0.97	24 [18, 30]	34 [28, 39]
Finland	20-39	Overweight	0.01 [-0.04, 0.09]	0.69	0 [-1, 1]	0 [-0, 2]	0.03 [-0.07, 0.16]	0.72	1 [-1, 3]	2 [-0, 5]
Finland	20-39	Obesity Class I	0.01 [-0.13, 0.15]	0.55	1 [-1, 3]	1 [-1, 4]	0.06 [-0.13, 0.31]	0.70	2 [-1, 6]	4 [1, 10]
Finland	20-39	Obesity Class II/III	0.01 [-0.30, 0.25]	0.56	2 [-0, 8]	2 [-1, 7]	0.12 [-0.18, 0.44]	0.79	4 [-0, 10]	8 [3, 15]
Finland	40-59	Overweight	0.04 [-0.06, 0.15]	0.81	1 [0, 2]	3 [1, 4]	0.24 [0.09, 0.39]	0.999	5 [3, 7]	12 [9, 15]
Finland	40-59	Obesity Class I	0.16 [0.01, 0.31]	0.98	1 [-0, 3]	5 [2, 8]	0.07 [-0.13, 0.28]	0.77	14 [11, 17]	16 [12, 21]
Finland	40-59	Obesity Class II/III	0.32 [0.10, 0.57]	0.998	1 [-1, 4]	9 [5, 13]	0.20 [-0.13, 0.54]	0.88	26 [20, 31]	32 [26, 39]
Japan	20-39	Overweight	0.12 [-0.28, 0.69]	0.70	2 [-1, 6]	5 [-0, 12]	0.09 [-0.02, 0.28]	0.93	1 [-1, 2]	4 [1, 8]
Japan	20-39	Obesity Class I					0.27 [-0.07, 0.78]	0.93	4 [-2, 9]	13 [5, 24]
Japan	40-59	Overweight	-0.01 [-0.26, 0.26]	0.54	3 [1, 6]	3 [-0, 7]	0.01 [-0.11, 0.13]	0.56	7 [6, 9]	8 [5, 10]
Japan	40-59	Obesity Class I	0.34 [-0.26, 0.98]	0.86	7 [1, 13]	14 [6, 22]	0.01 [-0.26, 0.28]	0.52	16 [12, 20]	16 [11, 22]
Japan	60-79	Overweight	0.03 [-0.33, 0.38]	0.55	7 [4, 10]	7 [3, 12]	0.24 [0.08, 0.39]	0.999	11 [9, 13]	18 [15, 21]
Japan	60-79	Obesity Class I	0.31 [-0.51, 1.11]	0.77	9 [2, 16]	15 [5, 25]	0.06 [-0.25, 0.36]	0.65	27 [22, 32]	29 [23, 35]
South Korea	20-39	Overweight	0.05 [-0.07, 0.15]	0.81	1 [-0, 2]	1 [0, 3]	0.02 [-0.06, 0.10]	0.70	1 [-0, 2]	1 [0, 3]
South Korea	20-39	Obesity Class I	-0.09 [-0.56, 0.27]	0.68	6 [2, 12]	4 [1, 8]	-0.08 [-0.45, 0.21]	0.70	7 [1, 14]	4 [1, 8]
South Korea	40-59	Overweight	0.30 [0.12, 0.48]	>.999	1 [-0, 3]	7 [5, 9]	0.21 [0.05, 0.37]	0.996	5 [3, 8]	10 [8, 13]
South Korea	40-59	Obesity Class I	0.81 [0.37, 1.23]	>.999	1 [-3, 6]	16 [12, 22]	0.45 [0.03, 0.85]	0.98	12 [5, 19]	23 [18, 28]
South Korea	60-79	Overweight	0.36 [0.11, 0.61]	0.997	2 [-1, 4]	9 [6, 12]	0.08 [-0.16, 0.33]	0.74	15 [11, 20]	18 [15, 20]
South Korea	60-79	Obesity Class I	0.35 [-0.19, 0.87]	0.90	4 [-1, 10]	11 [5, 17]	0.37 [-0.12, 0.85]	0.93	26 [17, 34]	35 [29, 41]
Taiwan	20-39	Overweight	-0.01 [-0.41, 0.18]	0.53	1 [-1, 10]	1 [-1, 5]	-0.02 [-0.38, 0.13]	0.61	2 [-1, 10]	2 [-1, 5]
Taiwan	40-59	Overweight	0.02 [-0.21, 0.27]	0.57	-1 [-4, 2]	-0 [-4, 4]	0.22 [-0.19, 0.64]	0.86	6 [1, 12]	12 [5, 18]
Taiwan	40-59	Obesity Class I	0.53 [0.03, 1.11]	0.98	-4 [-8, 0]	9 [-0, 19]	0.87 [0.29, 1.50]	0.999	1 [-6, 9]	22 [10, 36]
Taiwan	60-79	Overweight	0.12 [-0.31, 0.55]	0.71	2 [-5, 9]	5 [-1, 11]	0.34 [-0.36, 1.03]	0.83	10 [-1, 23]	19 [9, 28]
Taiwan	60-79	Obesity Class I	0.05 [-0.85, 0.84]	0.55	8 [-5, 24]	9 [-2, 20]	0.59 [-0.53, 1.58]	0.85	13 [-5, 35]	27 [12, 42]
Thailand	20-39	Overweight	-0.17 [-0.36, 0.00]	0.97	1 [0, 3]	-1 [-2, 1]	0.01 [-0.05, 0.07]	0.65	1 [-0, 2]	1 [0, 2]
Thailand	20-39	Obesity Class I	-0.13 [-0.38, 0.04]	0.92	2 [-0, 5]	0 [-2, 3]	-0.01 [-0.16, 0.11]	0.55	3 [0, 6]	2 [0, 4]
Thailand	20-39	Obesity Class II/III	0.18 [-0.47, 0.82]	0.73	2 [-1, 7]	4 [-0, 10]	0.02 [-0.55, 0.33]	0.54	4 [-2, 17]	4 [-1, 10]
Thailand	40-59	Overweight	0.17 [-0.01, 0.35]	0.97	0 [-1, 2]	4 [1, 7]	-0.02 [-0.13, 0.10]	0.61	5 [3, 8]	5 [3, 7]
Thailand	40-59	Obesity Class I	0.37 [0.17, 0.58]	>.999	1 [-1, 3]	9 [5, 12]	0.12 [-0.11, 0.34]	0.86	8 [4, 13]	12 [9, 15]
Thailand	40-59	Obesity Class II/III	0.88 [0.45, 1.30]	>.999	-2 [-5, 3]	16 [10, 23]	0.03 [-0.47, 0.47]	0.55	19 [10, 29]	19 [13, 26]
Thailand	60-79	Overweight	0.27 [-0.06, 0.58]	0.95	2 [-1, 6]	8 [4, 12]	0.08 [-0.20, 0.35]	0.70	6 [0, 11]	8 [4, 12]
Thailand	60-79	Obesity Class I	0.52 [-0.02, 0.98]	0.97	2 [-2, 10]	13 [6, 20]	0.08 [-0.51, 0.64]	0.61	16 [5, 27]	18 [11, 25]
Thailand	60-79	Obesity Class II/III	-0.30 [-1.63, 0.78]	0.70	9 [-3, 28]	4 [-9, 16]	-0.53 [-1.29, 0.29]	0.90	31 [13, 47]	15 [6, 25]

Country	Age group	BMI range	Lipid-lowering medicine use				Antihypertensive medicine use			
			Slope and 95% CI	Probability (*P*)*	Predicted value for difference at earliest year and 95% CI	Predicted value for difference at latest year and 95% CI	Slope and 95% CI	Probability (*P*)*	Predicted value for difference at earliest year and 95% CI	Predicted value for difference at latest year and 95% CI
USA	20-39	Overweight	0.03 [-0.04, 0.09]	0.79	-0 [-2, 1]	0 [-1, 2]	0.07 [-0.02, 0.16]	0.94	-0 [-1, 1]	2 [0, 4]
USA	20-39	Obesity Class I	0.05 [-0.03, 0.12]	0.89	-0 [-2, 1]	1 [0, 3]	0.09 [-0.03, 0.23]	0.93	1 [-1, 3]	4 [2, 7]
USA	20-39	Obesity Class II/III	0.05 [-0.01, 0.12]	0.94	0 [-1, 2]	2 [1, 3]	-0.11 [-0.27, 0.03]	0.93	9 [5, 12]	5 [3, 7]
USA	40-59	Overweight	0.05 [-0.13, 0.23]	0.71	3 [-0, 6]	4 [1, 8]	-0.13 [-0.38, 0.11]	0.86	10 [5, 14]	5 [1, 10]
USA	40-59	Obesity Class I	0.25 [0.01, 0.49]	0.98	3 [-1, 7]	11 [7, 15]	-0.13 [-0.41, 0.15]	0.82	18 [13, 24]	14 [9, 19]
USA	40-59	Obesity Class II/III	0.37 [0.15, 0.58]	>.999	3 [-0, 7]	15 [11, 19]	-0.13 [-0.44, 0.19]	0.78	32 [26, 39]	28 [23, 34]
USA	60-79	Overweight	0.07 [-0.26, 0.40]	0.66	3 [-3, 9]	5 [-1, 11]	0.10 [-0.24, 0.45]	0.72	10 [4, 16]	13 [7, 20]
USA	60-79	Obesity Class I	0.40 [0.05, 0.76]	0.99	-0 [-7, 6]	12 [5, 19]	0.11 [-0.28, 0.50]	0.71	20 [12, 27]	23 [16, 30]
USA	60-79	Obesity Class II/III	0.52 [0.13, 0.90]	0.995	-0 [-7, 7]	16 [9, 23]	0.03 [-0.36, 0.43]	0.57	32 [25, 39]	33 [26, 40]
**Males**										
England	20-39	Overweight	-0.02 [-0.26, 0.32]	0.57	1 [-3, 3]	0 [-3, 5]	0.01 [-0.08, 0.16]	0.64	0 [-1, 1]	1 [-1, 3]
England	20-39	Obesity Class I	0.01 [-0.28, 0.35]	0.53	2 [-2, 5]	2 [-2, 6]	0.02 [-0.10, 0.17]	0.62	2 [-0, 3]	2 [0, 5]
England	20-39	Obesity Class II/III	-0.02 [-0.52, 0.68]	0.52	5 [-3, 12]	5 [-1, 14]	0.31 [-0.06, 0.78]	0.94	1 [-4, 6]	10 [4, 19]
England	40-59	Overweight	0.06 [-0.25, 0.36]	0.65	2 [-2, 5]	3 [-1, 7]	-0.04 [-0.16, 0.07]	0.78	3 [1, 5]	2 [-1, 4]
England	40-59	Obesity Class I	-0.17 [-0.49, 0.15]	0.85	9 [5, 13]	5 [1, 10]	0.15 [-0.02, 0.31]	0.96	7 [5, 9]	11 [8, 14]
England	40-59	Obesity Class II/III	0.28 [-0.27, 0.85]	0.84	12 [5, 19]	18 [11, 26]	0.45 [0.16, 0.76]	0.998	14 [9, 19]	27 [22, 32]
England	60-79	Overweight	0.18 [-0.22, 0.56]	0.80	7 [3, 12]	11 [6, 17]	0.29 [0.02, 0.59]	0.98	5 [-0, 11]	14 [10, 18]
England	60-79	Obesity Class I	0.59 [0.13, 1.06]	0.994	9 [3, 15]	23 [16, 29]	0.60 [0.28, 0.91]	>.999	9 [3, 15]	27 [22, 31]
England	60-79	Obesity Class II/III	0.50 [-0.24, 1.22]	0.91	18 [8, 28]	30 [21, 39]	1.29 [0.90, 1.68]	>.999	12 [5, 19]	49 [43, 55]
Finland	20-39	Overweight	0.02 [-0.05, 0.10]	0.73	0 [-1, 1]	1 [-1, 2]	0.04 [-0.06, 0.14]	0.80	1 [-1, 2]	2 [-0, 4]
Finland	20-39	Obesity Class I	0.08 [-0.06, 0.27]	0.87	0 [-1, 3]	3 [0, 6]	0.22 [0.02, 0.43]	0.99	-0 [-2, 3]	7 [2, 12]
Finland	40-59	Overweight	0.27 [0.11, 0.43]	>.999	1 [-1, 3]	8 [5, 10]	0.30 [0.13, 0.46]	>.999	4 [2, 6]	13 [10, 17]
Finland	40-59	Obesity Class I	0.50 [0.30, 0.70]	>.999	1 [-1, 3]	14 [10, 17]	0.34 [0.09, 0.60]	0.996	13 [10, 17]	24 [18, 29]
Finland	40-59	Obesity Class II/III	0.57 [0.19, 0.94]	0.999	1 [-3, 6]	15 [9, 22]	0.58 [0.10, 1.06]	0.991	24 [17, 32]	43 [33, 52]
Japan	20-39	Overweight	0.08 [-0.19, 0.52]	0.71	1 [-2, 4]	3 [-1, 9]	0.02 [-0.07, 0.17]	0.66	1 [-0, 2]	2 [-0, 5]
Japan	20-39	Obesity Class I	0.06 [-0.67, 0.92]	0.56	7 [0, 14]	8 [-0, 19]	0.15 [-0.15, 0.48]	0.84	5 [1, 10]	10 [4, 17]
Japan	40-59	Overweight	0.23 [-0.06, 0.52]	0.94	2 [0, 5]	7 [3, 11]	0.18 [0.05, 0.33]	0.997	4 [2, 6]	10 [7, 13]
Japan	40-59	Obesity Class I	-0.28 [-1.16, 0.61]	0.73	14 [5, 24]	8 [0, 18]	0.29 [-0.08, 0.66]	0.93	16 [11, 22]	25 [18, 33]
Japan	60-79	Overweight	0.27 [-0.04, 0.59]	0.95	4 [2, 7]	9 [5, 14]	0.40 [0.23, 0.57]	>.999	7 [4, 10]	20 [16, 23]
Japan	60-79	Obesity Class I	0.20 [-0.74, 1.14]	0.67	14 [5, 23]	17 [6, 29]	0.46 [-0.01, 0.94]	0.97	19 [10, 28]	34 [26, 42]
South Korea	20-39	Overweight	0.02 [-0.07, 0.10]	0.67	1 [-0, 2]	1 [-0, 2]	0.04 [-0.03, 0.11]	0.86	1 [-0, 2]	2 [1, 3]
South Korea	20-39	Obesity Class I	-0.05 [-0.32, 0.17]	0.67	3 [1, 7]	2 [0, 5]	0.09 [-0.12, 0.29]	0.81	3 [-0, 7]	5 [3, 8]
South Korea	40-59	Overweight	0.23 [0.05, 0.41]	0.995	2 [0, 3]	6 [4, 8]	0.24 [0.08, 0.40]	0.999	6 [4, 9]	12 [10, 15]
South Korea	40-59	Obesity Class I	0.40 [-0.16, 0.93]	0.92	7 [1, 14]	15 [9, 20]	0.81 [0.29, 1.29]	0.999	12 [3, 21]	33 [27, 38]
South Korea	60-79	Overweight	0.50 [0.24, 0.76]	>.999	0 [-2, 3]	10 [7, 13]	0.49 [0.20, 0.77]	>.999	9 [3, 14]	21 [18, 24]
South Korea	60-79	Obesity Class I	0.60 [-0.37, 1.47]	0.89	10 [-1, 22]	21 [12, 30]	0.35 [-0.65, 1.34]	0.75	27 [10, 44]	35 [26, 44]
Taiwan	20-39	Overweight	0.05 [-0.23, 0.25]	0.70	0 [-2, 6]	2 [-1, 5]	0.06 [-0.22, 0.26]	0.72	0 [-2, 6]	2 [-1, 5]
Taiwan	40-59	Overweight	0.00 [-0.30, 0.31]	0.51	5 [2, 9]	5 [-0, 11]	-0.16 [-0.60, 0.25]	0.78	9 [3, 16]	6 [-2, 13]
Taiwan	40-59	Obesity Class I	0.42 [-0.33, 1.13]	0.87	1 [-5, 11]	11 [-1, 24]	-0.00 [-0.94, 0.96]	0.50	17 [4, 31]	17 [3, 33]
Taiwan	60-79	Overweight	0.34 [-0.01, 0.67]	0.97	2 [-3, 8]	10 [5, 16]	0.77 [0.02, 1.48]	0.98	7 [-6, 21]	26 [19, 33]
Taiwan	60-79	Obesity Class I					0.86 [-0.82, 2.22]	0.84	11 [-15, 44]	32 [18, 45]
Thailand	20-39	Overweight	-0.14 [-0.30, 0.01]	0.97	2 [1, 3]	0 [-1, 2]	-0.07 [-0.17, 0.01]	0.95	2 [0, 4]	-0 [-2, 1]

Country	Age group	BMI range	Lipid-lowering medicine use				Antihypertensive medicine use			
			Slope and 95% CI	Probability (*P*)*	Predicted value for difference at earliest year and 95% CI	Predicted value for difference at latest year and 95% CI	Slope and 95% CI	Probability (*P*)*	Predicted value for difference at earliest year and 95% CI	Predicted value for difference at latest year and 95% CI
Thailand	20-39	Obesity Class I	-0.12 [-0.39, 0.08]	0.89	2 [1, 5]	1 [-1, 3]	-0.03 [-0.73, 0.15]	0.58	2 [-1, 18]	1 [-4, 4]
Thailand	20-39	Obesity Class II/III	-0.62 [-1.98, 0.14]	0.88	7 [0, 20]	0 [-3, 4]	-0.11 [-0.63, 0.31]	0.68	6 [1, 14]	4 [0, 10]
Thailand	40-59	Overweight	0.12 [-0.05, 0.29]	0.92	2 [0, 3]	4 [1, 7]	0.04 [-0.11, 0.19]	0.69	5 [2, 8]	6 [4, 9]
Thailand	40-59	Obesity Class I	0.51 [0.12, 0.89]	0.993	2 [-1, 7]	12 [7, 18]	0.02 [-0.40, 0.40]	0.53	11 [3, 19]	11 [6, 16]
Thailand	40-59	Obesity Class II/III	0.31 [-0.54, 0.99]	0.79	5 [-2, 18]	12 [2, 22]	0.85 [-0.77, 1.38]	0.87	4 [-5, 38]	28 [14, 38]
Thailand	60-79	Overweight	0.32 [-0.10, 0.71]	0.93	6 [1, 12]	12 [8, 17]	0.04 [-0.30, 0.37]	0.60	11 [5, 18]	13 [8, 17]
Thailand	60-79	Obesity Class I	0.96 [0.04, 1.64]	0.98	2 [-6, 15]	21 [12, 29]	-0.29 [-1.33, 0.75]	0.71	30 [8, 51]	21 [10, 33]
USA	20-39	Overweight	0.04 [-0.05, 0.13]	0.82	-1 [-2, 1]	1 [-1, 3]	0.02 [-0.05, 0.08]	0.67	0 [-1, 2]	1 [-0, 2]
USA	20-39	Obesity Class I	0.12 [-0.01, 0.27]	0.97	-0 [-3, 2]	3 [1, 6]	0.02 [-0.09, 0.13]	0.61	3 [1, 5]	3 [2, 6]
USA	20-39	Obesity Class II/III	-0.02 [-0.18, 0.14]	0.58	3 [0, 6]	3 [0, 5]	0.09 [-0.16, 0.33]	0.75	7 [2, 13]	10 [6, 14]
USA	40-59	Overweight	0.10 [-0.11, 0.31]	0.83	5 [1, 9]	8 [4, 12]	0.04 [-0.18, 0.26]	0.64	8 [4, 12]	10 [6, 13]
USA	40-59	Obesity Class I	0.23 [-0.02, 0.47]	0.97	7 [2, 11]	14 [9, 18]	0.25 [-0.05, 0.55]	0.95	13 [7, 18]	21 [16, 25]
USA	40-59	Obesity Class II/III	0.29 [-0.05, 0.62]	0.95	9 [3, 15]	18 [12, 24]	-0.06 [-0.46, 0.33]	0.62	33 [25, 41]	31 [24, 37]
USA	60-79	Overweight	0.43 [0.05, 0.81]	0.99	-0 [-7, 7]	13 [6, 20]	0.56 [0.21, 0.91]	>.999	3 [-4, 10]	20 [14, 26]
USA	60-79	Obesity Class I	0.59 [0.17, 1.02]	0.996	4 [-3, 11]	22 [14, 30]	0.38 [-0.02, 0.79]	0.97	17 [9, 25]	29 [22, 36]
USA	60-79	Obesity Class II/III	0.57 [-0.02, 1.12]	0.97	7 [-4, 18]	24 [15, 34]	1.00 [0.47, 1.53]	>.999	18 [7, 30]	49 [42, 57]

* Probability of the slope being a true negative or a true positive

Appendix Figure 1. Flowchart of data cleaning.

Flowchart showing the process of data inclusion and cleaning.

* This number includes 21,217 participants in six studies in Finland who were aged <30 and >60 years because the analysis age range in Finland was 30-59 years as explained in Methods. It also includes a further 729 participants from The International Collaborative Study of Cardiovascular Disease in Asia (InterASIA) study in Thailand who were less than 40 years. These participants were dropped because the study only included those aged 35 years and older.

** 167,536 participants with missing anthropometric data were initially measured as part of the 16 rounds of Health Survey for England but their measurements were judged unreliable by the study's own validation protocol – for example height was considered invalid if the participant was wearing shoes or the measurement was taken on an uneven surface.

*** 91,734 participants with missing blood pressure and 118,087 participants with missing cholesterol data from the Health Survey for England (due to survey validation protocol) and from the National Health and Nutrition Surveys in Japan, due to lower participation in the clinical measurement and blood tests than in the anthropometric measurements.

**** The margin of error was determined using the Cholesterol Reference Method Laboratory Network's permitted measurement error limits – see Methods for details.

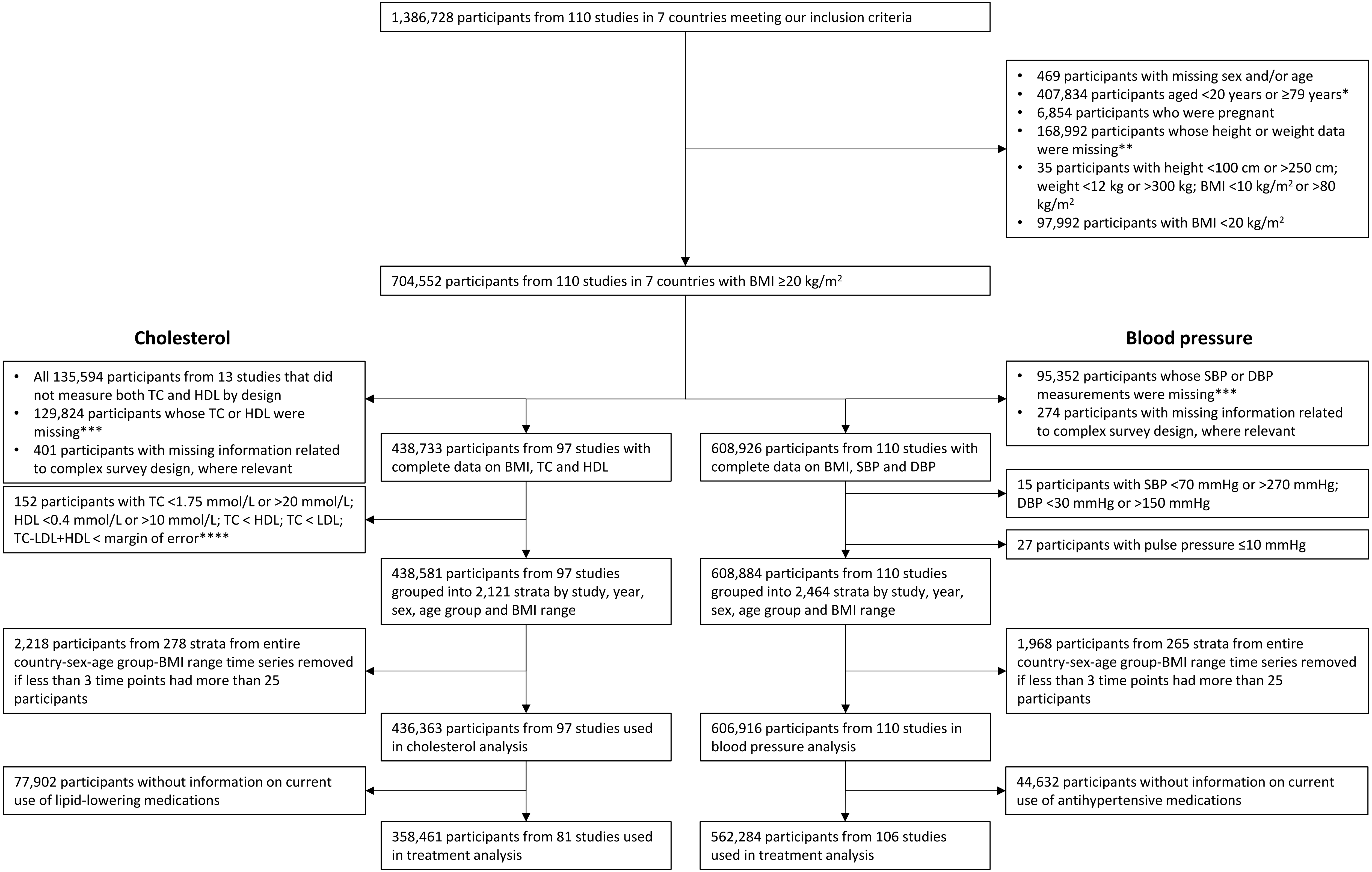
1,386,728 participants from 110 studies in 7 countries meeting our inclusion criteria
• 469 participants with missing sex and/or age
• 407,834 participants aged <20 years or ≥79 years*
• 6,854 participants who were pregnant
• 168,992 participants whose height or weight data were missing**
• 35 participants with height <100 cm or >250 cm; weight <12 kg or >300 kg; BMI <10 kg/m^2 or >80 kg/m^2
• 97,992 participants with BMI <20 kg/m^2
704,552 participants from 110 studies in 7 countries with BMI ≥20 kg/m^2
Cholesterol
Blood pressure
• All 135,594 participants from 13 studies that did not measure both TC and HDL by design
• 129,824 participants whose TC or HDL were missing***
• 401 participants with missing information related to complex survey design, where relevant
• 95,352 participants whose SBP or DBP measurements were missing***
• 274 participants with missing information related to complex survey design, where relevant
438,733 participants from 97 studies with complete data on BMI, TC and HDL
608,926 participants from 110 studies with complete data on BMI, SBP and DBP
152 participants with TC <1.75 mmol/L or >20 mmol/L; HDL <0.4 mmol/L or >10 mmol/L; TC < HDL; TC < LDL; TC-LDL+HDL < margin of error****
15 participants with SBP <70 mmHg or >270 mmHg; DBP <30 mmHg or >150 mmHg
27 participants with pulse pressure ≤10 mmHg
438,581 participants from 97 studies grouped into 2,121 strata by study, year, sex, age group and BMI range
608,884 participants from 110 studies grouped into 2,464 strata by study, year, sex, age group and BMI range
2,218 participants from 278 strata from entire country-sex-age group-BMI range time series removed if less than 3 time points had more than 25 participants
1,968 participants from 265 strata from entire country-sex-age group-BMI range time series removed if less than 3 time points had more than 25 participants
436,363 participants from 97 studies used in cholesterol analysis
606,916 participants from 110 studies in blood pressure analysis
77,902 participants without information on current use of lipid-lowering medications
44,632 participants without information on current use of antihypertensive medications
358,461 participants from 81 studies used in treatment analysis
562,284 participants from 106 studies used in treatment analysis

Appendix Figure 2. Trends in prevalence of obesity and overweight.

For each country and sex, the three panels show the share of population in different BMI ranges. Within each age group, prevalence was age standardised as described in Methods. Prevalences are from a pooled analysis of population-based studies.[7] BMI ranges for the displayed categories are defined in Appendix Text 1.

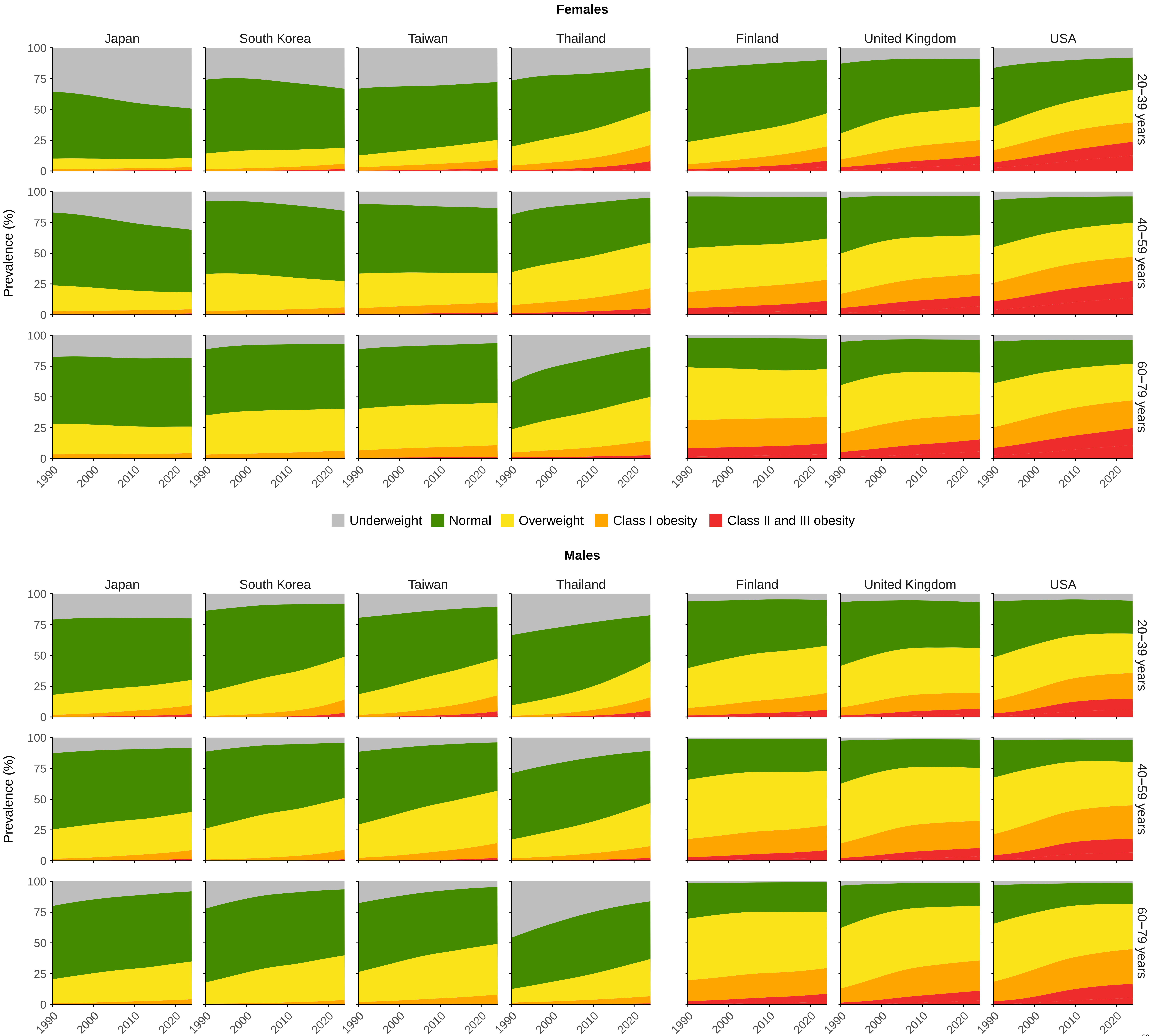
Females
Japan
South Korea
Taiwan
Thailand
Finland
United Kingdom
USA
20–39 years
40–59 years
60–79 years
Prevalence (%)
0
25
50
75
100
1990
2000
2010
2020
Underweight
Normal
Overweight
Class I obesity
Class II and III obesity
Males

Appendix Figure 3. Trends in difference in age-standardised mean non-HDL cholesterol, mean HDL cholesterol and mean SBP between obesity and overweight and normal BMI.

Each point represents one sex-age-BMI group from one study, calculated as stated in Methods. Error bars show 95% confidence intervals, calculated as stated in Methods. See Appendix Figure 9 for data points together with fitted trends. See Appendix Figure 13 for Figure without exclusions based on number of participants.

Age range for data from Finland is 30-59 years, grouped into 30-39 years and 40-59 years.

Non-HDL cholesterol

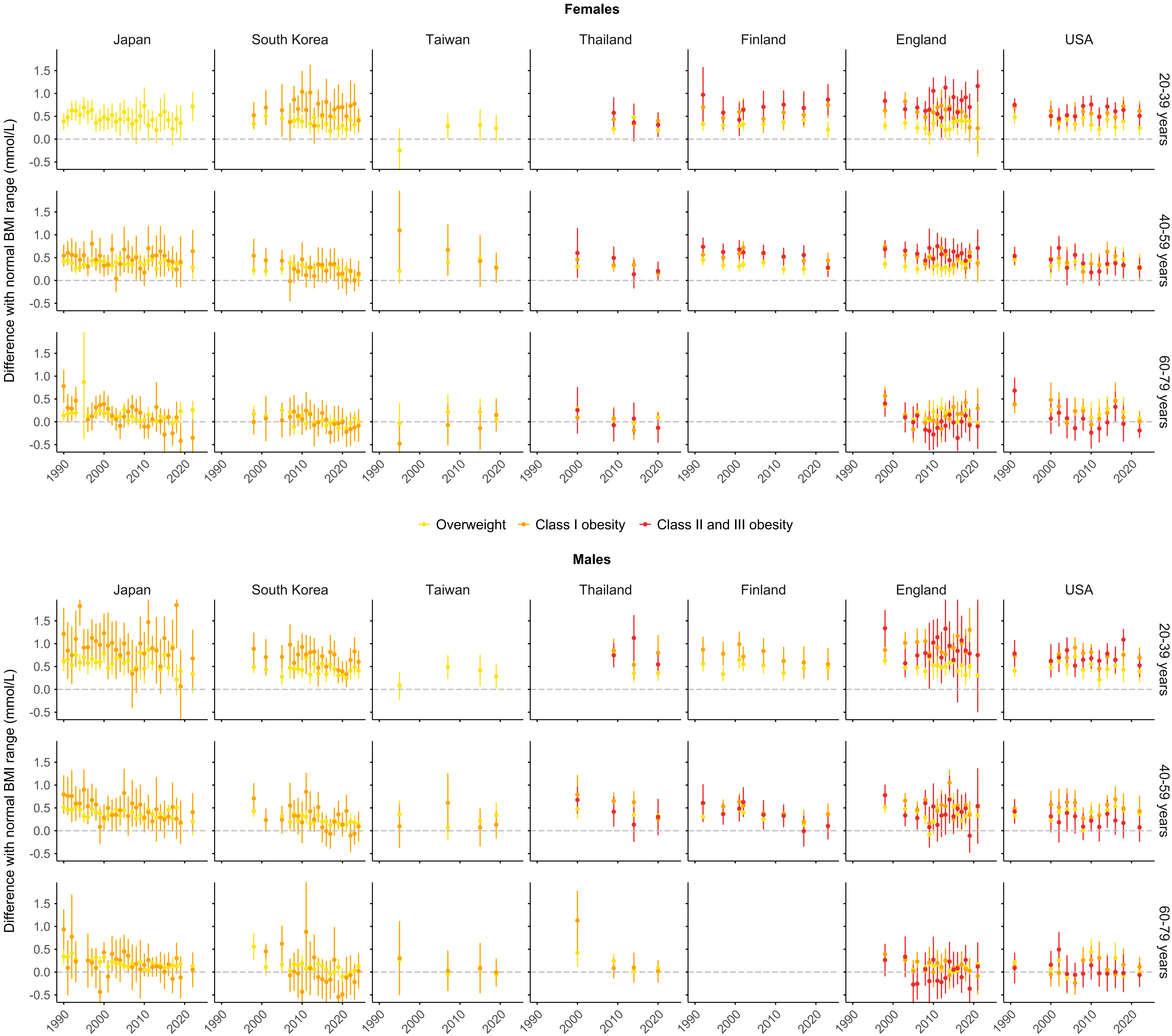

HDL cholesterol

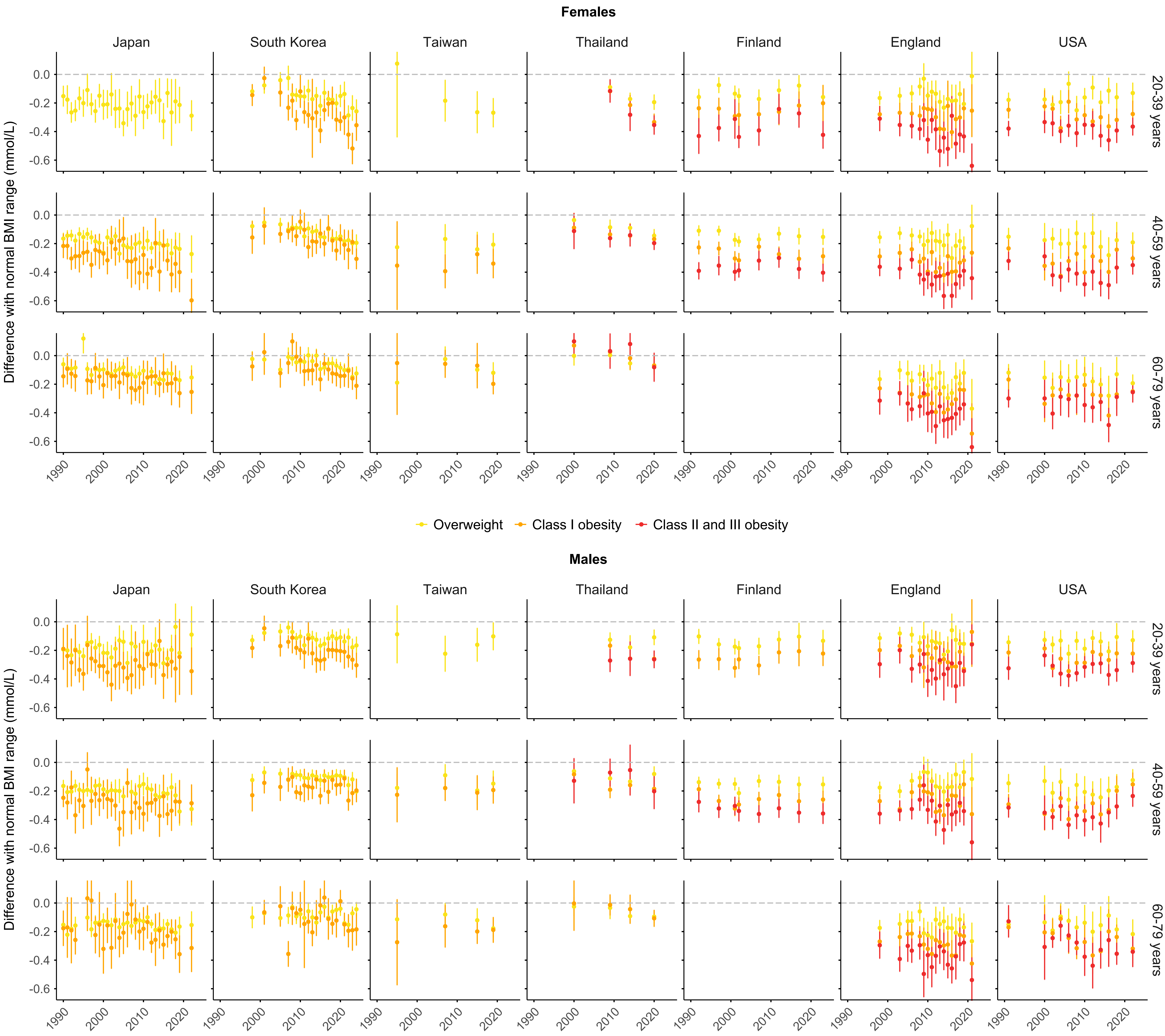

Systolic blood pressure

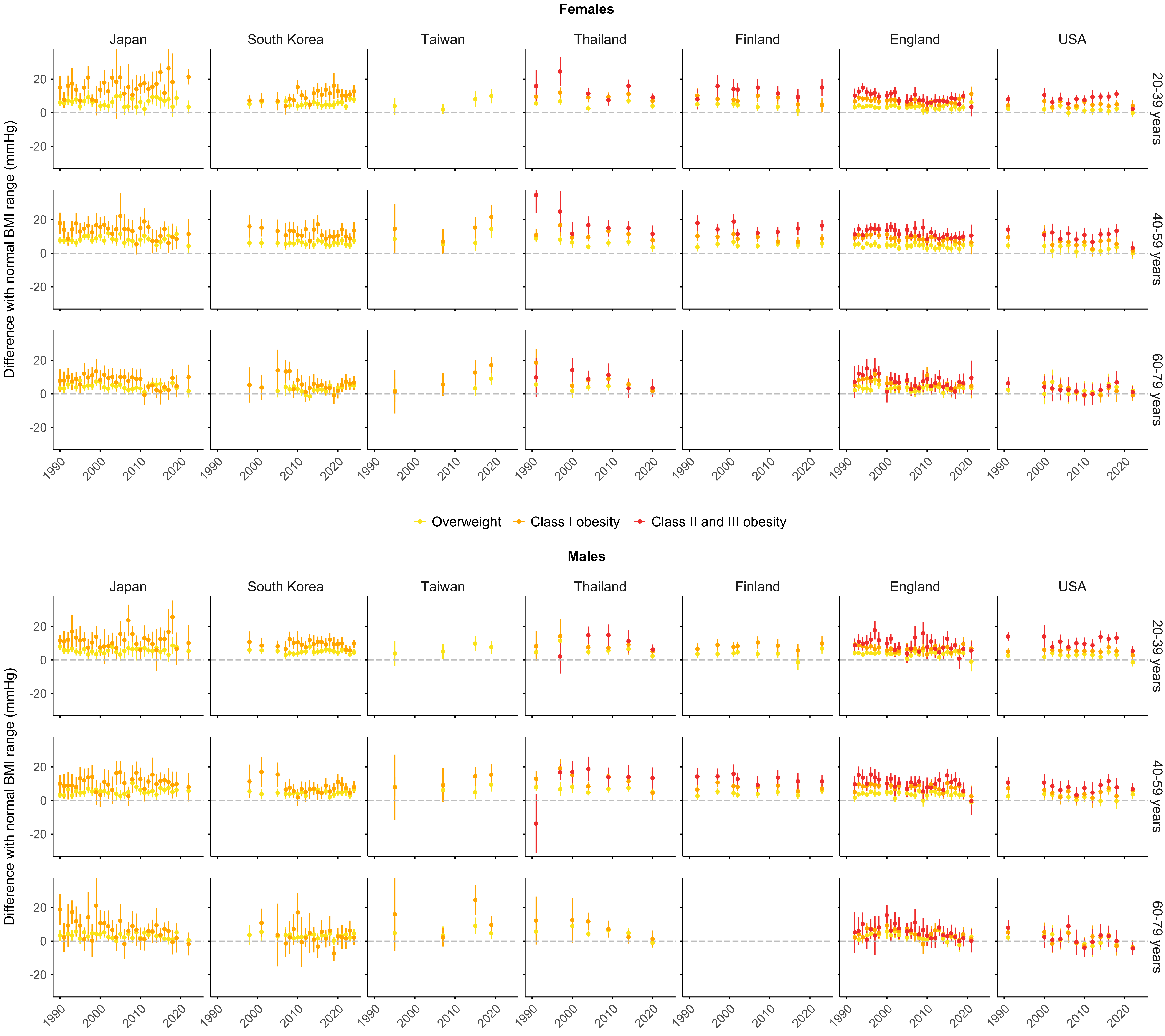

Appendix Figure 4. Cholesterol and blood pressure in normal BMI.

The figure shows age-standardised mean non-HDL cholesterol, mean HDL cholesterol and mean SBP at the earliest year of data versus at the latest year of data, by sex and age group. Probabilities *P* were calculated for the probability that change between the two years was different from null as described in Methods. The points are labelled with the country that they represent. ENG: England; FIN: Finland; JPN: Japan; KOR: South Korea; THA: Thailand; TWN: Taiwan; USA: United States of America. The year ranges for each country are ENG: 1992 to 2021; FIN: 1992 to 2023; JPN: 1990 to 2022; KOR: 1998 to 2024; THA: 1995 to 2020; TWN: 1995 to 2019; USA: 1991 to 2022.

Age range for data from Finland is 30-59 years, grouped into 30-39 years and 40-59 years.

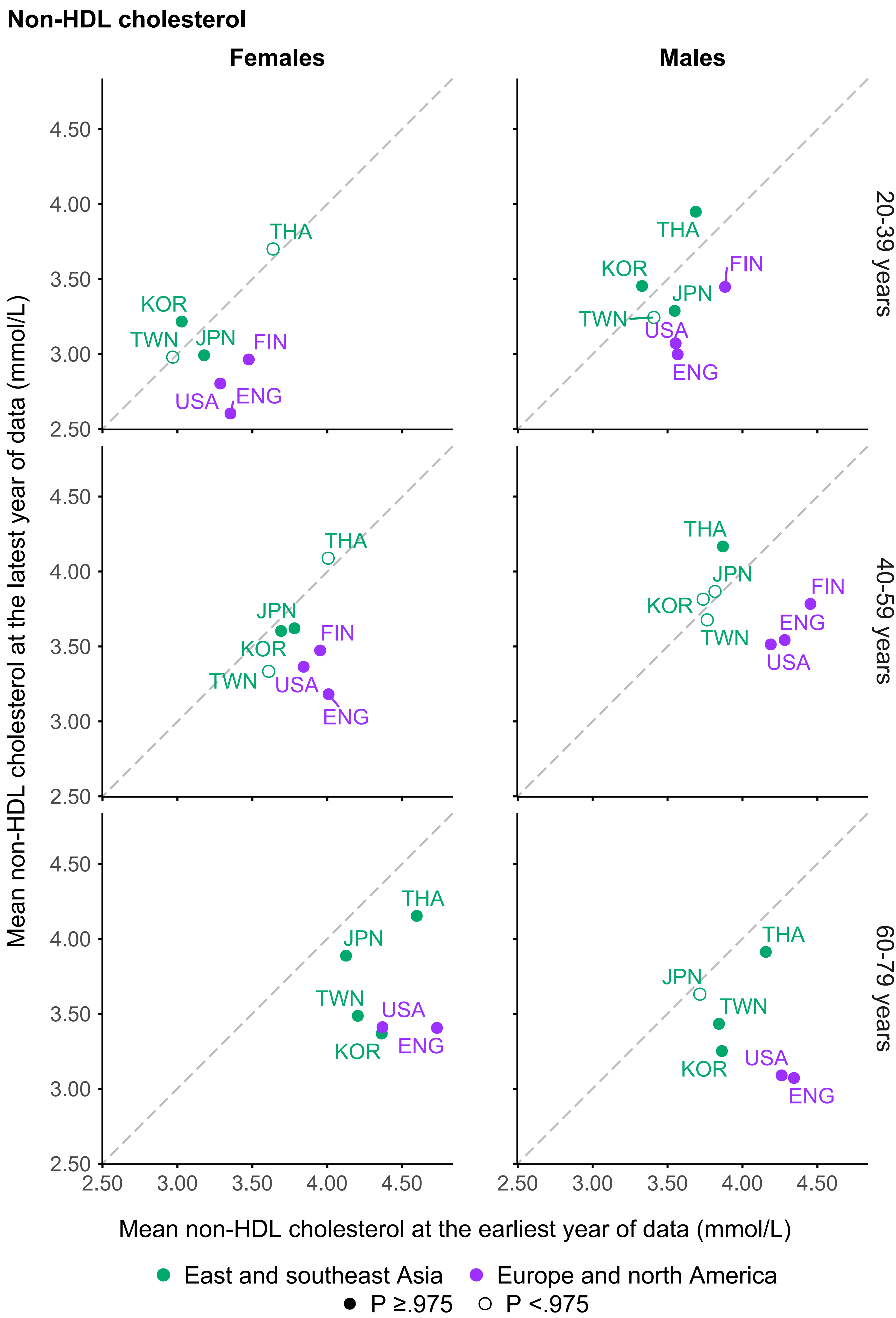

Non-HDL cholesterol
Females
Males
20-39 years
40-59 years
60-79 years
THA
KOR
TWN
JPN
FIN
USA
ENG
4.50
4.00
3.50
3.00
2.50
Mean non-HDL cholesterol at the latest year of data (mmol/L)
Mean non-HDL cholesterol at the earliest year of data (mmol/L)
East and southeast Asia
Europe and north America
P ≥.975
P <.975

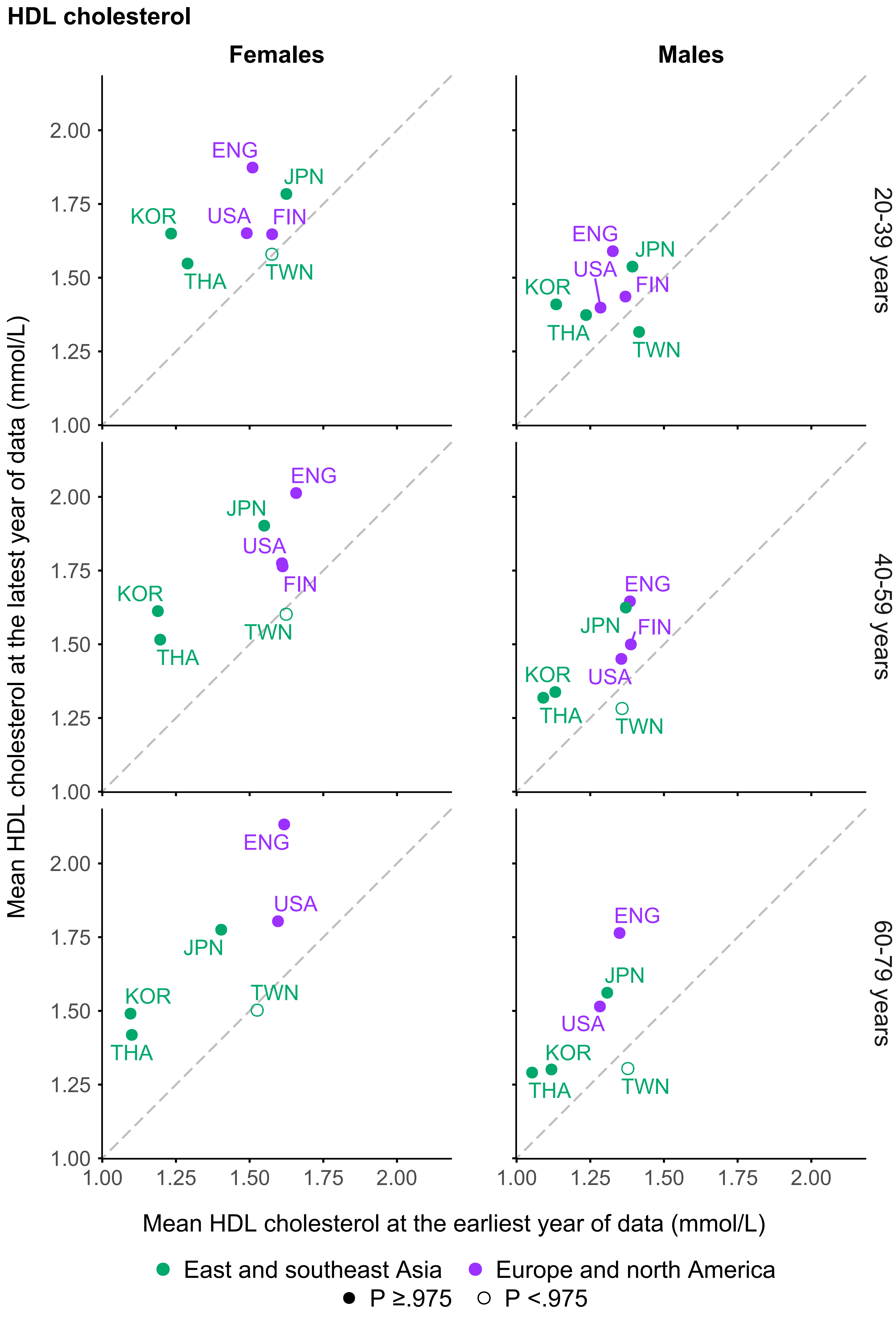
HDL cholesterol
Females
Males
20-39 years
40-59 years
60-79 years
Mean HDL cholesterol at the latest year of data (mmol/L)
Mean HDL cholesterol at the earliest year of data (mmol/L)
1.00
1.25
1.50
1.75
2.00
ENG
JPN
KOR
USA
FIN
THA
TWN
East and southeast Asia
Europe and north America
P ≥.975
P <.975

Systolic blood pressure

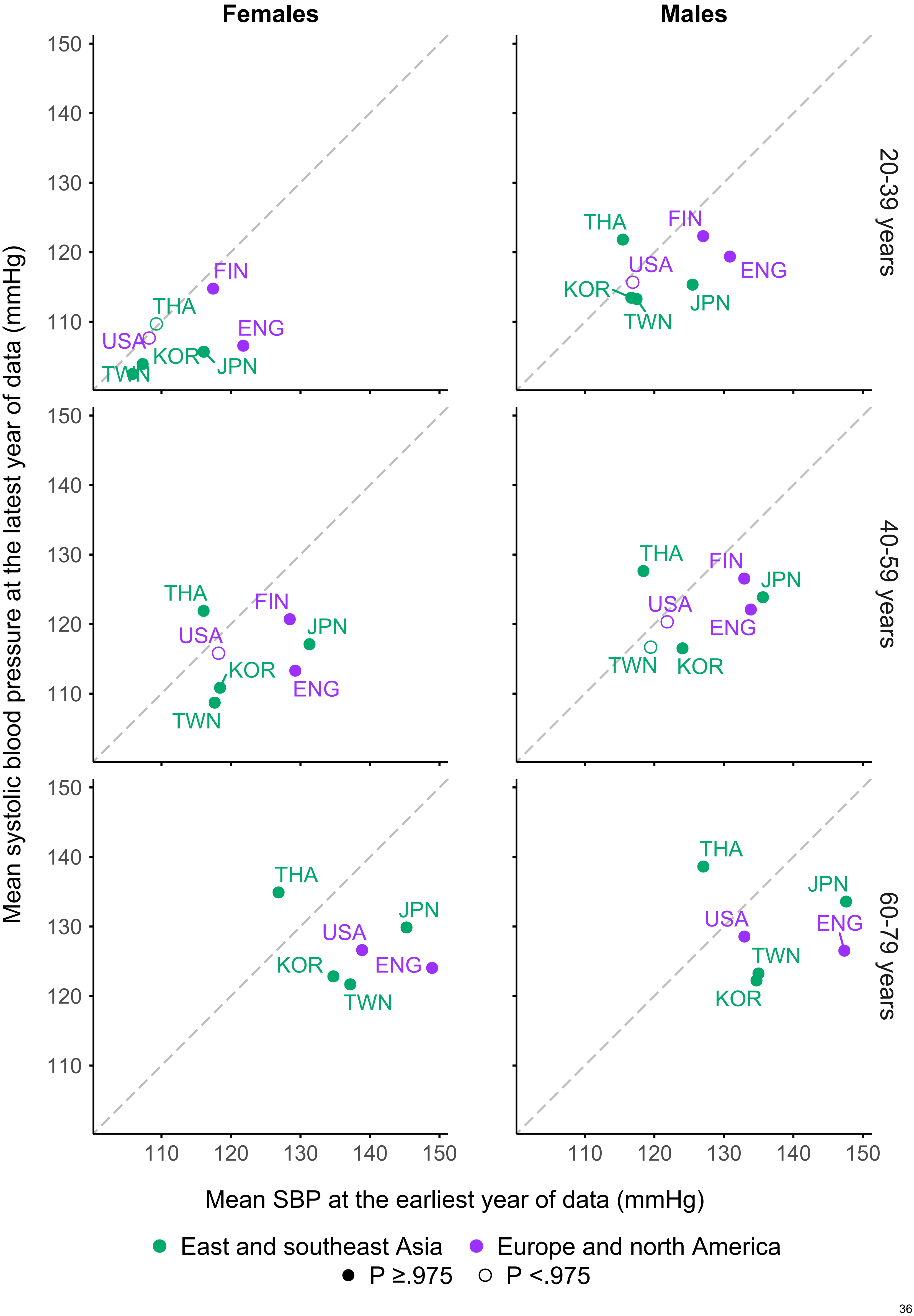

Appendix Figure 5. Difference in cholesterol and blood pressure between obesity and overweight and normal BMI.

The figure shows the difference in age-standardised mean non-HDL, mean HDL and mean SBP between obesity or overweight and normal BMI versus age-standardised mean in the normal BMI, at the latest year of data by sex, age group and BMI range. The points are labelled with the country that they represent. ENG: England; FIN: Finland; JPN: Japan; KOR: South Korea; THA: Thailand; TWN: Taiwan; USA: United States of America. The year ranges for each country are ENG: 1992 to 2021; FIN: 1992 to 2023; JPN: 1990 to 2022; KOR: 1998 to 2024; THA: 1995 to 2020; TWN: 1995 to 2019; USA: 1991 to 2022.

Age range for data from Finland is 30-59 years, grouped into 30-39 years and 40-59 years.

Non-HDL cholesterol

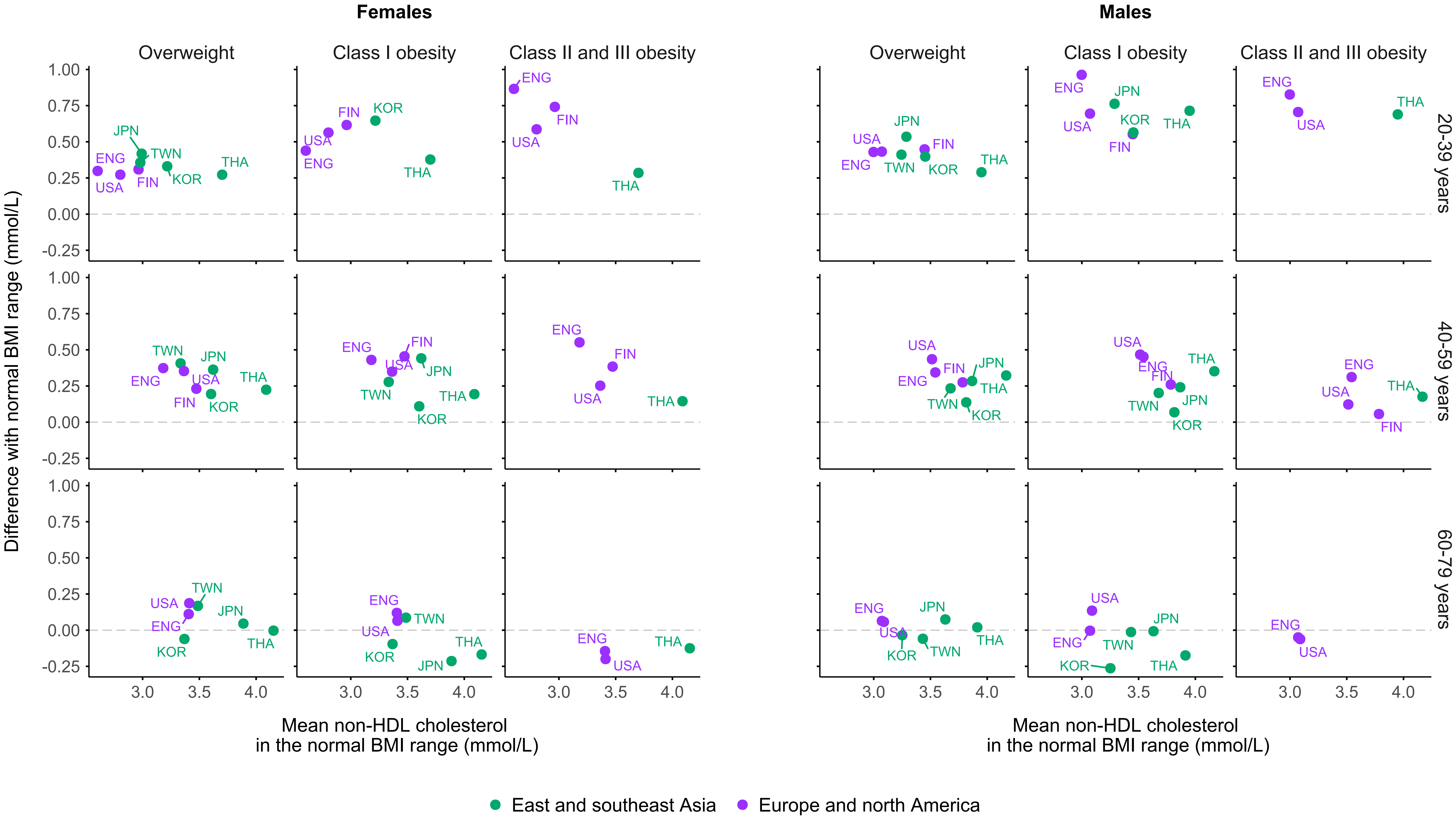

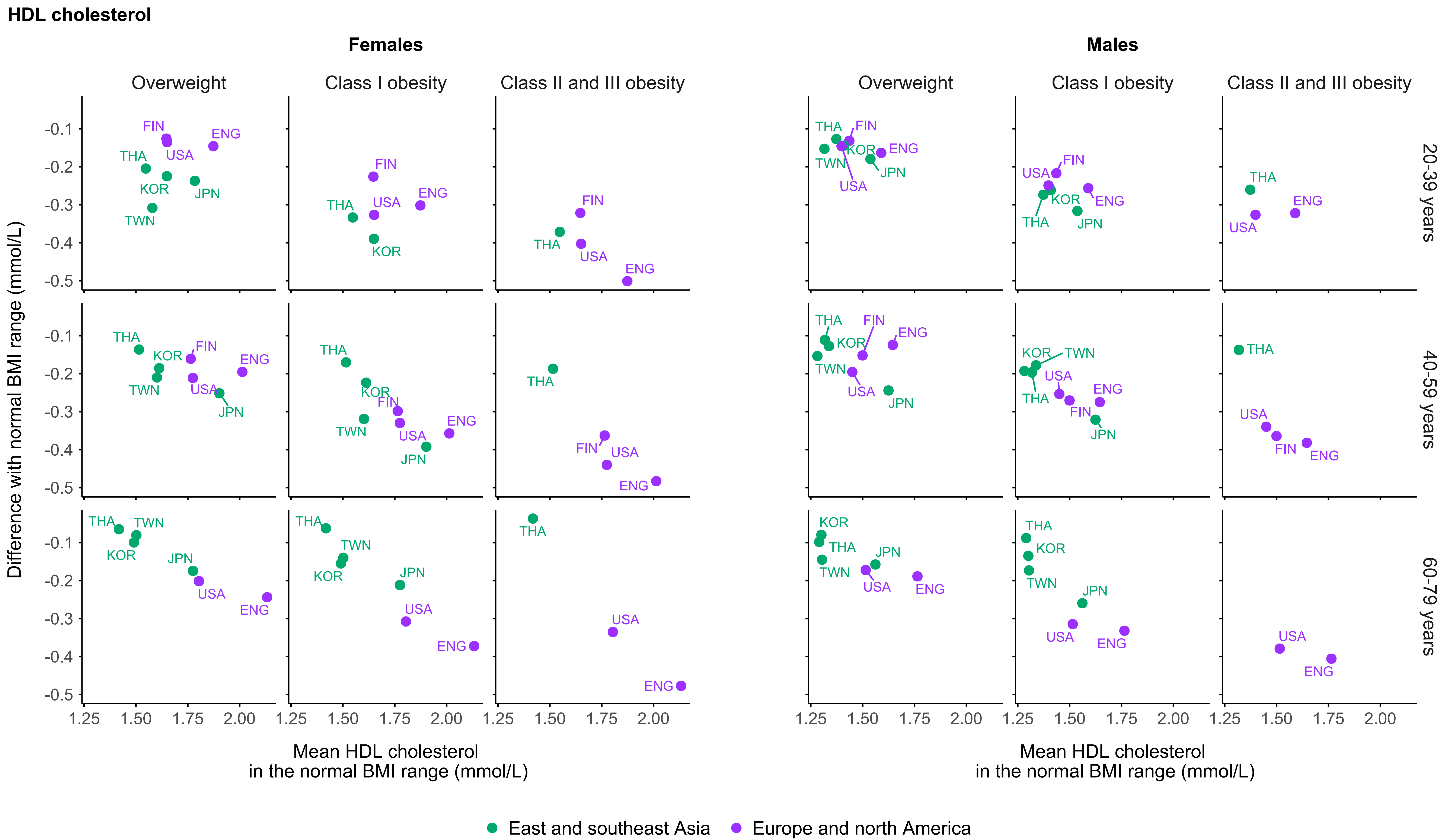
HDL cholesterol
Females
Males
Overweight
Class I obesity
Class II and III obesity
20-39 years
40-59 years
60-79 years
Difference with normal BMI range (mmol/L)
Mean HDL cholesterol in the normal BMI range (mmol/L)
East and southeast Asia
Europe and north America
THA
TWN
KOR
JPN
FIN
USA
ENG

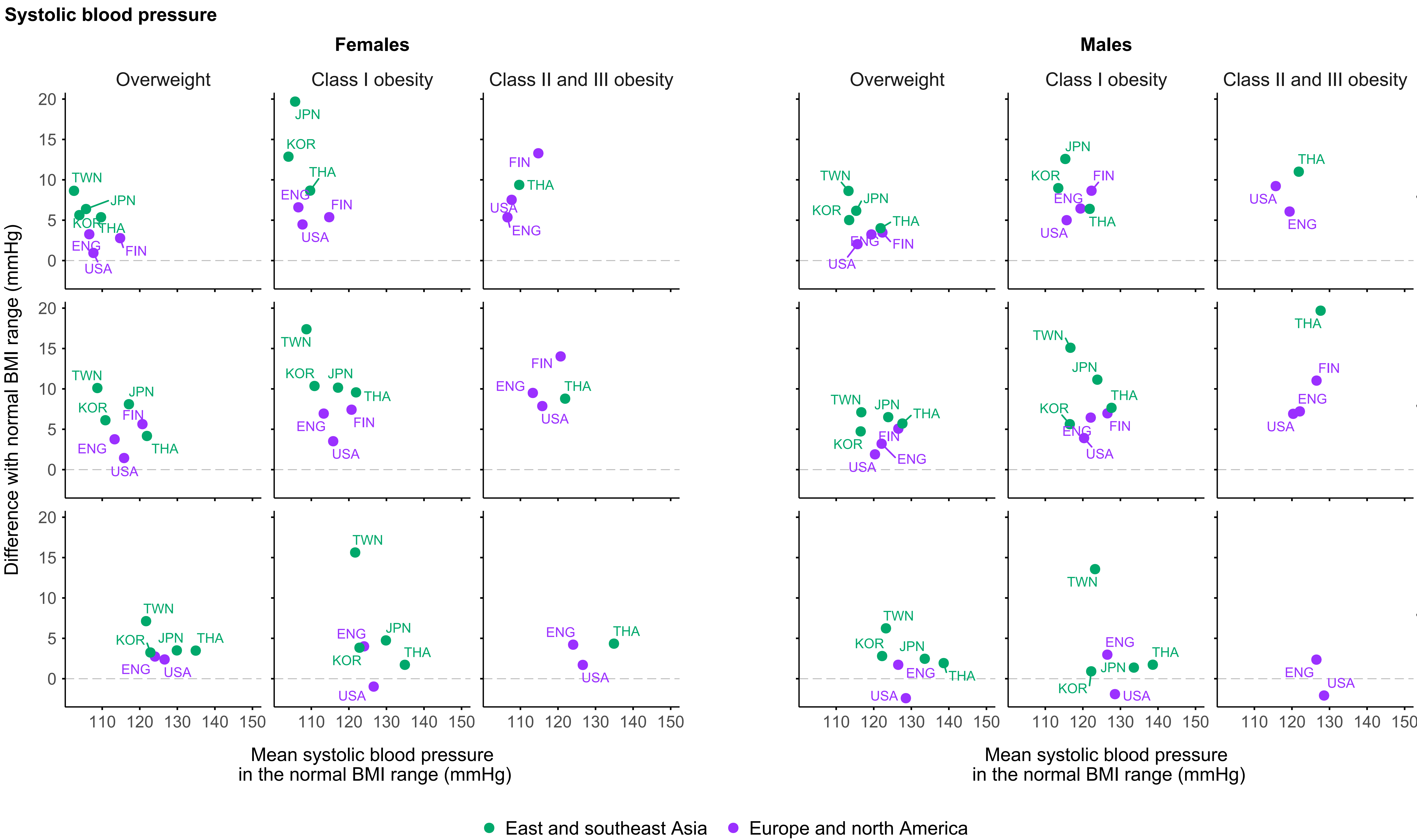

Systolic blood pressure
Females
Males
Overweight
Class I obesity
Class II and III obesity
20-39 years
40-59 years
60-79 years
Difference with normal BMI range (mmHg)
Mean systolic blood pressure in the normal BMI range (mmHg)
TWN
JPN
KOR
THA
ENG
FIN
USA
East and southeast Asia
Europe and north America

Appendix Figure 6. Trends in difference in age-standardised proportion of participants using lipid-lowering or antihypertensive medicines between obesity and overweight and normal BMI.

Each point represents one sex-age-BMI group from one study, calculated as stated in Methods. Error bars show 95% confidence intervals, calculated as stated in Methods. See Appendix Figure 12 for data points together with fitted trends. See Appendix Figure 18 for Figure without exclusions based on number of participants.

Age range for data from Finland is 30-59 years, grouped into 30-39 years and 40-59 years.

Proportion using lipid-lowering medicines

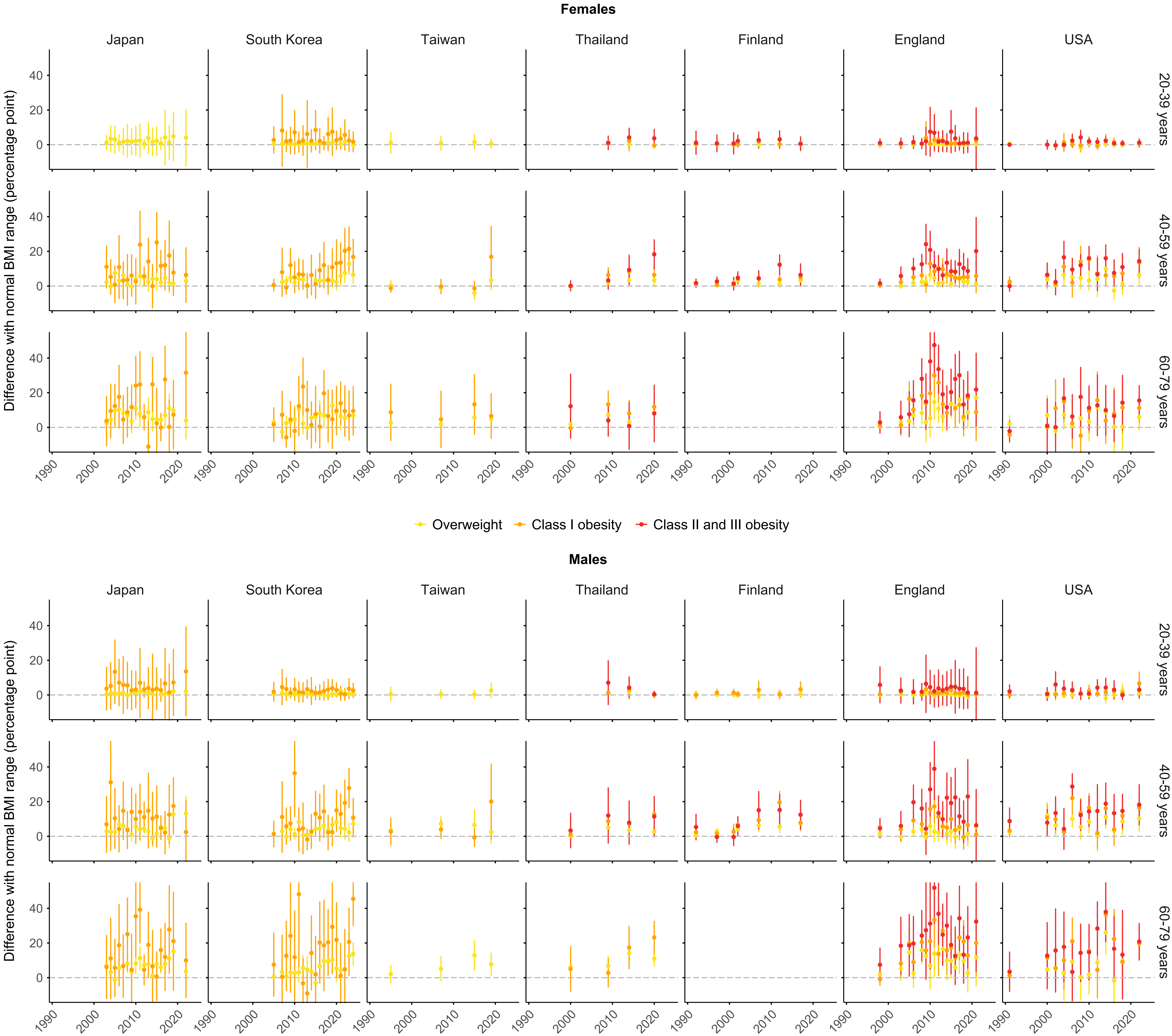

Proportion using antihypertensive medicines

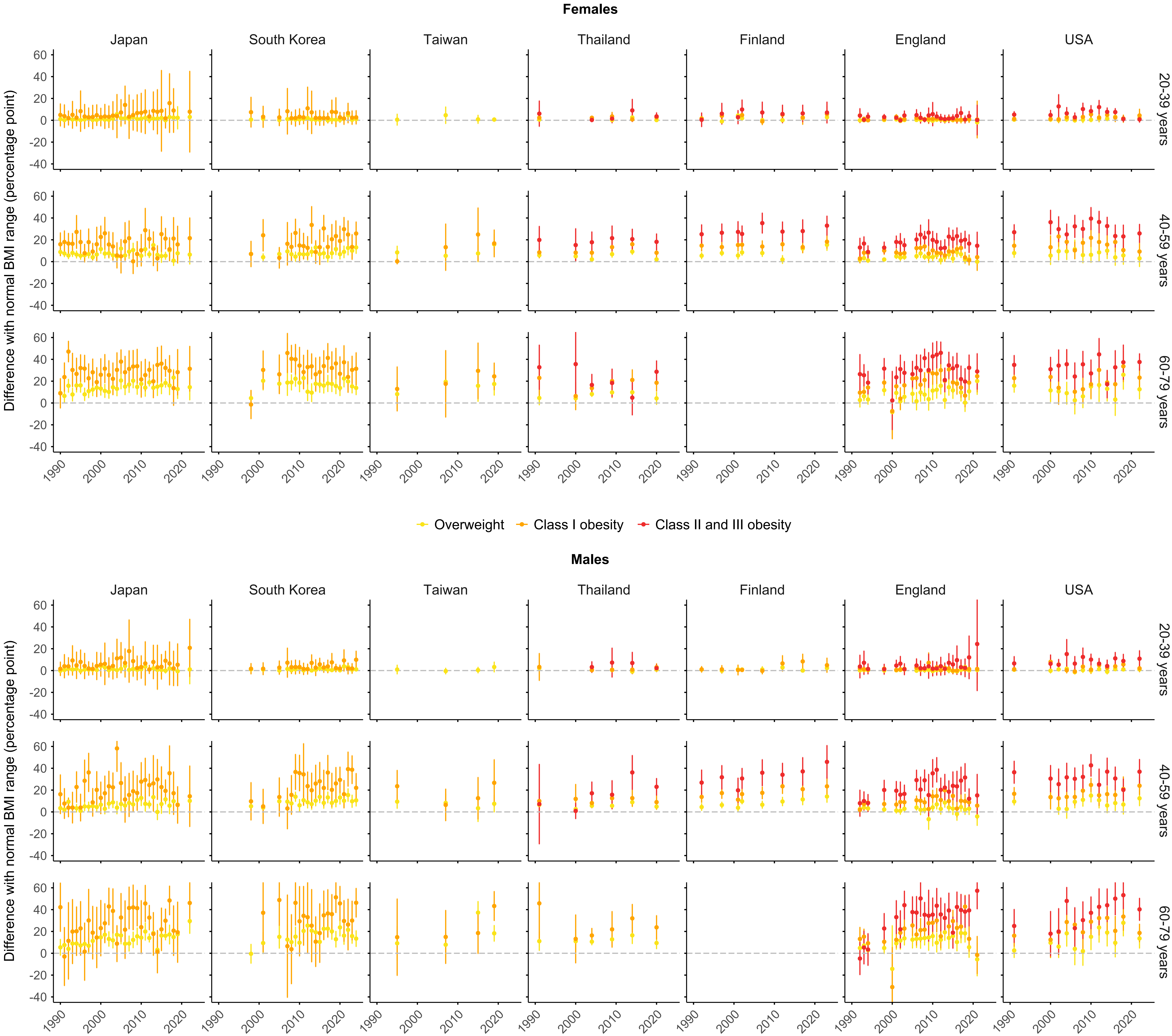

Appendix Figure 7. Use of lipid-lowering and antihypertensive medicines in normal BMI.

The figure shows age-standardised proportion of participants using lipid-lowering and antihypertensive medicines at the earliest year of data versus at the latest year of data, by sex and age group. Probabilities *P* were calculated for the probability that change between the two years was different from null as described in Methods. The points are labelled with the country that they represent. ENG: England; FIN: Finland; JPN: Japan; KOR: South Korea; THA: Thailand; TWN: Taiwan; USA: United States of America. The year ranges for each country are ENG: 1992 to 2021; FIN: 1992 to 2023; JPN: 1990 to 2022; KOR: 1998 to 2024; THA: 1995 to 2020; TWN: 1995 to 2019; USA: 1991 to 2022.

Age range for data from Finland is 30-59 years, grouped into 30-39 years and 40-59 years.

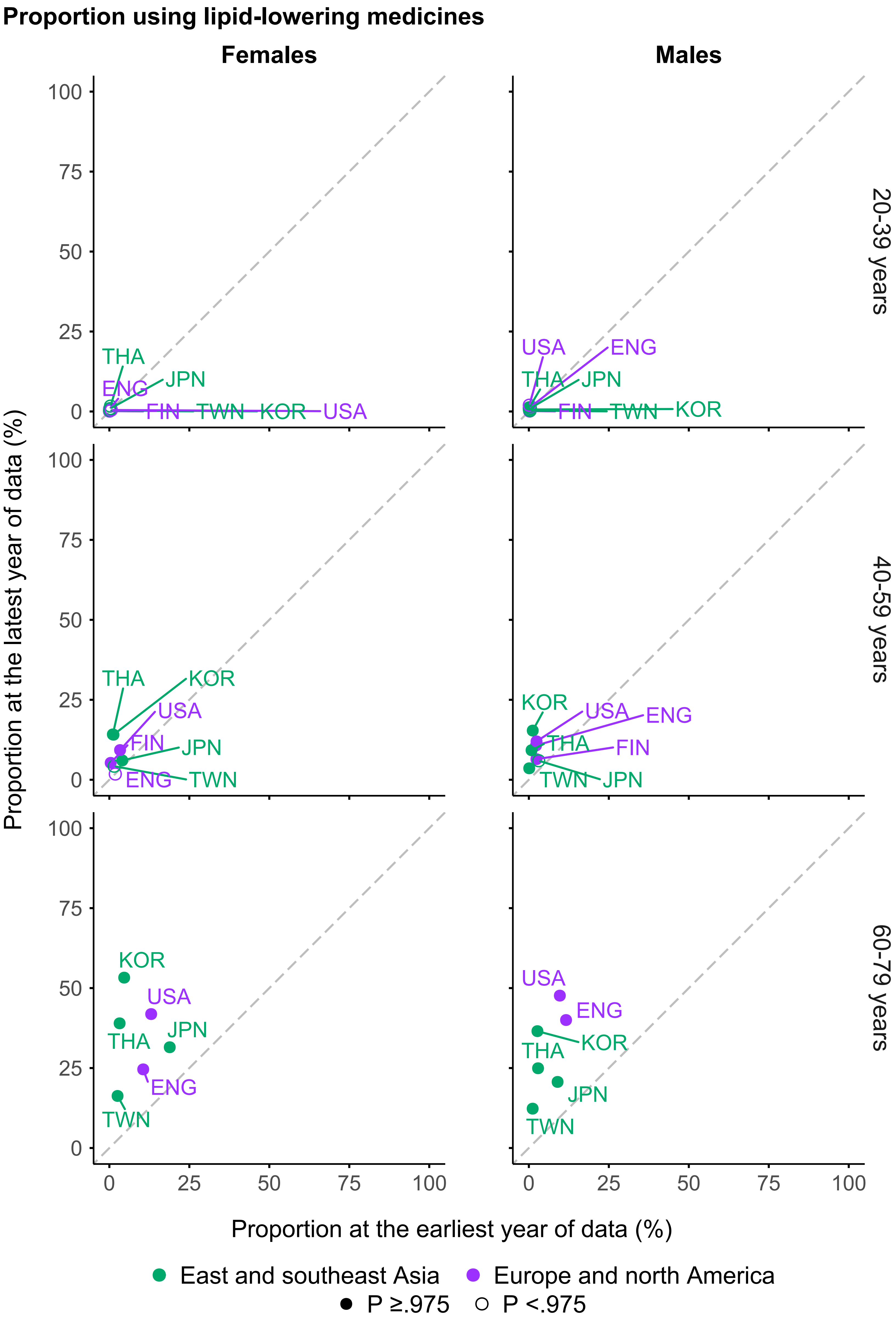
Proportion using lipid-lowering medicines
Females
Males
20-39 years
40-59 years
60-79 years
Proportion at the latest year of data (%)
Proportion at the earliest year of data (%)
0
25
50
75
100
THA
ENG
JPN
FIN
TWN
KOR
USA
East and southeast Asia
Europe and north America
P ≥.975
P <.975

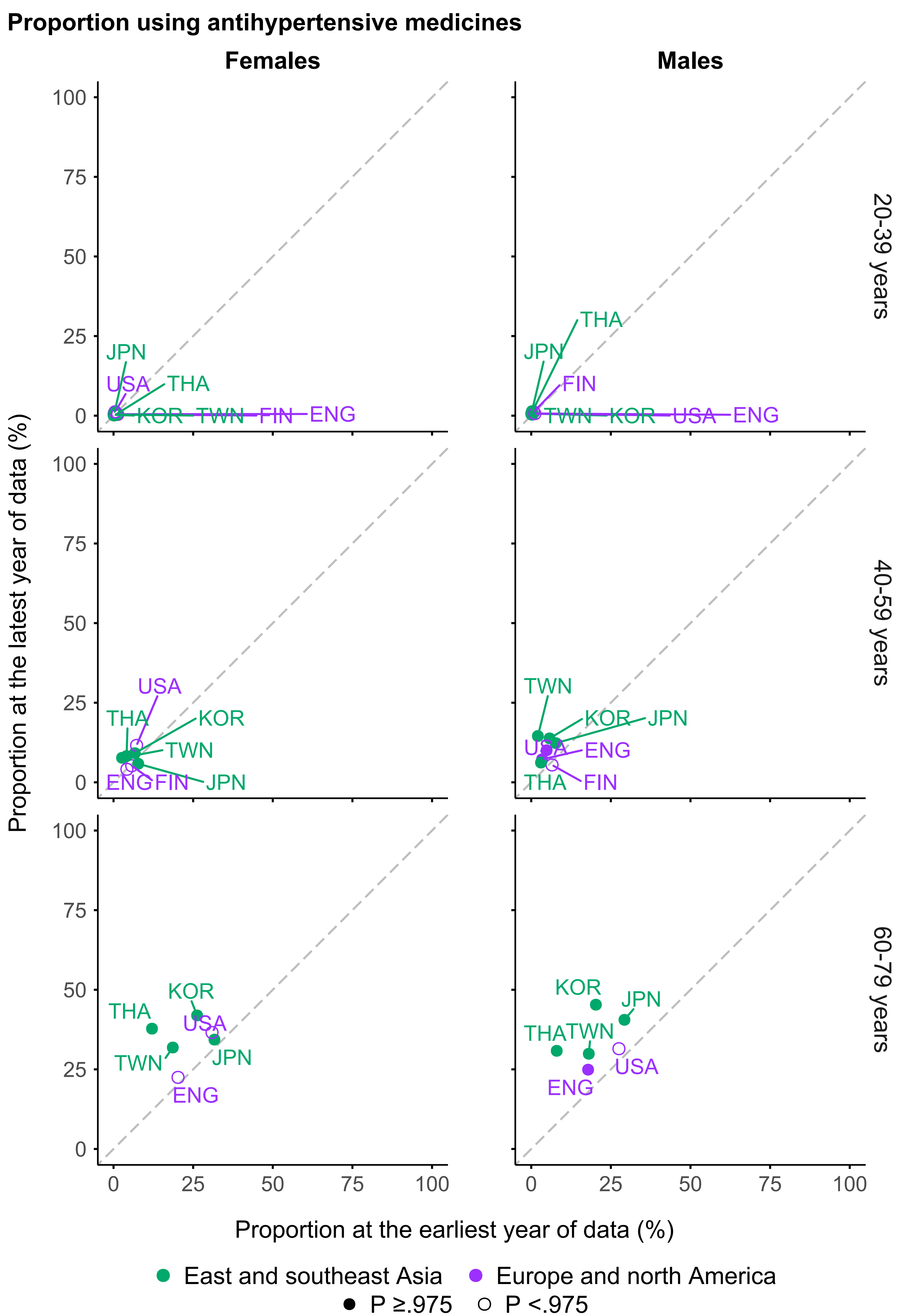

Proportion using antihypertensive medicines
Females
Males
20-39 years
40-59 years
60-79 years
Proportion at the latest year of data (%)
Proportion at the earliest year of data (%)
0
25
50
75
100
JPN
USA
THA
KOR
TWN
FIN
ENG
East and southeast Asia
Europe and north America
P ≥.975
P <.975

Appendix Figure 8. Difference in use of lipid-lowering or antihypertensive medicines between obesity and overweight and normal BMI.

The figure shows the difference in age-standardised proportion of participants using lipid-lowering or antihypertensive medicines between obesity or overweight and normal BMI versus age-standardised proportion in the normal BMI, at the latest year of data by sex, age group and BMI range. The points are labelled with the country that they represent ENG: England; FIN: Finland; JPN: Japan; KOR: South Korea; THA: Thailand; TWN: Taiwan; USA: United States of America. The year ranges for each country are ENG: 1992 to 2021; FIN: 1992 to 2023; JPN: 1990 to 2022; KOR: 1998 to 2024; THA: 1995 to 2020; TWN: 1995 to 2019; USA: 1991 to 2022.

Age range for data from Finland is 30-59 years, grouped into 30-39 years and 40-59 years.

Proportion using lipid-lowering medicines

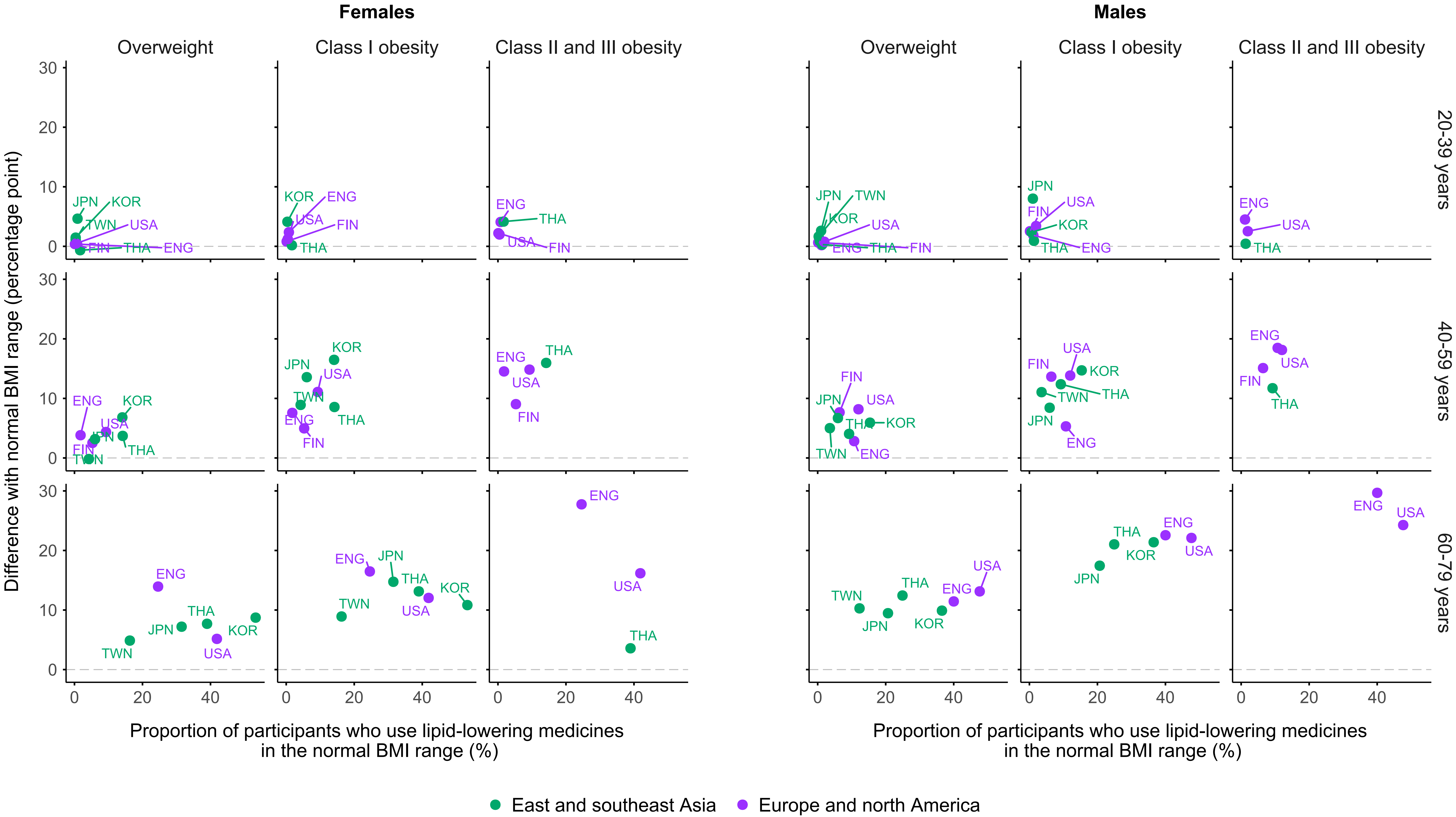

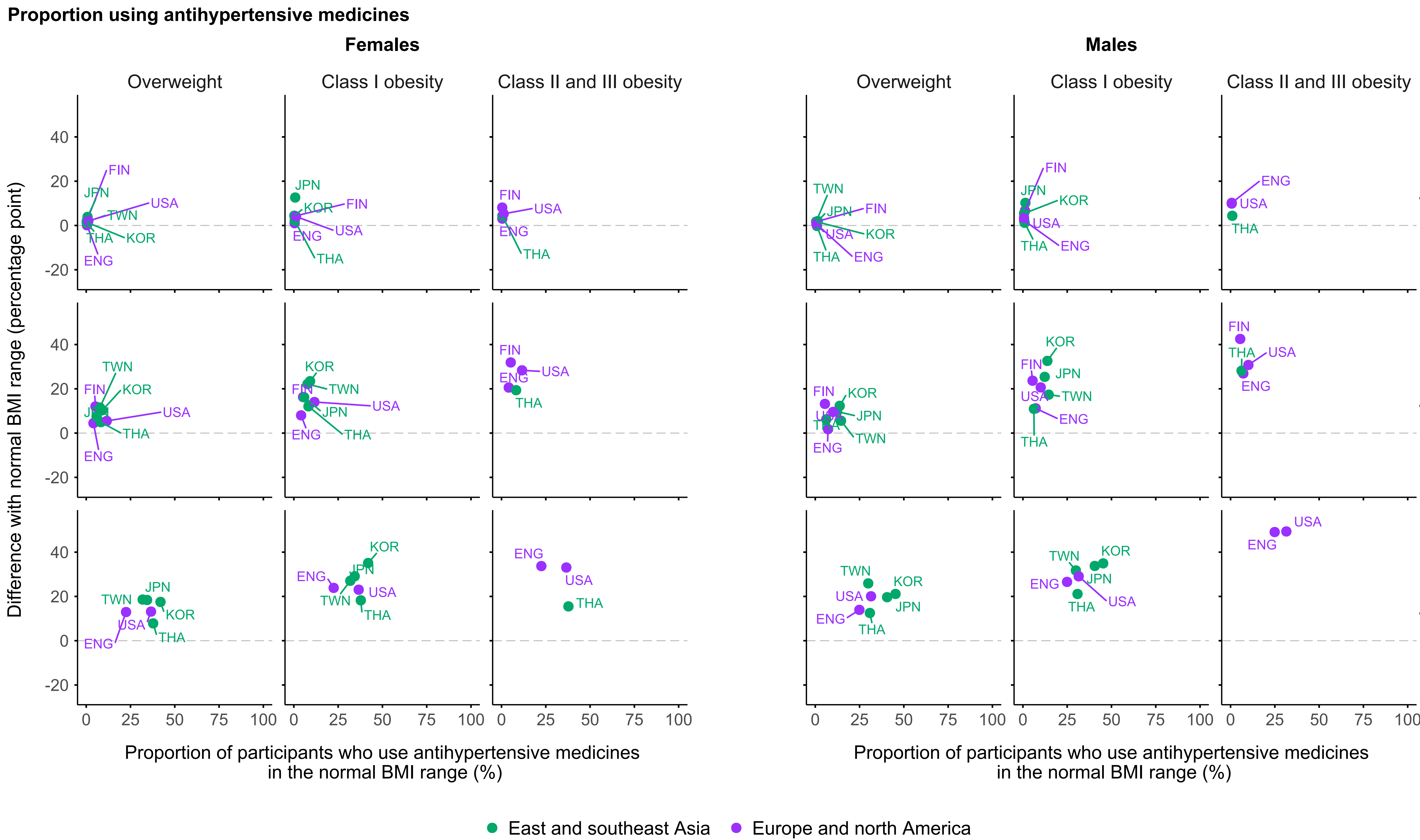
Proportion using antihypertensive medicines
Females
Males
Overweight
Class I obesity
Class II and III obesity
20-39 years
40-59 years
60-79 years
Difference with normal BMI range (percentage point)
Proportion of participants who use antihypertensive medicines in the normal BMI range (%)
East and southeast Asia
Europe and north America

Appendix Figure 9. Trends in age-standardised mean non-HDL cholesterol, HDL cholesterol and SBP in normal BMI with fitted linear and LOESS regressions.

Each point represents one sex-age-BMI group from one study, calculated as stated in Methods. Error bars show 95% confidence intervals, calculated as stated in Methods. As stated in Appendix Text 3, LOESS models were fitted to time series with five or more data points. Shaded areas show the 95% confidence intervals of the annual predictions, calculated as stated in Methods.

Age range for data from Finland is 30-59 years, grouped into 30-39 years and 40-59 years.

Japan

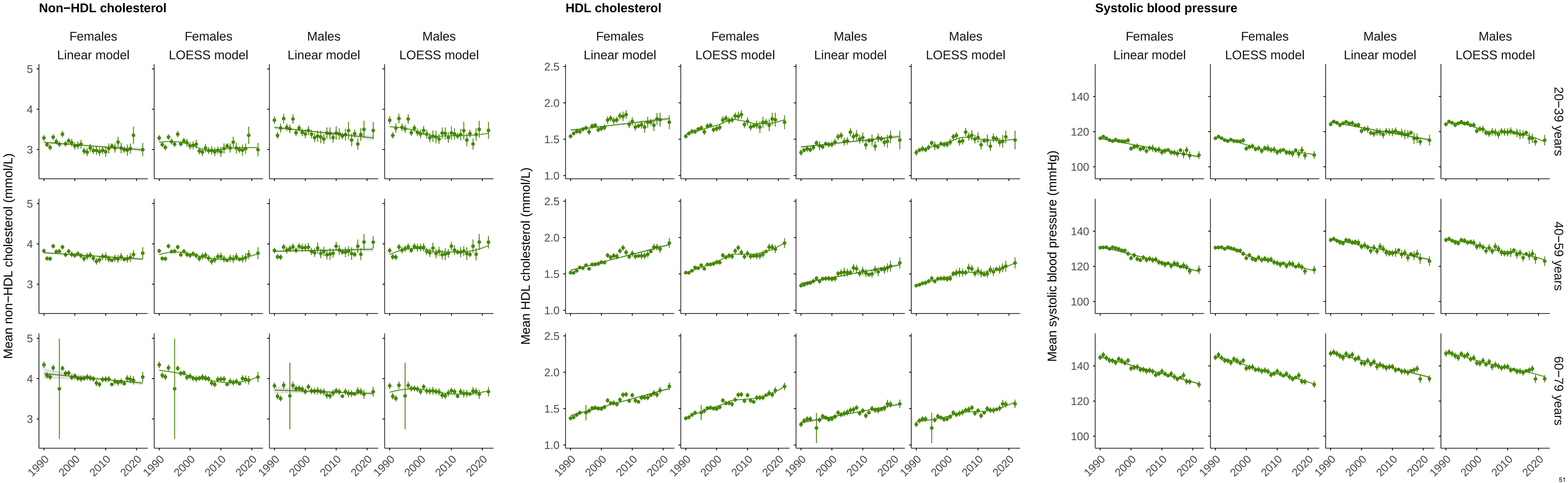

Non−HDL cholesterol
HDL cholesterol
Systolic blood pressure
Females
Linear model
Females
LOESS model
Males
Linear model
Males
LOESS model
Mean non−HDL cholesterol (mmol/L)
Mean HDL cholesterol (mmol/L)
Mean systolic blood pressure (mmHg)
20−39 years
40−59 years
60−79 years
1990
2000
2010
2020

South Korea

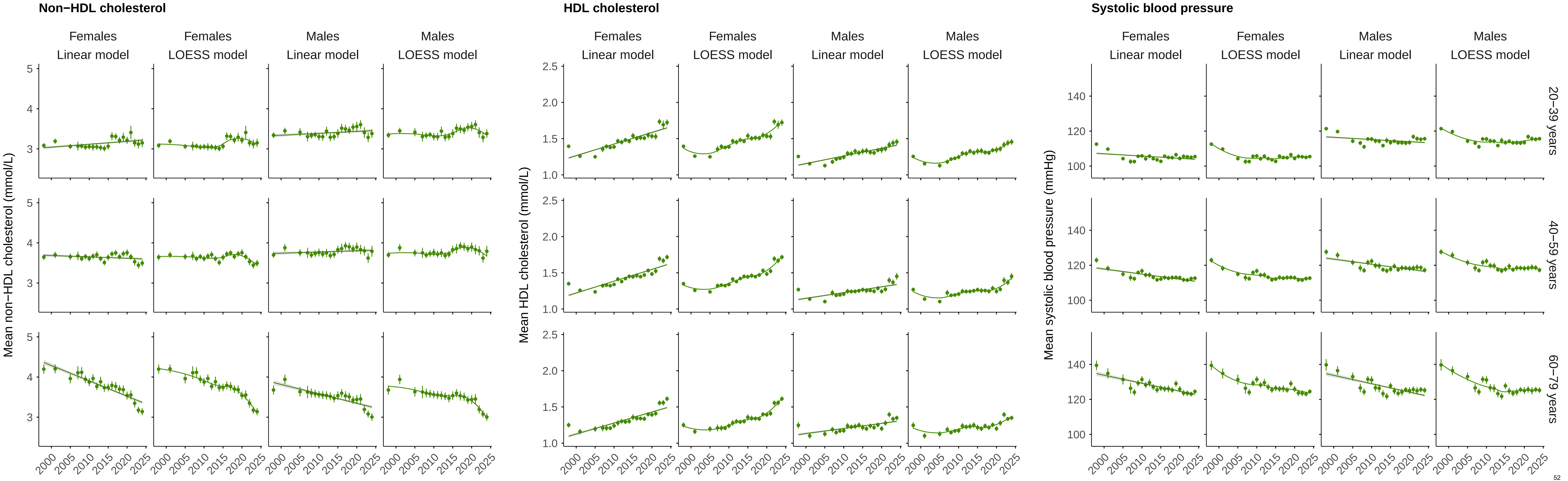

Non−HDL cholesterol
HDL cholesterol
Systolic blood pressure
Females
Linear model
Females
LOESS model
Males
Linear model
Males
LOESS model
Mean non−HDL cholesterol (mmol/L)
Mean HDL cholesterol (mmol/L)
Mean systolic blood pressure (mmHg)
20−39 years
40−59 years
60−79 years
2000
2005
2010
2015
2020
2025

Taiwan

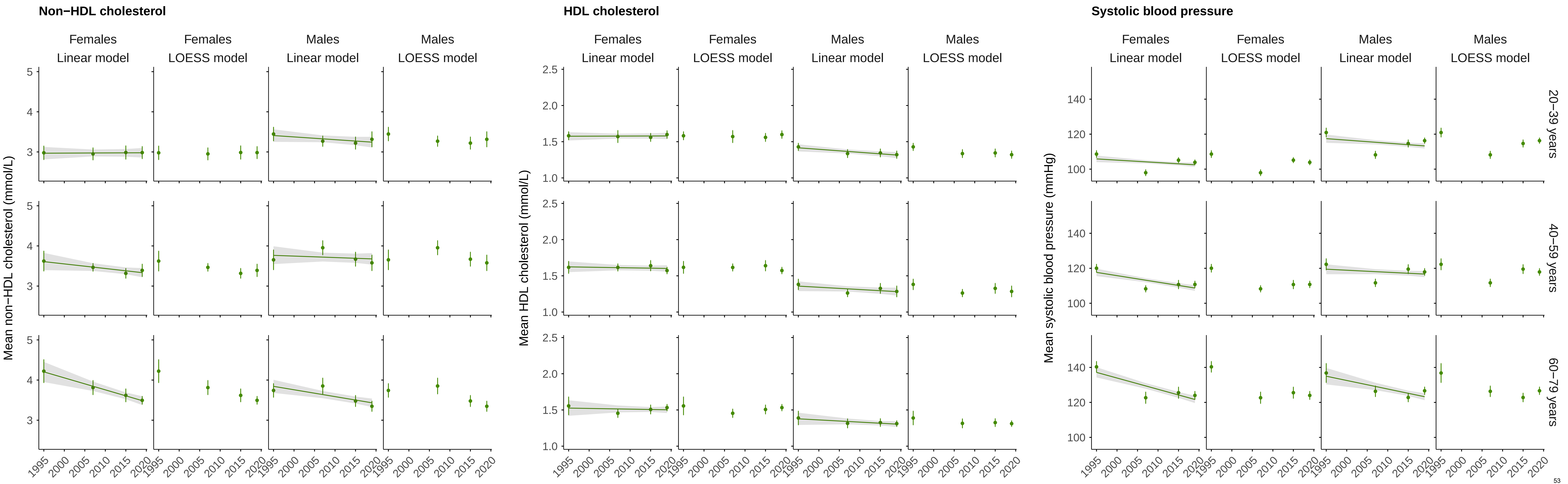

Non−HDL cholesterol
HDL cholesterol
Systolic blood pressure
Females
Linear model
Females
LOESS model
Males
Linear model
Males
LOESS model
Mean non−HDL cholesterol (mmol/L)
Mean HDL cholesterol (mmol/L)
Mean systolic blood pressure (mmHg)
20−39 years
40−59 years
60−79 years
1995
2000
2005
2010
2015
2020

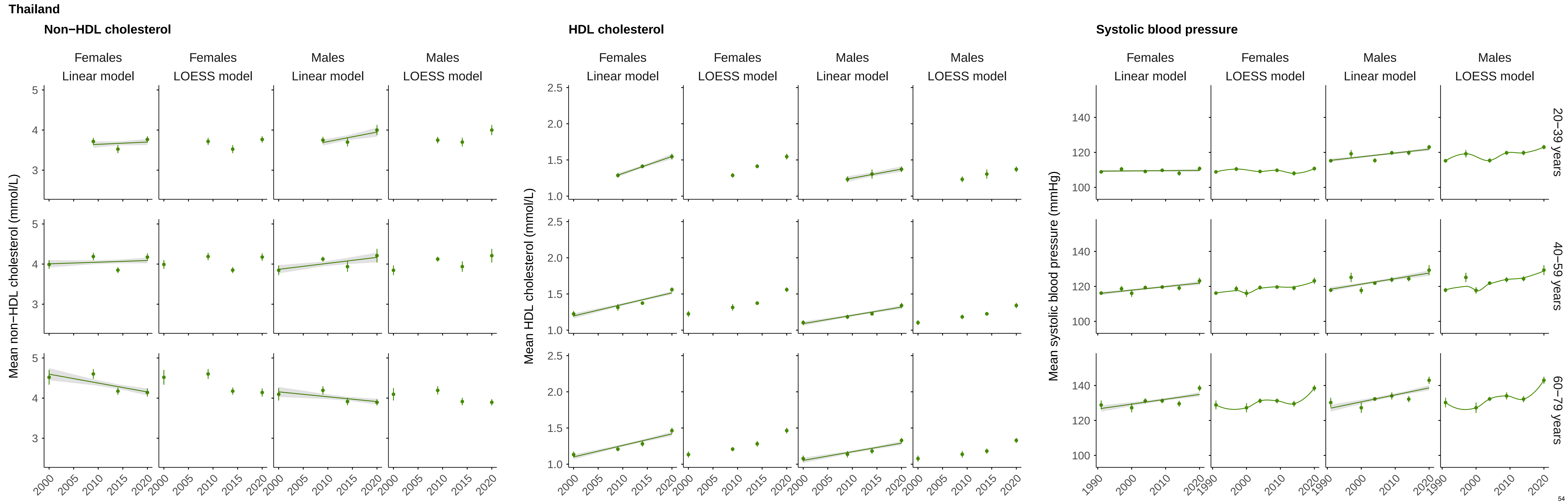

Thailand
Non−HDL cholesterol
HDL cholesterol
Systolic blood pressure
Females
Linear model
Females
LOESS model
Males
Linear model
Males
LOESS model
Mean non−HDL cholesterol (mmol/L)
Mean HDL cholesterol (mmol/L)
Mean systolic blood pressure (mmHg)
20−39 years
40−59 years
60−79 years

Finland

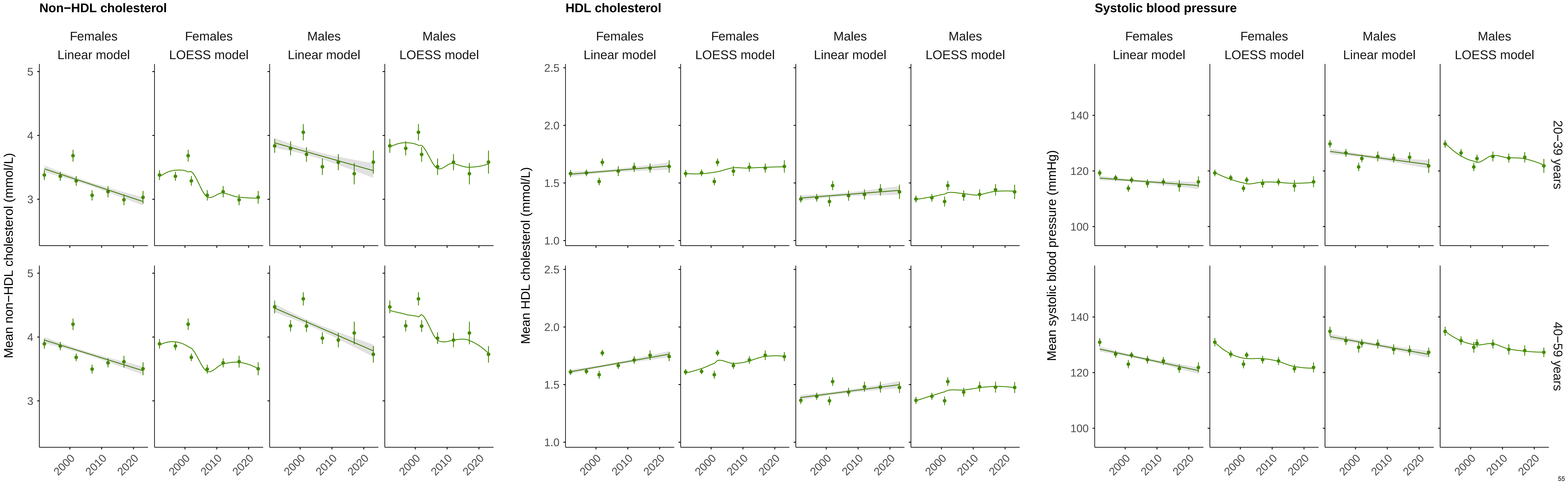

Non−HDL cholesterol
HDL cholesterol
Systolic blood pressure
Females
Linear model
Females
LOESS model
Males
Linear model
Males
LOESS model
Mean non−HDL cholesterol (mmol/L)
Mean HDL cholesterol (mmol/L)
Mean systolic blood pressure (mmHg)
20−39 years
40−59 years
2000
2010
2020

England

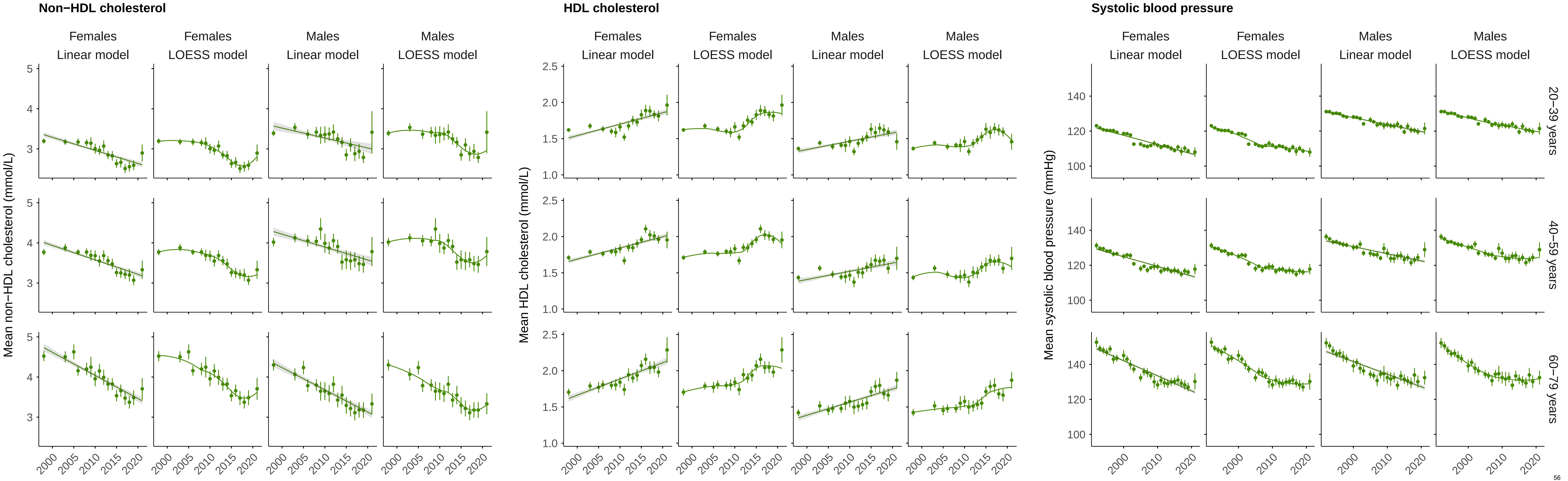
Non−HDL cholesterol
HDL cholesterol
Systolic blood pressure
Females
Males
Linear model
LOESS model
Mean non−HDL cholesterol (mmol/L)
Mean HDL cholesterol (mmol/L)
Mean systolic blood pressure (mmHg)
20−39 years
40−59 years
60−79 years

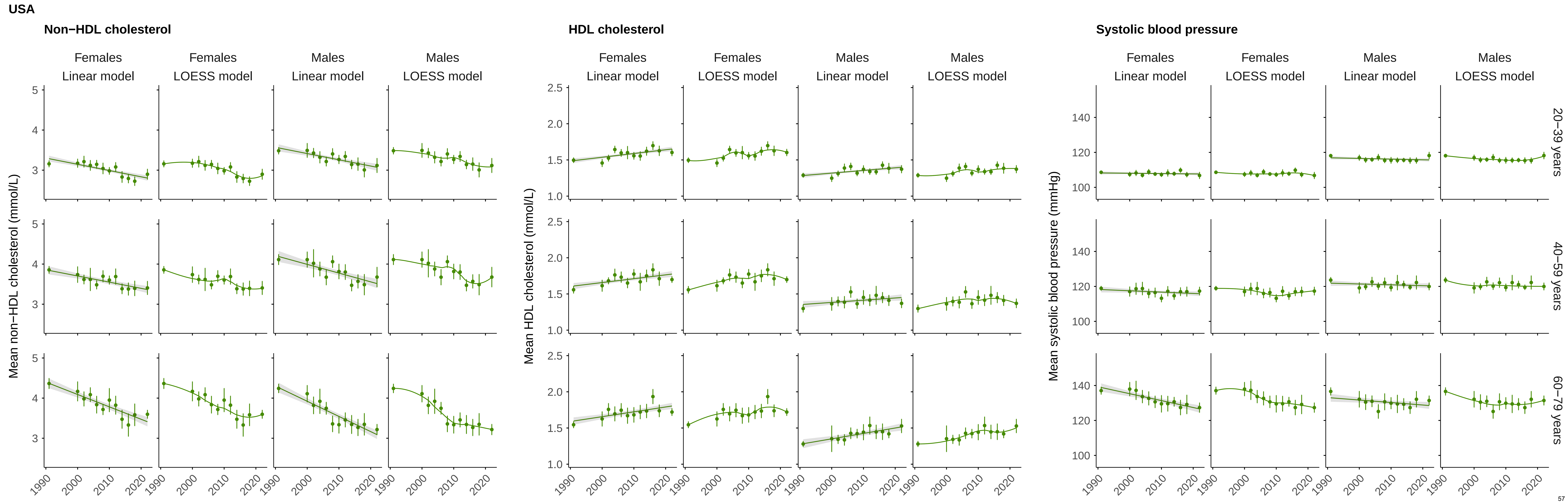
USA
Non−HDL cholesterol
HDL cholesterol
Systolic blood pressure
Females
Linear model
Females
LOESS model
Males
Linear model
Males
LOESS model
Mean non−HDL cholesterol (mmol/L)
Mean HDL cholesterol (mmol/L)
Mean systolic blood pressure (mmHg)
20−39 years
40−59 years
60−79 years
1990
2000
2010
2020

Appendix Figure 10. Trends in difference in age-standardised mean non-HDL cholesterol, mean HDL cholesterol and mean SBP between obesity and overweight and normal BMI with fitted linear and LOESS regressions.

Each point represents one sex-age-BMI group from one study, calculated as stated in Methods. Error bars show 95% confidence intervals, calculated as stated in Methods. As stated in Appendix Text 3, LOESS models were fitted to time series with five or more data points. Shaded areas show the 95% confidence intervals of the annual predictions, calculated as stated in Methods.

Age range for data from Finland is 30-59 years, grouped into 30-39 years and 40-59 years.

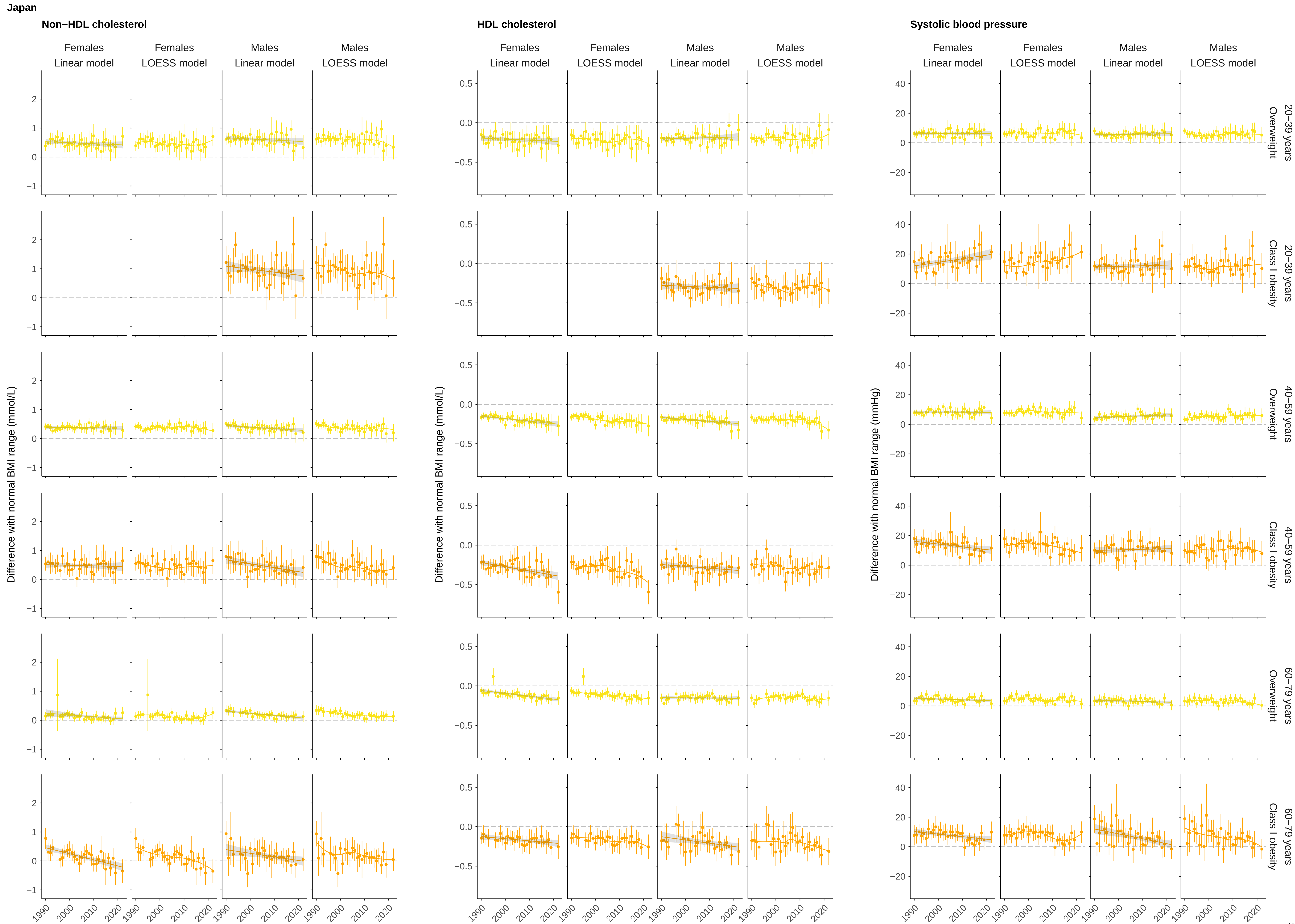
Japan
Non−HDL cholesterol
HDL cholesterol
Systolic blood pressure
Females
Linear model
Females
LOESS model
Males
Linear model
Males
LOESS model
20−39 years
Overweight
20−39 years
Class I obesity
40−59 years
Overweight
40−59 years
Class I obesity
60−79 years
Overweight
60−79 years
Class I obesity
Difference with normal BMI range (mmol/L)
Difference with normal BMI range (mmHg)
1990
2000
2010
2020

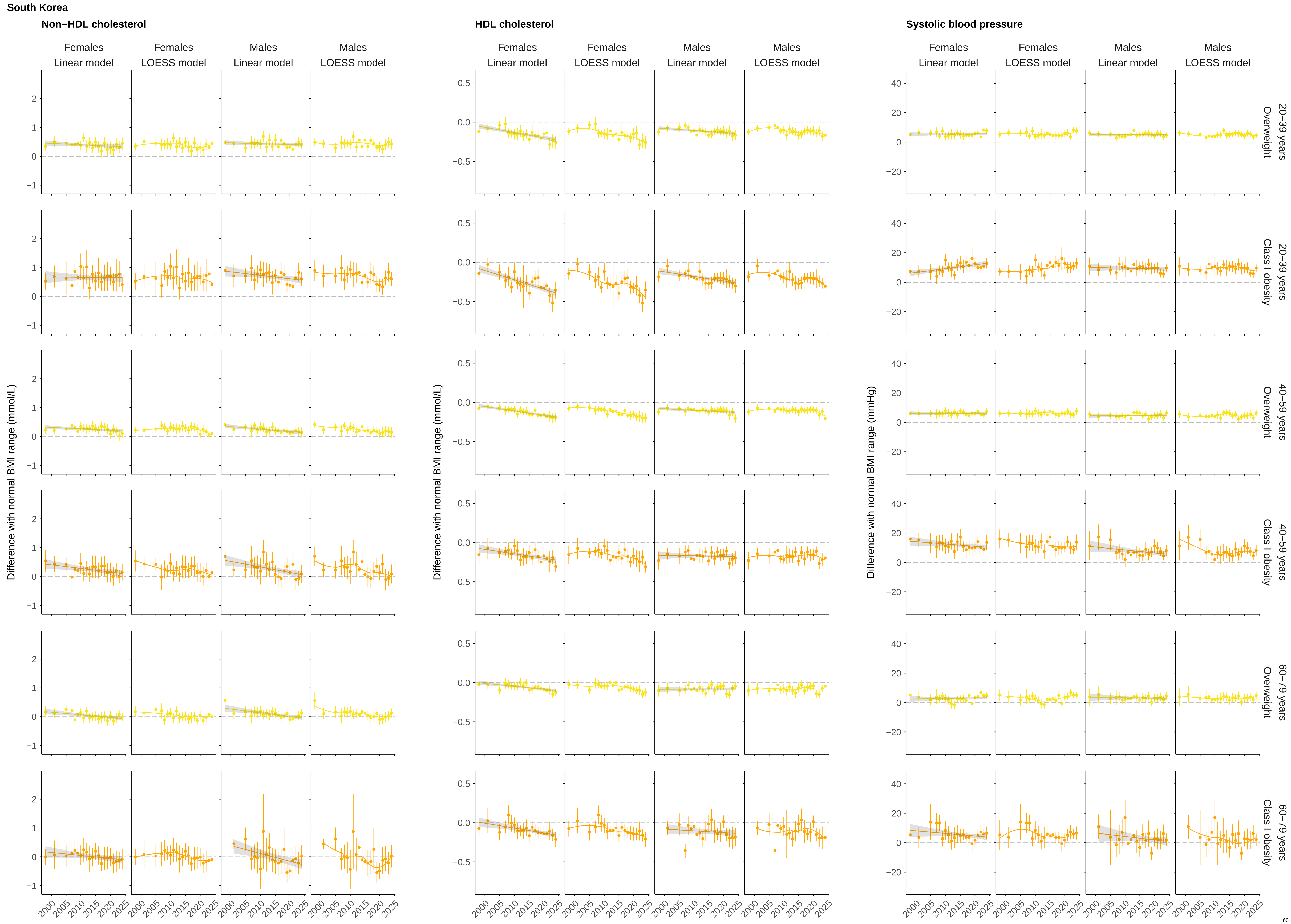

South Korea
Non−HDL cholesterol
HDL cholesterol
Systolic blood pressure
Females Linear model
Females LOESS model
Males Linear model
Males LOESS model
20–39 years Overweight
20–39 years Class I obesity
40–59 years Overweight
40–59 years Class I obesity
60–79 years Overweight
60–79 years Class I obesity
Difference with normal BMI range (mmol/L)
Difference with normal BMI range (mmHg)
2000 2005 2010 2015 2020 2025

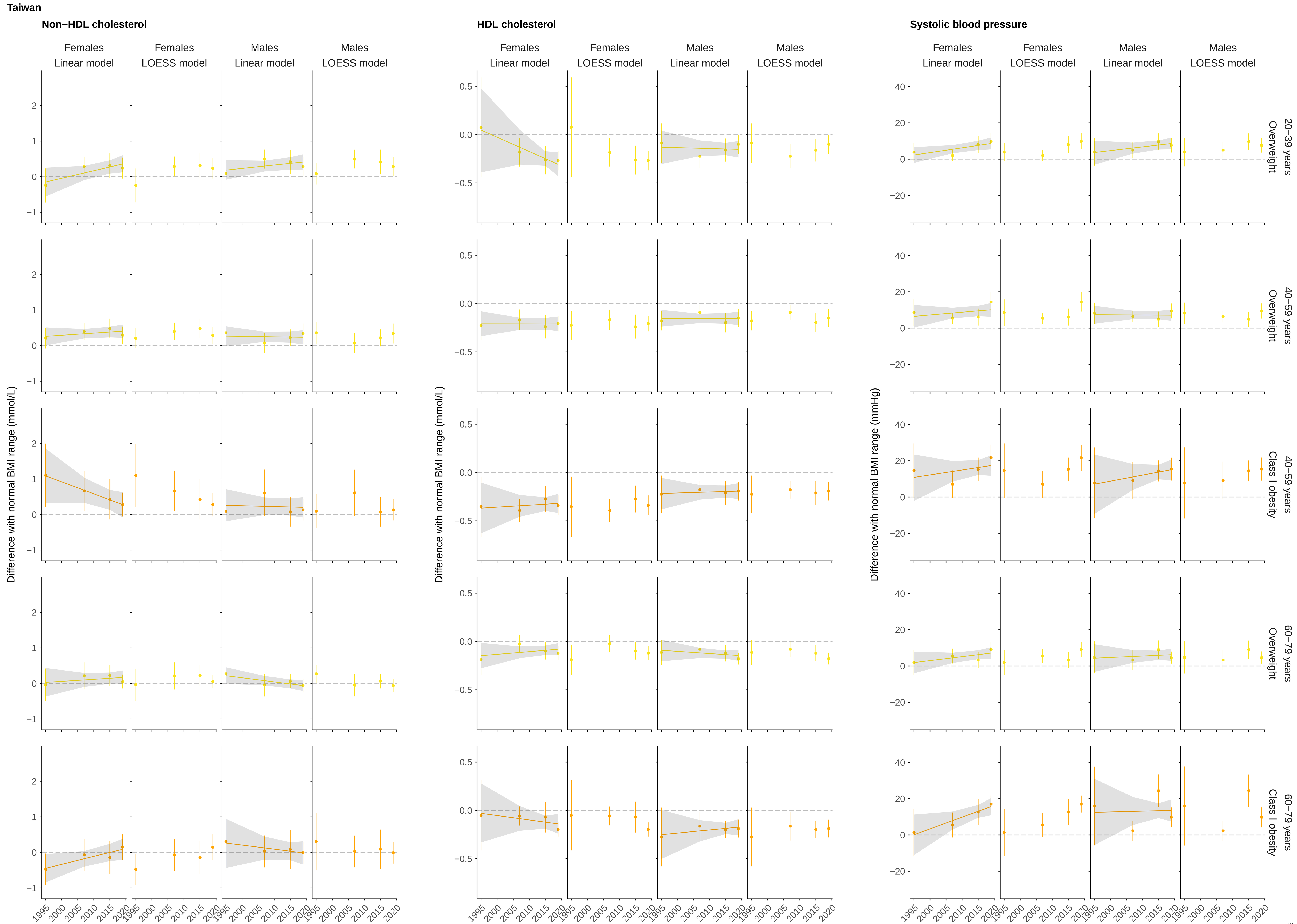

Taiwan
Non−HDL cholesterol
HDL cholesterol
Systolic blood pressure
Females Linear model
Females LOESS model
Males Linear model
Males LOESS model
Difference with normal BMI range (mmol/L)
Difference with normal BMI range (mmHg)
20−39 years Overweight
40−59 years Overweight
40−59 years Class I obesity
60−79 years Overweight
60−79 years Class I obesity
1995
2000
2005
2010
2015
2020

Thailand

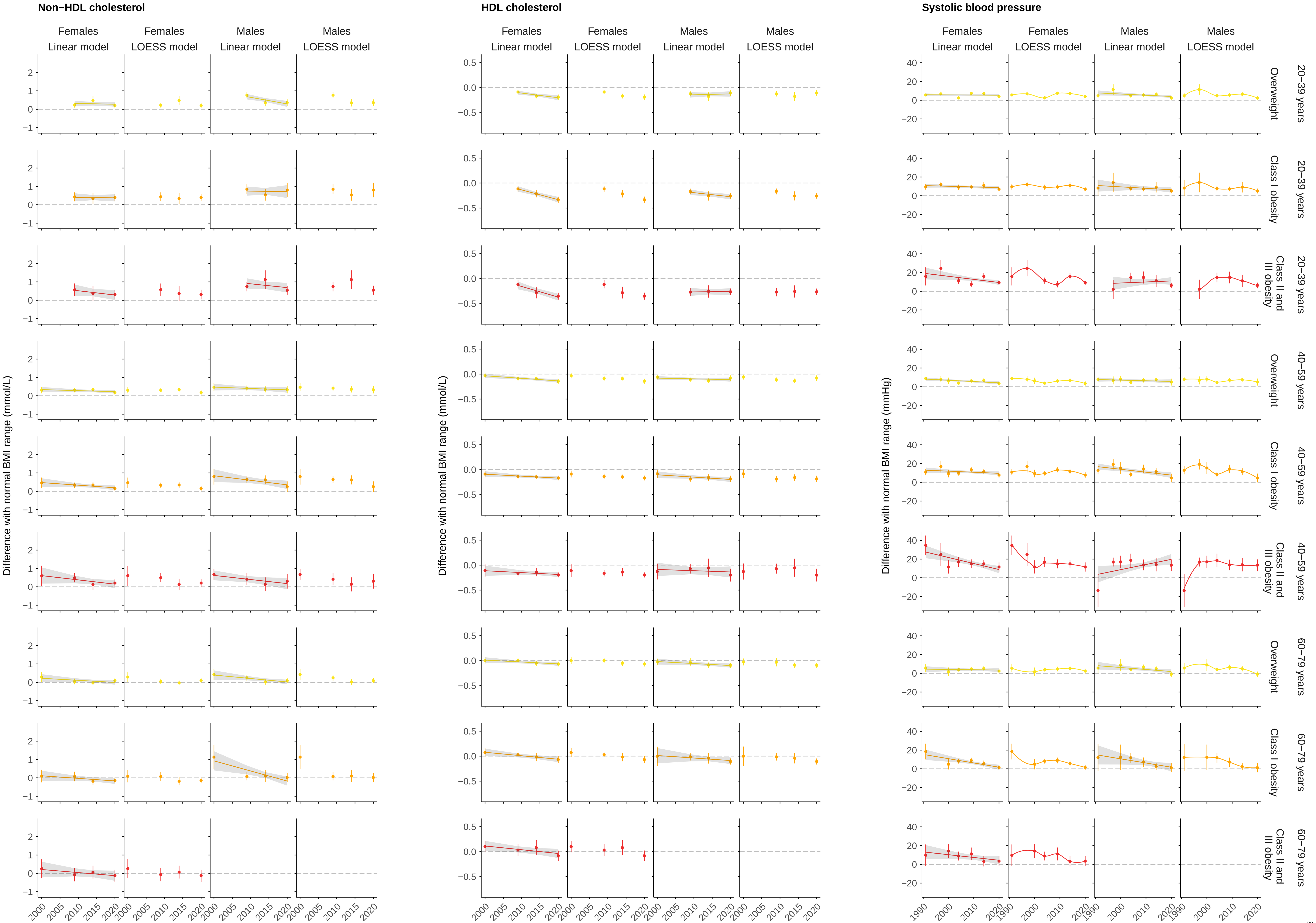
Non−HDL cholesterol
HDL cholesterol
Systolic blood pressure
Females
Linear model
Females
LOESS model
Males
Linear model
Males
LOESS model
Difference with normal BMI range (mmol/L)
Difference with normal BMI range (mmHg)
20−39 years
40−59 years
60−79 years
Overweight
Class I obesity
Class II and III obesity

Finland

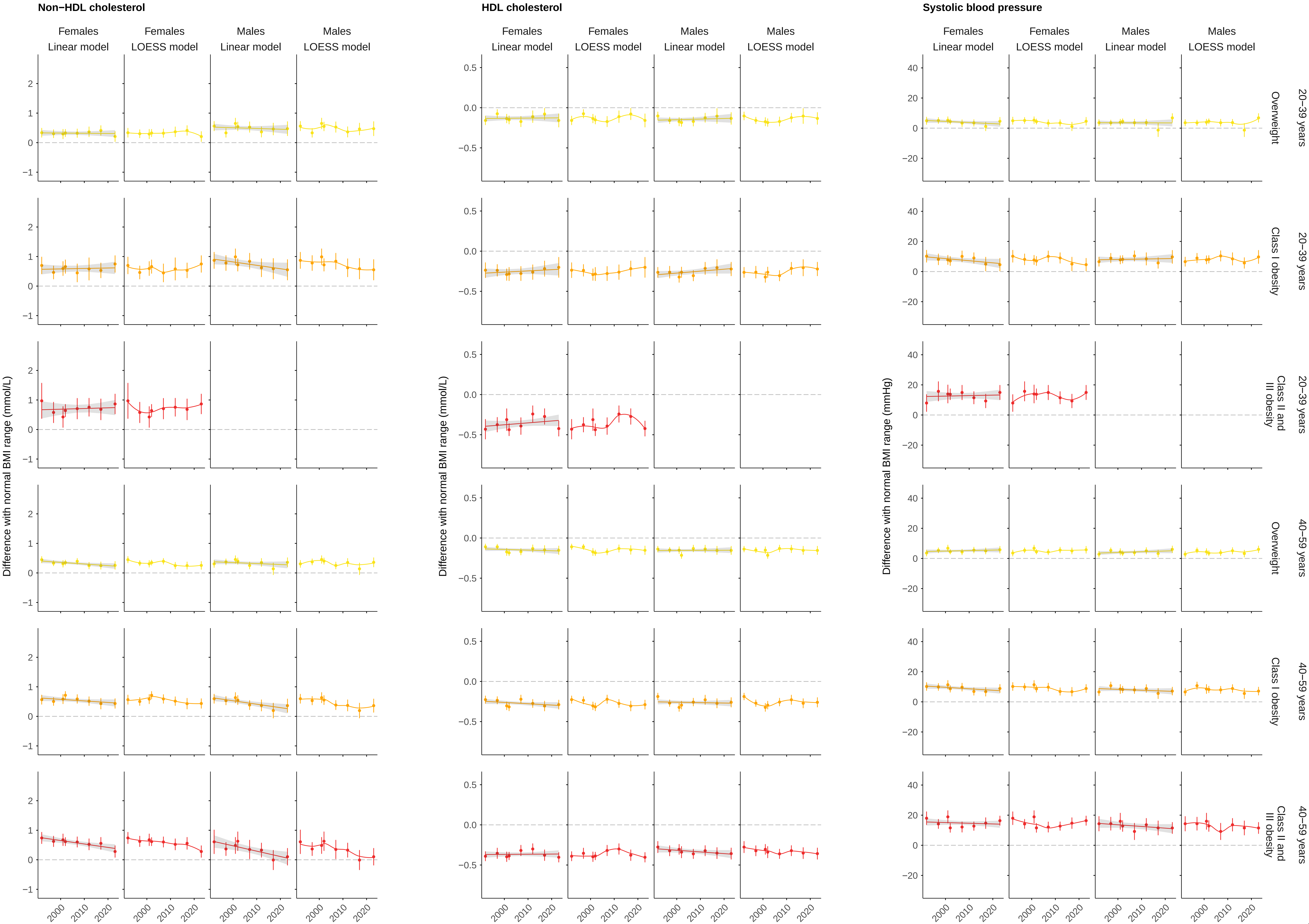

Non−HDL cholesterol
HDL cholesterol
Systolic blood pressure
Females
Linear model
Females
LOESS model
Males
Linear model
Males
LOESS model
Difference with normal BMI range (mmol/L)
Difference with normal BMI range (mmHg)
20−39 years
Overweight
Class I obesity
Class II and III obesity
40−59 years
2000
2010
2020

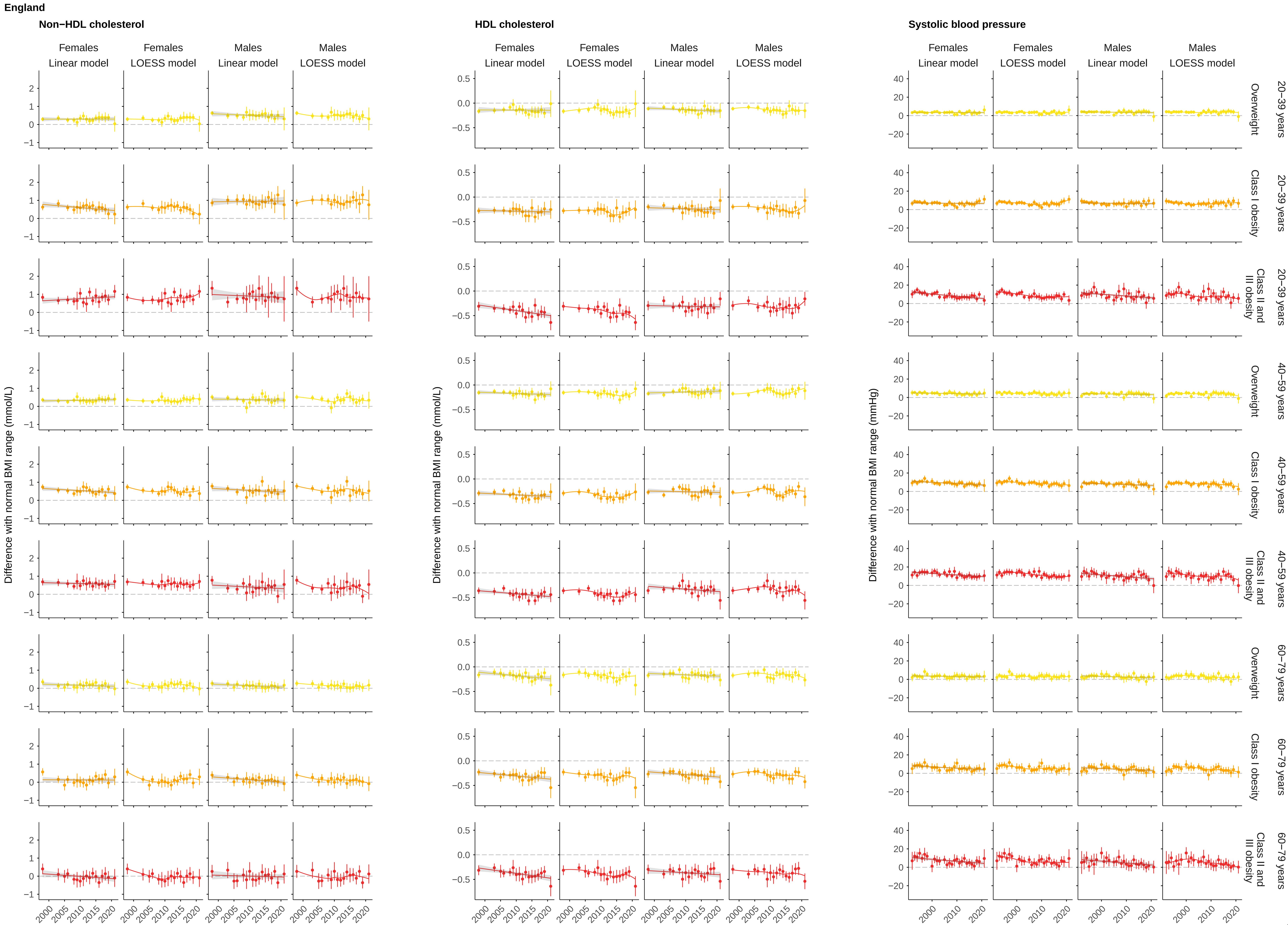
England
Non−HDL cholesterol
HDL cholesterol
Systolic blood pressure
Females
Linear model
Females
LOESS model
Males
Linear model
Males
LOESS model
Overweight
Class I obesity
Class II and III obesity
20−39 years
40−59 years
60−79 years
Difference with normal BMI range (mmol/L)
Difference with normal BMI range (mmHg)
2000
2005
2010
2015
2020

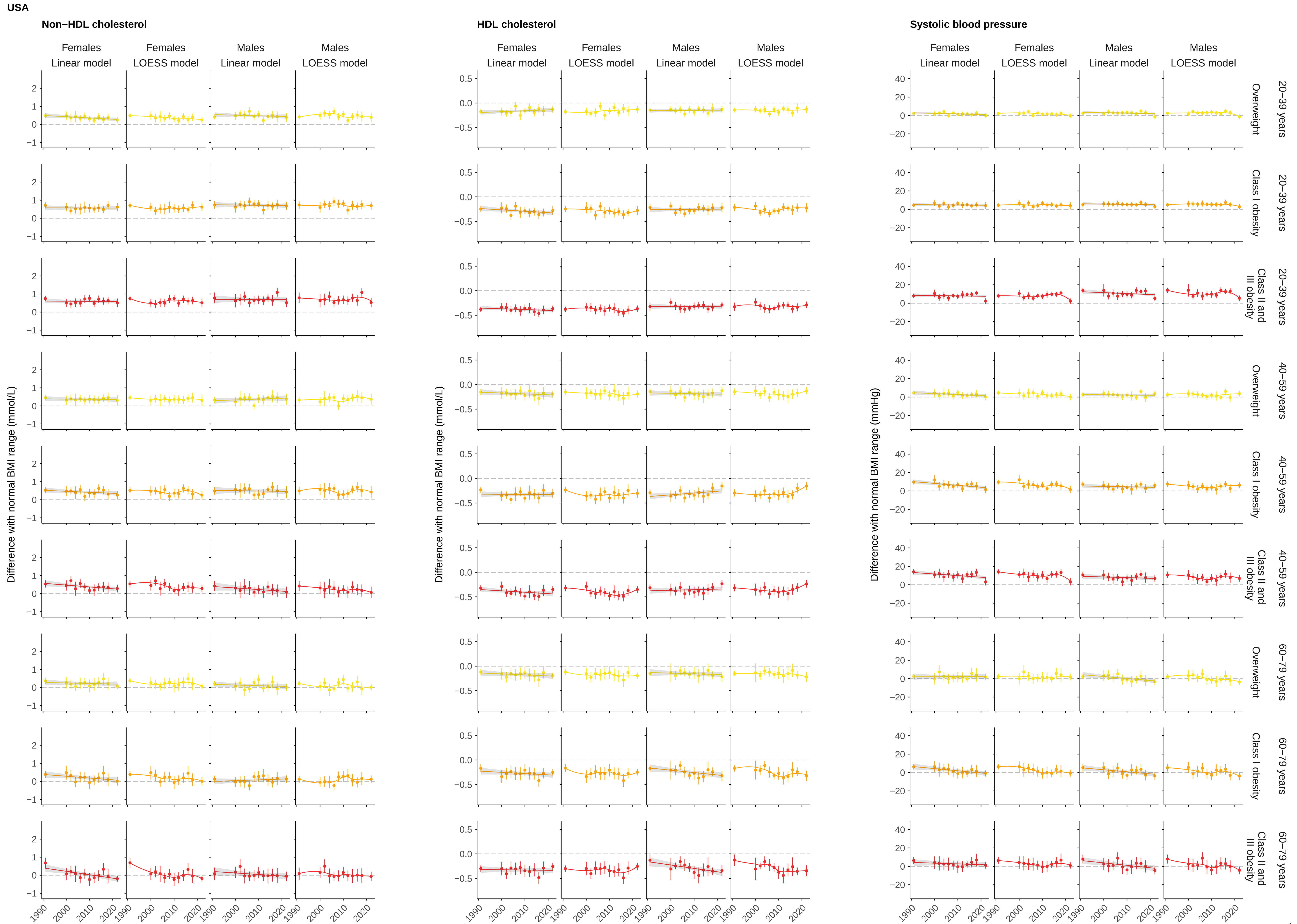

USA
Non−HDL cholesterol
HDL cholesterol
Systolic blood pressure
Females
Linear model
Females
LOESS model
Males
Linear model
Males
LOESS model
Difference with normal BMI range (mmol/L)
Difference with normal BMI range (mmHg)
Overweight
Class I obesity
Class II and III obesity
20−39 years
40−59 years
60−79 years

Appendix Figure 11. Trends in age-standardised proportion of participants using lipid-lowering or antihypertensive medicines in normal BMI with fitted linear and LOESS regressions.

Each point represents one sex-age-BMI range group from one study, calculated as stated in Methods. Error bars show 95% confidence intervals, calculated as stated in Methods. As stated in Appendix Text 3, LOESS models were fitted to time series with five or more data points. Shaded areas show the 95% confidence intervals of the annual predictions, calculated as stated in Methods.

Age range for data from Finland is 30-59 years, grouped into 30-39 years and 40-59 years.

Japan

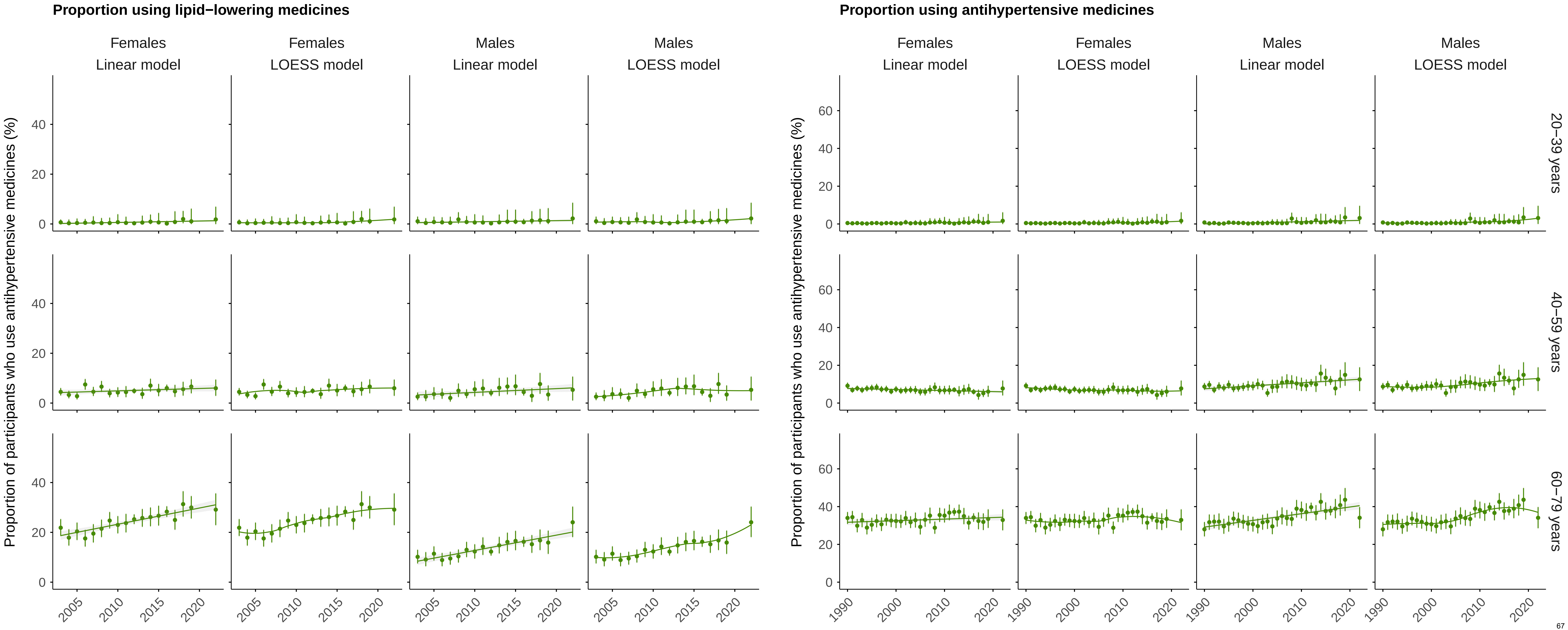

Proportion using lipid−lowering medicines
Proportion using antihypertensive medicines
Females
Linear model
Females
LOESS model
Males
Linear model
Males
LOESS model
20–39 years
40–59 years
60–79 years
Proportion of participants who use antihypertensive medicines (%)
0
20
40
60
2005
2010
2015
2020
1990
2000
2010
2020

South Korea

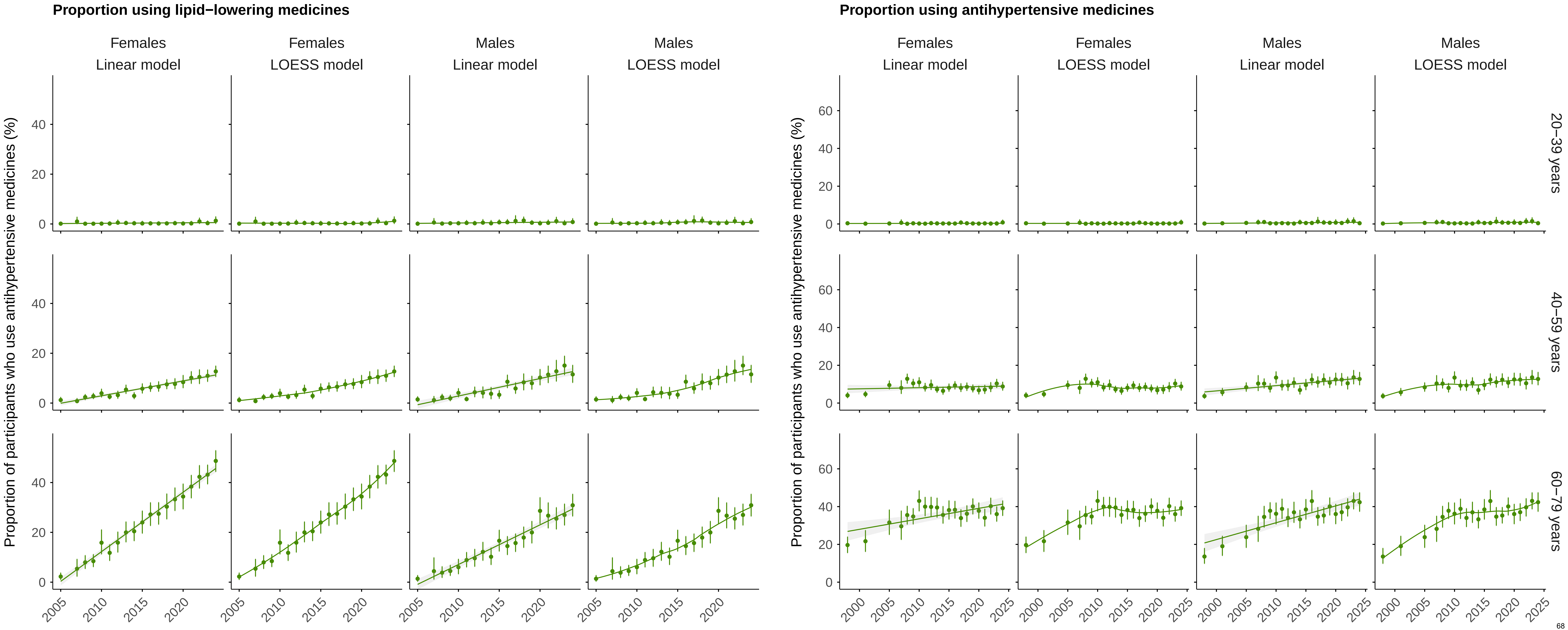
Proportion using lipid−lowering medicines
Proportion using antihypertensive medicines
Females
Linear model
Females
LOESS model
Males
Linear model
Males
LOESS model
20−39 years
40−59 years
60−79 years
Proportion of participants who use antihypertensive medicines (%)
0
20
40
60
2000
2005
2010
2015
2020
2025

Taiwan

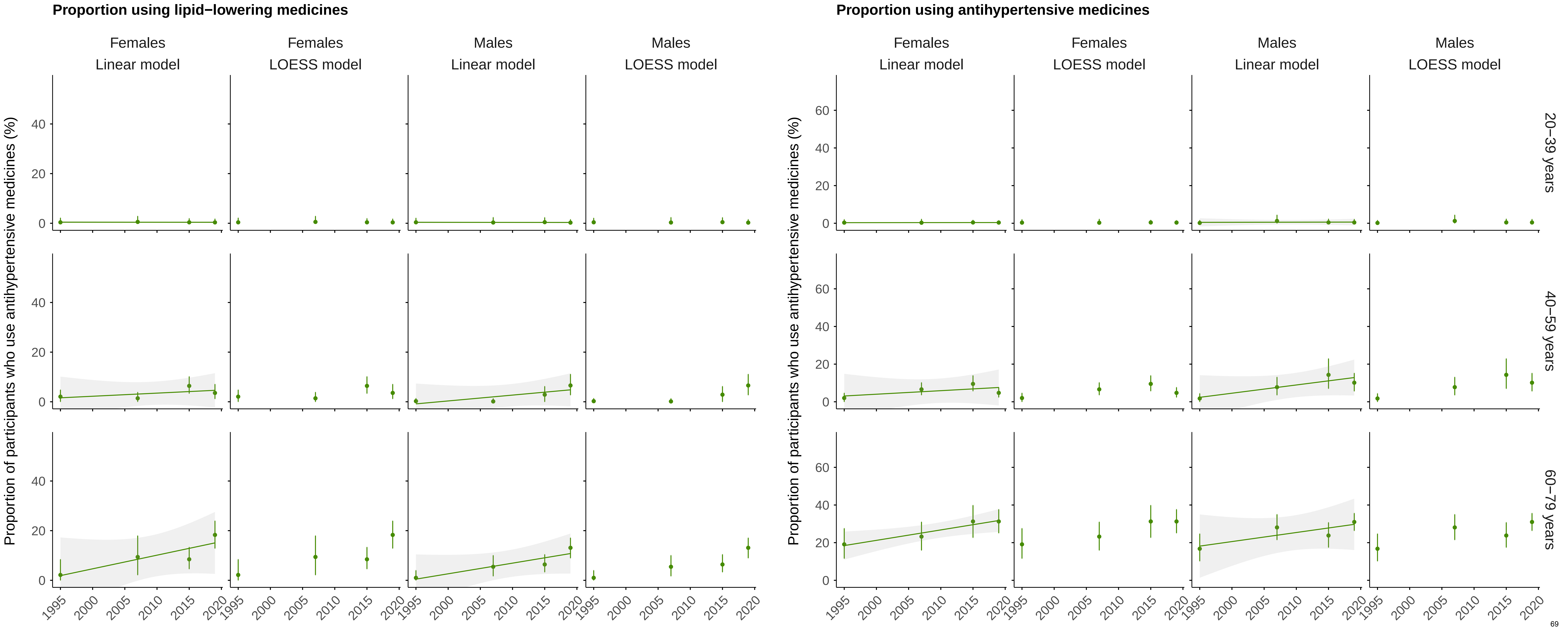
Proportion using lipid−lowering medicines
Proportion using antihypertensive medicines
Females
Linear model
Females
LOESS model
Males
Linear model
Males
LOESS model
Proportion of participants who use antihypertensive medicines (%)
20−39 years
40−59 years
60−79 years
0
20
40
60
1995
2000
2005
2010
2015
2020

Thailand

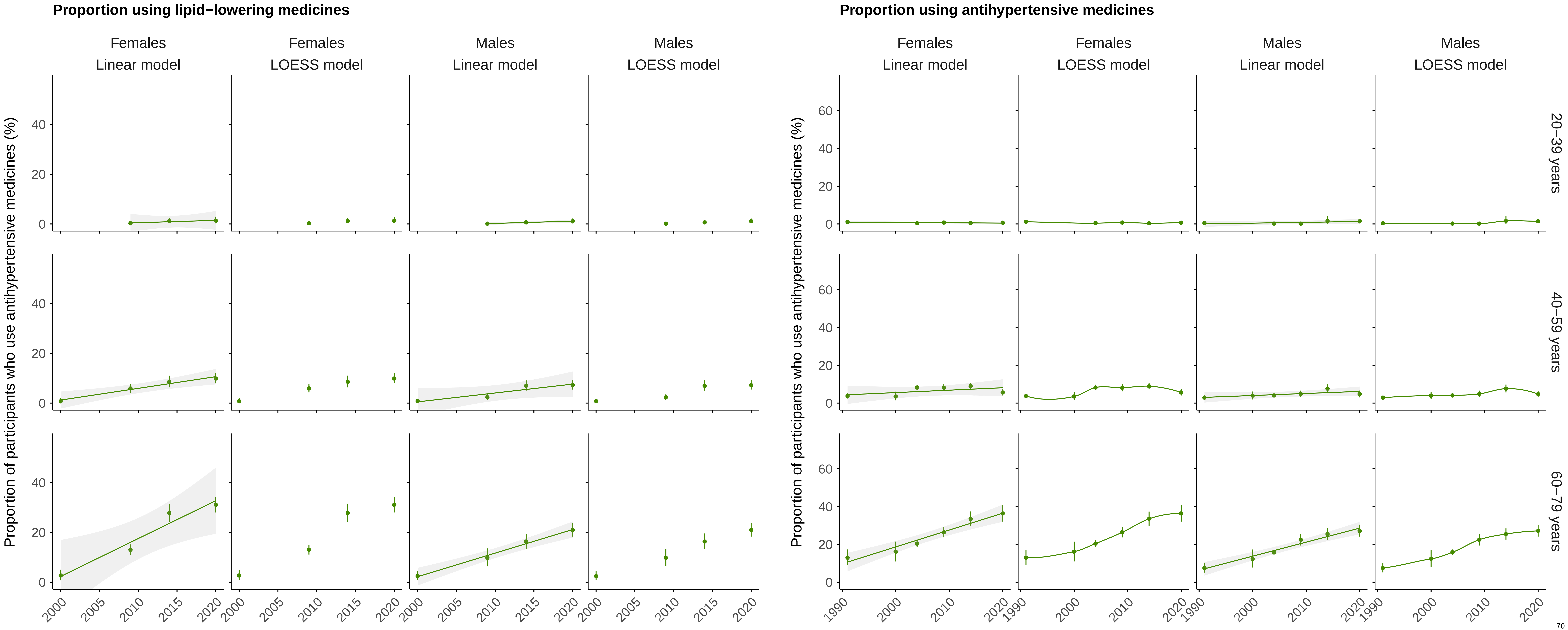

Proportion using lipid−lowering medicines
Proportion using antihypertensive medicines
Females
Linear model
Females
LOESS model
Males
Linear model
Males
LOESS model
Proportion of participants who use antihypertensive medicines (%)
20−39 years
40−59 years
60−79 years
0
20
40
60
2000
2005
2010
2015
2020
1990

Finland

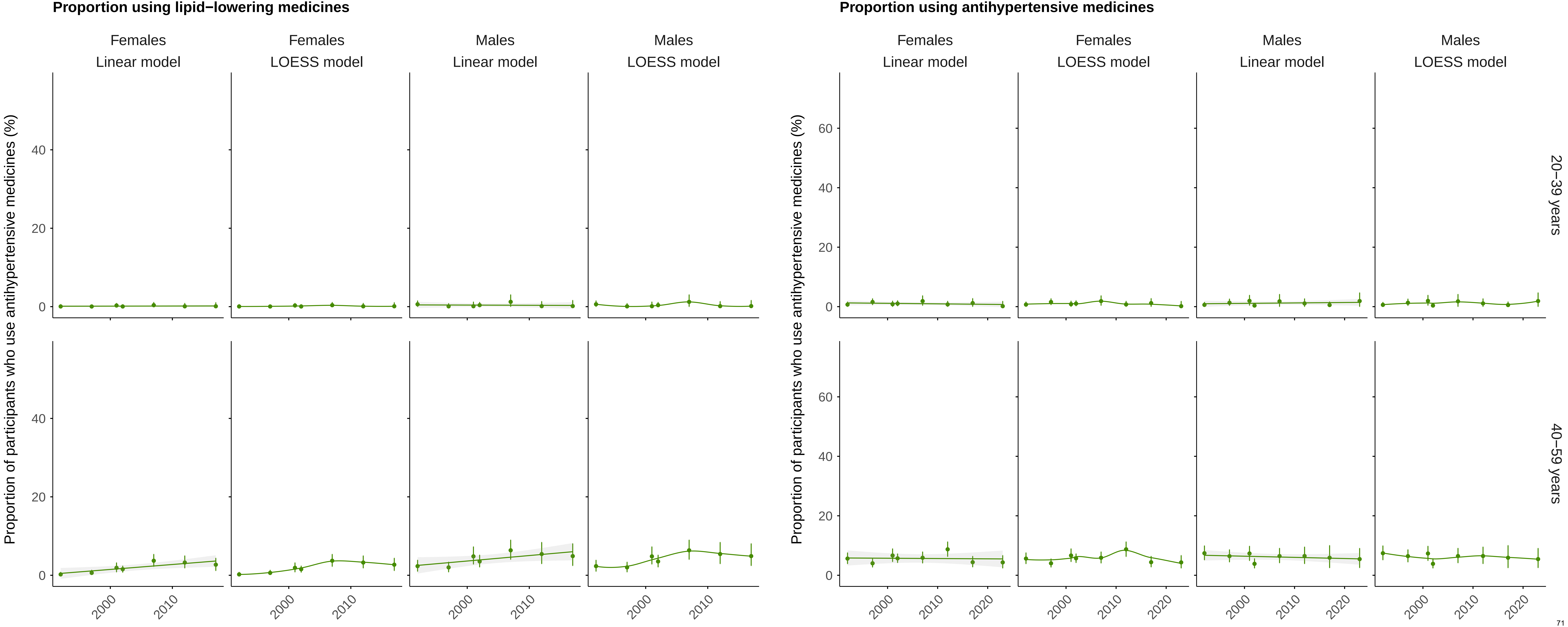
Proportion using lipid−lowering medicines
Proportion using antihypertensive medicines
Females
Linear model
Females
LOESS model
Males
Linear model
Males
LOESS model
Proportion of participants who use antihypertensive medicines (%)
20−39 years
40−59 years
0
20
40
60
2000
2010
2020

England

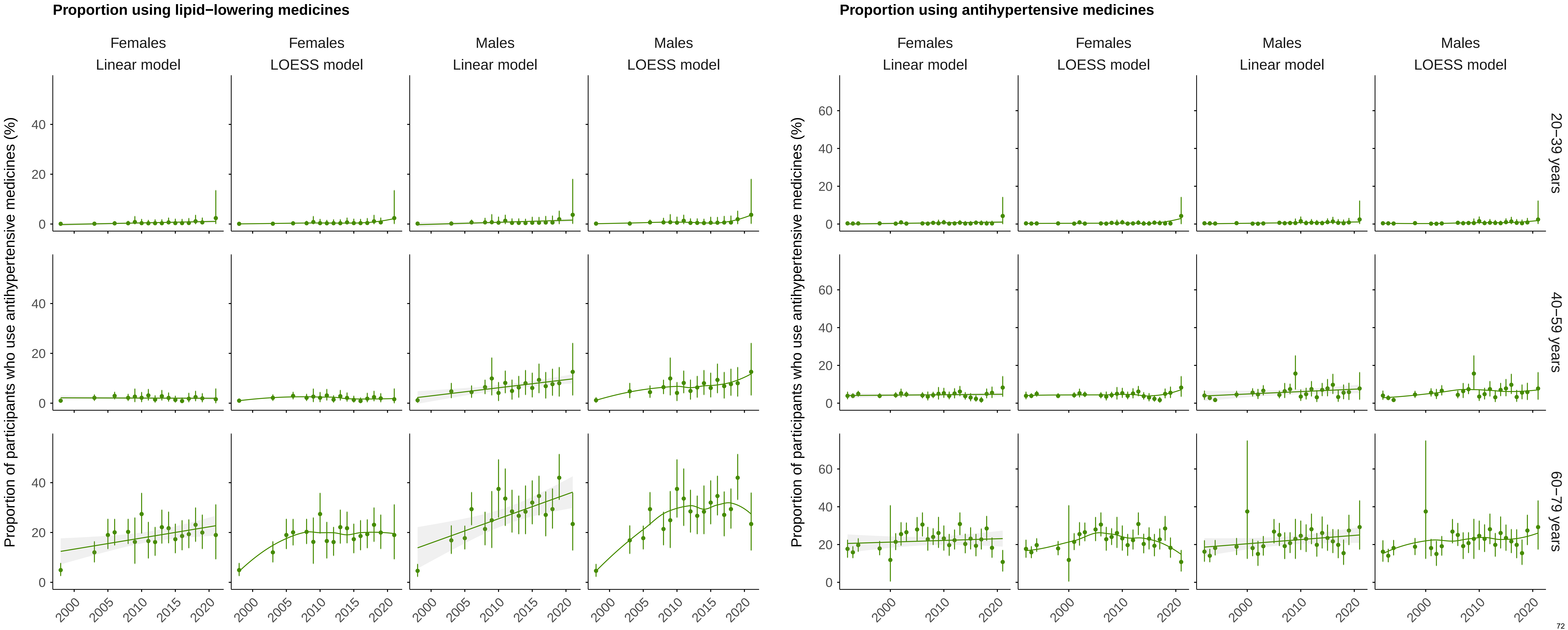
Proportion using lipid−lowering medicines
Proportion using antihypertensive medicines
Females
Linear model
Females
LOESS model
Males
Linear model
Males
LOESS model
20−39 years
40−59 years
60−79 years
Proportion of participants who use antihypertensive medicines (%)
0
20
40
60
2000
2005
2010
2015
2020

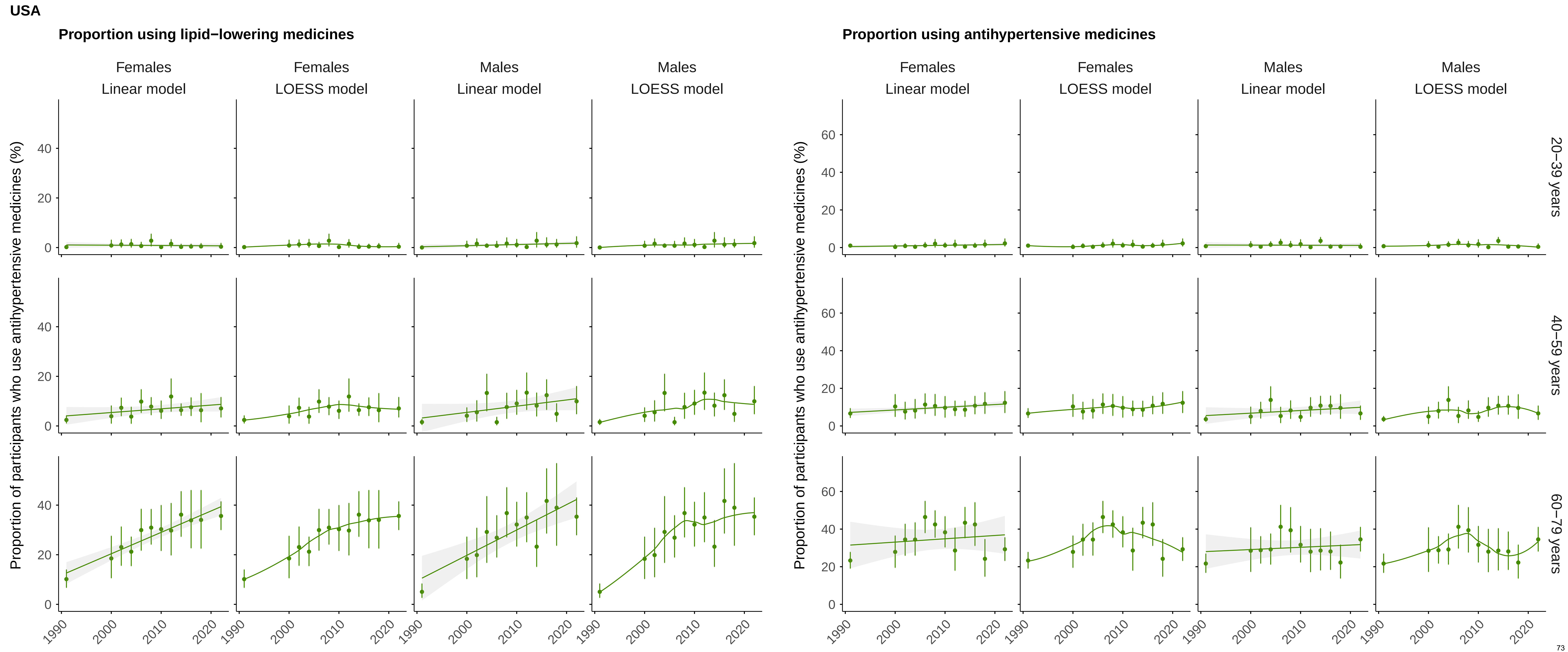

USA
Proportion using lipid−lowering medicines
Proportion using antihypertensive medicines
Females
Linear model
Females
LOESS model
Males
Linear model
Males
LOESS model
Proportion of participants who use antihypertensive medicines (%)
20−39 years
40−59 years
60−79 years
0
20
40
60
1990
2000
2010
2020

Appendix Figure 12. Trends in difference in age-standardised proportion of participants using lipid-lowering or antihypertensive medicines between obesity and overweight and normal BMI with fitted linear and LOESS regressions.

Each point represents one sex-age-BMI group from one study, calculated as stated in Methods. Error bars show 95% confidence intervals, calculated as stated in Methods. As stated in Appendix Text 3, LOESS models were fitted to time series with five or more data points. Shaded areas show the 95% confidence intervals of the annual predictions, calculated as stated in Methods.

Age range for data from Finland is 30-59 years, grouped into 30-39 years and 40-59 years.

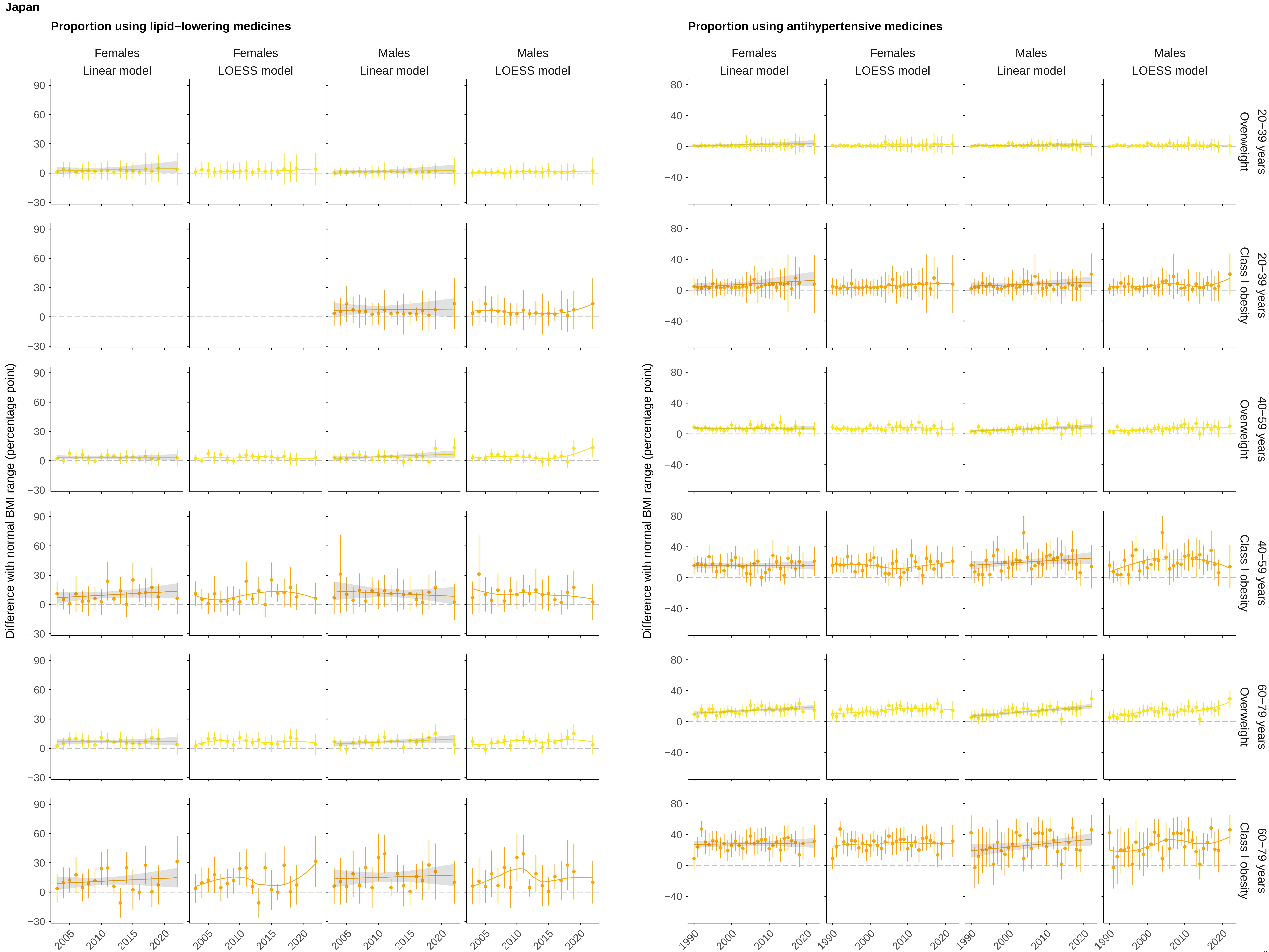
Japan
Proportion using lipid−lowering medicines
Proportion using antihypertensive medicines
Females
Linear model
Females
LOESS model
Males
Linear model
Males
LOESS model
20−39 years
Overweight
20−39 years
Class I obesity
40−59 years
Overweight
40−59 years
Class I obesity
60−79 years
Overweight
60−79 years
Class I obesity
Difference with normal BMI range (percentage point)
90
60
30
0
−30
80
40
0
−40
2005
2010
2015
2020
1990
2000
2010
2020

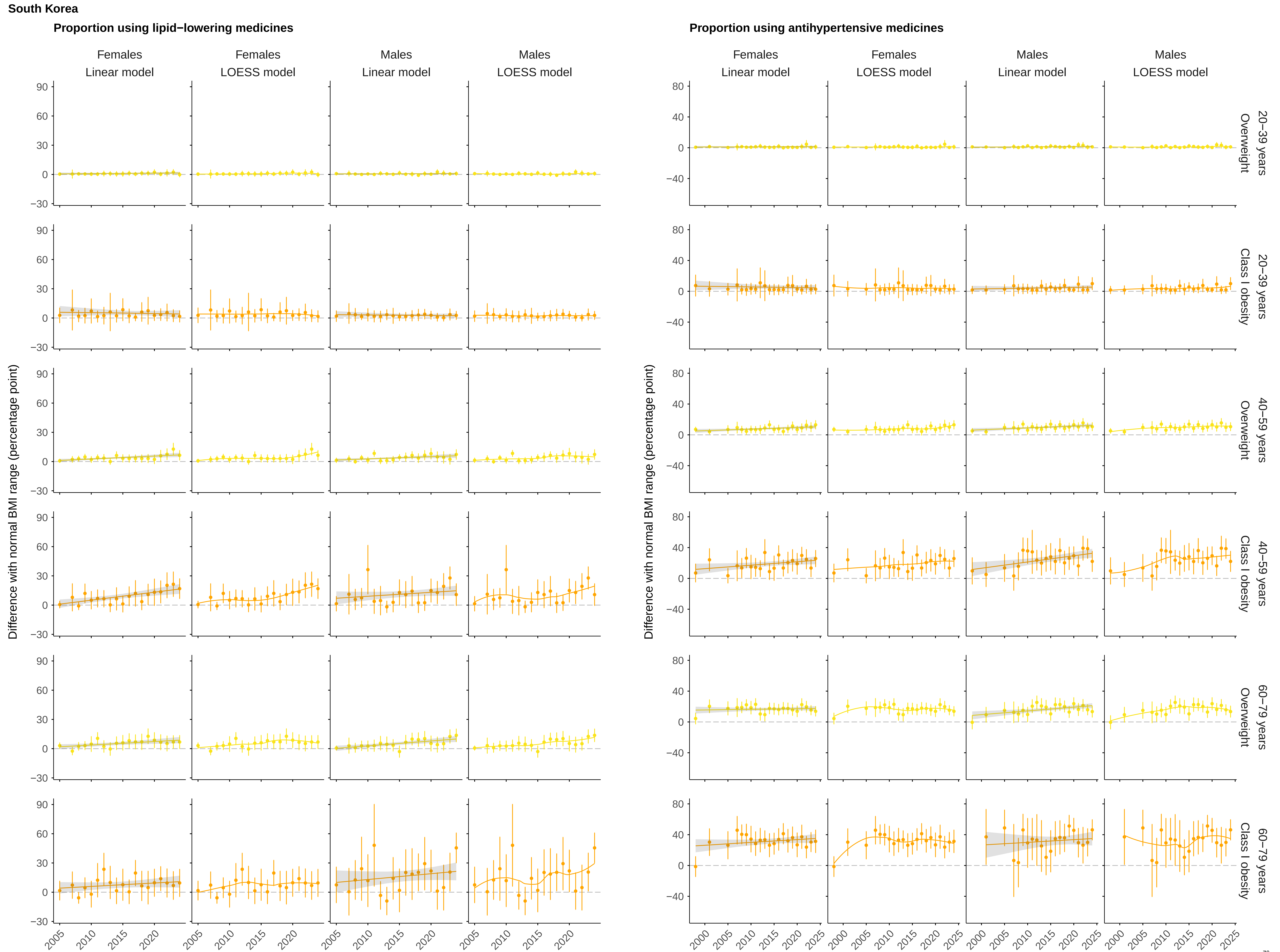

South Korea
Proportion using lipid−lowering medicines
Proportion using antihypertensive medicines
Females
Linear model
Females
LOESS model
Males
Linear model
Males
LOESS model
Difference with normal BMI range (percentage point)
20−39 years
Overweight
20−39 years
Class I obesity
40−59 years
Overweight
40−59 years
Class I obesity
60−79 years
Overweight
60−79 years
Class I obesity

Taiwan

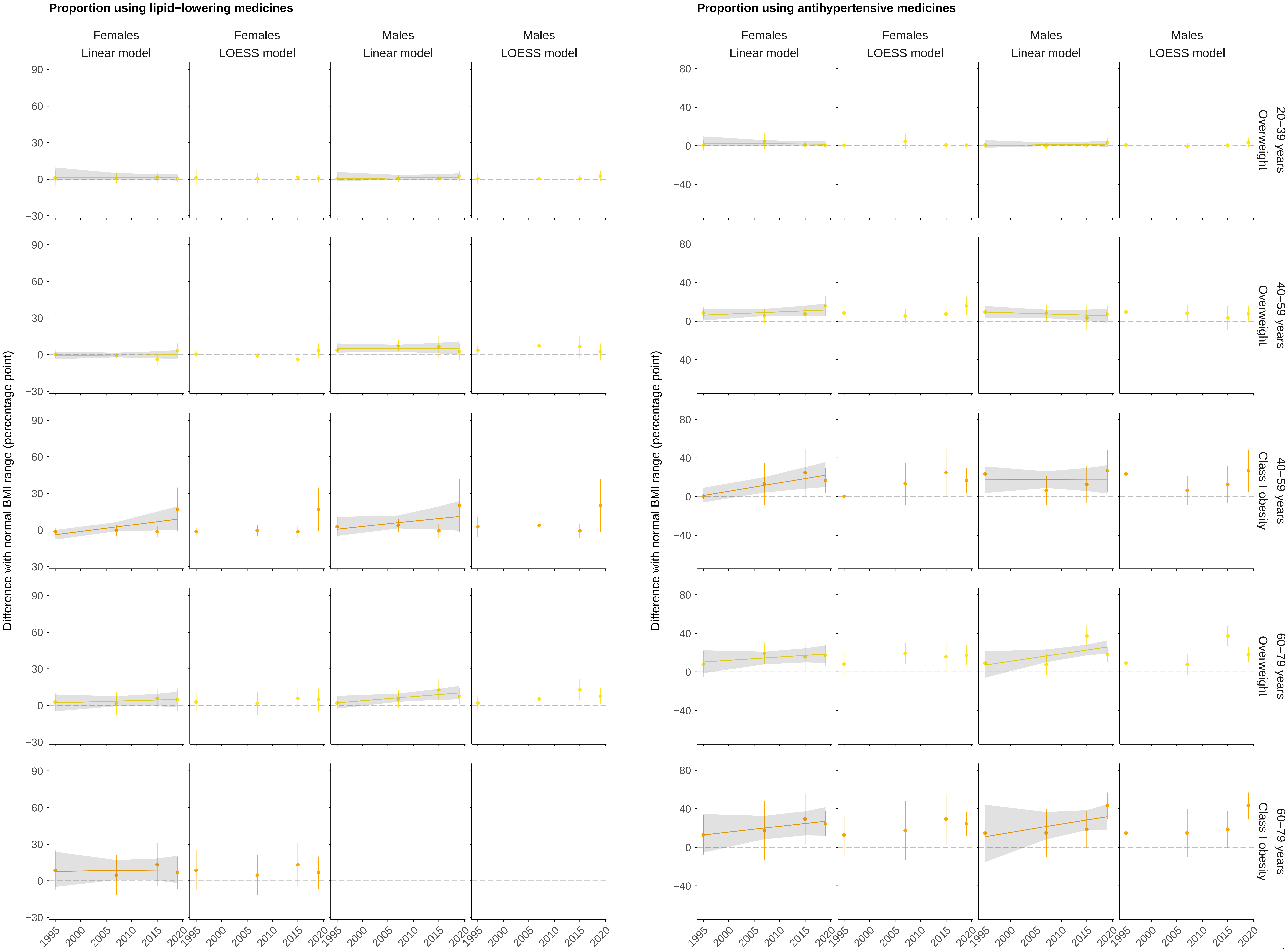

Proportion using lipid−lowering medicines
Proportion using antihypertensive medicines
Females
Linear model
Females
LOESS model
Males
Linear model
Males
LOESS model
Difference with normal BMI range (percentage point)
20−39 years
Overweight
40−59 years
Overweight
40−59 years
Class I obesity
60−79 years
Overweight
60−79 years
Class I obesity
90
60
30
0
−30
80
40
0
−40
1995
2000
2005
2010
2015
2020

Thailand

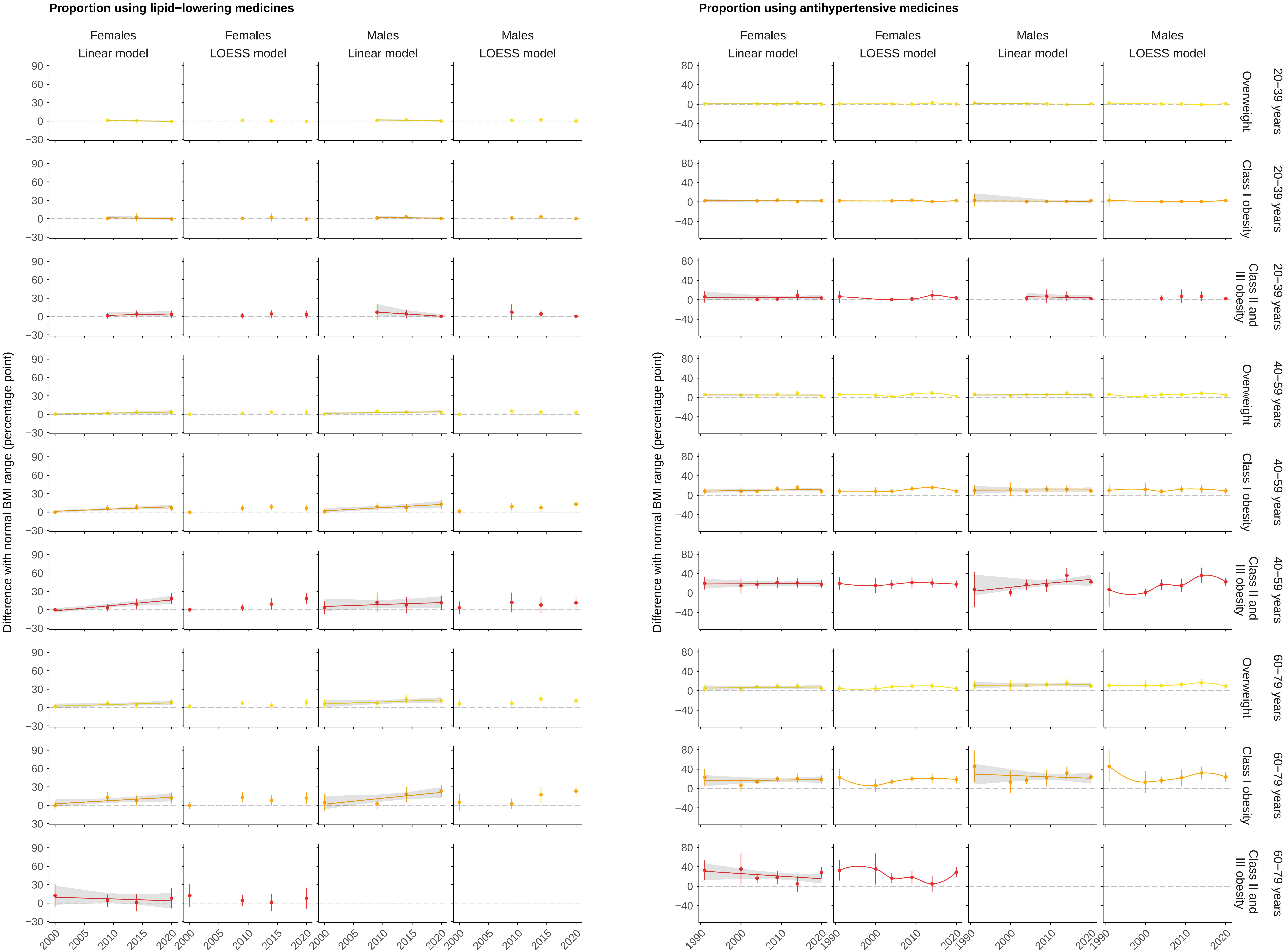
Proportion using lipid−lowering medicines
Proportion using antihypertensive medicines
Females
Linear model
Females
LOESS model
Males
Linear model
Males
LOESS model
Difference with normal BMI range (percentage point)
20−39 years
40−59 years
60−79 years
Overweight
Class I obesity
Class II and III obesity
90
60
30
0
−30
80
40
0
−40
2000
2005
2010
2015
2020
1990
2000
2010
2020

Finland

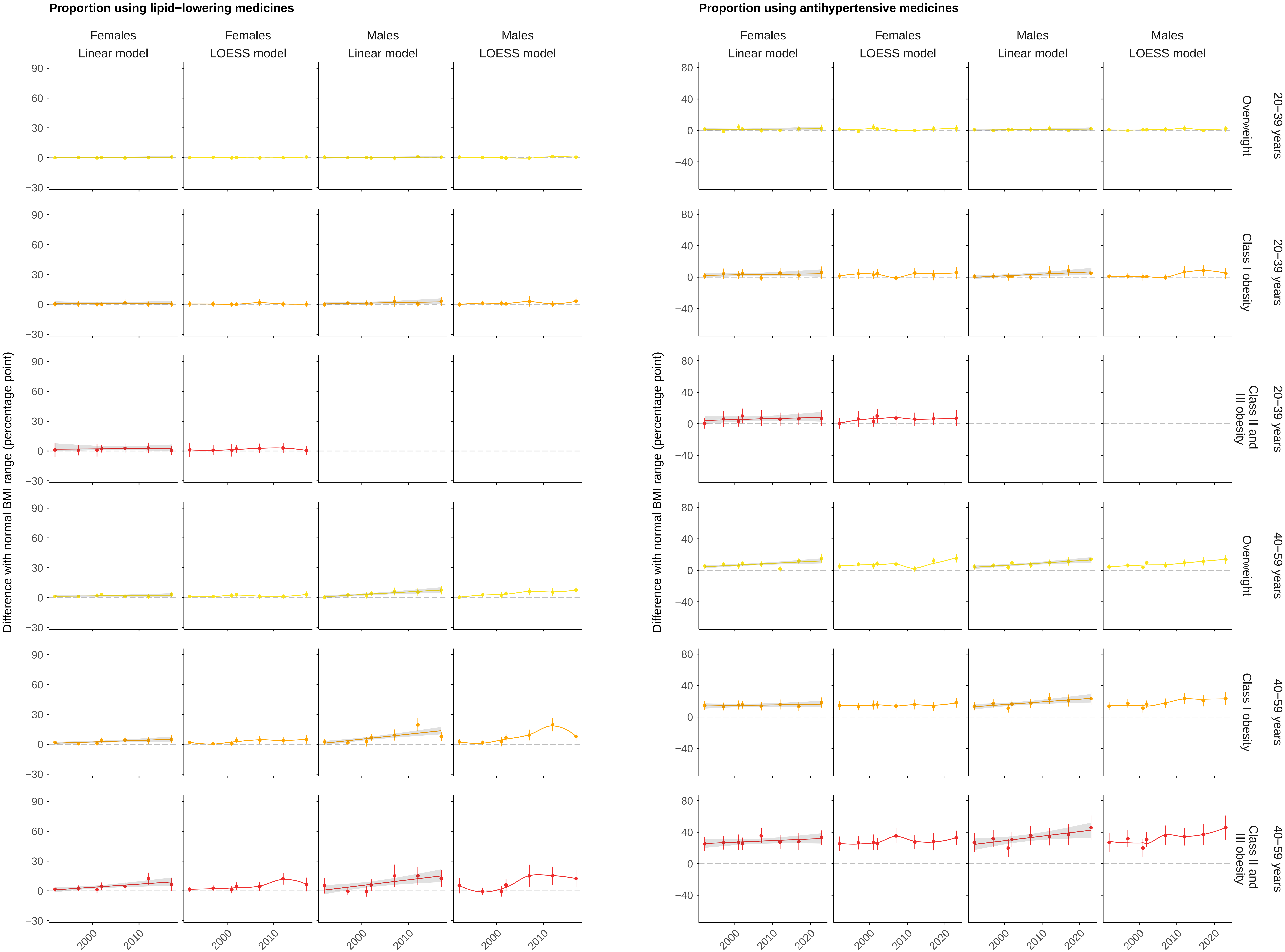
Proportion using lipid−lowering medicines
Proportion using antihypertensive medicines
Females
Linear model
Females
LOESS model
Males
Linear model
Males
LOESS model
Difference with normal BMI range (percentage point)
90
60
30
0
−30
80
40
0
−40
2000
2010
2020
20−39 years
Overweight
20−39 years
Class I obesity
20−39 years
Class II and III obesity
40−59 years
Overweight
40−59 years
Class I obesity
40−59 years
Class II and III obesity

England

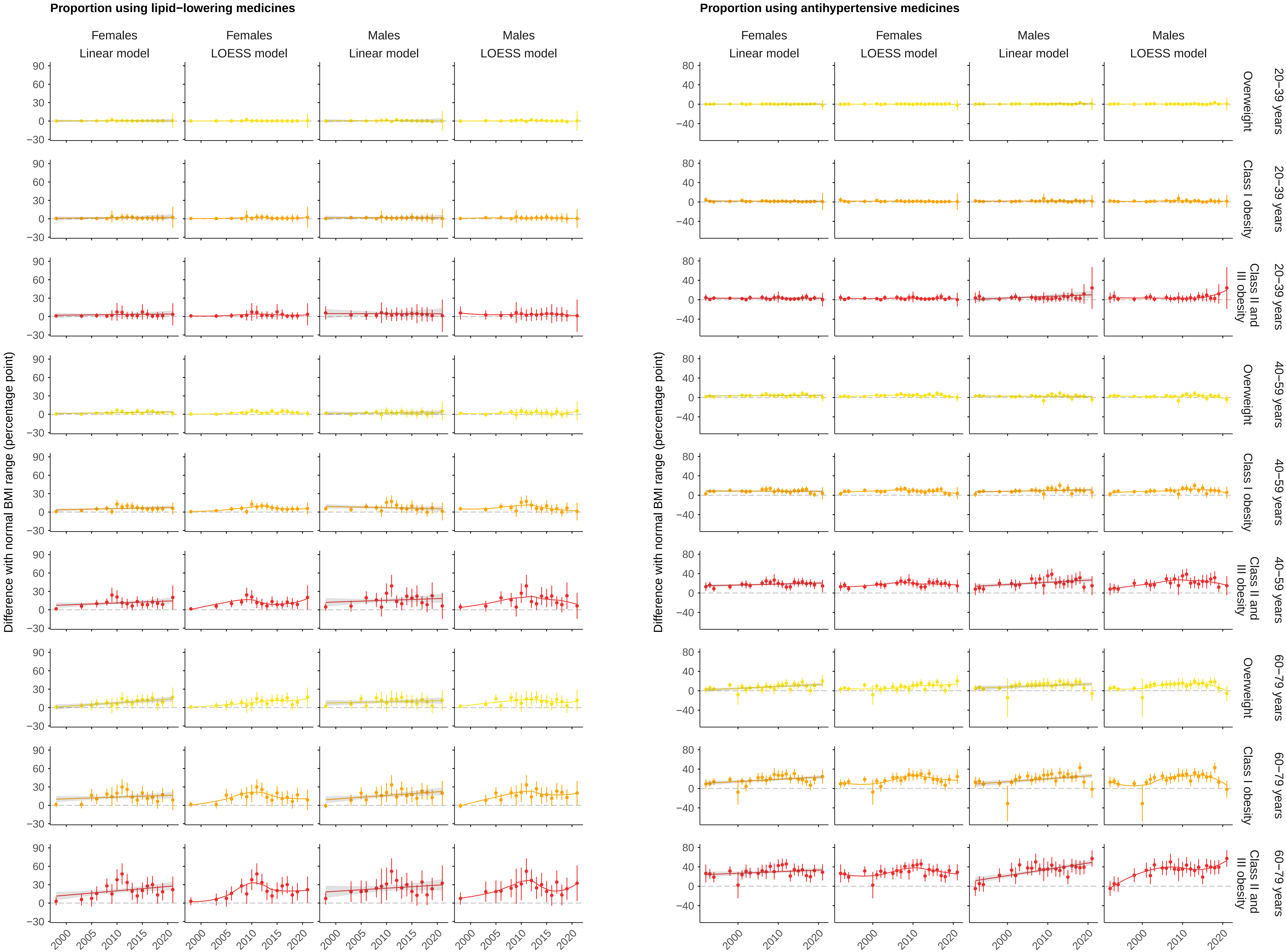

Proportion using lipid−lowering medicines
Proportion using antihypertensive medicines
Females Linear model
Females LOESS model
Males Linear model
Males LOESS model
Difference with normal BMI range (percentage point)
Overweight
Class I obesity
Class II and III obesity
20−39 years
40−59 years
60−79 years

USA

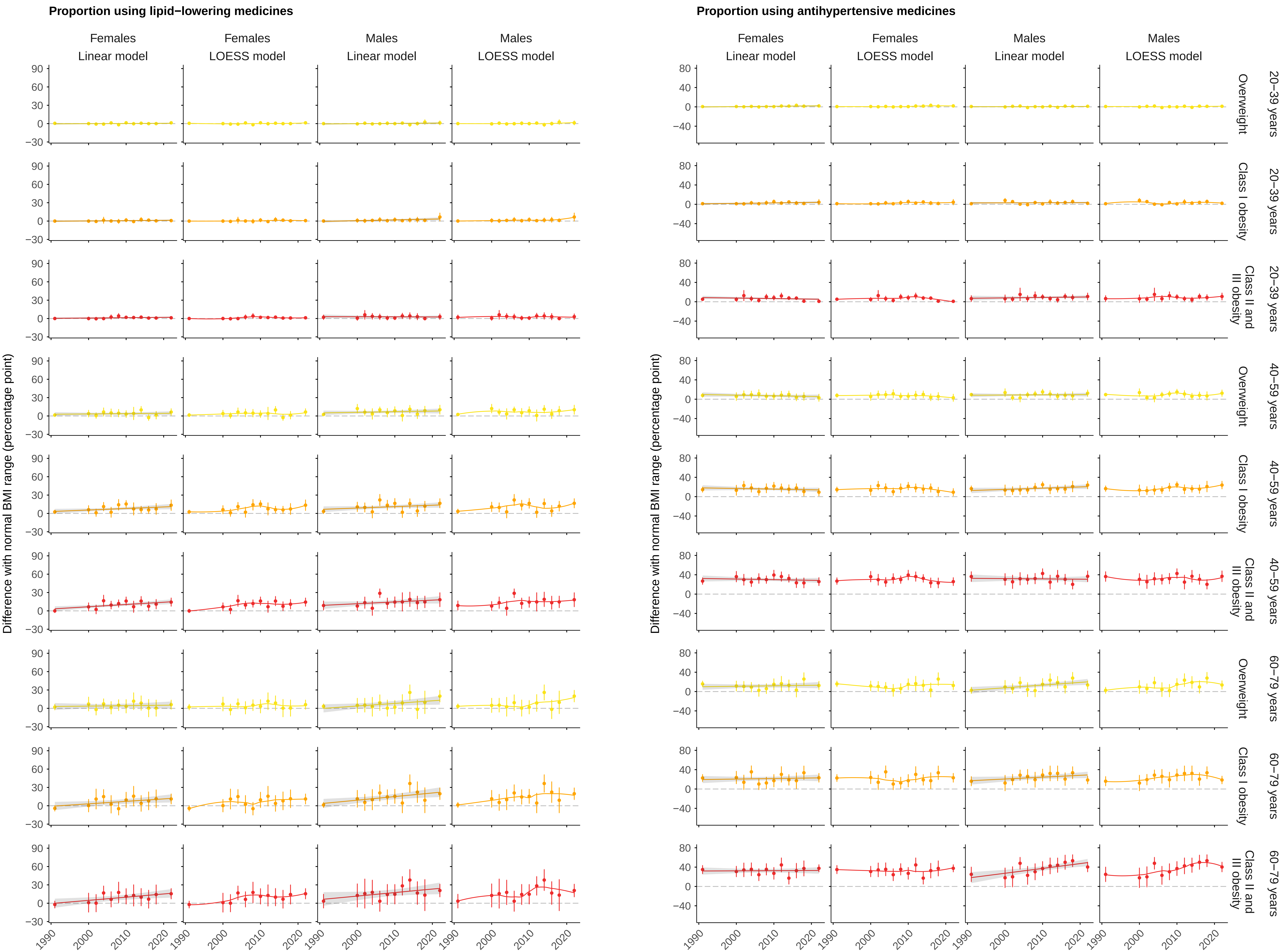

Proportion using lipid−lowering medicines
Proportion using antihypertensive medicines
Females
Linear model
Females
LOESS model
Males
Linear model
Males
LOESS model
Difference with normal BMI range (percentage point)
20−39 years
40−59 years
60−79 years
Overweight
Class I obesity
Class II and III obesity
1990
2000
2010
2020

Appendix Figure 13. Trends in age-standardised mean non-HDL cholesterol, HDL cholesterol and SBP by BMI range without exclusions based on number of participants.

Each point represents one sex-age-BMI group from one study, calculated as stated in Methods. Error bars show 95% confidence intervals, calculated as stated in Methods. See Appendix Figure 14 for difference between obesity or overweight and normal BMI.

Age range for data from Finland is 30-59 years, grouped into 30-39 years and 40-59 years.

Non-HDL cholesterol

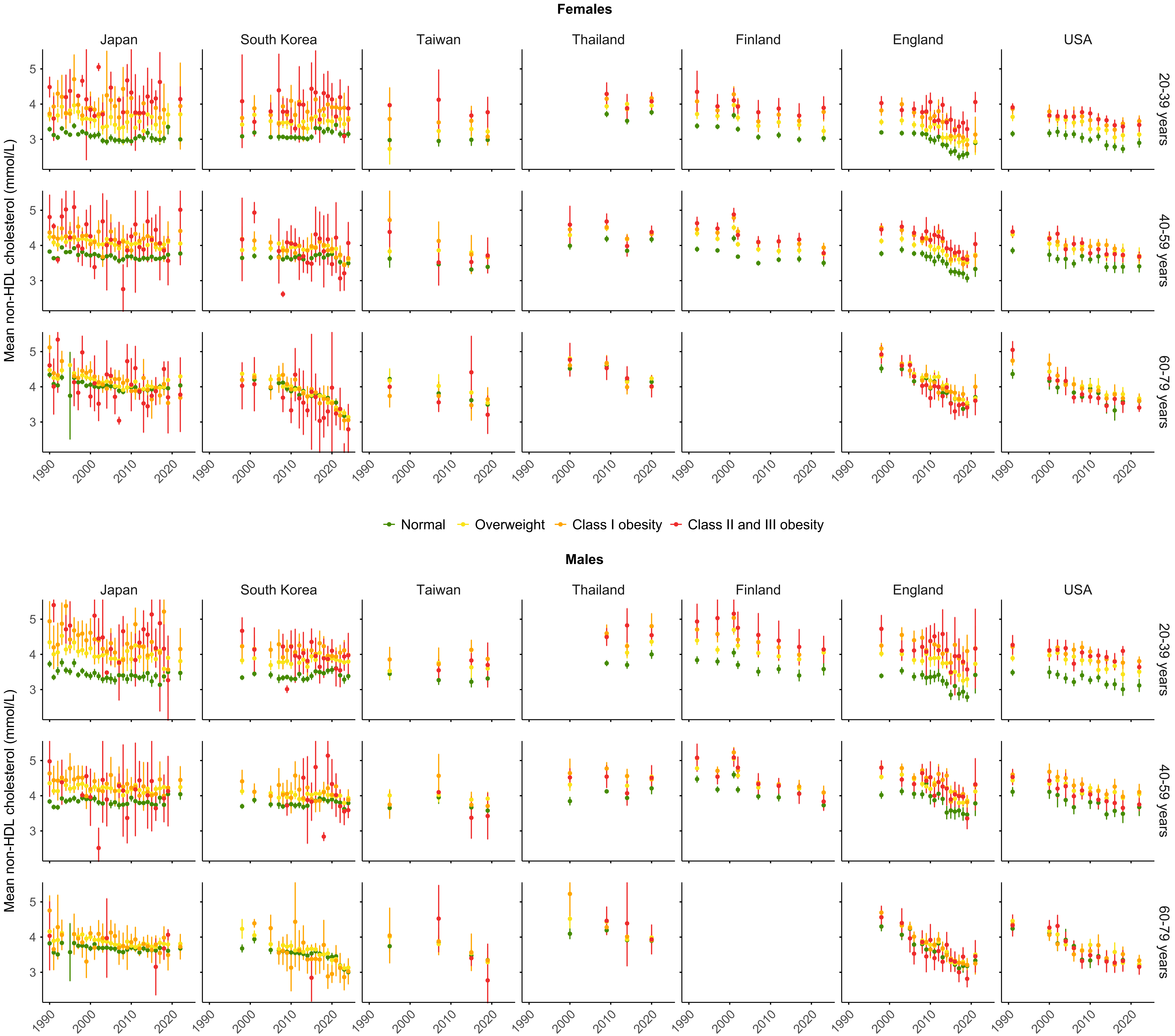

HDL cholesterol

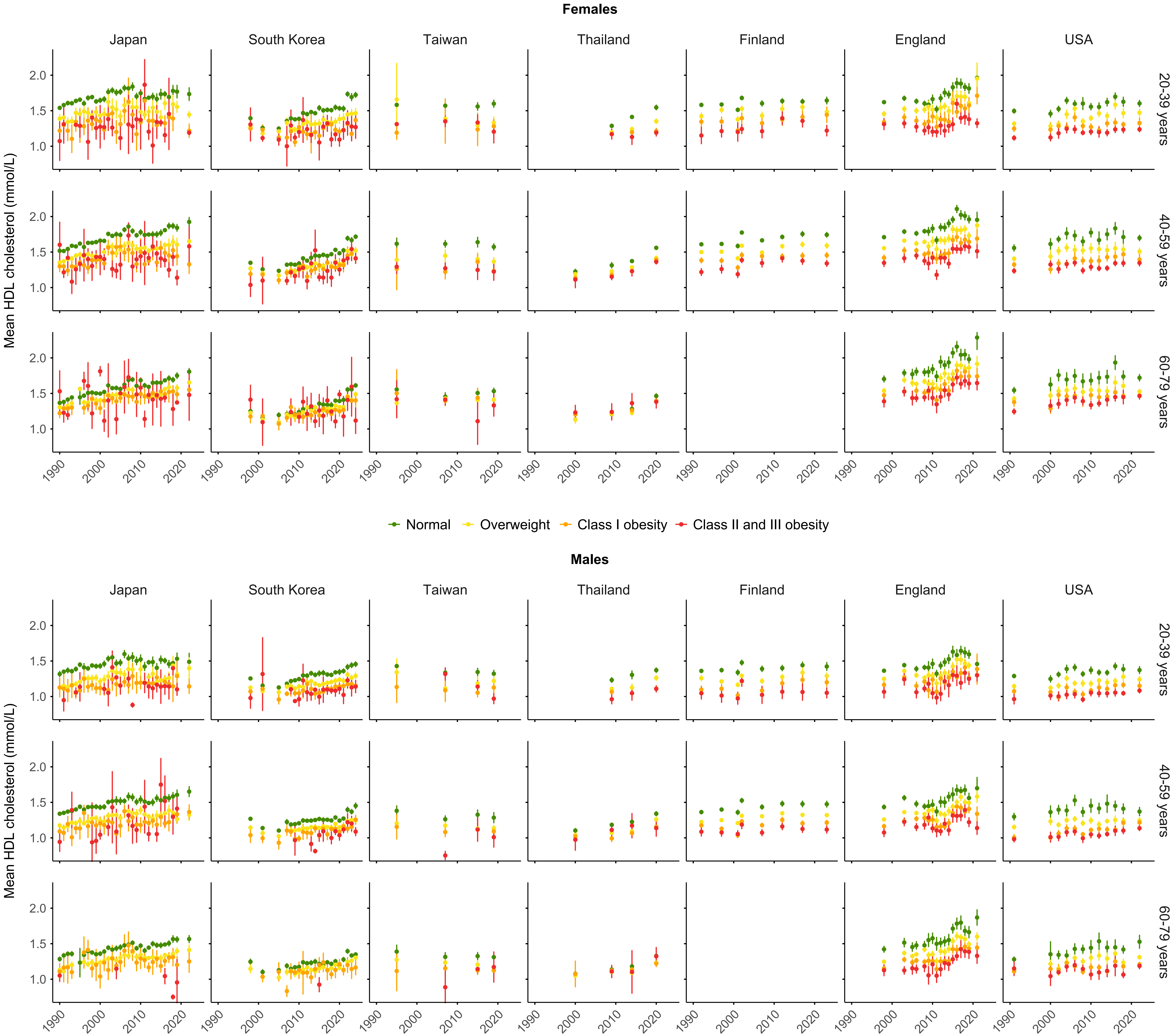

Systolic blood pressure

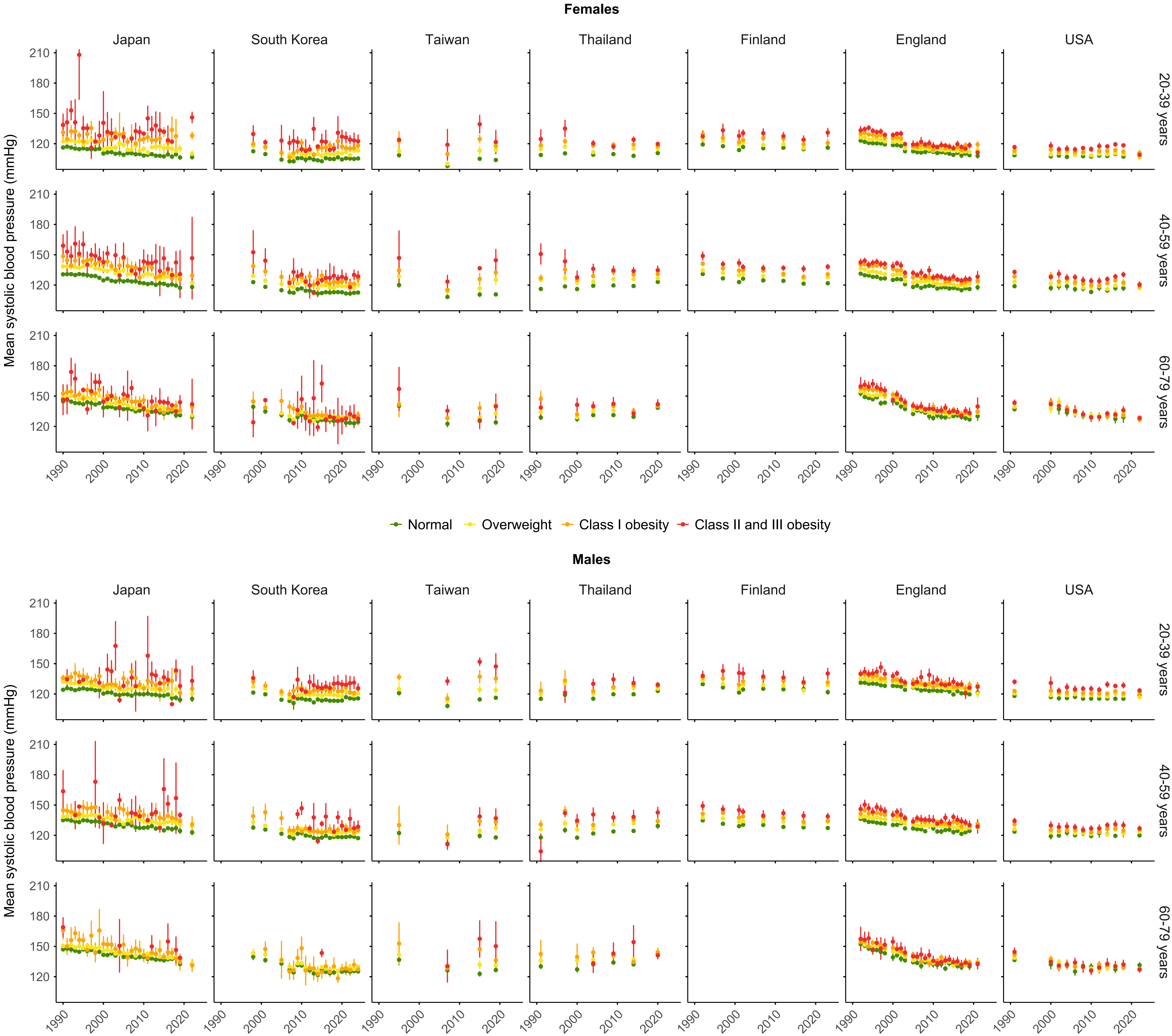

Appendix Figure 14. Trends in difference in age-standardised proportion of participants using lipid-lowering or antihypertensive medicines between obesity and overweight and normal BMI without exclusions based on number of participants.

Each point represents one sex-age-BMI group from one study, calculated as stated in Methods. Error bars show 95% confidence intervals, calculated as stated in Methods.

Age range for data from Finland is 30-59 years, grouped into 30-39 years and 40-59 years.

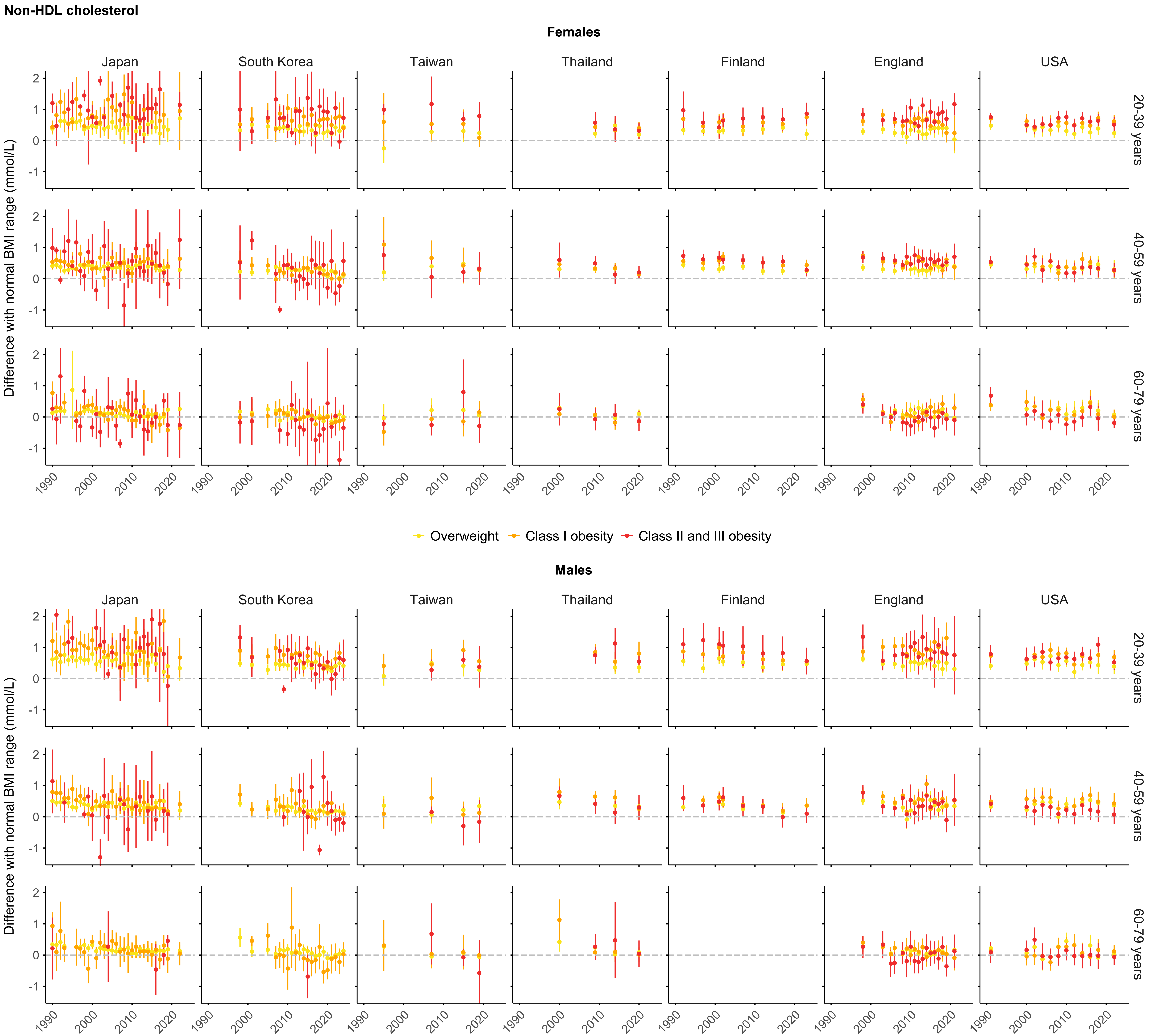

Non-HDL cholesterol
Females
Males
Japan
South Korea
Taiwan
Thailand
Finland
England
USA
20-39 years
40-59 years
60-79 years
Difference with normal BMI range (mmol/L)
Overweight
Class I obesity
Class II and III obesity
1990
2000
2010
2020

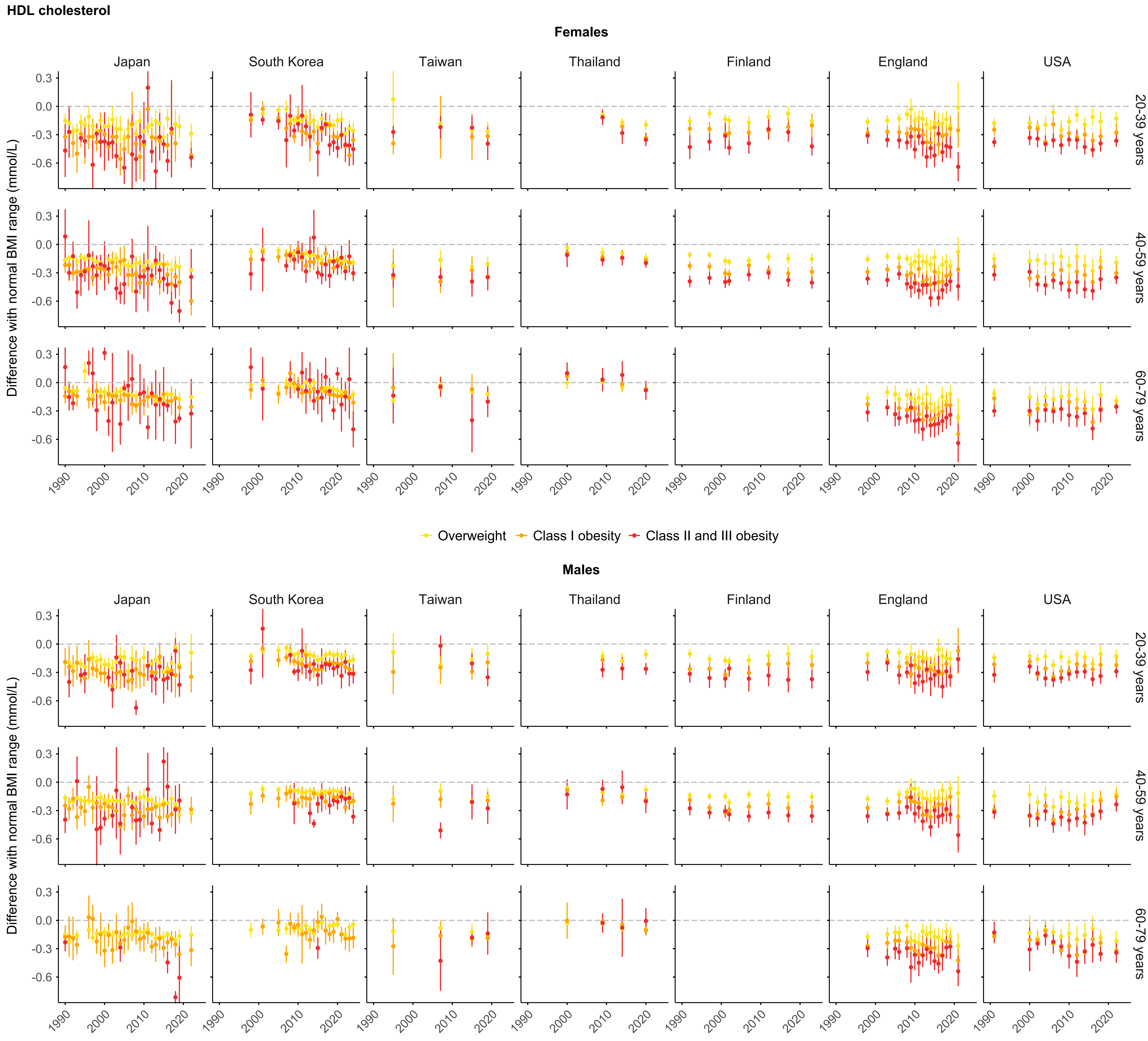

HDL cholesterol
Females
Males
Japan
South Korea
Taiwan
Thailand
Finland
England
USA
20-39 years
40-59 years
60-79 years
Difference with normal BMI range (mmol/L)
Overweight
Class I obesity
Class II and III obesity

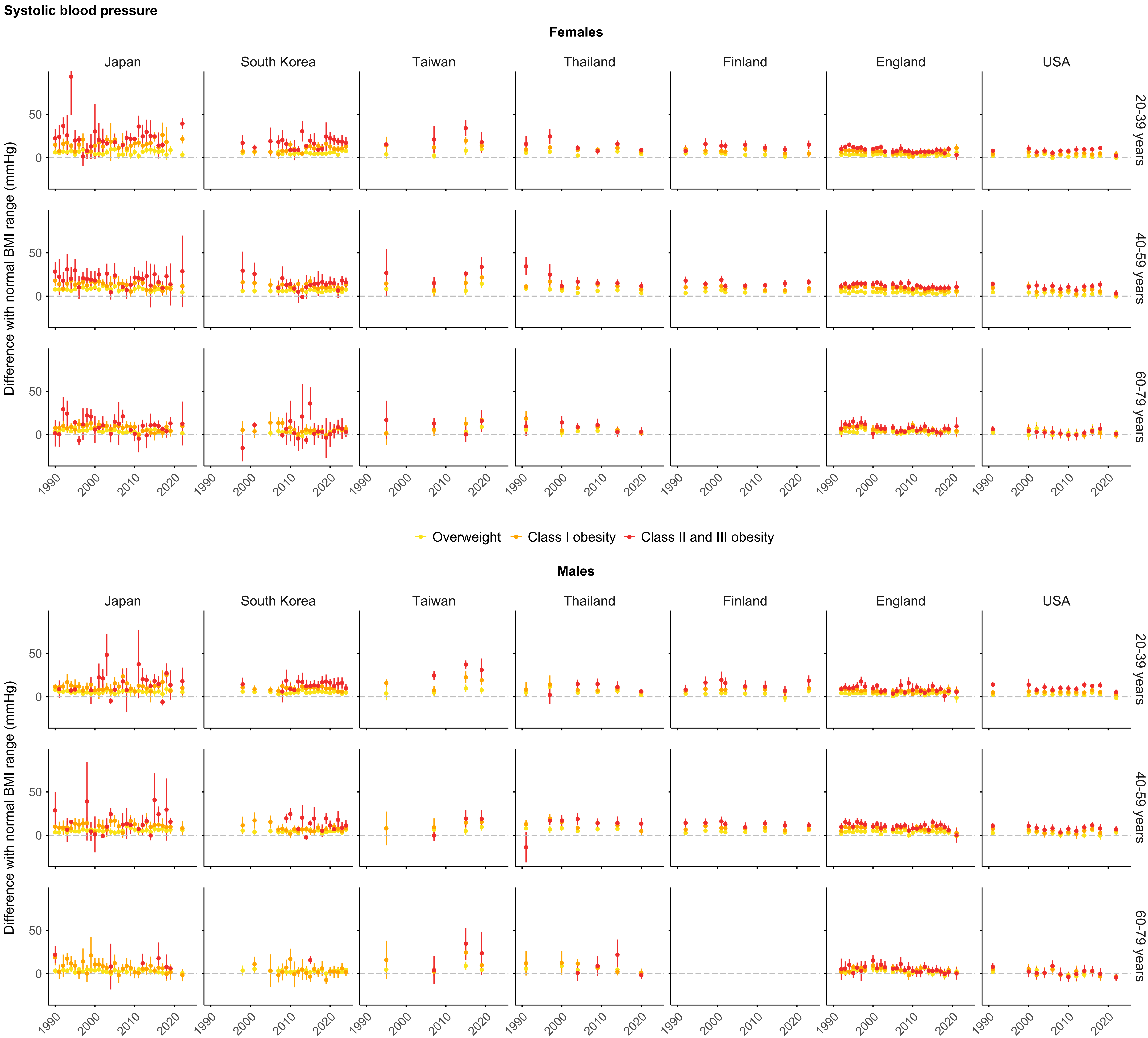
Systolic blood pressure
Females
Males
Japan
South Korea
Taiwan
Thailand
Finland
England
USA
20-39 years
40-59 years
60-79 years
Difference with normal BMI range (mmHg)
Overweight
Class I obesity
Class II and III obesity

Appendix Figure 15. Change in difference in cholesterol and blood pressure between obesity or overweight and normal BMI without exclusions based on number of participants.

The arrows show how the difference in cholesterol and blood pressure between obesity or overweight and normal BMI changed from earliest to latest years of data. An arrow pointing towards the vertical line at 0 indicates convergence, i.e., the difference in outcome between higher BMI and normal BMI shrank. An arrow pointing away from the vertical line indicates divergence, i.e., the difference in outcome between higher BMI and normal BMI expanded. Regardless of the arrow's direction, a positive number means cholesterol or blood pressure is higher in obesity and overweight BMI than in normal BMI, and vice versa. Orange arrows indicate a rightward (positive) change towards or above the level of normal BMI, blue arrows indicate a leftward (negative) change towards or below the level of normal BMI. The arrows are shaded based on the probability that the estimated change is a true decrease or increase. If an increase in difference is indistinguishable from a decrease in difference, the *P* is .5 and is shown in white. Probabilities close to .5 indicate less certainty of an increase or decrease in difference. Probabilities closer to 1 indicate more certainty that the estimated decrease or increase is a true change in that direction. For an estimated decrease, these high probabilities are shown in darker blue; for an estimated increase, they are shown in darker orange. See Appendix Figure 16 for change per decade, which is not affected by the differences in the earliest and latest years of data across countries.

Age range for data from Finland is 30-59 years, grouped into 30-39 years and 40-59 years.

Non-HDL cholesterol

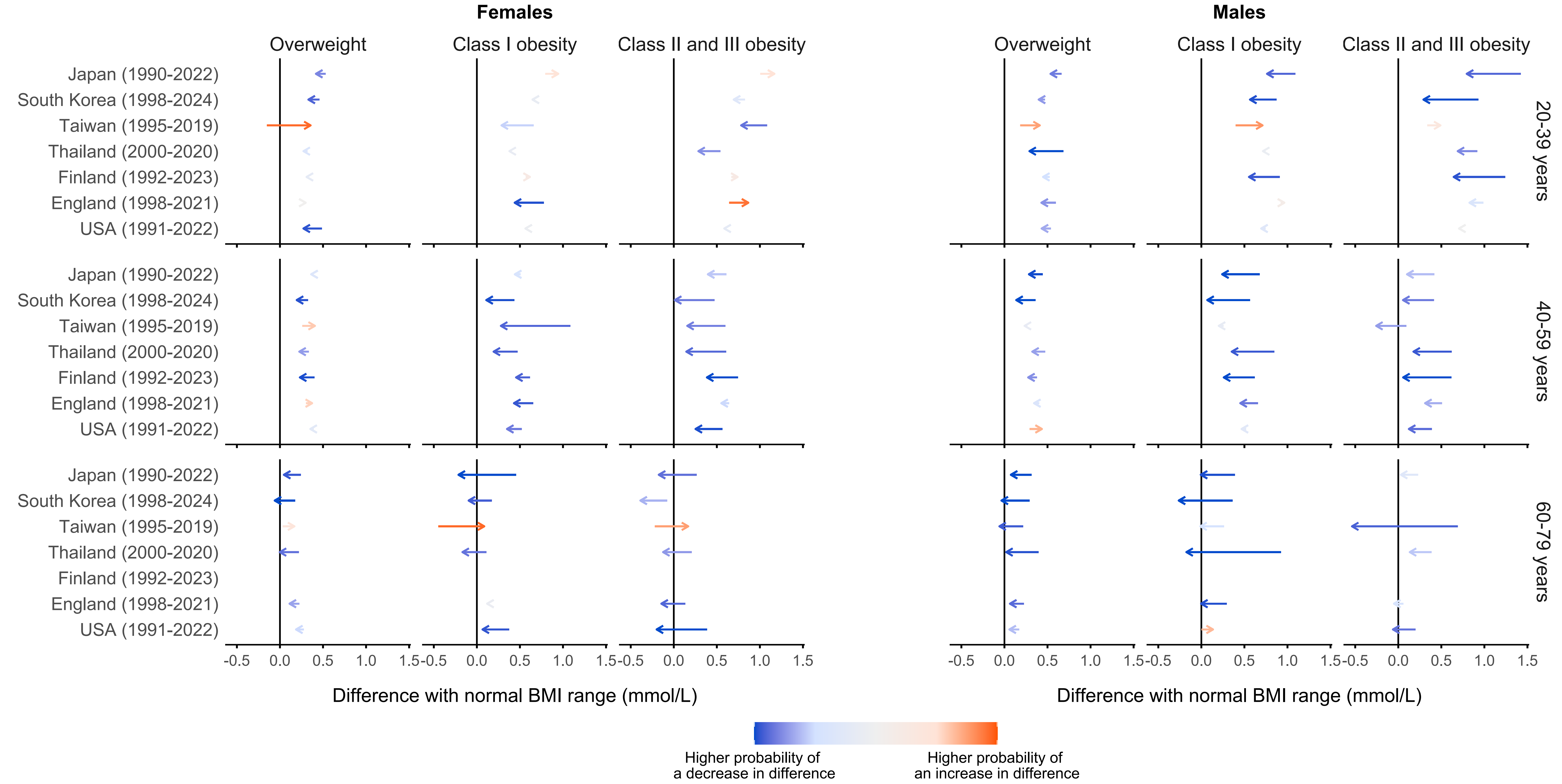

HDL cholesterol

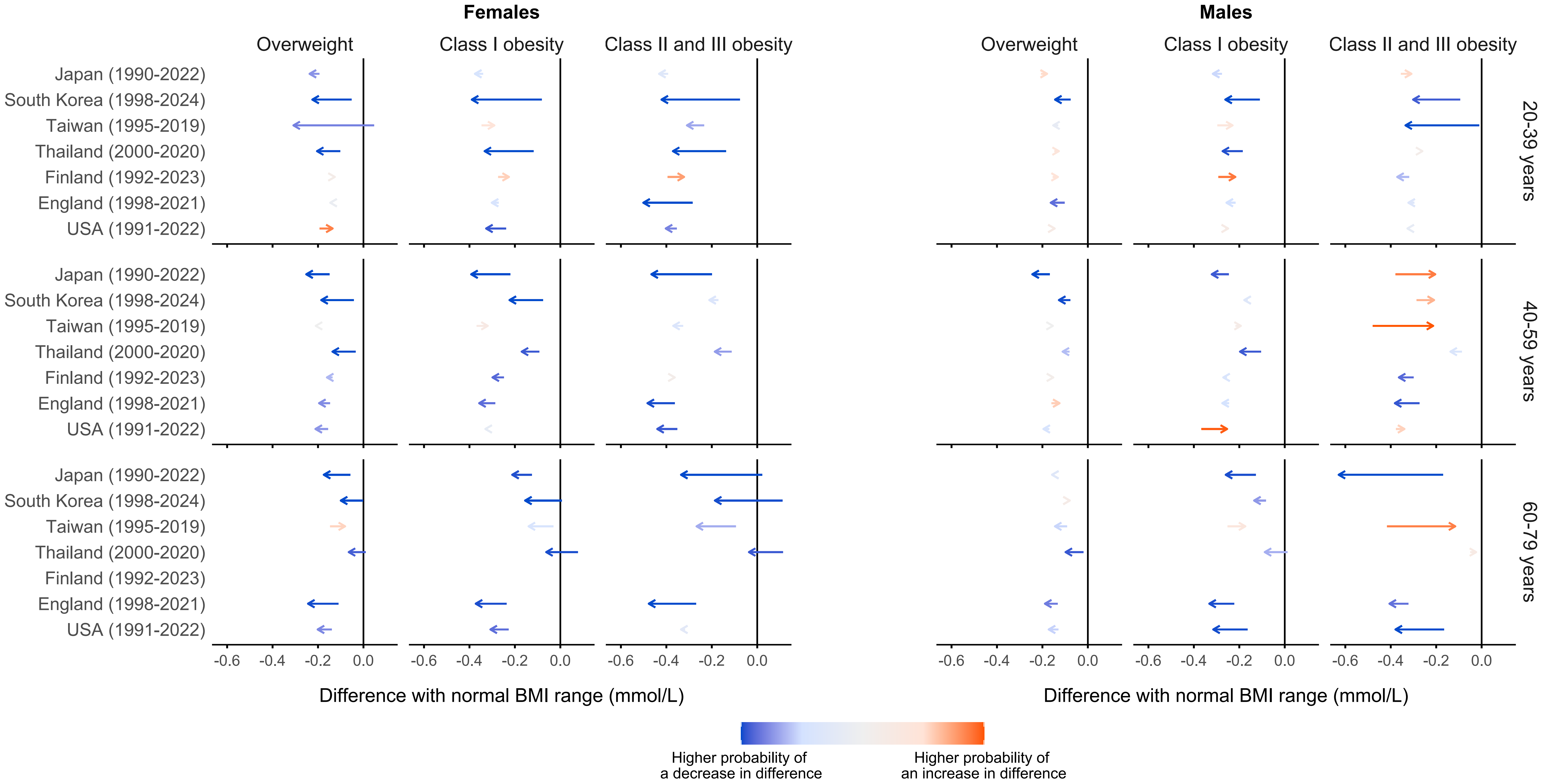

Systolic blood pressure

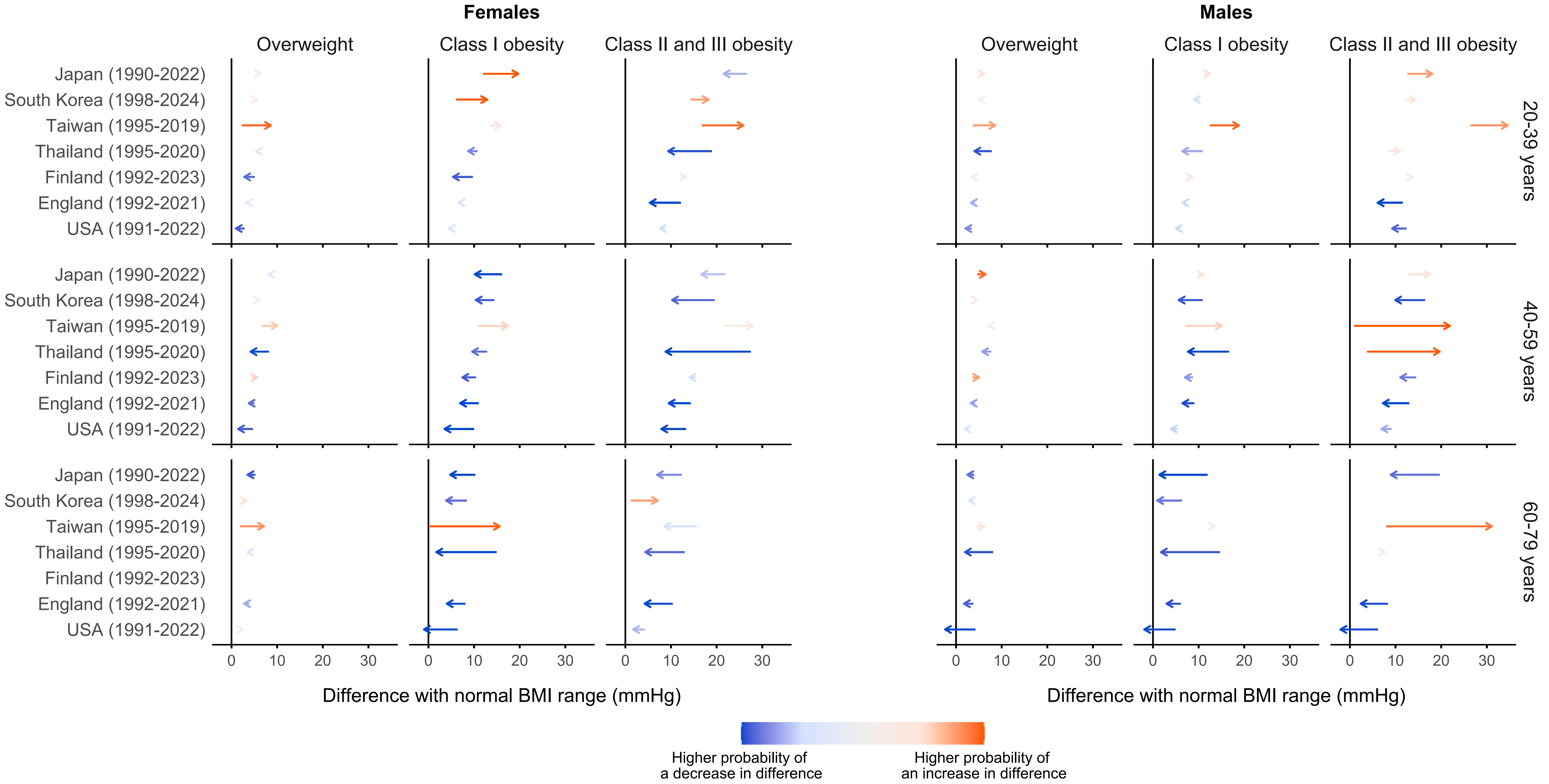

Appendix Figure 16. Pooled analysis of change in difference in cholesterol and blood pressure between obesity or overweight and normal BMI without exclusions based on number of participants.

For each sex and age group, the forest plot shows change per decade in the difference in cholesterol and blood pressure between obesity or overweight and normal BMI for each country as well as the pooled results across countries.

Non−HDL Cholesterol

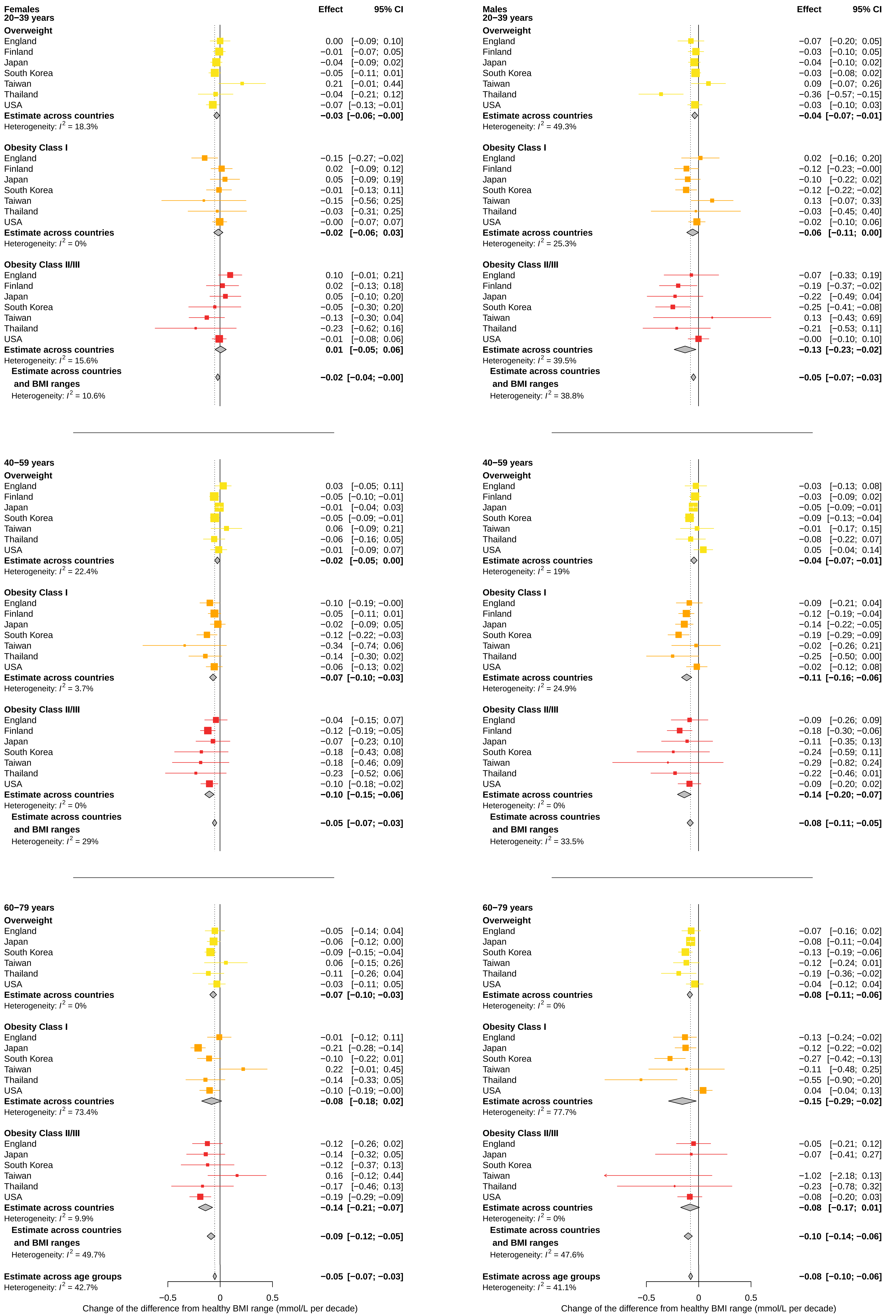

HDL Cholesterol

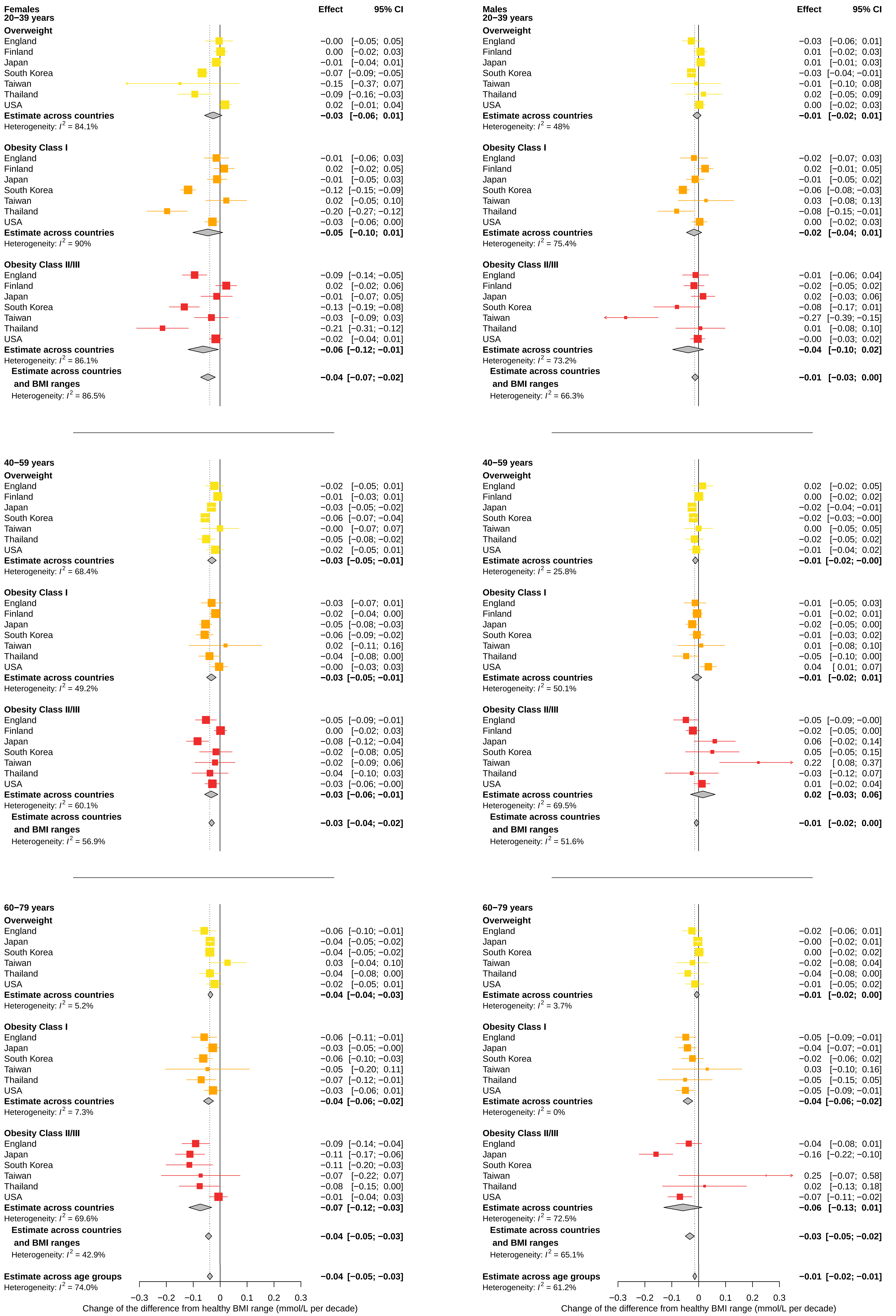

Systolic blood pressure

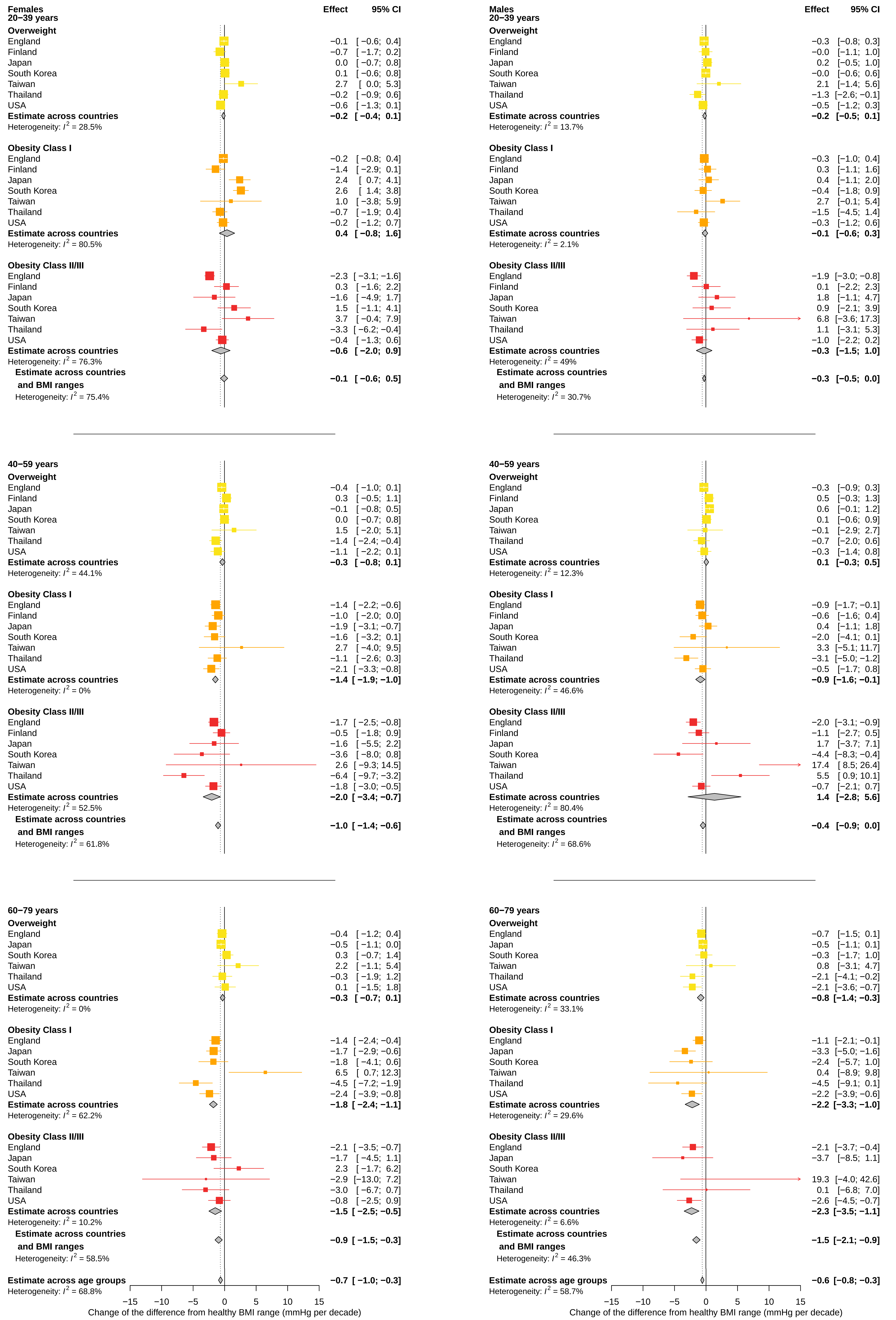

Appendix Figure 17. Trends in age-standardised proportion of participants using lipid-lowering or antihypertensive medicines, by BMI range without exclusions based on number of participants.

Each point represents one sex-age-BMI range group from one study, calculated as stated in Methods. Error bars show 95% confidence intervals, calculated as stated in Methods. See Appendix Figure 18 for difference between obesity or overweight and normal BMI.

Age range for data from Finland is 30-59 years, grouped into 30-39 years and 40-59 years.

Proportion using lipid-lowering medicines

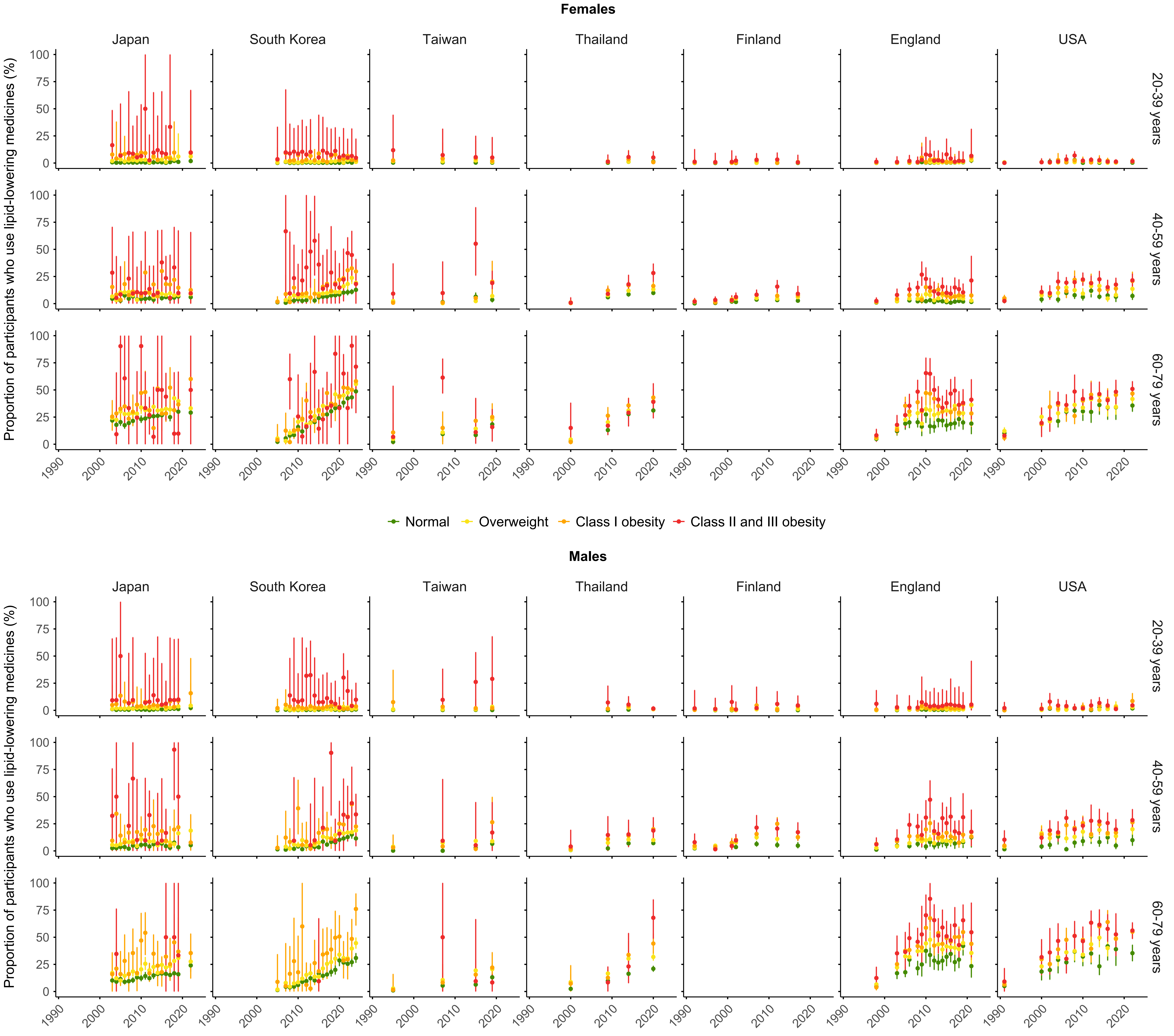

Proportion using antihypertensive medicines

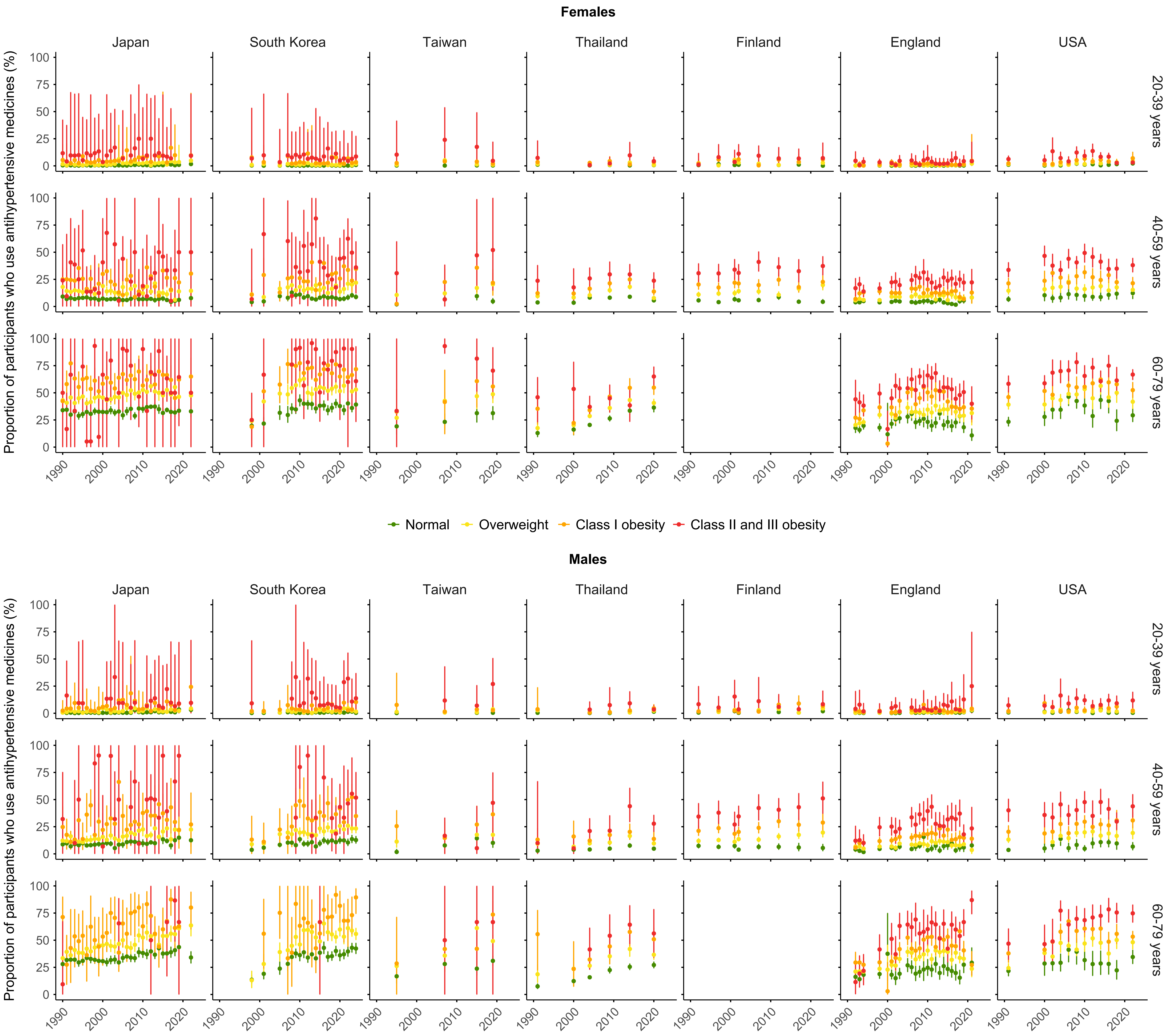

Appendix Figure 18. Trends in difference in age-standardised proportion of participants using lipid-lowering or antihypertensive medicines between obesity and overweight and normal BMI without exclusions based on number of participants.

Each point represents one sex-age-BMI group from one study, calculated as stated in Methods. Error bars show 95% confidence intervals, calculated as stated in Methods.

Age range for data from Finland is 30-59 years, grouped into 30-39 years and 40-59 years.

Proportion using lipid-lowering medicines

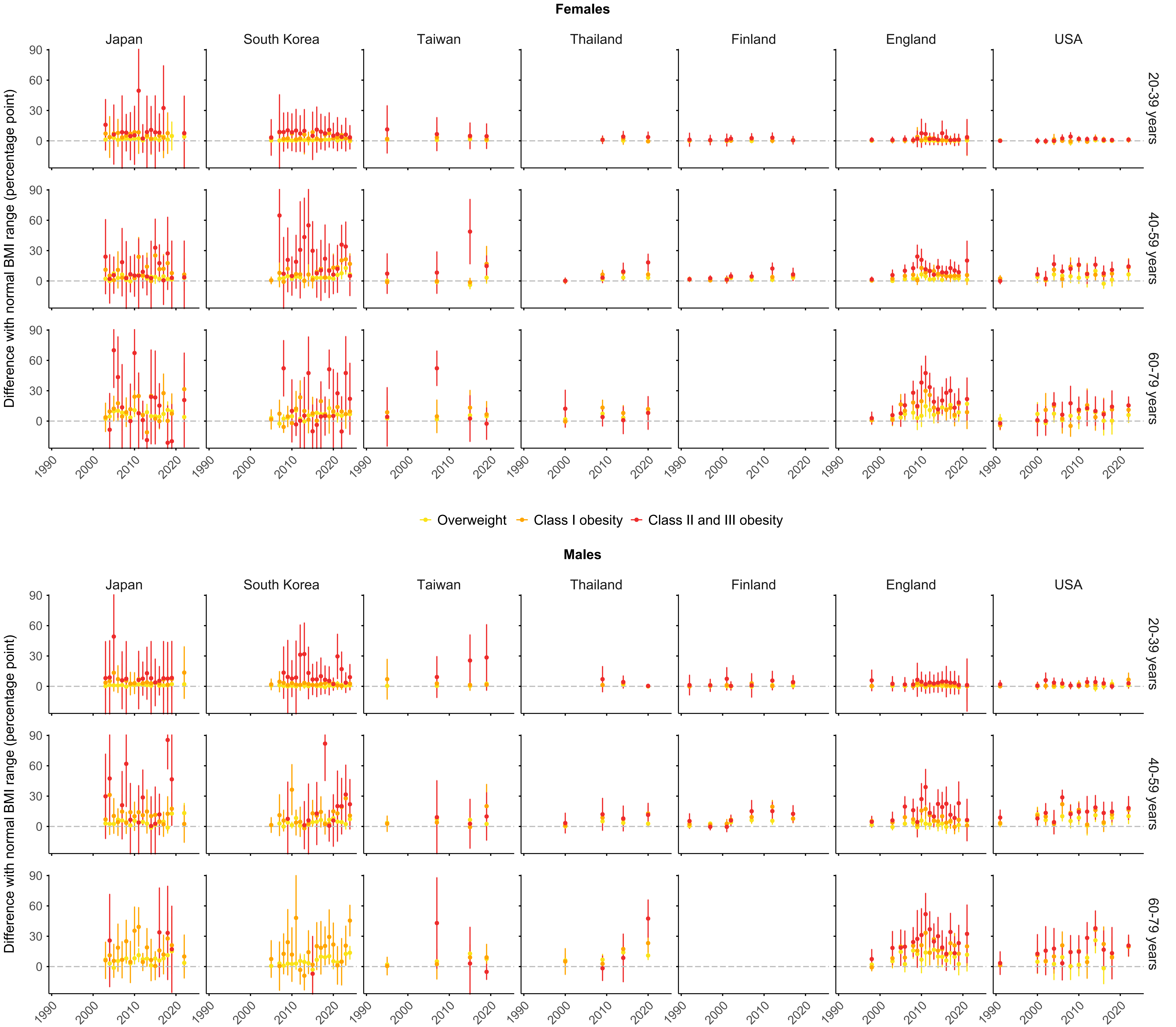

Proportion using antihypertensive medicines

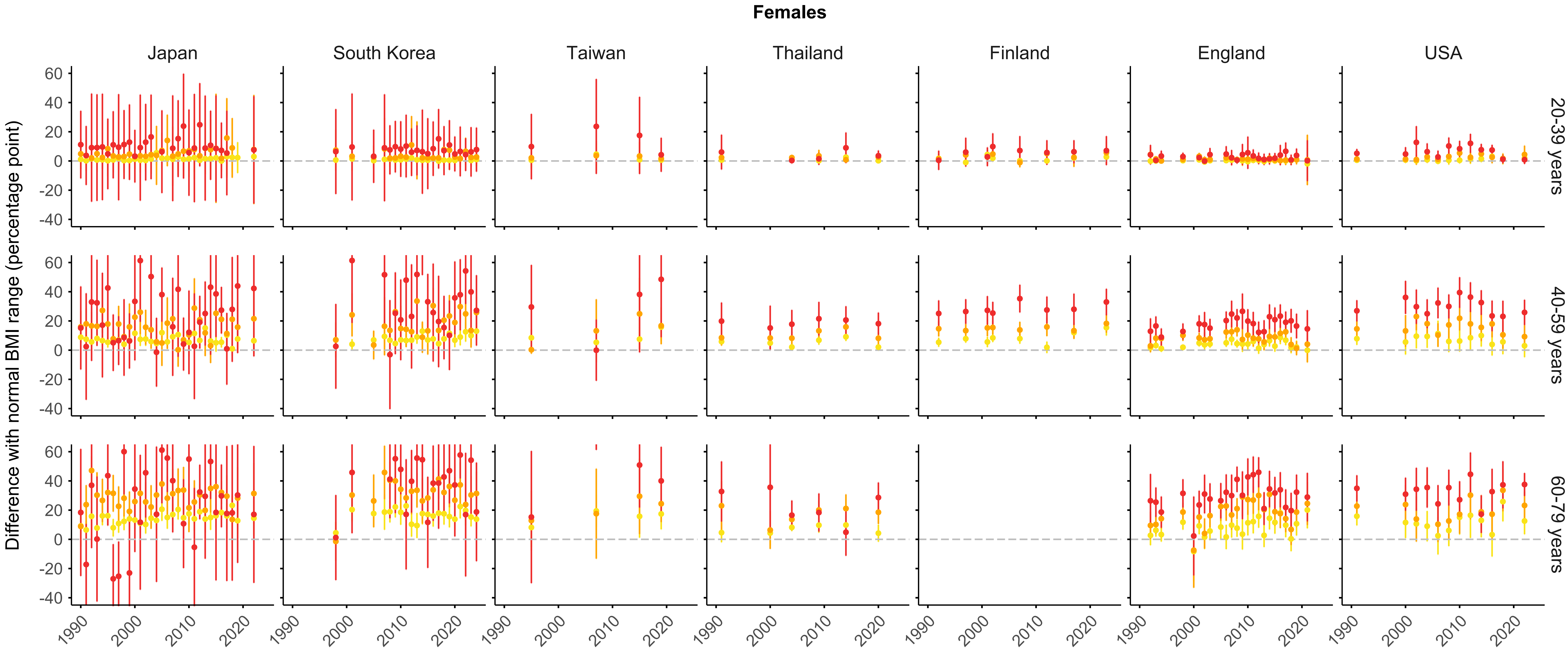

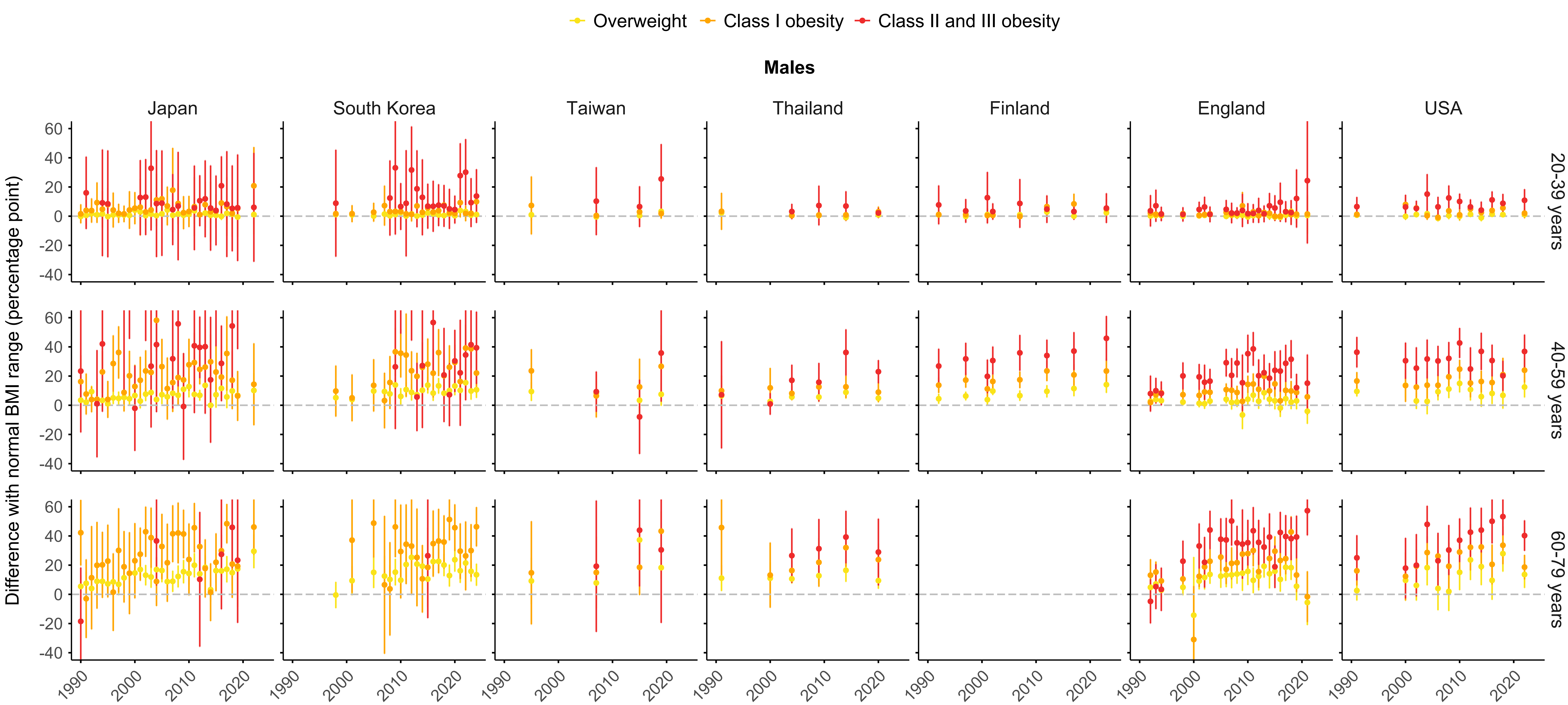

Appendix Figure 19. Change in difference in use of lipid-lowering and antihypertensive medicines between obesity and overweight and normal BMI without exclusions based on number of participants.

The arrows show how the difference in treatment between obesity or overweight and normal BMI changed from earliest to latest years of data. An arrow pointing towards the vertical line at 0 indicates convergence, i.e., the difference in outcome between higher BMI and normal BMI shrank. An arrow pointing away from the vertical line indicates divergence, i.e., the difference in outcome between higher BMI and normal BMI expanded. Regardless of the arrow's direction, a positive number means treatment is higher in obesity and overweight BMI than in normal BMI, and vice versa. Orange arrows indicate a rightward (positive) change towards or above the level of normal BMI, blue arrows indicate a leftward (negative) change towards or below the level of normal BMI. The arrows are shaded based on the probability that the estimated change is a true decrease or increase. If an increase in difference is indistinguishable from a decrease in difference, the *P* is .5 and is shown in white. Probabilities close to .5 indicate less certainty of an increase or decrease in difference. Probabilities closer to 1 indicate more certainty that the estimated decrease or increase is a true change in that direction. For an estimated decrease, these high probabilities are shown in darker blue; for an estimated increase, they are shown in darker orange. See Appendix Figure 20 for change per decade, which is not affected by the differences in the earliest and latest years of data across countries.

Age range for data from Finland is 30-59 years, grouped into 30-39 years and 40-59 years.

Proportion using lipid-lowering medicines

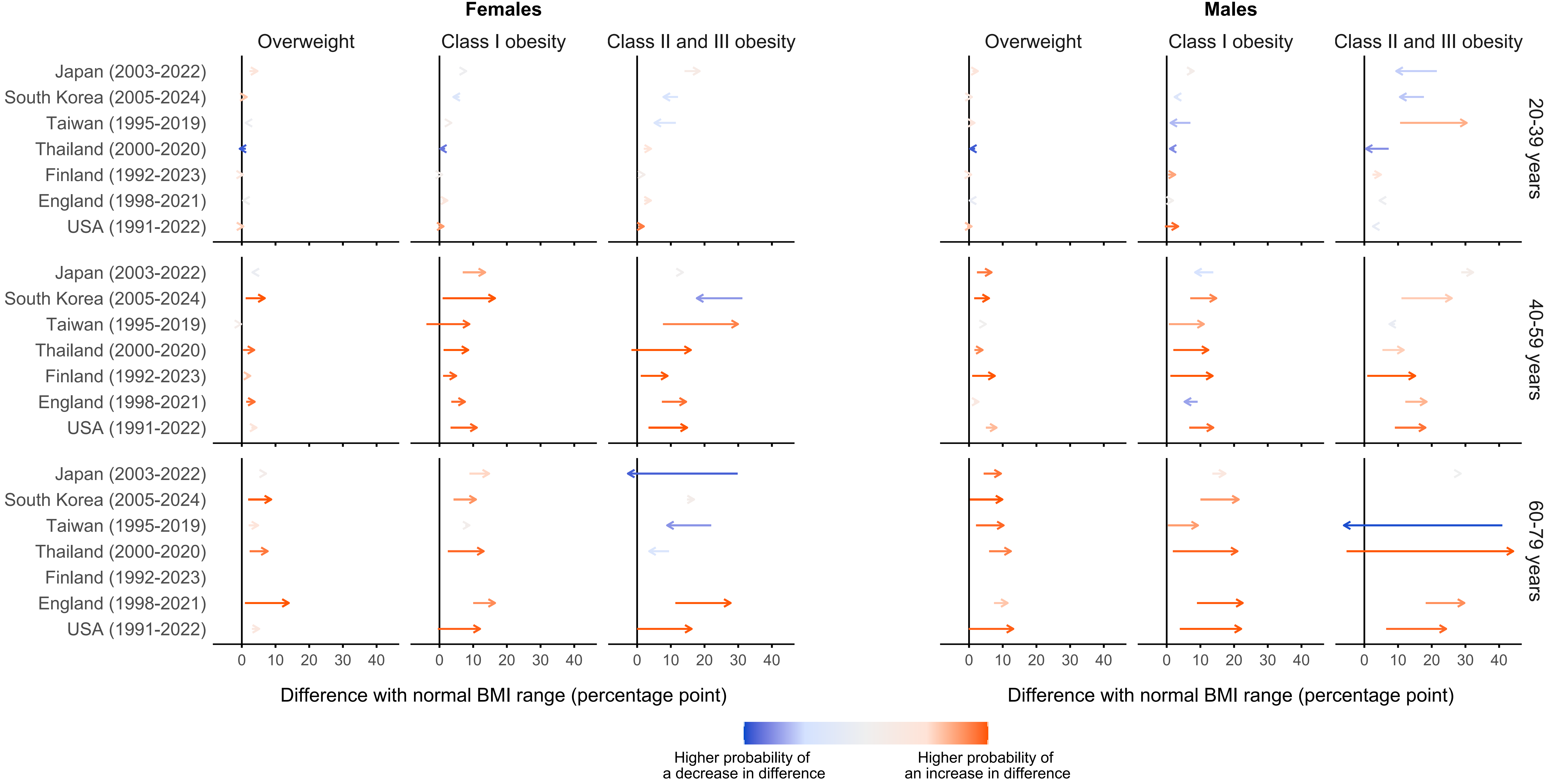

Proportion using antihypertensive medicines

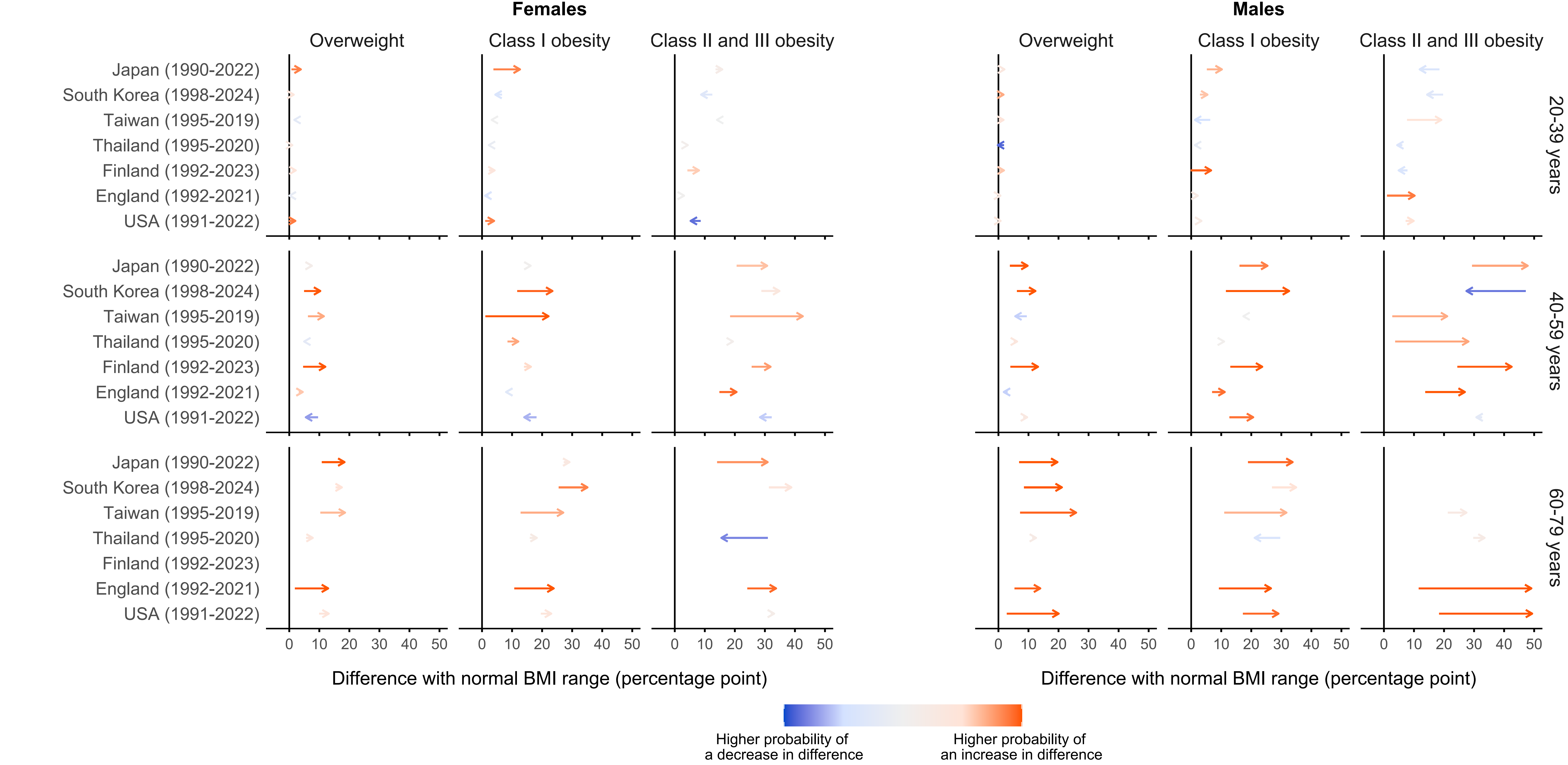

Appendix Figure 20. Pooled analysis of change in difference in antihypertensive and lipid-lowering medicines between obesity or overweight and normal BMI without exclusions based on number of participants.

For each sex and age group, the forest plot shows change per decade in the difference in in treatment between obesity or overweight and normal BMI for each country as well as the pooled results across countries.

Proportion using lipid−lowering medicines

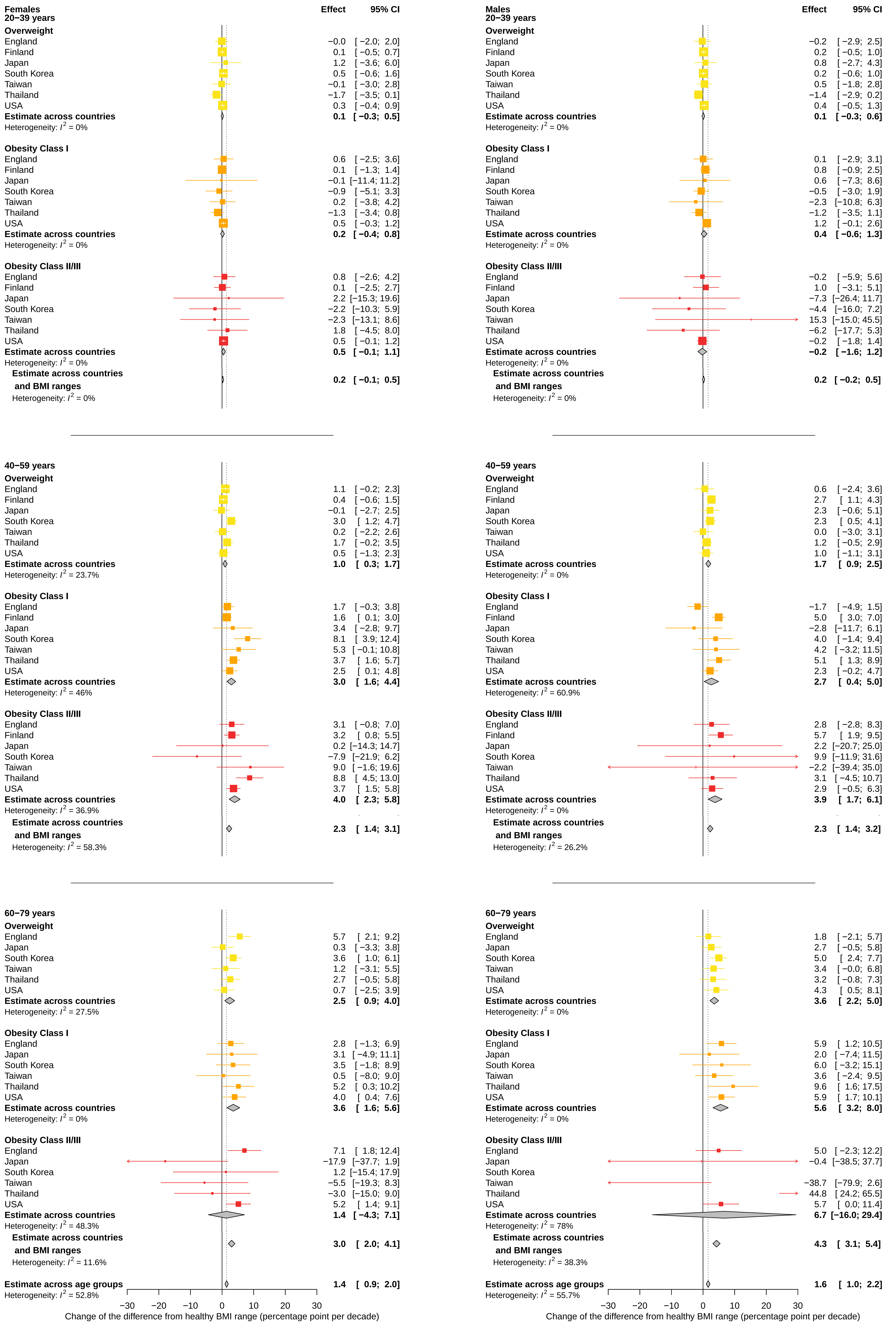

Proportion using antihypertensive medicines

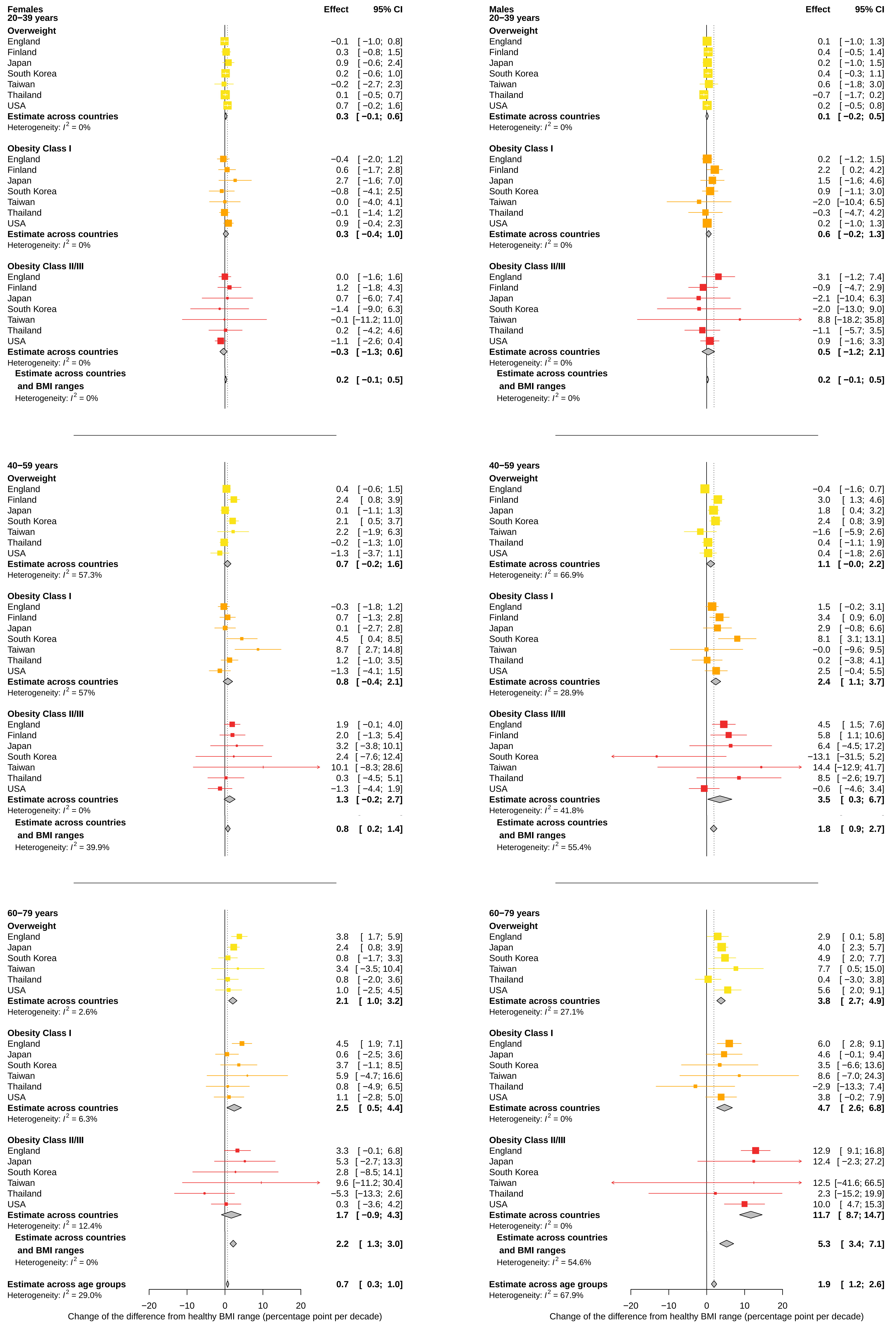

Appendix Figure 21. Relationship between BMI and non-HDL cholesterol per decade.

Each line shows the linear relationship between BMI and non-HDL cholesterol for one sex, age group, country and decade, obtained by fitting a linear regression between the two variables.

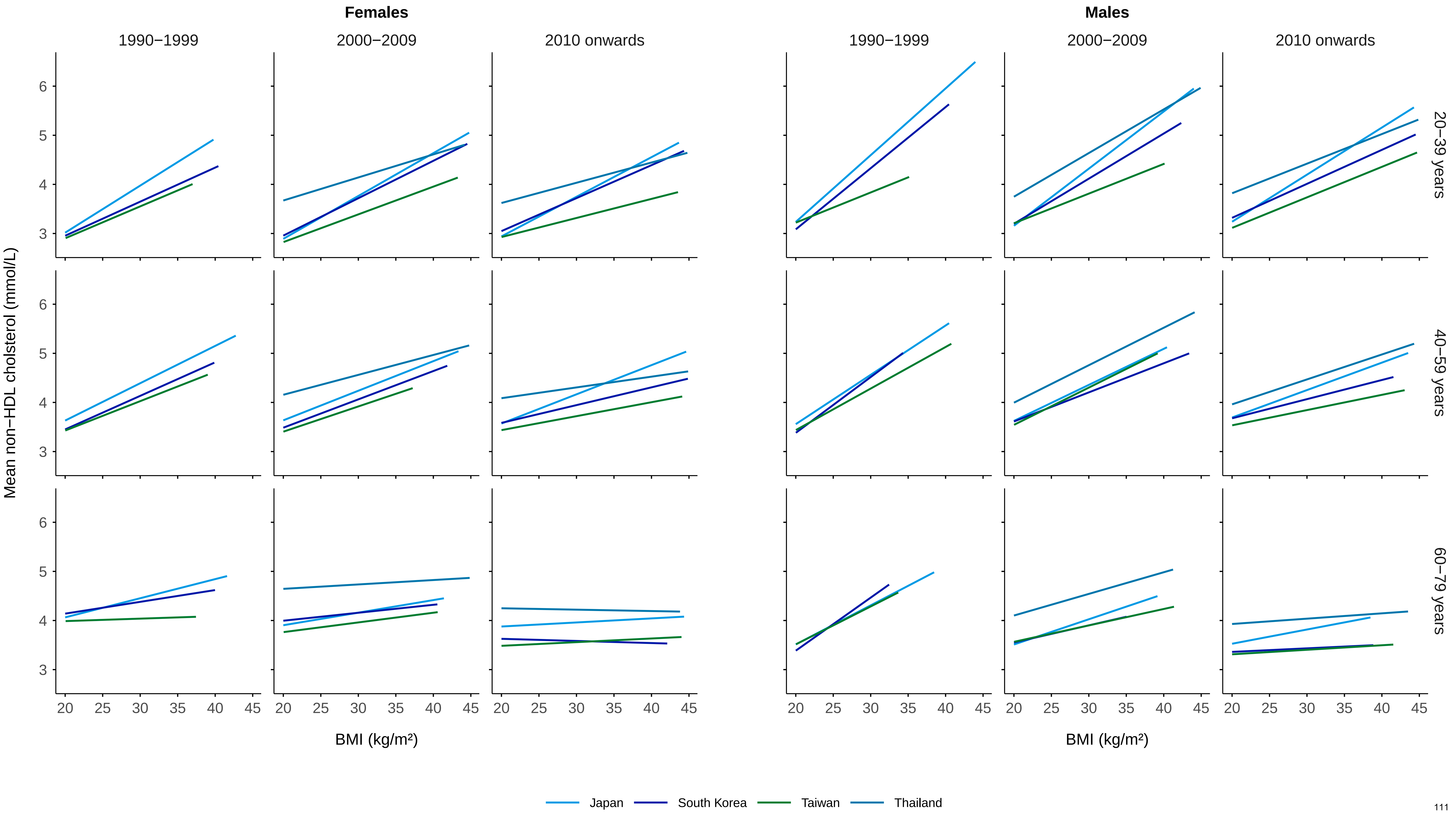
Females
Males
1990−1999
2000−2009
2010 onwards
20−39 years
40−59 years
60−79 years
Mean non−HDL cholsterol (mmol/L)
BMI (kg/m²)
3
4
5
6
20
25
30
35
40
45
Japan
South Korea
Taiwan
Thailand

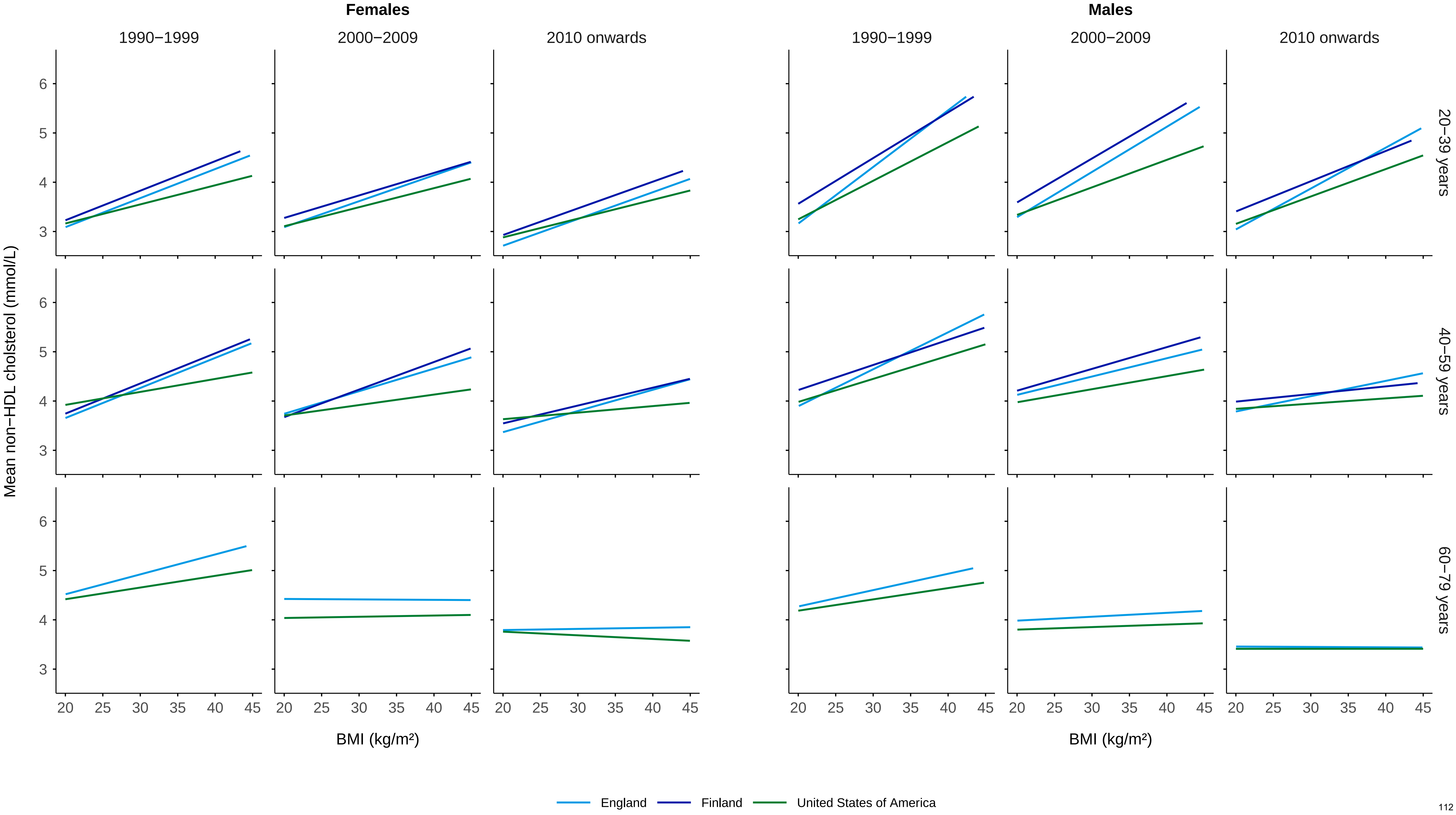
Females
Males
1990−1999
2000−2009
2010 onwards
20−39 years
40−59 years
60−79 years
Mean non−HDL cholsterol (mmol/L)
BMI (kg/m²)
England
Finland
United States of America

Appendix Figure 22. Relationship between BMI and systolic blood pressure per decade.

Each line shows the linear relationship between BMI and systolic blood pressure for one sex, age group, country and decade, obtained by fitting a linear regression between the two variables.

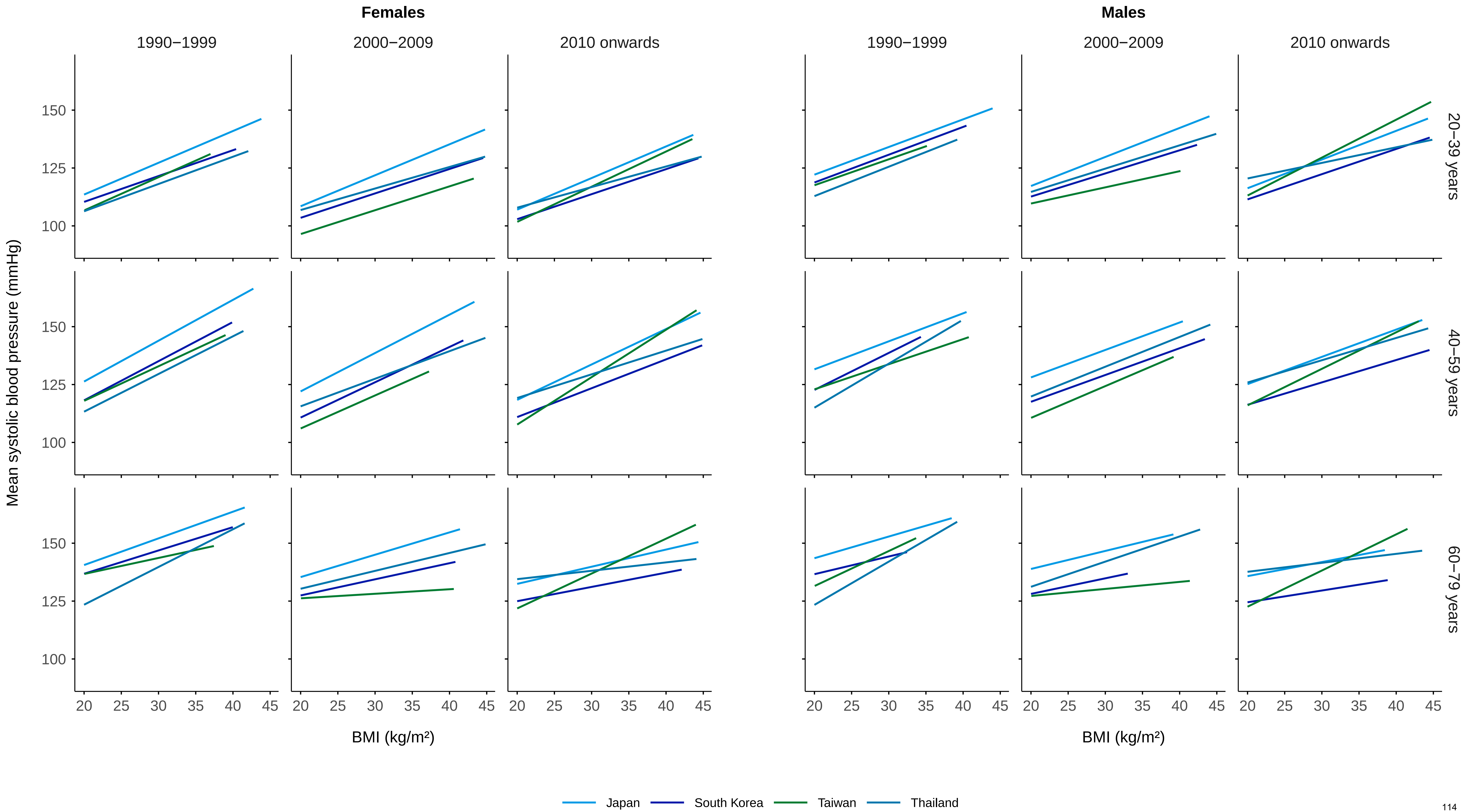
Females
Males
1990−1999
2000−2009
2010 onwards
20−39 years
40−59 years
60−79 years
Mean systolic blood pressure (mmHg)
BMI (kg/m²)
Japan
South Korea
Taiwan
Thailand

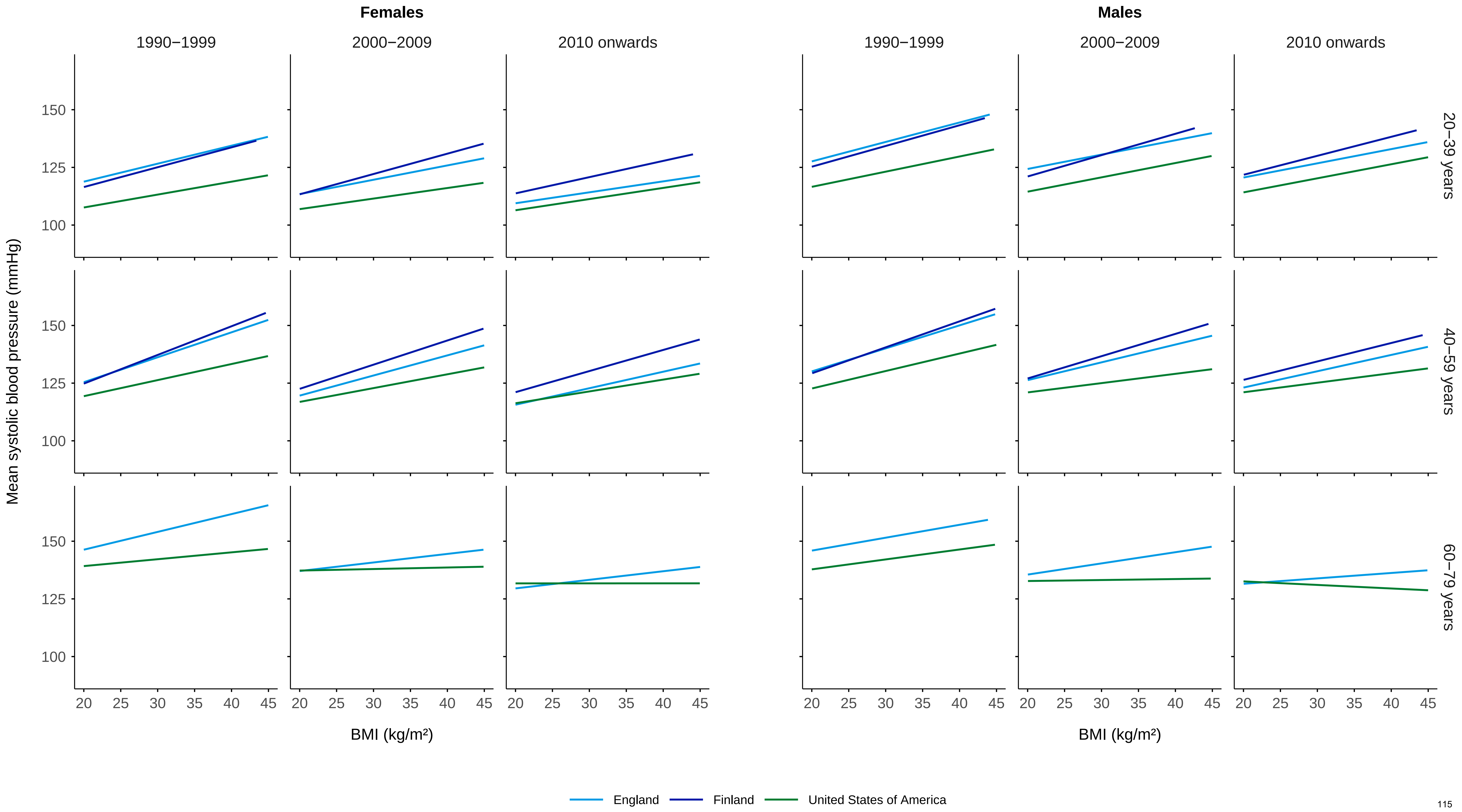
Females
Males
1990−1999
2000−2009
2010 onwards
20−39 years
40−59 years
60−79 years
Mean systolic blood pressure (mmHg)
100
125
150
20
25
30
35
40
45
BMI (kg/m²)
England
Finland
United States of America

References

1. NCD Risk Factor Collaboration (NCD-RisC). National trends in total cholesterol obscure heterogeneous changes in HDL and non-HDL cholesterol and total-to-HDL cholesterol ratio: a pooled analysis of 458 population-based studies in Asian and Western countries. *Int J Epidemiol* 2020; **49**(1): 173-92.

2. Arsenault BJ, Rana JS, Stroes ES, et al. Beyond low-density lipoprotein cholesterol: respective contributions of non-high-density lipoprotein cholesterol levels, triglycerides, and the total cholesterol/high-density lipoprotein cholesterol ratio to coronary heart disease risk in apparently healthy men and women. *J Am Coll Cardiol* 2009; **55**(1): 35-41.

3. Liu J, Sempos CT, Donahue RP, Dorn J, Trevisan M, Grundy SM. Non–high-density lipoprotein and very-low-density lipoprotein cholesterol and their risk predictive values in coronary heart disease. *The American Journal of Cardiology* 2006; **98**(10): 1363-8.

4. WHO expert consultation. Appropriate body-mass index for Asian populations and its implications for policy and intervention strategies. *The Lancet* 2004.

5. NCD Risk Factor Collaboration (NCD-RisC). General and abdominal adiposity and hypertension in eight world regions: a pooled analysis of 837 population-based studies with 7·5 million participants. *Lancet* 2024; **404**(10455): 851-63.

6. de Onis M, Onyango AW, Borghi E, Siyam A, Nishida C, Siekmann J. Development of a WHO growth reference for school-aged children and adolescents. *Bull World Health Organ* 2007; **85**(9): 660-7.

7. NCD Risk Factor Collaboration (NCD-RisC). Obesity rise plateaus in developed nations and accelerates in developing nations. *Nature* 2026; **653**(8114): 510-8.

8. NCD Risk Factor Collaboration (NCD-RisC). Worldwide trends in underweight and obesity from 1990 to 2022: a pooled analysis of 3663 population-representative studies with 222 million children, adolescents, and adults. *Lancet* 2024.

9. NCD Risk Factor Collaboration (NCD-RisC). Repositioning of the global epicentre of non-optimal cholesterol. *Nature* 2020; **582**(7810): 73-7.

10. NCD Risk Factor Collaboration (NCD-RisC). Worldwide trends in hypertension prevalence and progress in treatment and control from 1990 to 2019: a pooled analysis of 1201 population-representative studies with 104 million participants. *Lancet* 2021; **398**: 957-80.

11. Loerup L, Pullon RM, Birks J, et al. Trends of blood pressure and heart rate in normal pregnancies: a systematic review and meta-analysis. *BMC Med* 2019; **17**(1): 167.

12. Sun L, Gao B, Wang M, et al. The establishment of lipid profiles reference ranges during pregnancy: a systematic review and meta-analysis. *Reprod Biol Endocrinol* 2025; **23**(1): 110.

13. Cholesterol Reference Method Laboratory Network. Total Cholesterol Certification Protocol for Manufacturers.

14. Cholesterol Reference Method Laboratory Network. LDL Cholesterol Certification Protocol for Manufacturers.

15. Cholesterol Reference Method Laboratory Network. HDL Cholesterol Certification Protocol for Manufacturers.

16. Saydah S, Bullard KM, Cheng Y, et al. Trends in cardiovascular disease risk factors by obesity level in adults in the United States, NHANES 1999-2010. *Obesity* 2014; **22**(8): 1888-95.

17. Gregg EW, Cheng YJ, Cadwell BL, et al. Secular trends in cardiovascular disease risk factors according to body-mass index in US adults. *JAMA* 2005; **293**: 1868.

18. Amuda O, Okosun BO, Abdi H, Okosun IS. Prevalence and secular trends in premetabolic syndrome in the United States: Findings from 1999-2020 nationally representative data of adults. *Ann Epidemiol* 2024; **93**: 10-8.

19. Scholes S, Fat LN, Mindell JS. Trends in cardiovascular disease risk factors by BMI category among adults in England, 2003-2018. *Obesity* 2021; **29**(8): 1347-62.

20. Park K, Lim S, Park Y, Ju W, Shin Y, Yeom H. Cardiovascular disease risk factors and obesity levels in Korean adults: results from the Korea National Health and Nutrition Examination Survey, 2007–2015. *Osong Public Health and Research Perspectives* 2018; **9**(4): 150.

21. Danon-Hersch N, Chiolero A, Shamlaye C, Paccaud F, Bovet P. Decreasing association between body mass index and blood pressure over time. *Epidemiology* 2007; **18**: 493-500.

22. Ikeda N, Gakidou E, Hasegawa T, Murray CJ. Understanding the decline of mean systolic blood pressure in Japan: an analysis of pooled data from the National Nutrition Survey, 1986-2002. *Bull World Health Organ* 2008; **86**(12): 978-88.

23. Kim HJ, Kim Y, Cho Y, Jun B, Oh KW. Trends in the prevalence of major cardiovascular disease risk factors among Korean adults: results from the Korea National Health and Nutrition Examination Survey, 1998-2012. *Int J Cardiol* 2014; **174**(1): 64-72.

24. Borodulin K, Vartiainen E, Peltonen M, et al. Forty-year trends in cardiovascular risk factors in Finland. *Eur J Public Health* 2015; **25**(3): 539-46.

25. Leiviskä J, Sundvall J, Alfthan G, et al. What have we learnt about high-density lipoprotein cholesterol measurements during 32 years? Experiences in Finland 1980-2012. *Clin Chim Acta* 2013; **415**: 118-23.

26. Pinho-Gomes AC, Peters SAE, Thomson B, Woodward M. Sex differences in prevalence, treatment and control of cardiovascular risk factors in England. *Heart* 2020.

27. Tang R, An J, Bellows BK, Moran AE, Zhang Y. Trends in Hypertension Prevalence, Awareness, Treatment, and Control Among US Young Adults, 2003-2023. *Am J Hypertens* 2025; **38**(8): 551-9.

28. Carroll MD, Kit BK, Lacher DA, Shero ST, Mussolino ME. Trends in lipids and lipoproteins in US adults, 1988-2010. *Jama* 2012; **308**(15): 1545-54.

29. NCD Risk Factor Collaboration (NCD-RisC). Long-term and recent trends in hypertension awareness, treatment, and control in 12 high-income countries: an analysis of 123 nationally representative surveys. *Lancet* 2019; **394**(10199): 639-51.

30. NCD Risk Factor Collaboration (NCD-RisC). Worldwide trends in blood pressure from 1975 to 2015: a pooled analysis of 1479 population-based measurement studies with 19.1 million participants. *Lancet* 2017; **389**(10064): 37-55.

31. Valsta LM, Tapanainen H, Sundvall J, et al. Explaining the 25-year decline of serum cholesterol by dietary changes and use of lipid-lowering medication in Finland. *Public Health Nutr* 2010; **13**(6a): 932-8.

32. Ford ES, Capewell S. Trends in total and low-density lipoprotein cholesterol among U.S. adults: contributions of changes in dietary fat intake and use of cholesterol-lowering medications. *PLoS One* 2013; **8**(5): e65228.

33. Kypridemos C, Bandosz P, Hickey GL, et al. Quantifying the contribution of statins to the decline in population mean cholesterol by socioeconomic group in England 1991 - 2012: a modelling study. *PLoS One* 2015; **10**(4): e0123112.

34. Jung M, Choo E, Lee S. Comprehensive Trends and Patterns of Antihypertensive Prescriptions Using a Nationwide Claims Database in Korea. *Clin Epidemiol* 2020; **12**: 963-75.

35. Kwon O, Lee S-Y, Kim B, Han K, Ahn J. Dyslipidemia Fact Sheet in South Korea, 2024. *J Lipid Atheroscler* 2025; **14**(3): 298-311.

36. Hsieh HC, Hsu JC, Lu CY. 10-year trends in statin utilization in Taiwan: a retrospective study using Taiwan's National Health Insurance Research Database. *BMJ Open* 2017; **7**(5): e014150.

37. Walley T, Folino-Gallo P, Stephens P, Van Ganse E. Trends in prescribing and utilization of statins and other lipid lowering drugs across Europe 1997-2003. *Br J Clin Pharmacol* 2005; **60**(5): 543-51.

38. Rouette J, McDonald EG, Schuster T, Brophy JM, Azoulay L. Treatment and prescribing trends of antihypertensive drugs in 2.7 million UK primary care patients over 31 years: a population-based cohort study. *BMJ* 2022; **12**(6): e057510.

39. Aekplakorn W, Chariyalertsak S, Kessomboon P, et al. Trends in hypertension prevalence, awareness, treatment, and control in the Thai population, 2004 to 2020. *BMC Public Health* 2024; **24**(1): 3149.

40. Sophiea MK, Zaccardi F, Cheng YJ, Vamos EP, Holman N, Gregg EW. Trends in all-cause and cause-specific mortality by BMI levels in England, 2004-2019: a population-based primary care records study. *Lancet Reg Health Eur* 2024; **44**: 100986.
